# Longitudinal study on circulating miRNAs in patients after lung cancer resection

**DOI:** 10.18632/oncotarget.4322

**Published:** 2015-05-29

**Authors:** Petra Leidinger, Valentina Galata, Christina Backes, Cord Stähler, Stefanie Rheinheimer, Hanno Huwer, Eckart Meese, Andreas Keller

**Affiliations:** ^1^ Department of Human Genetics, Saarland University, Homburg, Germany; ^2^ Chair for Clinical Bioinformatics, Saarland University, Saarbrücken, Germany; ^3^ Siemens AG, Strategy Division, Erlangen, Germany; ^4^ Department of Cardiothoracic Surgery, Heart Center, Völklingen, Germany

**Keywords:** microRNA, plasma, lung cancer, metastases, follow-up

## Abstract

There is an urgent need of comprehensive longitudinal analyses of circulating miRNA patterns to identify dynamic changes of miRNAs in cancer patients after surgery. Here we provide longitudinal analysis of 1,205 miRNAs in plasma samples of 26 patients after lung cancer resection at 8 time points over a period of 18 months and compare them to 12 control patients. First, we report longitudinal changes with respect to the number of detected miRNAs over time and identified a significantly increased number of miRNAs in patients developing metastases (*p* = 0.0096). A quantitative analysis with respect to the expression level of the detected miRNAs revealed more significant changes in the miRNA levels in samples from patients without metastases compared to the non-cancer control patients. This analysis provided further evidence of miRNA plasma levels that are changing over time after tumor resection and correlate to patient outcome. Especially hsa-miR-197 could be validated by qRT-PCR as prognostic marker. Also for this miRNA, patients developing metastases had levels close to that of controls while patients that did not develop metastases showed a significant up-regulation.

In conclusion, our data indicate that the overall miRNome of a patient that later develops metastases is less affected by surgery than the miRNome of a patient who does not show metastases. The relationship between altered plasma levels of specific miRNAs with the development of metastases would partially have gone undetected by an analysis at a single time point only.

## INTRODUCTION

The fact that most non-small cell lung cancer (NSCLC) patients are diagnosed in late stages with locally advanced or metastatic disease, makes NSCLC to one of the most deadly cancers with a 5-year overall survival rate of around 17% [1]. The detection and resection of NSCLC in early stages is of profound relevance as it is normally correlated with a substantially improved prognosis [2]. Nevertheless, the rate of recurrences and metastases is high, even in early stage lung cancers. In a study on more than 900 patients who underwent early NSCLC curative-intend resection about 13% of patients developed lung cancer recurrence and 78% of the recurrences occurred within two years after operation. [3]. Disseminated tumor cells can already be present in early tumor stages before resection but they are not detected by conventional histopathology analysis and tumor staging and are often staged as N0 tumors [4]. The overall incidence of recurrence lies around 30% to 70% depending on lung cancer stage [5-7]. To improve the overall survival rate there is an urgent need for the identification of new prognostic factors. Second, intensive follow-up is important to reduce lung cancer mortality by the detection of recurrences after surgery [8].

MicroRNAs (miRNAs) found in body fluids indicate a high impact as diagnostic and prognostic biomarker as they play a crucial role in many cellular processes by regulating an extended number of target genes due to mRNA degradation or inhibition of the translation of the target mRNA [9, 10]. Until now, substantial effort has been undertaken to identify disease-specific miRNA profiles suitable for early diagnosis of diseases and to predict disease outcome [11, 12]. While many case-control studies have revealed a plentitude of miRNAs as biomarker candidates, dynamic changes over extended time periods have not been explored for the majority of them. Most respective studies are either limited in the number of time-points, patients, or considered miRNAs.

An analysis of the physiological fluctuation of serum miRNA profiles of samples taken from 12 healthy individuals over varying time periods up to 17 months revealed miRNA profiles that showed a high correlation and no significantly differentially expressed miRNAs were found. This suggests that circulating miRNAs are stable over extended time periods in healthy individuals [13]. Thus, changes in the overall abundance of circulating miRNAs due to a certain disease make them to good biomarker candidates. Changes of few miRNAs have for example already been monitored in a kinetic study over months in serum of 15 colorectal cancer patients [14]. However, just few studies investigate circulating miRNA profiles for changes between lung cancer samples collected before and after cancer resection [15, 16]. We recently performed a first follow-up study on lung cancer patients over a period of 18 months after lung cancer resection to identify miRNA signatures that possibly contribute to disease monitoring [17]. Although we analyzed 8 different time points and profiled a large number of miRNAs, a major limitation of this study was the small cohort size of only 5 patients. We now screened 26 patients for up to 8 time points – prior to surgery, following surgery and subsequently in 3 months intervals. Additionally, we compare the miRNAs identified in plasma of the lung cancer patients to those measured in samples obtained from12 control patients that suffered from other non-cancer lung diseases. Altogether, 215 single complex miRNA profiles have been generated using a microarray approach. Since one key criterion for a potential application in clinics beyond technical sensitivity and specificity is the reproducibility of measurements we applied a microarray technology that has been described to be most reproducible among 12 commonly used commercial systems [18]. Following background correction, adjustment for batch effects and normalization, bioinformatics analysis was applied in order to identify and validate the most relevant regulated miRNAs towards their usefulness as potential prognostic lung cancer biomarker.

## RESULTS

The main aim of our study was to provide a comprehensive longitudinal analysis of circulating miRNAs in plasma of lung cancer patients following surgery to identify miRNAs with prognostic relevance. In detail, we analyzed 1,205 different miRNAs in 26 lung cancer patients over a period of 18 months measured at 8 time points including one time point prior and up to seven time points after cancer resection. The expression profiles of the lung cancer samples were compared to 12 patients suffering from other non-cancer lung diseases that served as control.

### miRNA repertoire in lung cancer patients over time and in non-cancer controls

**Figure 1:**
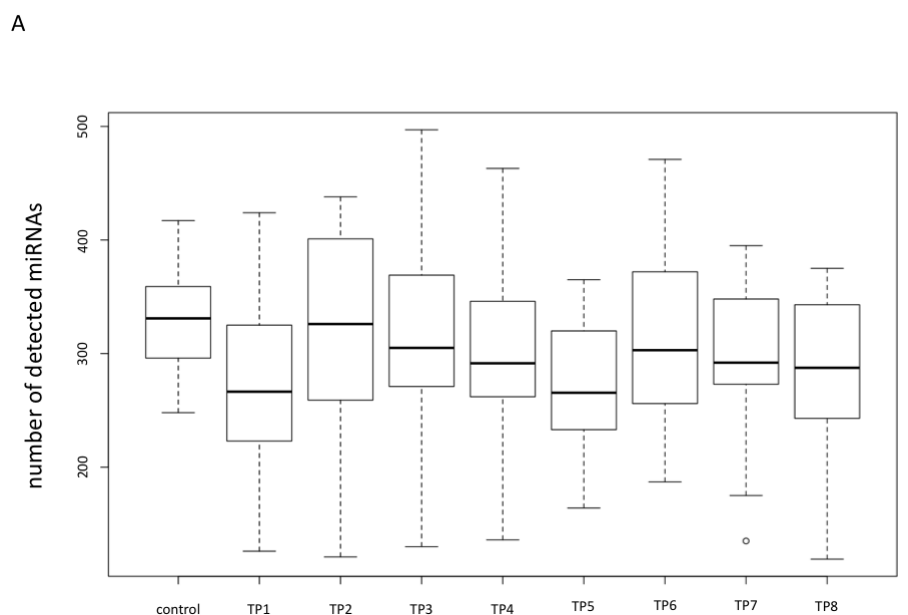

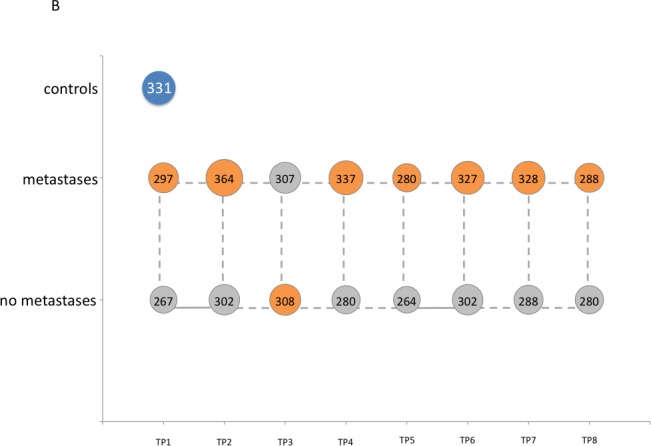
Comparisons of the overall numbers of detected miRNAs **A.** Box plot showing the overall number of detected miRNAs for all non-cancer control samples and all lung cancer samples for each time point separately. **B.** Bubble plot indicating the overall number of detected miRNAs for the non-cancer control patients as well as for the lung cancer patients that developed metastases and the lung cancer patients that did not develop metastases for each time point, separately.

We determined for all lung cancer patients and each time point (TP) and for all controls the average number of miRNAs detected in each sample (Figure 1). The samples obtained from lung cancer patients contained independent of the time point a lower number of miRNAs compared to the non-cancer controls (on average 295 miRNAs were detected in lung cancer samples and 331 in control samples). However, only for TP5 the difference between the average number of miRNAs detected in the lung cancer plasma samples compared to controls was significant (adjusted *p*-value 0.025). Lung cancer samples collected at TP2 showed with an average number of 321 detected miRNAs the lowest difference compared to controls (adjusted *p*-value of 0.67). Since the analysis of plasma samples obtained from the same individuals at different time points also enables paired testing of consecutive time-points we investigated whether significant changes of miRNA levels can be observed over time. Here, we found the most significant differences average number of detected miRNAs between TP1 and TP2 (raw *p*-value 0.019) and between TP5 and TP6 (raw *p*-value 0.016).

We also asked whether the miRNA repertoire differs in its quantity between lung cancer patients developing a metastases compared to those not developing metastases. The results are presented in Figure 1B, where for both groups and all time points the average number of miRNAs are shown. For patients not developing metastases we observed significant increase of miRNA repertoire from TP1 to TP2 and TP5 to TP6. For the other patients no significant alterations in the miRNA number were discovered, although the differences between different time points seems to be higher. But, as the standard deviation for the number of detected miRNAs is higher in the samples obtained from patients that developed metastases, the differences were not significant. But generally, we observed larger miRNA repertoire of patients that develop metastases. Independent of the time point we observed 286 miRNAs for patients not developing metastases while the remaining patients revealed 316 miRNAs (two-tailed unpaired *t*-test *p*-value of 0.0096). Interstingly, the analysis of the 12 non-cancer control samples revealed 331 detected miRNAs.

For the following quantitative analysis we only focused on the 485 miRNAs that were expressed in at least 5% of all tested 215 individual samples.

### Correlation analysis of miRNA pattern over time for all lung cancer patients combined and the non-cancer control patients

**Figure 2:**
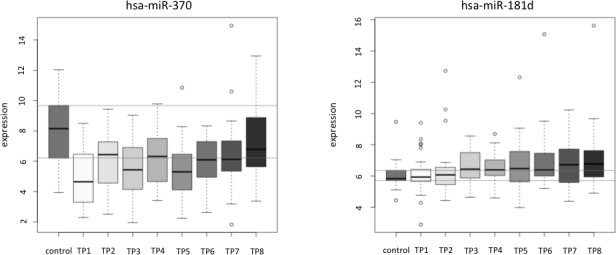
Examples of the correlation analysis of miRNA pattern over time for single miRNAs shown for each patient separately hsa-miR-370 is an example for a positive correlated miRNA and has-miR-181d is an example for a negative correlated miRNA. In both figure panels the y-axis shows the normalized expression values (in log scale) and the x-axis indicates the time points 1 to 8.

To identify miRNAs that show an overall increase or decrease from the first to the last measurement we first calculated pair-wise significance values between the miRNA profiles of the 12 non-cancer controls and the profiles of the 26 lung cancer patients for each of the time points using two-tailed unpaired *t*-test. Next, we correlated the logarithm of the significance values obtained by the two-tailed unpaired *t*-test with the rank of the time points. We discovered 6 negatively and 28 positively correlated miRNAs (raw *p*-value of correlation below 0.05). These 34 miRNAs with correlation values, *p*-values and upper and lower confidence interval are provided in Table 1. Notably, a strong negative correlation indicates that the respective miRNA is not de-regulated in samples from lung cancer patients at the beginning of the time course (high *p*-values at early time points) but shows increasing difference in miRNA plasma levels from non-cancer controls over time (low *p*-values at the end). In contrast, strong positive correlation indicates that the respective miRNA is de-regulated at the beginning (low *p*-values at early time points) but shows decreasing difference to the non-cancer control miRNA level over time (high *p*-values at the end). The miRNAs with correlation values around zero do not show increasing or decreasing significance over time but are rather constantly expressed. Although no miRNA was significant following adjustment, we observed a substantial increased number of miRNAs significant prior to adjustment as compared to the expected number of 24 random miRNAs. Figure 2 presents exemplarily the miRNA plasma levels of hsa-miR-370 (representative for positive correlated miRNAs) and hsa-miR-181d (representative for negative correlated miRNAs).

**Table 1:** Correlation analysis of miRNA pattern over time for all lung cancer patients combined and the non-cancer control patients

miRNA	Correlation	p-Value	Lower CI	Upper CI
hsa-miR-181d	−0.95	0.0003	−0.99	−0.80
hsa-miR-670	−0.81	0.0139	−0.95	−0.38
hsa-miR-196b	−0.80	0.0179	−0.95	−0.34
hsa-miR-3148	−0.78	0.0219	−0.95	−0.30
hsa-miR-762	−0.76	0.0290	−0.94	−0.25
hsa-miR-539	−0.74	0.0342	−0.93	−0.22
hsa-let-7d*	0.71	0.0467	0.16	0.93
hsa-miR-484	0.72	0.0432	0.17	0.93
hsa-miR-3663-5p	0.72	0.0429	0.18	0.93
hsa-miR-183	0.73	0.0385	0.20	0.93
hsa-miR-17*	0.74	0.0362	0.21	0.93
hsa-let-7c	0.74	0.0345	0.22	0.93
hsa-miR-548c-5p	0.75	0.0326	0.23	0.94
hsa-miR-3189	0.75	0.0325	0.23	0.94
hsa-miR-20b	0.75	0.0322	0.23	0.94
hsa-miR-29b	0.75	0.0321	0.23	0.94
hsa-miR-224	0.75	0.0317	0.24	0.94
hsa-miR-501-5p	0.76	0.0301	0.25	0.94
hsa-miR-20a	0.76	0.0280	0.26	0.94
hsa-miR-370	0.76	0.0272	0.26	0.94
hsa-miR-18a	0.78	0.0226	0.30	0.94
hsa-miR-532-5p	0.78	0.0220	0.30	0.95
hsa-miR-1915	0.78	0.0217	0.31	0.95
hsa-miR-146b-5p	0.78	0.0212	0.31	0.95
hsa-miR-3654	0.80	0.0177	0.34	0.95
hsa-miR-451	0.80	0.0161	0.36	0.95
hsa-miR-374a	0.81	0.0145	0.38	0.95
hsa-miR-3180-3p	0.84	0.0093	0.45	0.96
hsa-miR-10b*	0.84	0.0087	0.46	0.96
hsa-miR-184	0.85	0.0075	0.48	0.96
hsa-miR-141	0.85	0.0071	0.49	0.96
hsa-miR-4281	0.86	0.0061	0.51	0.97
hsa-miR-454	0.88	0.0038	0.57	0.97
hsa-miR-301a	0.88	0.0037	0.57	0.97

CI = confidence interval

### Correlation analysis of miRNA pattern over time for single lung cancer patients

Beside the analysis of the miRNA changes for all patients combined, our study set-up also allows the analysis of the miRNA time courses for single patients. We calculated for each patient and each miRNA separately correlation values between miRNA expression and time-points and estimated the significance values for the respective correlation. We excluded miRNAs that did not revealed significant correlation for at least 10% of all patients. For the remaining miRNAs we calculated in how many patients a miRNA was positive or negative correlated over time and calculated the difference of positive and negative correlated patients for each of these miRNAs. We excluded miRNAs for which the number of patients with a positive correlation largely corresponded to the number of patients with a negative correlation. As threshold we considered only miRNAs with a difference of at least 30% between positively and negatively correlated patients. We thereby identified 16 miRNAs including 10 positively and 6 negatively correlated miRNA. Although the overall tendency of certain miRNA levels to either increase or decrease over time is in agreement with the results obtained with the expression levels for all patients combined, the data for the single patients show strong variability. These miRNAs indicate that although a general trend exists single patients substantially deviate from the general trend (see Supplemental Figure 1 and Supplemental Table 1).

### Identification of plasma miRNAs influenced by the development of metastases

To understand the changes of miRNA levels over time we related the changes to a clinical endpoint. This was also in keeping with the goal to discover prognostic miRNAs. Thus, we choose the development of metastases as endpoint and asked whether patients with metastases show different plasma miRNA levels as compared to patients without clinically identified metastases. To this end we calculated significance values for each time point with respect to the two groups of patients, i.e., patients developing metastases (*n* = 8) versus patients not developing metastases (*n* = 18). At the time point directly before cancer resection (TP1) we found 25 plasma miRNAs that showed significantly different plasma levels between patients with and those without metastases (non-adjusted *p*-value < 0.05). At TP2, i.e., shortly after resection four of the 25 miRNAs were still significant, but in total 18 miRNAs showed significantly different abundance (non-adjusted *p*-value < 0.05). The highest number of 40 miRNAs with significantly different plasma levels between patients that developed lung cancer metastases and patients that did not develop metastases was obtained at TP3 around three moths after resection. At TP4 9 miRNAs were significant, at TP5 33 miRNAs, at TP6 13 miRNAs, at TP7 18 miRNAs, and at TP8 23 miRNAs. However, for the comparisons of the single time points no miRNAs remained significant after multiple testing. This fact is not necessarily due to decreased effect sizes for single time points but may reflect the comparably small cohort size.

We also performed a more general comparison of all expression values independent of the time point and compared all lung cancer samples to the non-cancer controls. To evaluate the patterns we considered both, raw and adjusted *p*-values. Of the 485 analyzed miRNAs, 139 were significantly altered between cancer patients and non-cancer controls, of which 56 remained significant following adjustment. Lowest *p*-values of below 10^−10^ were found for hsa-miR-3647-5p and hsa-miR-144. In the comparison of non-cancer controls versus lung cancer patients that did not develop metastases 138 miRNAs were significant (55 following adjustment) and 125 miRNAs for the comparison of controls versus metastases developing patients (41 following adjustment). Importantly, we also discovered 131 miRNAs that were significantly altered between patients that developed metastases and those that did not (38 following adjustment). Here, the highest significance was reached for hsa-miR-197 (*p* = 3×10^−7^). This miRNA was also significant in the previously mentioned comparison of controls compared to lung cancer patients that did not develop metastases (*p* = 0.004) while it was not significantly differentially regulated for controls versus patients that developed metastases (*p* = 1). The most significant changes (*p* < 0.05) for this miRNA were found at TP2, TP3, and TP5. Another miRNA, hsa-miR-630 was even significant in four time points, i.e., TP1, TP2, TP4, and TP6. Hsa-miR-130b was the most significant miRNA that showed larger deviation of lung cancer patients that developed metastases from controls (*p* = 0.0004) than patients that did not develop metastases (*p* = 0.083).

The full list of the 485 miRNAs with the expression data and the non-adjusted *p*-values is provided in Supplemental Table 2.

**Figure 3:**
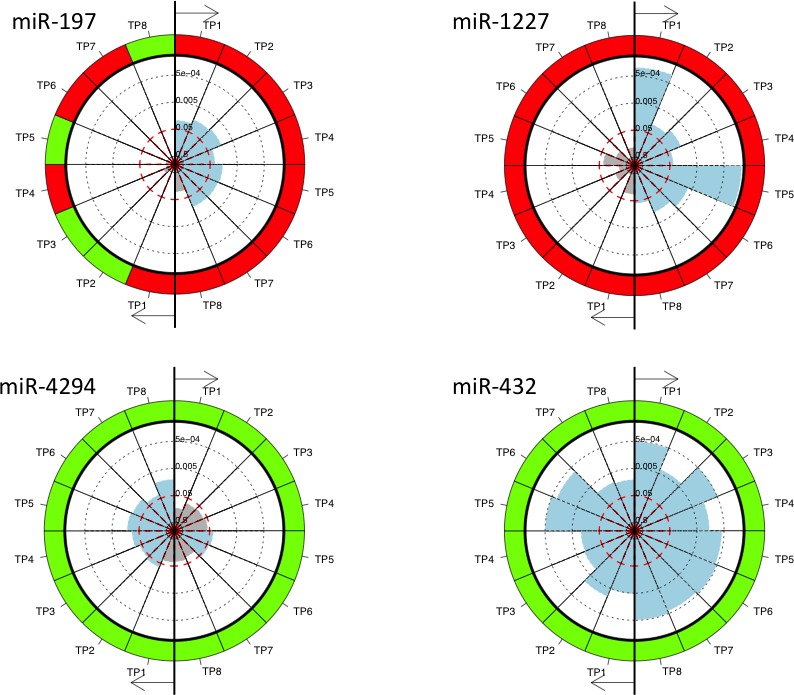
The pie charts for miRNAs significant in the comparison of non-cancer controls and the lung cancer samples collected at the different time points and for patients with and without metastases separately MiRNAs were measured at eight different time points. The time points are numbered TP1 to TP8 and each time point TP1 to TP8 is compared to the non-cancer controls. The right part of each pie chart represents the comparison between non-cancer controls and lung cancer patients without metastases and the left part of the pie chart represents the comparison between non-cancer controls and lung cancer patients with metastases. Each sector represents one comparison with the color of the outer ring indicating down-regulation (green) or up-regulation (red) at the respective time point compared to non-cancer controls. The inner part of the circle indicates the significance values with blue shaded sectors representing significant differences and the grey sectors not significant differences.

To compare the metastases and non-metastases group directly to non-cancer controls, we calculated for each miRNA the *p*-values for the comparison of its expression value in plasma samples collected from lung cancer patients that developed metastases and those that did not at each time point versus its expression value in plasma samples from non-cancer controls. In total, 139 miRNAs were significant in the comparison of the samples obtained before resection (TP1) from lung cancer patients that did not develop metastases with the non-cancer controls, but only 98 miRNAs in the comparison of the samples obtained before resection (TP1) from lung cancer patients that developed metastases with the non-cancer controls. We observed the same trend in the comparison of the non-cancer controls and the lung cancer samples obtained shortly after resection (TP2). Here 92 miRNAs were significant in the group of patients that did not develop metastases and only 72 in the group of patients that developed metastases. Figure 3 shows the above mentioned comparions for selected miRNAs as pie charts and all data are provided in Supplemental Table 3. The miRNA hsa-miR-197 was significantly up-regulated at 7 time points (non-adjusted *p*-values) for the group of patients that did not develop metastases while not in the group of patients that developed metastases. Similarly, hsa-miR-1227 was constantly up-regulated, however, again just the patients without metastases were significant. In contrast, hsa-miR-4292 was more significantly down-regulated in the group of patients that developed metastases as compared to the group of patients that did not develop metastases.

We next focused only on the samples obtained from lung cancer patients and compared the samples collected before surgery at TP1 with samples from each other time point after surgery (TP2 to TP8) resulting in 7 comparison. The calculated *t*-test *p*-values for the respective comparisons are listed in Supplemental Table 4. This analysis was done separately for patients with and without metastases. For the patients without metastases the comparison of the sample drawn before cancer resection (TP1) and the sample obtained shortly after resection (TP2) revealed 103 significant miRNAs, while we found for the same comparison only 44 significant miRNAs in samples obtained from patients that developed metastases during follow-up and this trend was observed for all of the 7 comparisons. This indicates a trend to a more profound change in the miRNA pattern for samples of patients that did not develop lung cancer metastases.

We found 2 miRNAs including hsa-miR-454 and hsa-miR-3152 that were significantly deregulated in all seven comparisons, and 2 miRNAs including hsa-miR-181b and hsa-miR-98 that were significantly deregulated in 6 out of the 7 comparisons. Of those miRNAs deregulated in patients without metastases hsa-miR-454 was also significantly deregulated in two comparisons of patients with metastases and hsa-miR-98 in only one. In contrast, hsa-miR-3152 and hsa-miR-181b that were significantly deregulated in patients without metastases were not significantly deregulated in patients with metastases. Hsa-miR-454, hsa-miR-181b, and hsa-miR-98 were down-regulated at TP 2-8 compared to TP 1 in patients without metastases and hsa-miR-3152 showed significantly increased plasma levels at TP 2-8 compared to TP 1.

We also found one miRNA, namely hsa-miR-101, that showed significantly decreased plasma abundance in all seven comparisons of patients with metastases but was not significantly deregulated in the comparisons of patients without metastases. Has-miR-186 was still significant in 6 of 7 comparisons of patients without metastases, but also in two comparisons of patients with metastases. Both miRNAs were down-regulated at time points 2-8 compared to time point 1 in patients with metastases.

In sum, the data demonstrate that miRNA changes over time can be related to clinical end points like the development of metastases and that effects are largest 3 months following surgery.

### qRT-PCR validation of selected miRNAs

In the previous section we described miRNAs identified by microarray that are correlated to lung cancer and that have a potential prognostic impact. Using qRT-PCR we exemplarily measured the time courses consisting of the up to 8 time points for 4 patients, including 2 patients did not develop metastases (patients J and P) and two patients that that later on developed metastases (patients V and Z) and three miRNAs (hsa-miR-197, hsa-miR-130b, hsa-miR-762). Additionally, the 12 samples from non-cancer control patients were analyzed using qRT-PCR. One very interesting and potentially prognostic miRNA was hsa-miR-197 as this miRNA was significantly up-regulated in 7 of 8 time points (TP1 to TP7) in plasma of patients that did not develop metastases compared to plasma of non-cancer control patients but it was similarly abundant in plasma from lung cancer patients that developed metastases and in plasma of non-cancer control patients. Investigating the miRNA abundance using qRT-PCR at the different time points for the four patients and 12 controls we were able to reproduce these results. Although the considered cohorts were comparably small, the difference between cases and controls was significant (0.004). While considering all measurements without respect to the time points slightly missed the alpha level of 0.05 (*p* = 0.059), the paired analysis of the time course for both lung cancer patient groups (with metastases and without metastases) was significant (*p* = 0.025). In detail, the time course of all patients matched in general well between microarray and qRT-PCR. The most significant miRNA where the mean expression value of all samples from patients of the metastases group was lower than the mean expression value of all samples from patients of the non-metastases group and all samples from non-cancer controls showed the highest mean value was hsa-miR-130b. Although the time courses of the analyzed patients generally showed a high concordance with a median correlation value of 0.75 for all patients and the controls we were not able to reproduce the lower expression of this miRNA in patients that developed metastases. Especially the time course of patient Z for hsa-miR-130b plasma levels that was measured by microarray could not be validated completely by qRT-PCR. However, the higher plasma levels in non-cancer control samples were indeed validated. As third candidate we picked hsa-miR-762, which shows a similar behavior in the mean expression values according to microarray as hsa-miR-130b. Here, we observed for two patients deviations in the time course as compared to array measurements (patients P and V).

In sum, for patient J all three miRNAs were validated, while for the other patients two of three miRNAs were reproduced. For patients P and V hsa-miR-762 diverged and for patient Z hsa-miR-130b.

In Supplemental Figure 2A-2L a comparison of the microarray data and the qRT-PCR data for the up to 8 samples for the four different lung cancer patients and the three miRNAs is shown.

As there is no endogenous smallRNA or miRNA that can reliably serve as “housekeeping gene” that is stably detected/abundant in serum or plasma [19] we used as normalizer the miRNA mimic syn-cel-miR-39, that was spiked into the plasma sample before RNA isolation. Interestingly, this synthetic miRNA cannot only serve as normalizer but can also be used to control the extraction process. In the present study the mean Ct value was 21±3.13.

## DISCUSSION

There is an undisputable requirement for molecular tests to assist in the diagnosis, prognosis and prediction of cancers including lung cancer. Although histological evaluation of tumor tissues from biopsies will at least for the near future remain the ‘gold standard’ of diagnosis, these samples necessarily represent only a single time point in the overall tumor development. Blood based tests open the possibility to monitor the course of tumor development. Currently, there are, however, only few blood based markers in clinical use including CA125 for ovarian cancer, CA19-9 for pancreatic cancer, CEA for colon cancer, and PSA for prostate cancer [20]. These established markers have, however, rather limited accuracy, which can be improved by longitudinal measurements as shown for PSA where continuously increasing levels strongly indicate a carcinoma [21]. As of now, there is no biomarker established for lung cancer in a screening setting.

Beside the need to have measuring from different time points of tumor development, there is a need to have biomarkers that do not rely on the measuring of a single kind of molecule like the aforementioned markers. Since combinations of different molecules can be more accurate and are likely to be more robust than single-molecule markers, an increasing number of studies aimed at identifying marker signatures. Notably miRNA signatures appear of especial interest due to their rather high stability in body fluids. Since the first description of miRNAs in serum of patients with diffuse large B cell lymphoma, blood born miRNAs have been related to tumor diagnosis and prognosis [19, 22]. The majority of these studies, however, analyzes miRNA pattern at one time point only. In addition, the analysis of circulating miRNAs has some methodological challenges. As these challenges are exhaustively summarized in a recent review article by Moldovan et al. [23] we do not want to further discuss them here in more detail. Nevertheless, Moldovan et al. [23] found out that there are many studies comparing different biological fluids side-by-side and find little or no difference in extracellular miRNA quantification. Interestingly, higher concentrations were consistently found in sera and a possible explanation for that might be that platelets, that contain a wide spectrum of miRNAs, may release their content into the serum during coagulation. This is one argument for the use of plasma samples. But, we are aware of the disadvantages of heparinized plasma samples in terms of the effect of heparin on downstream applications. However, as for the current study only heparinized plasma was available we established a protocol that includes a heparin digestion step to isolate RNA that could be used for downstream analyses like microarray and qRT-PCR. We also checked for RNA extraction efficiency by using a synthetic miRNA mimic (syn-cel-miR-39).

Our study on 1,205 different miRNAs in 26 lung cancer patients over a period of 18 months measured at up to 8 time points is the most comprehensive longitudinal analysis of miRNA signatures in cancer patients. This is the follow-up of a proof-of-principle study that we published previously [17]. However, our previous study focused only on the changes of the plasma miRNA profile over time after surgery without the comparison with non-cancer control samples. In addition, we compared our microarray data with circulating miRNAs that were previously described in literature as deregulated in lung cancer and found 11 of 35 published miRNAs detected in all samples prior to surgery. In the present study these 11 miRNAs were also detected in all analyzed plasma samples obtained from lung cancer patients at TP1, i.e., prior to surgery. However, these 11 miRNAs were also detectable in all of the analyzed samples from non-cancer controls and there was no difference in expression level after adjustment between both groups. These findings indicate that the respective miRNAs are not well suited as reliable diagnostic biomarkers for lung cancer.

For the correlation analysis of the single patients and time points, we identified in the present study 6 negative correlated miRNAs and 10 positive correlated miRNAs. The comparison of the correlated miRNAs for each lung cancer patient between our former study and the present study is complicated by the different analysis methods. In the former study, we considered the miRNAs with positive or negative correlation for each patient, respectively. In the present study, we also calculated the correlation of each miRNA for each patient but excluded those miRNAs that do not show a general trend to positive or negative correlation. Thus the list of miRNAs is smaller and we find only an overlap of two miRNAs. The miRNA hsa-miR-24 was negatively correlated in patient B in the former study and is also negatively correlated in most of the 26 patients, including patient B analyzed in the present study. The miRNA hsa-miR-1202 was negatively correlated in patient D in the former study but in the present study it is positively correlated in the majority of patients. Interestingly, when only considering patient D it shows a negative correlation.

A correlation analysis of the plasma miRNAs identified in samples of all lung cancer patients combined and the non-cancer control patients revealed 6 negative correlated miRNAs that showed no deregulation of the lung cancer samples at the beginning but increasing difference from the non-cancer control samples in expression over time and 28 positive correlated miRNAs that were deregulated in lung cancer samples at the beginning but levels to the non-cancer control expression level over time. As control samples were not included in our former study, a comparison for this analysis was not possible.

Overall, our data show that miRNA levels are changing over time after tumor surgery and that these changes are not necessarily fluctuating around a median value but can have a clear tendency to either increase or decrease. Since circulating miRNA profiles in healthy individuals seem to be rather stable over time, the observed changes in our study are likely to be disease related [13]. This idea of miRNA pattern changing in the course of a disease under treatment is consistent with previous reports on changes in the abundance of circulating miRNAs between samples collected prior and after radiochemotherapy of head and neck cancer patients [24]. A study on 4 miRNAs in 82 lung carcinoma patients identified altered serum levels in samples obtained before surgery and samples obtained 10 days after surgery [16]. Likewise, 90 miRNAs were analyzed in plasma obtained before and after tumor removal in 32 squamous cell lung cancer patients [15].

It remains the question of the biological meaning of the increasing or decreasing miRNA levels. In a longitudinal expression analysis of 3 miRNAs on serum samples of 15 patients with colorectal cancer over a period of three years post surgery or after chemotherapy, the authors found that serum levels of miRNAs returned to normal levels after cancer resection or chemotherapy in the samples from patients with good prognosis [14]. However, our data for single patients show a strong fluctuation between the different time points making a biological interpretation difficult. The specific variations of miRNA levels over time in single patients may be due to a combination of factors that are related to the physiological state and the specific treatment response of each patient and it will be highly demanding to define the specific influence of any of these factors on a specific miRNA plasma level.

Nevertheless, variations of miRNA levels over time might be related to clinical endpoints such as the development of metastases. For example, we found a higher number of miRNAs that were significantly changed in plasma levels between the time point TP1 before and the time points TP2 to TP8 after surgery in patients that did not develop metastases during the follow-up compared to the patients that developed metastases. These relationships between miRNA plasma levels would have gone undetected by an analysis at a single time point only. Besides a potential diagnostic value of altered miRNA levels, the changes observed in the present study might help to contribute to the understanding of systemic aspects associated with metastases. Overall, our data indicate more changes of miRNA levels in patients without metastases as compared to patients with metastases. This is not only true for the comparisons between the time point before surgery with all seven time points after surgery but also for the comparison between the time point before surgery with the first time point directly after surgery and also for the comparison of TP1 samples with the non-cancer control samples. As described above, the latter comparison identified more significantly altered miRNAs in patients without metastases as compared to patients with metastases, possibly indicating that the miRNome of patients that developed metastases is more similar to the miRNome of non-cancer controls than the miRNome of patients that do not develop metastases during the follow-up.

Nevertheless, we are aware of the limitations of the present study and thus do not intend to over-interprete our findings. For example we want to point out that we analyzed groups of different sizes, i.e., the group of patients that did not develop metastases encompassed 18 patients while we obtained only blood of eight patients that later on developed metastases. In addition, as discussed above, the choice of the right blood collections system is very crucial for downstream analyses. Furthermore, the here presented results have to be confirmed in larger patient cohorts in future studies.

Although highly hypothetical, our data may indicate that the overall miRNome of a patient that later develops metastases is less affected by surgery than the miRNome of a patient that is not prone to develop metastases. An overall stability of the miRNome has previously been reported for healthy adults by MacLellan et al. [13]. Possibly, such an overall stability can also be found for a pathological status and changes of the miRNA pattern would indicate either a treatment success or a significant deterioration of the patients' health.

## MATERIALS AND METHODS

### Study population

We obtained blood from 26 different NSCLC patients. Blood of lung cancer patients was drawn directly before tumor resection (TP1), around two weeks after tumor resection (TP2) and then around three months (TP3), six months (TP4), nine months (TP5), 12 months (TP6), 15 months (TP7) and 18 months (TP8) after tumor resection. From 3 patients we obtained only blood from 7 time points and from one patient we obtained blood only from 6 time points. In a follow-up of 4 years, 18 patients were free of metastases or recurrences. In addition, we obtained blood from 12 patients from the same clinic that did not suffered from lung cancer but from other non-tumor lung diseases. Blood of all patients was drawn in Lithium-Heparin monovettes (Sarstedt). Plasma was isolated by centrifugation at 3000 rpm for 10 min and stored at −80°C until use. Samples were collected with patient informed consent. The local Ethics Committee approved the study (Ärztekammer des Saarlandes, 01/08). Patient details are provided in Supplemental Table 5.

### Isolation of total RNA including miRNA

As it is well known that heparin is co-purified with RNA and can interfere with downstream applications the RNA was isolated using an optimized protocol for Lithium-Heparin plasma samples as previously described [17]. We first treated 100μl plasma with 10μg Heparinase I (Sigma) and 100U RNaseOUT^TM^ (Life Technologies) and incubated the mixture at 25°C for 1 hour. Nuclease free water (Life Technologies) was added to a final volume of 250μl. A total of 750μl TRIzol®LS (Life Technologies) was added and incubated at RT for 5 min. Then, 20μg glycogen, 5μl spike-in miRNA (miRNA mimic syn-cel-miR-39, 5nM, Qiagen) and 200μl chloroform were added, vigorously vortexed, and incubated for 3 min at RT. After centrifugation at 14000rpm and 4°C, the aquaeous phase was transferred into a new tube and RNA was precipitated with 1,5 volumes of 100% ethanol. RNA was then isolated using the miRNeasy Mini Kit (Qiagen) according to manufacturers instructions but with the use of the RNeasy Mini Elute column to allow for a reduced elution volume of 15μl. RNA concentration was measured using the Nanodrop2000 (ThermoScientific) and RNA quality was checked using the Bioanalyzer2100 and the Small RNA Kit (Agilent).

### Quantitative real time PCR (qRT PCR)

Using quantitative Real Time-Polymerase Chain Reaction (qRT-PCR) with the miScript PCR System (Qiagen) we validated the microarray data for three exemplarily chosen miRNAs (hsa-miR-130b, hsa-miR-762, hsa-miR-197) and the follow-up samples from two patients that developed metastases and two patients that did not. In brief, 2 μl RNA was converted into cDNA using the miScript II Reverse Transcription Kit and the HiSpec Buffer according to the manufacturers' protocol. The PCR was performed with the miScript SYBR^®^ Green PCR Kit in a total volume of 20μl per reaction containing 2μl (1:5 diluted) cDNA according to the manufacturers' protocol on a StepOne Plus Real Time Analyzer (Life Technologies). Data were normalized using the spike-in miRNA mimic syn-cel-miR-39 (Qiagen).

### miRNA microarray

Microarray analysis has been performed according to manufacturer's instructions and as previously described using SurePrint G3 8×60K miRNA microarrays (Agilent) [20]. In brief, a total of 100 ng total RNA was processed using the miRNA Complete Labeling and Hyb Kit (Agilent) to generate fluorescently (cyanine-3) labeled miRNA. The microarrays, that contain 40 replicates of each of the 1,205 miRNAs of miRBase v16 (http://www.mirbase.org/ [26]) were hybridized with the labeled miRNA for 20 hours at 55°C and 20rpm. Microarray scan data were further processed using Feature Extraction software (Agilent). The Feature Extraction software removes outlier pixels, does statistics on inlier pixels of features and backgrounds. It further flags outlier features and backgrounds and subtracts the background from features. The output of the Feature Extraction Software provides the raw background corrected miRNA data (gTotalGeneSignal) and the present calls (IsGeneDetected). The results of the microarray analyses are freely available in the GEO database under accession number GSE68951 (http://www.ncbi.nlm.nih.gov/geo/).

### Biostatistics

All downstream biostatistics calculations have been carried out using the freely available statistical programming environment R. Two analysis strategies were carried out. First, we focused on the present calls, i.e. the information whether a miRNA *m* in patient *p* is expressed significantly above the background. This information was obtained from the Agilent feature extraction software according to manufacturers instruction and as sketched above. For all samples and miRNAs a binary matrix was build, where entries (*m,p*) equaled 1 if miRNA *m* was present in patient *p* and 0 otherwise. To minimize the noise contributed by low expressed markers we focused for all analyses on the miRNAs that were expressed above background in at least 5% of all tested samples. Using this definition, we performed all further analyses using 485 miRNAs.

In addition to the present call analysis, we likewise carried out a quantitative analysis of the expression level for the detected miRNAs. Since microarrays frequently show batch effects we tested and corrected for such technological bias. In detail, the identification and visualization of the batch effects was performed using the R-package “pvca”. The ComBat function of the R-package “sva” was then applied in order to account for the found batch effects in the data. Quantil normalization has been carried out using the Bioconductor “preprocessCore” package. Pairwise two-tailed *t*-tests have been carried out. Here, each time point following resection has been compared to the time point prior to resection. The results have been displayed as circular diagrams, specifically, time points are ordered clockwise such that each time point has an own sector. The shading of the sector denotes the significance, the further the shading, the more significant the respective time point is for this miRNA. Moreover, correlation between time-points and expression or significance values have been calculated using Pearson Correlation coefficient and a significance value for each correlation has been calculated using the “cor.test” function. For assessing the significance of correlations we calculated a statistic based on Pearson's product moment correlation coefficient, which follows a t-distribution. Additionally, 90% Confidence Intervals for the correlation are provided, which are calculated based on Fishers Z Transform. If not mentioned explicitly, *p*-values have been adjusted for multiple testing using the Benjamini-Hochberg approach.

## SUPPLEMENTAl MATERIAL FIGURES AND TABLES












