# Supplementary material for: Longitudinal study on circulating miRNAs in patients after lung cancer resection
Source: Oncotarget. 2015 May 29;6(18):16674–85. doi: 10.18632/oncotarget.4322 (PMC4599298; doi:10.18632/oncotarget.4322)
Supplement: Supplementary file 1 [file oncotarget-06-16674-s001.pdf]

Longitudinal study on circulating miRNAs in patients after lung cancer resection

Supplementary Material

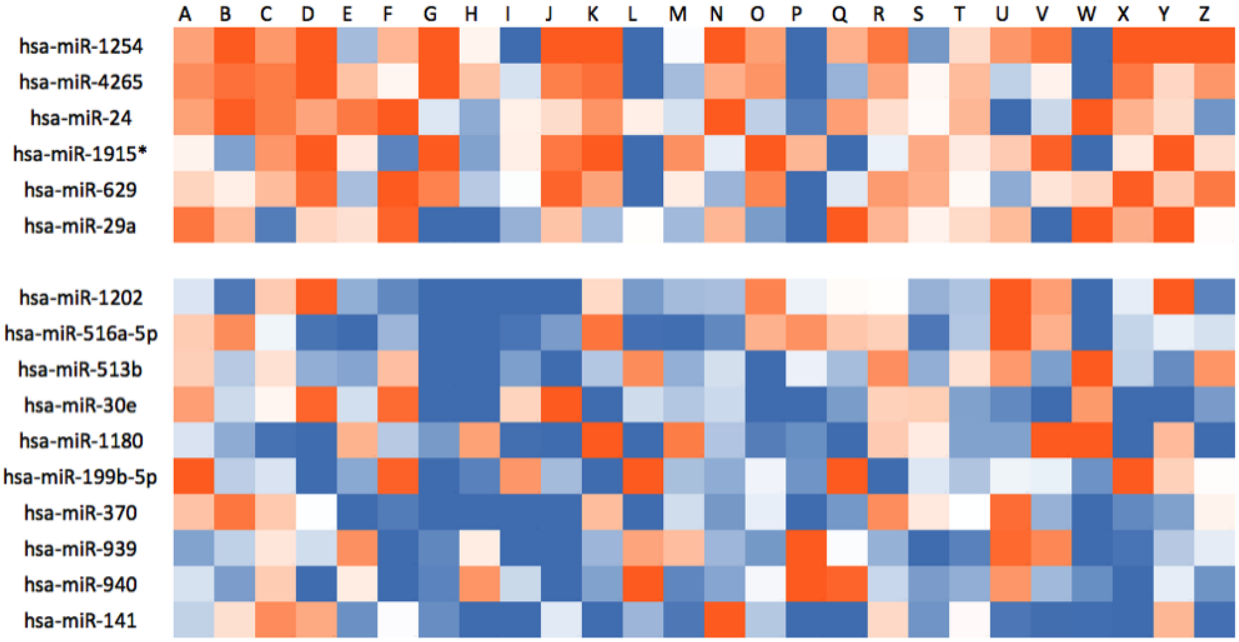

Supplemental Figure 1: Heatmap with 6 negative and 10 positive correlated miRNAs calculated for all patients separately over all time points. Orange color shadings indicate negative correlation and blue color shadings indicate positive correlation.

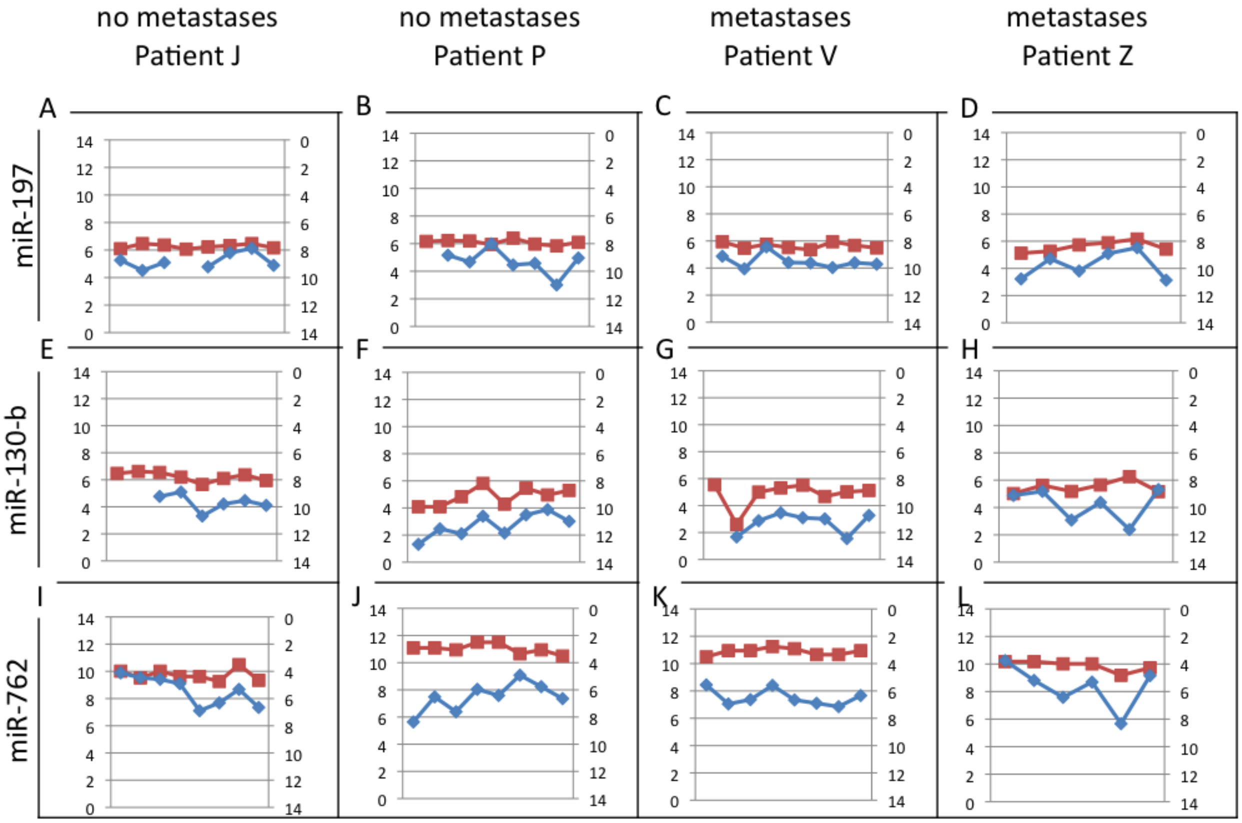

Supplemental Figure 2: Comparison of the microarray data (y-axis left = expression value) and the qRT-PCR results (y-axis left = ΔCt value). The x-axis indicates the 8 time points of blood collection.

Supplemental Table 1: Results of the correlation analysis of miRNA pattern over time for single lung cancer patients. In concordance to Supplemental Figure 1, blue color indicates positive correlation while orange color indicates negative correllation.

Supplemental Table 2: A summary of the expression values for the 485 detected miRNAs at the different time points for lung cancer patients that developed metastases and those that did not develop metastases separately and the significance values for the comparison of each time point between both lung cancer patient groups. The 485 miRNAs are listed in column A, column B shows the mean expression value for all non-cancer control samples, column C shows the mean expression value for all lung cancer samples, column D shows the mean expression value for the samples from lung cancer patients without metastases, column E shows the mean expression value for the samples from lung cancer patients with metastases, columns F-I show the p-values for the respective comparison listed in the header, columns L-AA show the mean expression values for the samples at the different time points for both lung cancer groups that were compared, and column K indicates how many of these comparisons were significant (p-values are listed in columns AB-AI).

Supplemental Table 3: A summary of the comparison of the miRNA levels in the non-cancer control samples and the lung cancer samples at each time point and for each of the two groups (metastases or no metastases) separately. The 485 miRNAs are listed in column A, column B shows the sum of all comparisons with significant p-value < 0.05, and columns C and D show the sum of comparisons with significant p-value < 0.05 for both lung cancer groups separately. Columns E-L show the p-values for the patients without metastases, columns M-T show the p-values for the patients with metastases. Columns U-AJ indicate whether the respective miRNA was down-regulated (1) or up-regulated (2) in the comparison.

Supplemental Table 4: A summary of the comparison of the miRNA levels in the the lung cancer samples collected before surgery (TP1) compared to the samples collected at each other time point (TP2-TP8) and for each of the two groups (metastases or no metastases) separately. The 485 miRNAs are listed in column A, column B shows the sum of all comparisons with significant p-value < 0.05, and columns C and D show the sum of comparisons with significant p-value < 0.05 for both lung cancer groups separately. Columns E-K show the p-values for the patients without metastases, columns L-R show the p-values for the patients with metastases. Columns S-AF indicate whether the respective miRNA was down-regulated (1) or up-regulated (2) in the comparison.

Supplemental Table 5: Patient details.
