# Supplementary material for: Longitudinal study on circulating miRNAs in patients after lung cancer resection
Source: Oncotarget. 2015 May 29;6(18):16674–85. doi: 10.18632/oncotarget.4322 (PMC4599298; doi:10.18632/oncotarget.4322)
Supplement: Supplementary file 2 [file oncotarget-06-16674-s002.pdf]

	A	B	C	D	E	F	G	H	I	J	K	L	M	N	O	P	Q	R	S	T	U	V	W	X	Y	Z
hsa-miR-1254	-0.268	-0.511	-0.300	-0.747	0.218	-0.209	-0.767	-0.030	0.690	-0.864	-0.600	0.900	0.009	-0.573	-0.278	0.596	-0.229	-0.404	0.343	-0.096	-0.305	-0.404	0.849	-0.526	-0.532	-0.614
hsa-miR-4265	-0.337	-0.423	-0.388	-0.894	-0.174	-0.023	-0.711	-0.168	0.093	-0.377	-0.427	0.673	0.217	-0.233	-0.313	0.753	0.249	-0.261	-0.019	-0.192	0.150	-0.033	0.509	-0.398	-0.113	-0.306
hsa-miR-24	-0.274	-0.492	-0.384	-0.263	-0.396	-0.769	0.079	0.277	-0.041	-0.096	-0.312	-0.043	0.097	-0.775	0.153	0.439	-0.278	-0.089	-0.013	-0.205	0.749	0.123	-0.752	-0.222	-0.097	0.350
hsa-miR-1915*	-0.031	0.310	-0.306	-0.621	-0.060	0.415	-0.585	0.306	-0.045	-0.400	-0.771	0.904	-0.326	0.059	-0.615	-0.205	0.514	0.046	-0.243	-0.057	-0.148	-0.478	0.612	-0.062	-0.888	-0.092
hsa-miR-629	-0.120	-0.045	-0.190	-0.439	0.207	-0.714	-0.369	0.173	0.008	-0.462	-0.266	0.833	-0.049	0.244	-0.358	0.810	0.073	-0.290	-0.233	-0.017	0.275	-0.076	-0.117	-0.490	-0.147	-0.395
hsa-miR-29a	-0.408	-0.191	0.439	-0.114	-0.085	-0.463	0.810	0.772	0.254	-0.169	0.211	-0.003	0.225	-0.206	0.328	0.867	-0.564	-0.212	-0.032	-0.103	-0.193	0.578	-0.521	-0.237	-0.666	-0.007
hsa-miR-1202	0.084	0.455	-0.150	-0.490	0.267	0.395	0.710	0.510	0.507	0.561	-0.105	0.333	0.219	0.207	-0.363	0.042	-0.010	-0.003	0.250	0.193	-0.719	-0.279	0.729	0.057	-0.720	0.417
hsa-miR-516a-5p	-0.145	-0.338	0.035	0.471	0.739	0.241	0.692	0.795	0.469	0.326	-0.412	0.484	0.495	0.398	-0.224	-0.321	-0.161	-0.131	0.458	0.180	-0.735	-0.226	0.845	0.134	0.051	0.096
hsa-miR-513b	-0.134	0.171	-0.084	0.266	0.299	-0.183	0.738	0.587	0.321	0.901	0.180	-0.337	0.263	0.100	0.587	0.045	0.214	-0.329	0.265	-0.080	-0.296	0.318	-0.817	0.149	0.382	-0.308
hsa-miR-30e	-0.279	0.119	-0.023	-0.459	0.103	-0.444	0.541	0.613	-0.120	-0.879	0.580	0.114	0.181	0.120	0.551	0.837	0.327	-0.129	-0.140	0.312	0.393	0.517	-0.295	0.559	0.760	0.329
hsa-miR-1180	0.091	0.275	0.478	0.556	-0.217	0.171	0.334	-0.272	0.491	0.784	-0.599	0.748	-0.386	0.187	0.435	0.373	0.612	-0.148	-0.054	0.307	0.308	-0.590	-0.601	0.914	-0.195	0.539
hsa-miR-199b-5p	-0.601	0.159	0.089	0.803	0.288	-0.473	0.519	0.422	-0.305	0.218	0.545	-0.635	0.206	0.271	0.036	0.357	-0.868	0.533	0.079	0.190	0.039	0.051	0.361	-0.904	-0.129	-0.004
hsa-miR-370	-0.171	-0.408	-0.154	0.007	0.636	0.441	0.784	0.530	0.819	0.654	-0.189	0.546	0.109	0.342	0.053	0.731	0.344	-0.333	-0.062	0.003	-0.437	0.250	0.507	0.390	0.312	-0.030
hsa-miR-939	0.304	0.149	-0.068	0.108	-0.324	0.678	0.402	-0.048	0.598	0.773	0.240	-0.261	-0.187	0.236	0.341	-0.563	0.009	0.254	0.812	0.423	-0.450	-0.350	0.721	0.474	0.175	0.062
hsa-miR-940	0.092	0.323	-0.142	0.775	-0.053	0.557	0.416	-0.308	0.123	0.722	0.309	-0.609	0.405	0.295	0.025	-0.729	-0.462	0.128	0.326	0.266	-0.305	0.225	0.376	0.591	0.059	0.359
hsa-miR-141	0.146	-0.087	-0.338	-0.250	0.368	0.008	0.378	0.876	0.791	0.065	0.498	0.240	0.457	-0.755	0.178	0.617	0.518	-0.107	0.357	-0.011	0.463	0.493	0.496	0.741	-0.205	0.484
