# Supplementary material for: Longitudinal study on circulating miRNAs in patients after lung cancer resection
Source: Oncotarget. 2015 May 29;6(18):16674–85. doi: 10.18632/oncotarget.4322 (PMC4599298; doi:10.18632/oncotarget.4322)
Supplement: Supplementary file 3 [file oncotarget-06-16674-s003.pdf]

A	B	C	D	E	F	G	H	I	J	K	L	M	N	O	P	Q	R	S	T	U	V	W
											mean expression values											
mirna	mean non-cancer control	mean lung cancer	mean lung cancer no metastases	mean lung cancer metastases	p non-cancer control vs lung cancer	p non-cancer control vs lung cancer no metastases	p non-cancer control vs lung cancer metastases	p lung cancer no metastases vs lung cancer metastases		sum significant TP no metastases vs metastases	mean TP1 no metastases	mean TP1 metastases	mean TP2 no metastases	mean TP2 metastases	mean TP3 no metastases	mean TP3 metastases	mean TP4 no metastases	mean TP4 metastases	mean TP5 no metastases	mean TP5 metastases	mean TP6 no metastases	mean TP6 metastases
hsa-miR-3647-5p	1.6591	4.7693	5.1075	3.9632	0.0000	0.0000	0.0000	0.0001		3	5.8290	3.4453	5.3885	3.2166	4.4477	5.1101	4.8493	3.8478	4.4390	4.4690	4.7164	3.7926
hsa-miR-144	111.1225	327.7126	321.2733	343.0596	0.0000	0.0000	0.0000	0.6483		0	184.8244	161.4185	199.2567	283.8735	506.7452	369.5489	326.2379	465.5349	313.4780	327.9026	431.1719	339.7301
hsa-miR-34b	3.3195	6.4190	6.4109	6.4384	0.0000	0.0000	0.0000	0.9594		0	6.3948	7.7698	5.9526	5.5048	7.3763	7.7458	5.8020	5.3642	7.9448	6.7368	5.5370	4.8450
hsa-miR-4290	6.8035	12.9617	13.1929	12.4108	0.0000	0.0000	0.0000	0.4395		0	14.6233	15.1218	13.0121	12.6652	15.1503	13.0245	11.9937	11.2796	13.4683	14.4752	12.2346	10.5167
hsa-miR-605	7.7473	11.6893	11.3445	12.5112	0.0000	0.0000	0.0000	0.1677		0	11.5862	15.6958	13.1476	12.3206	11.4053	9.9298	10.3330	10.1489	11.0681	14.6297	11.0895	13.8026
hsa-miR-449b*	3.9846	7.1217	7.0177	7.3696	0.0000	0.0000	0.0000	0.6226		0	6.7469	10.0370	7.0954	6.1131	7.6528	7.6776	6.8454	6.4950	8.6113	6.8798	5.9042	5.6427
hsa-miR-483-3p	8.4652	17.0531	17.2901	16.4882	0.0000	0.0000	0.0000	0.5779		0	16.6447	20.7799	16.9872	14.2687	18.7086	17.1415	16.3064	17.7841	17.7821	19.2925	16.9666	13.1744
hsa-miR-4261	7.4424	4.7109	4.5383	5.1223	0.0000	0.0000	0.0001	0.0059		1	4.7426	4.9618	4.2571	4.0967	4.6127	5.6925	4.4815	5.3574	4.3512	5.1205	4.5964	4.8164
hsa-miR-125a-3p	28.2912	42.6754	41.8637	44.6099	0.0000	0.0001	0.0001	0.3868		0	42.2885	52.2947	46.6093	45.5422	36.1709	34.3809	46.5103	45.9902	37.2431	48.7022	42.4973	49.4064
hsa-miR-590-5p	29.2138	21.8650	22.0875	21.3348	0.0001	0.0001	0.0001	0.6264		0	20.7233	16.3069	23.4717	26.0443	24.2842	21.3150	21.1937	24.6498	22.7235	18.2858	21.8624	22.3599
hsa-miR-885-5p	6.7643	12.0221	12.1786	11.6490	0.0001	0.0001	0.0007	0.6437		0	12.6360	16.5029	12.5108	9.9218	14.1512	12.9898	11.4967	10.9523	12.2338	13.7971	10.8150	8.5940
hsa-miR-3152	110.0838	18.7536	16.7798	23.4578	0.0002	0.0001	0.0002	0.0504		1	26.9305	23.1853	14.1959	11.9357	13.5243	33.3389	15.4884	15.1586	17.2527	18.2533	15.2731	40.6143
hsa-miR-96	6.5555	8.7347	8.3799	9.5803	0.0002	0.0022	0.0000	0.0751		0	8.3649	6.7293	7.3591	12.2951	10.1210	8.2562	8.0364	10.9907	8.8217	10.3056	8.1959	9.3118
hsa-miR-764	3.8824	6.2784	6.3293	6.1571	0.0002	0.0002	0.0006	0.6750		0	6.2175	7.7903	6.2694	6.5827	7.2093	5.9323	5.9592	6.4095	6.5725	6.1166	6.0444	4.9885
hsa-miR-1208	17.3418	10.2189	9.7934	11.2329	0.0003	0.0002	0.0008	0.0055		0	10.9729	10.5536	9.0663	8.6424	9.8889	12.5768	9.8719	11.0189	9.0469	11.5216	9.4875	10.6096
hsa-miR-3186-3p	9.6147	6.3665	6.0342	7.1587	0.0004	0.0002	0.0040	0.0044		2	6.7314	5.9434	4.9813	5.8753	5.3340	8.1998	6.3252	7.2394	5.9870	7.9870	6.0883	7.0701
hsa-miR-3622b-5p	11.6717	6.6901	6.6143	6.8708	0.0004	0.0003	0.0005	0.4281		0	6.9432	6.5615	6.5233	5.9635	6.3589	7.8636	6.5687	6.9671	5.7233	5.9845	6.6885	7.5754
hsa-miR-3147	20.7028	13.0682	11.8607	15.9462	0.0005	0.0001	0.0213	0.0007		3	13.6725	12.5080	9.8528	12.0714	10.8002	15.5957	13.5508	16.9876	10.2872	19.2242	11.8509	12.6600
hsa-miR-4299	100.7789	184.8717	181.6321	192.5926	0.0005	0.0008	0.0004	0.5279		0	183.1245	197.4695	259.6366	193.3556	165.1709	198.9043	140.3124	154.6616	172.3570	214.8220	188.2609	219.9572
hsa-miR-3646	9.6895	12.5567	12.1917	13.4266	0.0006	0.0020	0.0001	0.0682		0	13.9175	15.0887	10.9982	12.7773	11.8143	14.3948	11.7540	12.1961	13.1104	15.2152	11.4447	12.0162
hsa-miR-4259	11.9964	8.8882	8.5375	9.7240	0.0006	0.0003	0.0077	0.0044		0	8.5555	9.8451	8.3072	9.4167	8.4212	10.9491	8.6503	10.1542	7.9595	8.0342	8.7783	9.3892
hsa-miR-1227	5.9467	8.5008	8.7616	7.8793	0.0007	0.0003	0.0119	0.1051		1	9.9996	8.0377	9.0658	7.3495	8.2631	7.9471	7.5827	7.2687	9.3705	10.5667	8.7274	7.4953
hsa-miR-1224-3p	3.6143	5.3037	5.4223	5.0210	0.0007	0.0004	0.0034	0.0782		0	5.8685	5.6999	5.1413	4.5017	5.6029	5.3076	4.8473	5.1252	5.2984	4.9981	5.3129	4.2197
hsa-miR-3620	21.1411	26.2531	27.0059	24.4589	0.0008	0.0002	0.0338	0.0348		1	29.5006	26.2025	25.0061	23.1551	26.1697	24.0720	28.5537	24.5535	27.3211	27.2037	25.7632	21.4797
hsa-miR-432	11.8768	7.3567	7.3054	7.4788	0.0008	0.0007	0.0010	0.6129		0	7.1368	6.7235	7.8786	7.6072	7.0372	8.3004	7.7202	7.6601	7.2033	6.7624	7.1140	7.3430
hsa-miR-1260	20.0625	33.5912	33.3903	34.0701	0.0009	0.0011	0.0011	0.7942		0	33.7448	38.8287	30.3648	27.9087	34.5411	42.7605	34.3043	34.4640	38.4048	32.8881	31.0528	32.0890
hsa-miR-3173	7.1852	5.0484	4.8820	5.4449	0.0009	0.0005	0.0044	0.0114		2	5.1291	5.3780	4.5560	4.2574	4.7320	6.0064	4.4267	5.1667	4.5772	5.1997	5.0897	5.7964
hsa-miR-4297	4.5155	6.9596	7.0632	6.7127	0.0010	0.0007	0.0036	0.4712		1	7.6714	8.0152	7.0673	5.5706	8.1855	5.6170	7.1029	7.6204	5.8726	8.0162	6.7803	4.8971
hsa-miR-574-3p	10.4090	17.8251	17.7874	17.9151	0.0010	0.0011	0.0011	0.9135		0	19.2907	21.3185	16.7920	15.6079	19.3037	22.2654	16.0482	15.9419	21.7001	20.7319	16.5179	15.0892
hsa-miR-1260b	17.0723	24.1046	24.4827	23.2035	0.0011	0.0007	0.0045	0.3322		0	23.3530	23.0328	25.1566	22.4863	25.1239	19.6714	25.1708	20.7120	27.0817	25.9208	21.7583	27.7638
hsa-miR-548q	31.1374	40.7595	38.9594	45.0498	0.0012	0.0061	0.0004	0.0655		0	39.5311	42.9972	41.4911	53.8669	33.6279	33.5786	41.6097	42.2955	38.8156	53.8427	37.5515	53.7801
hsa-miR-3189	7.4384	4.9192	4.8474	5.0903	0.0013	0.0011	0.0021	0.1733		0	4.6050	4.7840	4.6413	4.4123	4.6857	5.4824	5.1227	5.1065	4.3700	5.0961	4.8692	4.9376
hsa-miR-124	5.7699	4.0602	3.9678	4.2804	0.0015	0.0010	0.0040	0.0319		1	4.3457	4.0900	3.5424	3.4948	3.9447	4.7641	4.0981	4.6803	3.7194	4.0101	3.9903	4.0141
hsa-miR-500a	9.3469	5.6770	5.6352	5.7767	0.0017	0.0015	0.0020	0.6142		0	5.7196	5.2661	5.3875	4.8083	5.5867	5.6564	5.7758	5.1951	4.8776	6.0433	5.4896	5.9005
hsa-miR-652	10.6927	15.6497	16.1761	14.3953	0.0017	0.0008	0.0203	0.1039		0	14.1771	14.0776	17.6671	18.0595	18.3848	13.0286	15.4613	15.2412	18.1610	13.4113	16.2742	14.4279
hsa-miR-1281	25.3284	38.3470	38.3912	38.2416	0.0018	0.0018	0.0054	0.9649		0	40.8115	39.0264	31.5240	33.8545	31.8335	40.4829	36.8452	38.0090	46.4111	47.0996	35.4157	35.7230
hsa-miR-498	31.5552	38.2139	36.3283	42.7080	0.0018	0.0197	0.0002	0.0131		0	42.9451	49.0708	33.8208	41.5618	32.5446	43.4350	40.6690	44.8667	34.1510	46.5899	34.7559	39.0407
hsa-miR-335*	5.7746	7.9533	7.9818	7.8855	0.0021	0.0021	0.0042	0.8439		0	8.1357	7.3969	7.3355	7.9103	8.0789	8.9312	7.5561	7.9271	9.3161	9.2868	7.6765	7.3704
hsa-miR-1274b	145.5792	211.2652	219.7184	191.1185	0.0024	0.0010	0.0264	0.0068		1	218.4232	201.4568	226.5885	173.0797	184.6017	207.1104	230.4230	180.5952	229.1249	201.5744	219.0534	199.4252
hsa-miR-365	13.2648	17.0432	16.3172	18.7735	0.0024	0.0106	0.0002	0.0229		0	17.3659	19.8664	14.5146	17.4680	14.8526	20.1737	15.6430	18.4971	18.1233	23.5144	17.1916	18.7214
hsa-miR-518e*	8.1839	5.6325	5.4044	6.1762	0.0027	0.0015	0.0152	0.0386		0	5.5720	5.2582	4.6529	5.5333	5.1596	6.3404	5.7941	7.1840	5.2467	6.9750	5.4370	5.5651
hsa-miR-4286	79.0922	118.3867	119.5393	115.6397	0.0028	0.0023	0.0090	0.6640		0	117.0635	97.9431	121.6068	106.0970	103.9576	120.0804	136.4309	128.1880	126.9907	108.6622	118.8777	151.4289
hsa-miR-152	7.1550	4.5293	4.6387	4.2685	0.0029	0.0039	0.0015	0.1760		0	4.3628	3.5232	5.4077	5.4724	5.0959	4.3321	4.5231	4.2772	4.5933	3.7251	4.5554	4.4198
hsa-miR-501-5p	7.8421	6.0170	5.9657	6.1392	0.0031	0.0026	0.0052	0.3726		0	5.6970	5.6896	5.6048	5.4628	5.6145	5.7596	6.5181	5.9614	5.9218	6.9555	5.8725	6.0732
hsa-miR-648	5.0082	3.4655	3.4545	3.4918	0.0032	0.0030	0.0037	0.8211		1	3.1530	3.6717	3.5955	3.2149	3.7092	2.9799	3.6207	3.4121	2.8683	3.1532	3.5335	3.8101
hsa-miR-3617	20.9409	10.3924	10.5604	9.9919	0.0032	0.0035	0.0025	0.2911		0	11.8307	9.8313	10.7793	9.0306	10.8794	11.5081	10.4304	10.1750	9.2959	7.7443	10.0684	9.9187
hsa-miR-3654	6.5949	4.5288	4.3860	4.8691	0.0038	0.0024	0.0123	0.0504		0	4.1036	4.7913	4.2662	4.2891	4.1010	4.5929	4.6229	4.6027	3.7504	4.9133	4.5447	4.9388
hsa-miR-1825	21.5448	31.4676	31.4678	31.4671	0.0038	0.0041	0.0097	0.9998		0	33.7061	33.6356	26.1739	27.8611	28.6000	33.7870	29.6942	30.6923	36.9470	38.2846	29.1505	29.6045
hsa-miR-615-3p	4.2348	5.5301	5.6350	5.2799	0.0039	0.0023	0.0177	0.1538		0	5.6125	5.6370	5.5390	5.0595	6.1516	4.8828	5.9425	5.1601	5.9363	5.5468	5.0504	4.9504
hsa-miR-187*	41.9878	56.5584	54.6651	61.0708	0.0042	0.0107	0.0012	0.1197		0	61.8078	70.7004	53.6311	71.3142	54.1380	64.7201	59.3395	55.8341	49.5115	50.7770	53.3610	58.1545
hsa-miR-193a-5p	10.7478	13.3514	12.4386	15.5268	0.0044	0.0507	0.0002	0.0044		1	13.6778	12.5620	9.5138	17.8801	12.8924	14.8905	13.3530	16.0868	11.8981	15.8610	12.2158	14.8722
hsa-miR-937	5.6927	8.1526	8.1895	8.0645	0.0047	0.0044	0.0082	0.8062		0	8.9880	8.6036	8.1881	6.8418	8.3613	8.1706	7.5476	8.2727	7.9860	10.2231	7.6131	6.6439
hsa-miR-877*	8.4031	11.7118	11.6880	11.7685	0.0051	0.0055	0.0091	0.9249		0	11.9923	10.1283	11.0874	11.3957	10.2224	12.9890	11.0682	11.3746	13.8826	14.3795	11.4864	11.7990
hsa-miR-29c*	6.2569	7.6335	7.6571	7.5773	0.0053	0.0050	0.0133	0.8253		0	7.6128	7.7501	7.2674	7.7702	7.9517	7.4293	8.1733	7.2266	8.0694	8.8111	7.5847	7.1792
hsa-miR-3200-5p	6.4448	3.9137	3.9079	3.9276	0.0053	0.0052	0.0057	0.9369		0	4.2970	3.8909	4.0190	3.4470	3.8861	3.3974	3.9393	4.2411	3.2402	3.6644	4.0582	4.2566

hsa-miR-200b*	5.5035	4.1435	4.2204	3.9602	0.0055	0.0079	0.0025	0.1549		0	3.7163	4.0469	4.5031	3.8272	4.3162	3.2897	4.5486	3.9294	3.8680	4.1721	4.0874	3.9946
hsa-miR-24	546.2312	378.9329	382.2812	370.9527	0.0065	0.0075	0.0056	0.6782		0	379.6735	367.8365	435.6951	410.1724	376.0131	383.6331	353.2362	338.8934	423.3431	360.9086	409.9178	367.7436
hsa-miR-4281	4678.2430	########	6454.8529	6562.4632	0.0066	0.0075	0.0247	0.8737		0	6550.3117	7542.9330	6215.6558	7771.0343	6726.6366	6004.9700	6373.5197	7232.4150	6988.3992	5453.4912	6495.0423	5031.9288
hsa-miR-3679-3p	8.3772	10.4565	10.2791	10.8795	0.0073	0.0129	0.0046	0.3119		0	10.8216	10.3370	9.6238	10.8865	9.4430	10.9665	10.4685	10.4902	11.3244	13.2826	10.0119	10.3411
hsa-miR-27a	434.8478	259.6334	263.7009	249.9391	0.0082	0.0095	0.0061	0.5015		0	272.5534	235.2986	274.8507	234.3378	276.9976	263.2407	218.1148	232.2526	286.5067	222.6955	275.4927	271.9454
hsa-miR-634	6.0679	8.8993	9.0916	8.4409	0.0090	0.0061	0.0394	0.3629		1	9.5997	10.4934	9.1134	8.1372	7.7821	7.2875	9.7861	8.9817	10.3158	10.6065	8.1401	8.3685
hsa-miR-3124	12.7331	9.7431	9.1302	11.2040	0.0093	0.0028	0.2046	0.0074		1	11.0265	10.5190	7.2160	6.5792	8.2577	12.7377	9.5777	13.6508	8.8240	11.6927	9.3940	10.8082
hsa-miR-212	6.6309	7.9323	8.0867	7.5642	0.0094	0.0049	0.0606	0.0681		1	7.9759	8.3747	9.5520	7.8586	7.9119	6.7327	7.8212	7.4926	7.6057	6.8455	8.3193	8.0326
hsa-miR-1	6.1395	3.0765	3.1834	2.8216	0.0104	0.0127	0.0067	0.1153		1	2.6256	2.5426	3.7459	3.7800	3.2026	3.1327	3.1328	2.8084	3.2366	2.8092	3.3511	2.6331
hsa-miR-513a-5p	20.7460	14.4001	14.2072	14.8599	0.0110	0.0094	0.0182	0.4704		0	16.4744	16.2239	14.4962	13.1895	13.2677	16.7255	12.3928	13.9544	13.3128	14.9370	13.2022	16.8660
hsa-miR-1249	42.8708	57.6686	53.1340	68.4763	0.0112	0.0620	0.0005	0.0034		0	55.1816	74.0371	55.8403	78.5394	49.1933	65.1234	57.2946	65.1496	47.9981	74.4511	53.5127	59.8288
hsa-miR-3659	6.8454	5.4005	5.3808	5.4477	0.0112	0.0105	0.0159	0.7794		0	5.5343	5.5716	5.2264	5.7803	5.2906	4.4592	5.3428	5.3234	5.0411	5.5671	5.5353	5.3476
hsa-miR-199a-3p	187.4291	103.7595	108.3400	92.8425	0.0117	0.0162	0.0059	0.1522		0	102.6425	96.2700	139.3255	138.9798	125.4703	91.9759	85.9032	81.9945	132.3922	76.1535	96.5039	92.1029
hsa-miR-449c*	7.1047	11.1070	11.5738	9.9945	0.0130	0.0071	0.0643	0.0290		2	13.7283	10.3763	10.9696	7.5735	11.6865	10.0439	11.7092	11.5818	9.3049	11.1053	12.0148	7.9202
hsa-miR-1288	11.7260	8.0096	7.9611	8.1251	0.0131	0.0123	0.0163	0.6833		3	8.1631	8.0407	8.0503	6.3687	7.8182	6.9737	8.7583	6.7957	7.4224	9.2140	6.8882	8.2519
hsa-miR-493*	5.8422	4.5045	4.4605	4.6093	0.0132	0.0112	0.0215	0.3589		0	4.2929	4.3766	4.7898	4.8146	4.3370	4.6381	4.6651	4.3980	4.2960	4.4668	4.2871	4.8265
hsa-miR-376a	29.4060	13.5951	14.0706	12.4618	0.0133	0.0157	0.0092	0.2246		0	12.1996	9.2152	14.5177	12.2885	13.8905	14.0809	14.7347	13.5712	15.0466	9.7760	17.4153	13.4940
hsa-miR-3680*	22.9451	17.2742	16.6590	18.7406	0.0136	0.0076	0.0650	0.0433		1	19.7546	18.9082	13.3250	11.7823	15.7550	21.6983	16.6950	18.3231	16.4545	22.1728	15.1076	17.4500
hsa-miR-339-3p	13.9045	8.9176	9.0472	8.6086	0.0143	0.0165	0.0109	0.4373		0	10.0747	9.2407	10.5995	7.8527	9.7753	8.0420	9.0332	9.5340	6.8203	8.6135	8.9848	7.6108
hsa-miR-22*	7.1165	5.9967	5.9718	6.0560	0.0148	0.0136	0.0262	0.7464		0	5.9626	5.7686	6.2950	6.2300	5.8811	6.7187	5.8717	6.0684	6.1591	5.2736	5.8886	6.6006
hsa-miR-526b	10.4098	8.9593	8.3050	10.5189	0.0151	0.0011	0.8821	0.0004		1	8.3843	10.5640	7.9838	10.1854	7.5083	10.8658	8.1990	11.4077	7.6525	11.7410	9.1702	8.5335
hsa-miR-130b	52.2241	42.1581	45.1908	34.9302	0.0166	0.0830	0.0004	0.0001		1	39.3879	28.1976	48.3848	38.1655	47.8981	34.2142	47.7236	38.3785	44.1535	33.4762	48.1456	35.4773
hsa-miR-10b*	6.8931	5.8332	5.5120	6.5987	0.0168	0.0031	0.5759	0.0098		0	5.1450	5.8566	5.2972	6.9075	4.8829	5.8106	5.6816	5.9861	5.2478	7.3695	5.4753	6.6989
hsa-miR-551b*	4.2052	2.9556	2.8654	3.1704	0.0175	0.0122	0.0443	0.0524		1	2.4267	3.4274	2.9812	3.1710	3.1086	2.5984	2.8692	3.3304	2.4504	3.0923	3.0309	2.8823
hsa-miR-296-5p	16.4379	21.4511	20.2085	24.4125	0.0177	0.0636	0.0019	0.0179		0	19.6529	26.8896	22.0199	31.6017	18.8347	21.9587	21.9423	23.9100	18.7166	24.3823	19.4199	20.9254
hsa-miR-370	7.8752	5.9839	5.9212	6.1332	0.0186	0.0161	0.0304	0.4861		0	4.8825	5.2474	6.1948	5.9565	5.6717	5.4689	6.1737	6.2461	5.1337	6.3064	6.0497	5.6548
hsa-miR-3125	19.6883	13.3897	13.1322	14.0033	0.0187	0.0154	0.0323	0.2770		1	12.2145	13.6322	13.0543	11.3034	11.9683	12.5186	15.2657	13.8236	11.3745	16.3582	11.9480	12.9288
hsa-miR-144*	10.1405	14.3164	13.7248	15.7265	0.0187	0.0507	0.0095	0.2838		0	8.8352	9.0482	11.6998	26.0703	18.2836	13.6616	14.8151	20.5480	16.2434	11.3878	13.2658	15.3212
hsa-miR-4274	13.1706	16.1850	15.5374	17.7283	0.0190	0.0586	0.0040	0.0605		0	15.2069	16.8166	14.8243	21.6614	15.5764	17.1092	15.8279	18.0947	15.6722	21.1016	15.1623	14.7972
hsa-miR-378b	7.3937	5.5980	5.4721	5.8980	0.0199	0.0142	0.0515	0.1549		0	5.1388	5.7588	5.6402	5.3002	5.2841	5.4473	5.1723	6.0513	4.9140	6.0364	5.7886	5.7274
hsa-miR-664	9.0753	11.5268	11.6679	11.1906	0.0199	0.0158	0.0612	0.5190		0	11.0980	10.8183	10.8991	10.3518	11.6957	12.0160	10.9018	10.5245	13.4477	13.7334	10.6019	11.1778
hsa-miR-184	8.0212	6.5066	6.3081	6.9796	0.0202	0.0104	0.1290	0.0946		0	6.1885	5.4045	5.8515	6.0538	5.7042	8.0088	6.6790	7.0064	5.4547	7.9219	6.3623	6.5835
hsa-miR-3154	8.8566	7.4199	7.1614	8.0360	0.0213	0.0088	0.2035	0.0275		0	7.0927	9.8174	6.9557	8.0444	6.4044	7.7613	7.0109	8.0126	6.3463	6.8696	7.8208	7.8813
hsa-miR-1236	4.2550	5.2462	5.2093	5.3341	0.0220	0.0273	0.0236	0.6711		0	5.8624	5.4225	5.1529	5.5128	5.6366	4.9344	4.9968	5.9203	5.2609	6.2170	5.0182	4.4863
hsa-miR-3132	7.6222	5.4971	5.4459	5.6191	0.0227	0.0205	0.0335	0.6179		0	5.1844	5.6854	5.9618	6.2228	5.7931	5.2192	5.2911	5.7830	4.4132	5.2502	5.5822	5.3553
hsa-miR-194	5.2193	6.5295	6.5691	6.4349	0.0231	0.0257	0.0542	0.7948		1	4.8052	4.1846	6.1023	8.7307	9.0978	5.7059	6.5910	8.2204	6.6981	5.7912	6.2653	5.8121
hsa-miR-376c	40.5171	19.9883	21.1232	17.2836	0.0240	0.0313	0.0131	0.0521		0	18.4661	12.1243	22.1663	19.1490	20.2093	17.3917	21.6910	18.6884	24.0035	13.5858	25.2250	19.6889
hsa-miR-1254	12.6992	6.4584	6.1456	7.2038	0.0250	0.0198	0.0444	0.0335		0	6.7043	9.6504	6.7347	7.7780	6.5042	5.8230	6.1039	7.7042	4.7522	6.3964	6.8722	5.5660
hsa-miR-148a	54.6770	38.1679	37.1522	40.5887	0.0257	0.0198	0.0571	0.2818		0	33.5631	41.6763	47.4788	53.8018	37.4609	40.0883	30.3852	41.7448	34.3171	31.9697	40.8113	41.0874
hsa-miR-148b	29.6630	21.9475	22.5440	20.5258	0.0259	0.0389	0.0122	0.2113		0	20.7264	17.7760	24.3875	25.2035	24.7108	20.0906	21.2700	22.9011	25.8938	19.2001	22.4559	21.1570
hsa-miR-622	26.8028	17.6721	16.6926	20.0066	0.0276	0.0169	0.0957	0.0228		0	17.8679	23.9835	15.7670	19.9452	14.8959	15.9576	19.1958	18.4474	14.6051	20.0606	17.3405	22.2797
hsa-miR-378	8.4920	6.5946	6.3306	7.2238	0.0277	0.0147	0.1401	0.0270		0	5.8272	7.4493	6.0330	7.3657	6.8409	6.8682	6.4425	7.5413	5.0388	7.4049	6.7424	6.9842
hsa-miR-410	14.8572	9.2057	9.5996	8.2667	0.0290	0.0400	0.0141	0.0506		1	8.3691	6.8898	10.1577	8.7604	9.8117	9.7620	9.9625	8.4069	10.1303	6.3359	11.1151	8.5658
hsa-miR-197	56.9282	66.4866	70.4971	56.9281	0.0295	0.0042	1.0000	0.0000		3	69.1621	63.1997	71.7448	52.9174	77.0749	52.0956	67.0736	59.1294	71.4987	54.1300	69.9230	59.2106
hsa-miR-486-5p	443.9560	734.3557	746.6522	705.0493	0.0299	0.0257	0.0588	0.5697		0	710.7628	451.9656	561.9675	594.9204	770.4781	592.9885	914.7949	885.9963	801.0025	1057.2347	823.2444	674.4085
hsa-miR-3648	295.4201	137.6523	129.8966	156.1369	0.0300	0.0241	0.0527	0.1102		0	141.9059	198.7355	139.6602	168.6520	123.8859	99.6347	148.4752	132.7038	111.4367	151.6658	131.7786	167.8132
hsa-miR-495	10.0791	6.9128	6.8448	7.0750	0.0302	0.0277	0.0403	0.5813		0	6.7763	6.3696	7.7147	6.8525	6.9759	8.0864	6.5343	7.0861	7.5947	7.0533	6.8170	7.4579
hsa-miR-3605-5p	7.0944	5.6820	5.6741	5.7009	0.0304	0.0309	0.0351	0.9216		0	5.5360	5.1332	5.5838	5.7188	5.6338	5.5400	5.4264	6.3296	4.7163	5.5117	5.8011	5.6774
hsa-miR-337-5p	6.5580	4.7170	4.7155	4.7207	0.0305	0.0306	0.0334	0.9850		0	4.1915	4.8861	4.9119	5.0516	4.7194	4.8353	4.8400	4.7453	4.5889	3.6916	4.9442	4.7523
hsa-miR-129*	7.5632	9.3936	9.4362	9.2923	0.0307	0.0284	0.0628	0.8051		0	10.5060	9.0175	8.9428	8.2522	8.3489	9.5653	9.0632	9.5353	11.4146	11.3624	8.9744	9.4849
hsa-miR-609	5.4979	7.2539	7.5874	6.4590	0.0309	0.0135	0.2200	0.0024		2	8.4435	6.2816	7.0105	4.7941	7.1097	6.3668	7.7297	6.8343	6.5749	7.8828	7.5868	5.9989
hsa-miR-892b	8.3172	6.5362	6.6351	6.3003	0.0309	0.0398	0.0182	0.1775		2	6.6868	5.7050	6.6984	5.6083	6.7671	5.5898	7.3323	5.7386	6.4386	6.7882	5.6894	6.9250
hsa-miR-30c	13.7511	17.6915	18.3119	16.2129	0.0310	0.0161	0.1884	0.0769		2	15.6458	14.3956	20.1576	23.6551	22.2836	15.0390	17.4550	17.0338	20.6749	13.5190	18.6142	17.1069
hsa-miR-766	22.1050	27.4305	28.6911	24.4260	0.0312	0.0109	0.3393	0.0012		2	27.9460	25.7046	27.3973	25.0398	28.8661	26.3524	28.1136	23.5066	33.1978	27.4705	28.2911	22.6109
hsa-miR-423-5p	46.3556	56.3652	55.9704	57.3062	0.0313	0.0378	0.0493	0.7397		1	49.1901	44.3774	53.5322	64.3119	59.2475	54.6120	66.3918	73.2279	53.2213	63.0379	57.0443	54.0867
hsa-miR-760	31.8798	18.0874	17.6208	19.1996	0.0315	0.0274	0.0463	0.3099		1	19.8908	16.5484	12.4734	15.3027	16.8294	18.2871	23.3396	18.4649	13.4596	21.8884	15.2830	17.9954
hsa-miR-539	4.8215	5.5194	5.6578	5.1896	0.0321	0.0134	0.2482	0.0026		2	5.3044	4.5761	5.3350	5.7036	5.2345	5.1668	6.0781	5.1773	5.6874	5.3333	5.7480	5.3170
hsa-miR-27b	61.7455	43.9612	45.9162	39.3019	0.0326	0.0551	0.0109	0.0702		0	43.1059	33.9304	54.1092	51.8297	47.2817	39.5639	40.7306	36.6235	52.0107	35.3370	45.1917	42.6356
hsa-miR-330-3p	4.6488	3.5325	3.6862	3.1663	0.0331	0.0609	0.0082	0.0053		1	3.1028	2.9901	4.1090	3.8087	4.0372	2.9358	3.5351	3.1182	3.5211	2.7388	3.7693	3.1675
hsa-miR-204	5.4646	7.3361	7.3040	7.4128	0.0334	0.0380	0.0409	0.8506		0	7.5172	8.4599	7.6009	7.6572	7.9109	7.9087	7.5061	7.2444	7.7795	8.0399	6.5948	6.1670
hsa-miR-30d	125.7467	148.2987	153.8774	135.0028	0.0335	0.0110	0.4417	0.0363		1	146.1589	106.4385	131.9416	119.4275	155.0372	122.3306	179.2243	160.0387	155.4028	150.9921	159.9728	131.3221
hsa-miR-3187	11.3404	8.7004	8.6096	8.9169	0.0347	0.0308	0.0596	0.6212		0	8.3518	8.6746	8.8582	10.8777	8.4515	7.4639	8.6875	9.0756	7.4903	9.2074	9.2778	7.6030
hsa-miR-575	273.2678	363.2080	354.3391	384.3456	0.0356	0.0571	0.0184	0.2800		0	348.4054	358.0843	423.6219	395.2436	319.2011	385.1022	381.2214	312.4379	310.9224	411.9098	329.1281	444.2145
hsa-miR-4327	455.2199	591.4293	570.6099	641.0489	0.0366	0.0726	0.0104	0.0837		0	643.3059	781.8555	549.9483	583.6092	590.6958	667.9250	610.3204	700.3398	460.6832	560.5392	625.7601	611.1034

hsa-miR-1305	29.9238	21.0455	20.9978	21.1593	0.0368	0.0362	0.0417	0.9016		1	21.6580	18.5359	21.0627	20.0419	18.2040	18.8086	23.2607	18.1782	20.2629	24.2947	18.0267	21.7998
hsa-miR-494	53.1544	75.3861	68.1592	92.6101	0.0370	0.1371	0.0087	0.0467		0	56.2341	57.8888	65.8699	113.6909	60.8340	89.0319	79.7295	114.1539	58.5460	112.6181	64.0850	79.0523
hsa-miR-210	10.9621	12.4967	12.8013	11.7708	0.0371	0.0179	0.3147	0.1103		0	10.3242	8.3748	13.5358	14.0088	13.0990	10.4733	14.7183	14.0019	12.9176	13.9398	13.0087	10.5522
hsa-miR-627	5.8773	4.9032	4.9053	4.8983	0.0371	0.0384	0.0423	0.9739		0	4.2638	4.2463	4.7444	5.0974	5.4983	4.8033	5.1555	5.8715	4.1147	4.1060	5.1749	5.1034
hsa-miR-409-3p	19.7238	13.0183	13.2109	12.5594	0.0388	0.0440	0.0309	0.4772		0	13.0180	10.3977	12.5730	13.0850	12.8489	13.4806	14.0334	13.4436	14.4984	12.2081	14.2912	11.6194
hsa-miR-382	7.9280	6.0909	6.2706	5.6624	0.0390	0.0594	0.0158	0.0636		0	5.2946	4.6556	6.3912	6.0284	6.3205	5.5459	6.9738	6.0585	6.0847	4.9925	6.9158	6.0304
hsa-miR-1295	21.0597	8.8994	9.1702	8.2541	0.0395	0.0435	0.0318	0.2300		0	11.3606	6.7485	7.5458	5.3936	8.8248	10.0783	9.5748	6.7707	8.0398	9.0031	9.0181	8.7064
hsa-miR-199b-5p	5.1342	5.8767	5.7506	6.1772	0.0396	0.0805	0.0090	0.0275		0	5.4748	5.4863	5.7835	7.1166	5.9552	5.8885	5.5550	6.3236	5.5466	6.1614	5.6942	6.8869
hsa-miR-516b	4.5633	3.7189	3.6401	3.9066	0.0396	0.0271	0.1054	0.0614		0	3.4247	4.1271	3.8406	4.0814	3.5482	3.7090	3.7397	3.9954	3.2389	4.0074	3.6990	3.8578
hsa-miR-513b	3.3275	3.9455	4.0654	3.6597	0.0400	0.0180	0.2585	0.0029		2	3.7530	3.3724	4.3930	3.9350	3.8335	3.4567	4.0652	3.8409	3.5725	3.7682	4.1000	4.0029
hsa-miR-1973	6.9519	4.8916	5.1252	4.3349	0.0402	0.0653	0.0135	0.0169		0	4.9737	3.7859	5.6236	5.3613	4.7613	4.2371	5.5939	4.5643	4.6288	3.4877	5.8873	4.6446
hsa-miR-128	15.6173	12.2003	12.1255	12.3785	0.0410	0.0388	0.0637	0.7824		0	10.1635	10.4071	15.5826	18.5904	14.4722	11.8579	10.8097	14.2841	11.6290	9.9085	12.5101	12.0895
hsa-miR-155	6.3251	7.1037	7.1252	7.0524	0.0421	0.0396	0.0929	0.8103		1	6.3974	6.4906	6.9642	8.4579	7.1505	7.0544	7.4803	7.1377	7.2858	6.0693	7.2515	7.9315
hsa-miR-3177	5.7920	4.8310	4.7755	4.9634	0.0426	0.0344	0.0801	0.2896		1	4.6546	4.9297	4.8239	5.0865	5.2348	4.7392	4.5124	5.4687	4.2330	4.4765	5.0353	4.5990
hsa-miR-361-3p	6.2144	6.8115	6.8782	6.6525	0.0438	0.0288	0.1598	0.2345		0	6.0718	6.1431	7.0293	7.7264	7.4776	6.6647	7.2016	6.9230	6.6354	6.1471	6.8930	6.6986
hsa-miR-4294	12.3188	8.6225	8.8967	7.9688	0.0442	0.0597	0.0220	0.0100		0	9.6326	8.6760	9.0425	8.0239	8.9331	8.3037	8.7526	7.8750	8.3357	7.2150	8.4652	7.7957
hsa-miR-1274a	10.3258	13.1037	13.6250	11.8614	0.0445	0.0212	0.2572	0.0056		1	12.8183	10.4455	15.9186	13.7090	11.7754	12.1844	14.6038	12.7824	13.4037	11.1162	13.5398	12.8556
hsa-miR-193b*	11.1784	8.6719	8.2728	9.6230	0.0457	0.0241	0.2173	0.0186		0	8.9757	8.0923	8.3481	9.1924	7.3111	9.0810	8.3237	9.7497	7.2390	11.3478	8.2423	8.8817
hsa-miR-1181	30.9568	36.4090	37.0033	34.9927	0.0467	0.0313	0.1688	0.2786		0	42.3919	35.2602	37.6839	33.3059	33.8058	33.5137	39.5531	34.2192	34.8596	42.2828	34.1541	32.8099
hsa-miR-139-5p	6.0400	7.2607	7.5530	6.5641	0.0478	0.0191	0.4114	0.0185		0	6.5464	4.9182	9.0396	8.4868	8.1690	6.3951	7.6983	7.1912	7.7191	5.6364	7.2008	6.7517
hsa-miR-30e	72.3166	84.5620	85.8757	81.4310	0.0531	0.0365	0.1673	0.2423		1	73.4473	56.1663	84.5573	83.7322	92.0416	78.2995	86.5189	98.0306	85.6003	83.5517	95.4096	82.9857
hsa-miR-1273e	27.3295	21.6959	20.4810	24.5913	0.0531	0.0230	0.3568	0.0083		1	21.3600	26.1533	22.2986	20.8549	17.7567	22.0338	20.5069	25.1470	17.5029	27.1207	21.3870	21.5209
hsa-miR-377	18.9925	12.0053	12.4432	10.9616	0.0537	0.0686	0.0318	0.1471		0	11.5110	8.6927	12.9778	11.2741	12.6910	13.4561	13.0786	11.6929	14.1003	9.4567	13.6790	10.6353
hsa-miR-3156	80.8210	59.0930	55.0163	68.8091	0.0576	0.0282	0.2973	0.0066		0	54.4779	69.4202	53.4520	64.7276	47.8274	65.6434	63.9789	72.6878	50.1041	71.4440	51.9980	64.5903
hsa-miR-487b	12.8554	8.0410	7.6857	8.8878	0.0600	0.0459	0.1165	0.0469		0	6.7380	8.9188	8.7695	9.9146	8.1976	9.3107	7.6492	8.9872	7.6207	7.4874	8.1060	9.0463
hsa-miR-328	17.9753	23.5525	25.3708	19.2189	0.0608	0.0184	0.6791	0.0010		3	27.1660	24.7279	25.5009	17.5092	31.1534	16.4025	24.4704	17.7684	24.4358	20.2587	23.3586	15.9510
hsa-miR-222	9.0022	10.7366	10.9721	10.1754	0.0612	0.0385	0.2280	0.1724		0	9.4188	7.2011	10.5233	9.7352	11.7867	9.8194	11.0879	12.3329	10.9205	9.4784	12.1227	10.8970
hsa-miR-485-3p	11.2614	13.7914	14.4291	12.2717	0.0613	0.0248	0.4568	0.0054		0	15.2403	12.3445	14.4835	10.5663	15.9021	13.8200	14.8742	12.1758	13.8719	12.9903	13.3402	9.9944
hsa-miR-3926	9.3816	7.6857	7.4090	8.3453	0.0614	0.0339	0.2649	0.0499		1	7.5604	9.7805	8.0141	8.4740	7.0810	8.2477	6.0796	8.6646	6.5309	7.6294	8.7006	7.7141
hsa-miR-18b	9.1460	10.6153	10.9013	9.9337	0.0632	0.0370	0.3658	0.1840		0	8.8504	6.9416	12.1355	13.9742	13.0678	9.4225	10.9119	11.2904	11.3622	8.9940	10.9202	10.1524
hsa-miR-10b	6.5418	7.3190	7.0591	7.9387	0.0672	0.2175	0.0067	0.0178		1	7.6068	6.9350	6.5506	6.7543	6.7719	9.7886	7.2822	7.9237	7.6161	9.3127	6.3942	7.9370
hsa-miR-3622a-5p	3.7853	2.9762	2.9353	3.0739	0.0673	0.0569	0.1130	0.4208		0	2.7779	3.3638	3.3234	3.3210	3.1452	2.7632	2.7858	3.1881	2.2304	2.6388	3.0113	2.9683
hsa-miR-4312	11.6779	14.6032	14.6662	14.4531	0.0674	0.0654	0.1102	0.8425		0	15.3164	13.1525	12.9016	13.3519	12.0829	14.5337	14.8098	13.6037	17.2150	19.0876	14.2709	14.5984
hsa-miR-16-2*	5.9761	6.7724	6.6492	7.0659	0.0686	0.1214	0.0236	0.1162		0	6.3393	7.2187	6.4150	8.0752	7.3718	7.2036	6.6080	7.2980	6.6402	7.0288	6.3525	6.6738
hsa-miR-431	5.8190	4.4451	4.6053	4.0632	0.0703	0.1061	0.0275	0.0243		0	3.9230	3.7129	5.3191	4.6859	5.0490	4.3180	4.5878	3.9980	4.7023	3.5513	4.5924	3.9384
hsa-miR-497	5.7465	4.9369	4.9274	4.9596	0.0710	0.0697	0.0871	0.8647		0	5.0271	4.0599	4.9860	4.6739	4.8155	5.3092	4.9785	5.2751	5.0134	5.1860	4.7231	4.9117
hsa-miR-149	15.9020	20.1705	19.7409	21.1945	0.0719	0.1061	0.0546	0.4441		0	22.4830	23.2078	16.9026	18.6223	18.1349	22.6553	18.0282	19.7730	24.5983	26.6390	17.8761	20.7425
hsa-miR-3651	5.9194	4.4903	4.2241	5.1248	0.0720	0.0376	0.3312	0.0300		0	3.4795	6.1507	5.3619	6.6059	4.2513	3.8065	3.6915	5.1686	3.4493	4.2872	4.8749	4.8784
hsa-miR-423-3p	5.5269	5.0827	5.1484	4.9261	0.0726	0.1253	0.0264	0.1377		0	5.0568	4.4966	5.1864	5.3586	5.1694	4.7438	5.2384	5.1877	5.0477	4.7859	5.2467	4.9100
hsa-miR-3610	45.1761	63.5513	63.0555	64.7332	0.0727	0.0797	0.1047	0.8369		1	75.9334	67.6795	59.4899	71.6985	62.7321	85.9324	67.0544	56.9266	55.7273	49.1576	57.9484	40.4459
hsa-miR-3679-5p	605.8853	671.0967	650.8002	719.4698	0.0748	0.2274	0.0146	0.0804		0	604.8706	727.8310	707.4742	615.5064	601.4125	712.4585	630.6109	682.5528	567.1492	802.9666	654.0176	705.5603
hsa-miR-337-3p	6.3131	7.7985	7.7274	7.9680	0.0772	0.0955	0.0669	0.6428		0	8.3269	8.7856	7.8191	8.1769	8.8222	8.5397	7.9294	7.3260	7.9951	9.2522	6.8083	7.3895
hsa-miR-720	500.5809	710.1715	757.4520	597.4863	0.0774	0.0366	0.4094	0.0038		3	741.6709	545.6489	703.6964	415.9699	618.3026	834.7454	810.8155	588.6042	883.3732	640.8153	764.8893	662.7931
hsa-miR-379	5.1970	4.0241	4.1099	3.8195	0.0848	0.1084	0.0505	0.1732		0	3.4416	3.3148	4.4172	4.0612	4.1554	3.7504	4.5520	4.1703	4.0612	3.2659	4.3602	3.7734
hsa-miR-550a	22.7000	16.3360	16.1806	16.7064	0.0875	0.0814	0.1160	0.7005		0	17.3934	14.4017	14.1431	16.1899	13.2046	17.9587	14.7231	16.1763	20.1377	19.4816	16.6974	18.5927
hsa-miR-542-5p	5.6482	4.9662	4.9545	4.9942	0.0887	0.0848	0.1272	0.8575		0	4.5810	5.1857	5.3562	5.0782	5.1690	4.8521	4.7460	5.0975	4.2808	4.3802	5.2013	5.2524
hsa-miR-671-3p	7.5680	5.9544	5.8603	6.1786	0.0892	0.0743	0.1512	0.3632		1	6.3554	5.1214	4.9250	5.8116	5.2597	5.9593	5.2843	5.9461	6.7801	7.5303	6.2545	6.8816
hsa-miR-1287	23.7551	18.8967	18.5661	19.6844	0.0903	0.0734	0.1748	0.4360		0	21.7793	18.3047	13.3367	11.3300	16.0406	25.2619	21.3621	19.6393	19.2498	21.1664	16.3531	19.6456
hsa-miR-363	21.8629	29.6882	30.0273	28.8800	0.0916	0.0862	0.1440	0.6796		0	24.4221	19.9607	27.9804	41.3809	34.6822	26.1336	29.5937	32.9009	32.6647	27.2347	32.0973	29.7285
hsa-miR-23a	476.2388	343.5368	351.0243	325.6918	0.0917	0.1112	0.0636	0.3567		0	357.0505	352.6903	398.9825	383.8643	383.8642	335.6717	282.1394	307.8946	379.7949	274.6584	348.3745	331.4250
hsa-miR-1280	37.6557	47.8751	47.7854	48.0888	0.0929	0.0978	0.1162	0.9370		0	48.9948	39.5460	41.6701	43.0303	38.6395	50.1497	49.1799	46.0505	57.7734	60.1278	43.8092	55.2802
hsa-let-7b*	18.7914	23.6015	23.3889	24.1083	0.1018	0.1229	0.1159	0.7630		0	25.3576	26.8239	18.5555	21.4034	22.6790	26.4455	21.9850	21.7090	29.3640	29.9673	21.5733	23.7519
hsa-miR-7	5.5466	6.3307	6.2984	6.4075	0.1020	0.1237	0.0998	0.7457		0	5.3901	5.2703	6.2622	8.4123	7.1562	6.1628	6.8651	7.1155	5.9573	5.8651	6.2653	6.8487
hsa-miR-583	6.7158	5.7064	5.7093	5.6996	0.1021	0.1040	0.1115	0.9685		0	5.8841	5.8176	5.7682	4.9462	5.1825	5.2825	6.1069	4.9639	5.3212	6.6090	5.4304	5.9631
hsa-miR-543	6.0142	5.2659	5.2051	5.4108	0.1050	0.0834	0.1977	0.2595		0	5.0669	5.5526	5.4595	5.2340	5.3801	5.7464	5.2001	5.1026	5.6791	5.5854	4.9238	5.4560
hsa-miR-371-5p	32.5423	37.6119	35.9945	41.4668	0.1074	0.2635	0.0343	0.1071		0	32.3941	35.6125	31.8126	40.7980	35.1964	42.5794	43.8503	43.3695	30.0859	44.3511	35.2731	38.2999
hsa-miR-623	35.7836	43.6425	44.5760	41.4177	0.1091	0.0839	0.2858	0.3876		0	39.5815	38.9674	48.2448	35.7444	39.3367	33.8358	47.1736	34.9497	43.3961	51.8319	37.7744	49.3267
hsa-miR-3911	45.8262	54.2887	50.8777	62.4182	0.1097	0.3270	0.0159	0.0283		0	48.4186	65.0224	56.1228	70.2575	49.4652	58.3626	53.0271	58.3902	43.8309	70.4543	53.4698	50.7593
hsa-miR-1303	6.0071	6.8809	6.7246	7.2532	0.1104	0.1870	0.0385	0.0957		0	5.7981	7.5644	6.8697	8.9742	7.4213	6.1265	7.0992	8.0916	6.1422	6.6387	7.0183	6.5704
hsa-miR-670	5.2613	6.3519	6.3608	6.3307	0.1114	0.1111	0.1377	0.9312		0	5.5744	5.6932	5.5892	7.5648	6.0830	6.9564	6.9489	6.1703	6.3526	5.9094	6.6406	5.8547
hsa-miR-3176	6.7569	6.1129	5.8321	6.7822	0.1142	0.0306	0.9529	0.0001		2	6.0182	6.4283	5.4997	5.8138	5.9549	6.6437	5.7499	7.3725	5.1598	7.2806	6.1142	5.8014
hsa-miR-129-3p	8.4382	10.0110	10.0701	9.8702	0.1161	0.1062	0.1985	0.7706		0	11.2699	10.2747	9.5326	8.9003	8.5742	10.3771	9.6684	10.1449	11.8440	12.2657	9.2328	9.4898
hsa-miR-130a	303.2332	220.2510	230.1320	196.7013	0.1164	0.1637	0.0540	0.0608		0	239.3010	179.4911	254.6275	208.0267	230.7736	215.0290	220.8906	189.6020	258.1896	189.3451	237.5819	216.0039
hsa-miR-3181	7.1991	5.8871	6.2202	5.0932	0.1167	0.2354	0.0197	0.0003		3	6.2519	5.0737	6.5019	6.2576	6.2443	4.2021	6.0805	5.5752	5.3749	4.4585	6.1619	4.3483

hsa-miR-150	73.1520	92.4982	96.7732	82.3094	0.1172	0.0670	0.4626	0.0562		1	125.2998	69.4582	67.7447	90.1217	89.7507	97.5001	94.3746	76.6029	110.8246	71.7418	87.3077	100.2959
hsa-let-7f-1*	15.8544	19.4748	19.4467	19.5419	0.1172	0.1249	0.1579	0.9565		0	22.3767	18.8547	17.9074	16.5783	15.3053	22.3849	17.8340	17.3900	24.2106	24.6105	19.2024	20.3376
hsa-miR-300	4.5409	5.4847	5.6908	4.9934	0.1184	0.0649	0.4468	0.0047		3	6.3206	4.7603	5.5040	4.0481	5.6138	5.2483	5.3638	4.8464	5.0988	5.4935	5.8874	4.4267
hsa-miR-625	9.0707	7.0746	7.1958	6.7859	0.1189	0.1425	0.0866	0.4141		1	6.0077	5.8518	9.6067	11.0523	7.9322	6.2720	6.9395	6.7210	7.3614	5.0091	6.7320	6.5223
hsa-miR-3198	46.0495	35.4188	34.6434	37.2668	0.1190	0.0973	0.2104	0.3493		0	36.2395	32.7241	34.7280	36.4442	27.3975	28.7871	39.1370	30.2145	34.2239	49.1322	30.1318	37.1249
hsa-miR-376b	4.2913	3.3856	3.4029	3.3443	0.1191	0.1267	0.1135	0.7750		0	3.0534	3.0610	3.8611	3.4892	3.4080	3.6979	3.4234	3.4527	3.1798	2.8217	3.6330	3.2535
hsa-miR-532-3p	3.6813	4.1425	4.1832	4.0453	0.1212	0.0985	0.2326	0.3634		1	3.7936	3.4934	4.5451	4.7418	4.9078	3.7532	4.1115	4.0281	4.1205	3.7689	4.1596	4.2784
hsa-miR-181b	7.9937	9.1611	9.0825	9.3485	0.1225	0.1513	0.0909	0.4916		0	7.5302	7.7722	9.1766	9.7972	9.2949	8.9286	8.8426	9.6346	8.3922	8.4729	9.8369	10.7778
hsa-miR-186	10.3473	11.9287	11.9554	11.8650	0.1225	0.1208	0.1655	0.8815		1	10.3354	8.0503	11.4744	14.3617	13.6512	11.3094	12.7331	13.6659	11.6118	12.1009	12.3673	12.3327
hsa-miR-566	6.3022	6.9327	6.8179	7.2065	0.1238	0.2076	0.0444	0.1136		0	7.1374	8.0190	7.2281	7.8702	6.4564	7.1804	6.5087	7.2796	6.6548	6.8960	7.2476	6.5175
hsa-miR-150*	405.5600	494.5705	471.4132	549.7622	0.1284	0.2537	0.0272	0.0174		0	567.1483	739.3288	457.9290	544.5572	487.7059	576.9319	479.8323	585.3634	376.5595	433.3833	508.8890	492.6877
hsa-miR-3682	22.4306	19.8362	19.5679	20.4756	0.1290	0.0992	0.2931	0.4108		0	21.2641	19.0149	17.7816	17.4449	17.0477	19.4875	21.7161	18.8151	17.9525	24.2092	19.5800	23.2791
hsa-miR-3675-3p	9.8393	11.6770	11.8882	11.1736	0.1311	0.0982	0.3177	0.3852		1	12.4946	9.6443	10.9252	11.0475	10.2702	11.4516	11.0164	9.9605	15.0160	15.6505	11.2214	11.7021
hsa-miR-1323	7.3883	8.1367	7.9523	8.5762	0.1336	0.2593	0.0463	0.1463		0	8.0551	9.3313	8.1478	9.7180	7.6446	7.3354	8.4113	9.2092	7.6191	8.6998	8.6636	8.5292
hsa-miR-1202	1581.4772	########	1282.7719	1573.1420	0.1350	0.0434	0.9575	0.0029		0	1321.1225	1552.3833	1277.7817	1454.1737	1049.8388	1470.3360	1285.9558	1470.0550	1284.9931	1806.0548	1303.8751	1579.6644
hsa-miR-601	36.8787	32.1176	32.0531	32.2714	0.1353	0.1335	0.2035	0.9262		0	34.6922	32.6953	33.2127	32.7866	29.2103	27.1975	36.1818	33.1794	28.5494	39.5525	30.1223	29.5555
hsa-miR-223*	6.6679	5.6934	5.5724	5.9821	0.1375	0.1004	0.3107	0.1886		0	4.6539	5.6354	6.2587	7.3139	5.9234	5.4936	5.2834	6.8157	4.9723	5.2767	6.0760	6.2093
hsa-miR-711	11.9062	9.7897	9.5807	10.2879	0.1395	0.1100	0.2717	0.2920		0	10.2939	11.8144	10.5120	10.8051	8.9620	9.4092	9.1007	11.2151	7.4846	10.8968	10.9215	9.0559
hsa-miR-29a	163.1564	132.8085	133.4871	131.1910	0.1437	0.1562	0.1366	0.8035		0	124.1178	121.7902	134.3398	131.5979	137.1787	149.3609	110.2854	151.1116	134.1230	109.3164	152.0461	129.8993
hsa-miR-146a	86.7268	68.4922	73.0247	57.6900	0.1449	0.2727	0.0310	0.0118		0	74.5816	51.6669	77.4204	67.4170	79.5661	58.2059	69.4376	55.5974	80.5592	55.4758	71.2414	65.3715
hsa-miR-30e*	6.3180	5.7004	5.8036	5.4545	0.1456	0.2271	0.0764	0.2779		0	4.8199	4.6008	6.4387	7.2892	6.0302	4.8847	5.6994	5.7196	6.0163	4.6579	5.9850	5.9482
hsa-miR-3148	5.5645	6.7261	6.7447	6.6817	0.1470	0.1431	0.1867	0.8760		0	6.2028	6.3442	6.1122	7.8667	6.2919	7.0141	7.2112	6.4600	6.5165	6.0997	7.2869	6.3599
hsa-miR-324-5p	15.7368	13.4580	14.0097	12.1432	0.1474	0.2740	0.0370	0.0501		0	11.4644	9.3279	16.5418	17.4224	15.0525	10.6342	13.1199	12.5092	15.6403	10.9677	14.7360	12.1984
hsa-miR-92a	409.5549	516.9532	536.4645	470.4512	0.1481	0.0973	0.4208	0.1006		0	601.8253	362.3887	428.1428	358.9468	523.0487	466.6582	584.4369	579.8186	534.6021	561.9939	592.3119	517.8676
hsa-miR-30a	9.7012	10.6549	10.5024	11.0184	0.1485	0.2223	0.1013	0.3694		1	10.7990	8.1618	9.3347	8.6992	9.6641	12.3846	11.9590	13.0736	10.8178	12.5519	10.1649	11.0604
hsa-miR-629*	6.3972	6.9374	6.9314	6.9516	0.1495	0.1635	0.1718	0.9378		1	6.4975	7.9674	6.6991	6.5772	7.5455	6.5803	6.8223	7.3008	6.8523	6.3431	6.9784	7.6183
hsa-miR-3907	11.2612	12.3919	12.3927	12.3898	0.1499	0.1533	0.2326	0.9966		0	11.5584	11.3407	15.4803	16.3509	11.4440	9.3252	12.2128	12.2991	12.9696	11.8225	13.2197	15.1742
hsa-miR-1228	47.0943	55.2390	52.9143	60.7794	0.1541	0.3022	0.0515	0.1156		0	57.1366	57.2799	46.7025	59.9090	43.6395	63.6980	56.0192	59.4475	56.5039	74.3993	48.2246	59.2218
hsa-miR-3692*	11.6804	9.1594	8.8326	9.9382	0.1542	0.1122	0.3451	0.1967		1	8.4936	12.3065	8.5222	13.5486	8.8444	8.2087	10.0188	10.9973	7.5056	10.0677	9.8440	6.5703
hsa-miR-199a-5p	49.0636	38.3465	40.8639	32.3465	0.1579	0.2771	0.0395	0.0081		0	34.0024	26.2265	49.8334	47.8746	44.4892	31.8340	39.7416	30.3375	43.6914	28.7441	42.3907	31.0409
hsa-miR-598	4.8162	4.3385	4.2817	4.4736	0.1613	0.1227	0.3231	0.1712		0	3.9906	4.5736	4.8520	5.4635	4.6193	4.5869	4.1767	4.4517	4.2201	3.9122	4.1088	4.4873
hsa-miR-1469	6.5529	5.4638	5.2658	5.9356	0.1616	0.1049	0.4516	0.1208		0	4.6693	5.4962	5.1285	7.7323	5.3520	4.8808	5.2648	5.6396	4.7204	5.3128	5.4655	6.3681
hsa-miR-26a	179.5802	221.2803	241.9132	172.1050	0.1651	0.0536	0.8050	0.0015		1	197.0353	132.9010	243.3952	203.1596	262.3229	182.1529	268.0015	169.3243	291.2708	147.4053	259.4492	188.2982
hsa-miR-3138	66.3214	56.0630	52.0624	65.5978	0.1652	0.0608	0.9344	0.0311		0	57.8325	83.7703	55.2925	73.7896	46.7392	58.3266	52.8337	54.6344	44.7549	67.6128	58.9931	57.0157
hsa-miR-4253	22.0520	20.6028	19.5352	23.1472	0.1652	0.0218	0.4210	0.0013		2	21.7261	22.8340	18.0408	20.2126	17.7597	24.2848	20.6134	24.6306	18.5095	24.5977	19.1606	20.6460
hsa-miR-122	13.0785	8.9484	9.0179	8.7828	0.1679	0.1760	0.1654	0.8340		1	11.3655	11.3020	7.8498	5.6592	6.1957	11.9707	9.3557	7.5505	8.6585	10.8826	6.2682	8.6416
hsa-miR-181d	6.1350	6.6765	6.7034	6.6123	0.1706	0.1581	0.2572	0.7075		0	6.2872	5.8250	5.9836	7.4293	6.6865	6.4236	6.4706	6.4267	6.6244	6.8666	7.3226	6.3408
hsa-miR-133b	9.7091	7.9568	8.0379	7.7637	0.1730	0.1917	0.1493	0.5756		0	8.0818	8.5742	8.4433	7.1470	8.1455	7.5402	8.1136	7.5360	8.7381	10.4637	7.5320	6.8632
hsa-miR-595	7.7184	9.6744	9.6946	9.6263	0.1743	0.1727	0.2080	0.9224		0	8.7358	8.9204	8.1654	10.8799	9.1827	11.0594	10.6323	9.0792	9.9909	9.9781	10.0505	8.6842
hsa-miR-505	7.4513	7.7708	7.7719	7.7680	0.1771	0.1889	0.2256	0.9830		0	7.5659	7.5878	8.2647	8.6909	7.9196	7.9975	7.5136	8.1770	7.9821	7.8126	7.9183	7.4120
hsa-miR-340	16.7717	14.4174	14.6831	13.7839	0.1785	0.2390	0.1172	0.4312		0	12.5649	10.0190	15.6038	16.5894	16.9122	13.4868	13.7658	15.5859	16.0919	12.7564	15.2537	15.5260
hsa-miR-33a	4.9899	4.4344	4.5122	4.2488	0.1821	0.2511	0.0936	0.1746		0	4.0146	3.6764	4.9236	4.8580	4.6282	4.3116	4.4866	4.6053	4.5235	3.7564	4.7074	4.3051
hsa-miR-1224-5p	86.7740	72.9276	68.0404	84.5757	0.1823	0.0790	0.8465	0.0129		0	67.6487	89.3805	68.4794	94.1811	64.2211	66.0218	73.5703	77.6699	59.4180	93.4216	71.6887	76.5357
hsa-miR-320c	1290.3467	########	1045.1799	1216.5878	0.1841	0.1027	0.6189	0.0081		0	919.1138	1270.9672	1154.3319	1590.7618	1186.1194	1008.9293	1004.8278	1201.3653	839.8762	1006.3660	1151.0314	1292.7756
hsa-miR-140-5p	26.6117	21.9751	22.0446	21.8096	0.1842	0.1920	0.1879	0.8747		0	18.0666	18.1471	24.2575	25.1647	23.2856	21.5060	20.2727	25.2438	23.7342	18.2673	22.0428	22.1771
hsa-miR-21*	6.6035	5.6188	5.6696	5.4976	0.1842	0.2085	0.1462	0.4758		1	4.9305	4.4793	6.0661	6.4648	5.5486	5.1892	6.2608	5.8535	5.0537	5.6492	5.9430	5.1541
hsa-miR-671-5p	150.6249	166.4859	152.4049	200.0454	0.1845	0.8735	0.0111	0.0063		0	178.5919	222.4906	136.5469	161.4235	132.1391	190.8250	156.2023	221.5692	140.3258	219.7423	156.9383	160.2356
hsa-miR-584	39.0941	34.5806	32.7460	38.9531	0.1864	0.0665	0.9751	0.0773		0	34.1435	32.0526	29.7962	30.9005	32.0621	37.8632	37.4825	49.5631	32.6619	44.8893	30.8486	32.5933
hsa-miR-550a*	5.4864	6.1314	6.2713	5.7978	0.1916	0.1205	0.5333	0.0303		1	5.8367	5.7673	6.5424	6.9916	7.3115	5.2496	6.0515	5.7671	6.4056	6.0458	6.2149	5.2660
hsa-miR-1299	49.0151	36.7840	34.9885	41.0632	0.1927	0.1403	0.4197	0.1906		0	38.0262	28.1403	21.8817	29.4291	33.3723	45.2313	47.5741	44.2546	26.9010	50.1089	28.0108	29.1123
hsa-miR-1273d	9.1173	7.8427	7.7824	7.9865	0.1933	0.1808	0.2838	0.7419		0	7.2244	8.7020	9.9733	8.4552	7.4166	7.0376	6.5522	8.3064	6.2829	7.8572	8.9141	6.9373
hsa-miR-22	802.2910	701.9636	706.0339	692.2626	0.1949	0.2168	0.1778	0.7222		0	650.8231	561.6225	693.5801	735.7208	701.2909	793.8710	732.6199	761.6503	720.5325	680.4458	756.3613	700.0373
hsa-miR-3713	7.5227	6.7219	6.6910	6.7956	0.1993	0.1856	0.3137	0.8262		0	6.0820	6.1028	6.6544	7.9259	6.3529	5.1304	7.2332	5.9478	6.4212	7.8616	6.3539	7.5570
hsa-let-7e	14.7131	9.9026	10.3586	8.8158	0.2023	0.2475	0.1277	0.1327		0	7.1361	8.0405	13.1880	14.1367	11.8674	8.0032	9.9299	9.1807	11.1691	7.6785	10.5033	8.4626
hsa-miR-181c	5.2669	4.7752	4.8269	4.6521	0.2055	0.2577	0.1392	0.3773		0	4.1515	4.0170	4.9075	5.0648	5.2141	4.3652	4.9152	5.3137	4.7169	4.1453	5.1169	4.9707
hsa-miR-23a*	5.2747	4.4574	4.3692	4.6677	0.2060	0.1660	0.3607	0.2674		0	3.7713	5.0720	4.6964	4.6103	4.1502	3.9380	4.4381	5.0211	3.4690	4.7641	4.6189	4.2296
hsa-miR-936	28.4701	24.5280	23.5304	26.9054	0.2077	0.1230	0.6440	0.0897		0	21.2103	27.7148	29.5903	31.8080	22.3833	24.7387	23.5055	26.3682	19.1093	26.8524	23.2725	24.5579
hsa-miR-100	4.2973	4.6517	4.4738	5.0756	0.2095	0.5253	0.0166	0.0005		1	4.5831	4.3952	4.1640	5.3767	4.7200	5.5571	4.4290	5.5044	4.2868	4.9292	4.4154	4.9399
hsa-miR-4314	18.1414	15.3775	15.5704	14.9178	0.2098	0.2456	0.1682	0.5583		0	14.5032	14.9859	17.1515	12.9789	13.6096	12.4999	17.7005	14.0301	12.8684	18.6300	14.7877	15.3376
hsa-miR-663	146.8403	109.8485	113.0600	102.1942	0.2115	0.2525	0.1446	0.2298		1	141.8483	112.8285	92.2040	80.0429	87.4404	76.0264	123.8455	77.5311	110.7890	143.9383	100.0175	113.4334
hsa-miR-4323	10.2959	11.4774	11.5343	11.3419	0.2180	0.2024	0.3273	0.7710		0	11.5946	10.9882	10.2985	10.4500	10.8158	11.9779	10.9815	10.4370	13.1811	13.7016	11.4265	11.5580
hsa-miR-126*	11.3780	10.2704	10.1472	10.5640	0.2198	0.1816	0.3972	0.4476		0	9.9862	9.2483	11.0412	13.1364	10.0787	10.5350	9.8760	11.3640	11.0412	10.7287	9.7471	10.4943
hsa-miR-877	22.6186	19.9799	20.0420	19.8319	0.2245	0.2387	0.2384	0.8712		0	20.5566	21.1039	20.6871	25.1345	19.5251	17.0088	22.5246	20.8162	17.5656	17.8989	18.0235	16.5217
hsa-miR-361-5p	28.9687	26.0520	27.3592	22.9365	0.2300	0.5092	0.0287	0.0075		0	24.3831	20.6660	29.5294	25.2336	29.5065	22.6913	26.0005	24.7293	28.2815	20.0505	29.8499	24.1255

hsa-miR-3141	274.9312	239.4725	227.8308	267.2185	0.2305	0.1199	0.8042	0.0123		0	270.6039	311.4651	227.9088	219.8800	212.8430	266.6469	224.4203	262.7163	192.8458	280.5305	231.0227	233.0814
hsa-miR-3613-3p	10.3271	11.9694	11.9864	11.9289	0.2437	0.2423	0.2918	0.9427		0	13.6411	11.1007	10.6588	10.5248	10.4309	13.1891	11.2685	11.5295	14.2596	14.2509	11.7905	12.7245
hsa-miR-1183	68.2579	57.9901	58.3988	57.0159	0.2455	0.2669	0.2252	0.7285		1	57.8066	55.7756	56.0173	41.7527	52.8703	51.2365	69.2854	51.3075	52.3682	68.4541	56.2275	67.1193
hsa-miR-135b*	4.6937	3.3319	3.5220	2.8788	0.2463	0.3154	0.1323	0.0015		1	3.2531	3.3733	3.9074	3.2724	4.1104	2.7232	3.2354	2.8407	2.8764	2.2413	3.6973	2.6269
hsa-miR-32*	7.7838	9.6736	9.6202	9.8009	0.2500	0.2642	0.2459	0.8106		1	10.3104	9.7343	8.3969	12.0592	8.2846	10.4927	10.1099	10.5200	9.4350	9.8417	10.2330	7.5590
hsa-miR-3917	28.6275	25.6608	24.8065	27.6968	0.2592	0.1550	0.7310	0.0176		0	25.4255	29.5934	26.3193	31.2700	24.1623	26.0830	24.6856	28.5262	21.5044	24.5736	26.2238	24.3993
hsa-miR-1306	6.6680	7.4364	7.5246	7.2263	0.2680	0.2241	0.4513	0.4573		0	6.3853	5.8931	7.1431	8.7460	6.9748	6.8655	8.1967	6.8428	7.9097	7.8495	7.4381	7.2095
hsa-miR-99a	4.6540	4.3560	4.2554	4.5956	0.2690	0.1491	0.8349	0.0120		2	4.1327	4.1330	4.3529	4.6629	4.1803	4.9525	4.7203	4.9057	4.1226	4.7107	3.9689	4.5543
hsa-miR-21	1413.2721	########	1251.2836	1026.9273	0.2720	0.4446	0.0797	0.0572		1	1012.7155	877.4147	1552.0632	1390.3460	1383.5709	1111.5853	1130.4837	989.7028	1529.2840	851.4620	1179.4275	1106.3258
hsa-miR-3127	18.5107	16.0542	16.1606	15.8006	0.2738	0.2964	0.2447	0.6803		0	17.7299	14.8786	15.4963	14.4189	14.7490	12.9903	16.4236	15.4684	14.2178	19.2490	15.6327	16.0277
hsa-miR-501-3p	4.0323	3.7293	3.7393	3.7053	0.2758	0.2956	0.2583	0.7815		1	3.7466	3.6172	3.8377	3.9315	4.2390	3.3046	3.7398	3.8643	3.3180	3.7505	3.6937	3.7786
hsa-miR-520e	7.3856	8.4774	8.6922	7.9655	0.2814	0.2042	0.5680	0.0267		1	8.6040	8.7543	9.1153	8.9460	9.3045	8.9133	8.9930	7.5905	8.2417	7.4801	7.9704	7.3252
hsa-miR-516a-5p	15.5817	13.1128	12.5992	14.3368	0.2839	0.2027	0.6160	0.1993		0	11.3957	12.9220	11.7686	15.9569	10.5662	10.8465	13.7672	10.1907	12.6906	19.0638	11.2724	14.2049
hsa-miR-3937	49.6594	54.6524	52.2380	60.4069	0.2885	0.5767	0.0573	0.0226		0	58.3724	67.9066	49.4872	61.1191	48.8274	62.7375	56.9099	57.5297	48.7457	56.3145	49.9379	53.5944
hsa-miR-514b-5p	28.1762	24.6876	23.0266	28.6463	0.2904	0.1289	0.8998	0.0196		0	21.4811	34.1244	27.6394	33.9482	21.2517	24.6952	22.7866	28.7077	19.3449	30.6607	25.2388	22.2978
hsa-miR-4291	11.4311	10.1162	10.2153	9.8798	0.2911	0.3315	0.2433	0.6027		0	10.0052	7.4848	10.4378	10.7450	10.4145	11.1768	9.7644	11.5317	9.9327	7.5492	11.1137	11.0689
hsa-miR-3660	7.0936	6.3435	6.2772	6.5015	0.2928	0.2585	0.4603	0.6563		0	6.1646	7.2530	7.7978	7.2193	6.4127	5.6917	5.6516	6.2628	5.0306	5.7364	6.7205	7.4687
hsa-miR-4265	5.4865	4.6414	4.8038	4.2544	0.2961	0.3954	0.1406	0.0016		1	4.6170	4.1411	5.3701	4.6460	5.2890	4.4456	4.6419	4.0712	4.2640	3.9089	4.7207	4.0058
hsa-miR-149*	13.6236	15.3856	14.8183	16.7375	0.2979	0.4795	0.1097	0.1094		0	12.9064	17.6880	15.8053	22.1597	15.5220	13.5607	16.3328	17.3026	11.6489	16.3480	14.7955	15.5539
hsa-miR-3197	5.4880	5.0364	5.1629	4.7351	0.3010	0.4558	0.1134	0.0649		1	5.4610	4.4612	5.2256	5.1563	5.1862	4.2672	4.7851	4.1102	5.0075	5.5055	5.5006	4.8001
hsa-miR-502-3p	6.1666	5.7992	5.7844	5.8345	0.3049	0.2949	0.4220	0.8619		0	5.2122	4.7466	5.9106	6.6140	6.4203	5.3620	5.8391	6.5605	5.4204	6.3009	6.0528	5.6656
hsa-miR-3621	12.9789	11.2636	10.9544	12.0006	0.3193	0.2480	0.5805	0.1842		0	9.7627	13.7817	13.1025	12.6262	10.6536	9.7853	9.6036	12.8986	9.4660	10.8805	11.2579	11.6706
hsa-miR-4317	4.3944	3.9909	4.0141	3.9358	0.3254	0.3553	0.2848	0.6476		0	3.7551	3.7884	4.4100	4.7232	4.2693	3.7789	3.8716	4.1704	3.7805	3.2530	4.2775	4.0319
hsa-miR-1471	92.3389	82.2443	78.6854	90.7263	0.3283	0.1950	0.8810	0.0167		0	92.4163	106.1944	71.8538	73.3669	72.3170	84.9528	83.3176	91.1281	71.7604	85.7816	83.6632	89.3307
hsa-miR-617	5.2067	5.5318	5.4596	5.7036	0.3297	0.4492	0.1874	0.2799		0	4.7446	5.3070	5.9070	6.9426	5.4824	5.3502	5.6205	5.6657	5.0259	5.9552	5.2822	4.9669
hsa-miR-1246	602.8381	493.4420	464.8249	561.6462	0.3329	0.2305	0.7182	0.0266		1	499.2780	732.9578	500.3019	563.5733	462.5882	601.6356	373.9389	603.9824	408.3802	409.4874	573.4966	533.0067
hsa-miR-631	7.3716	7.8926	7.7974	8.1194	0.3392	0.4349	0.2165	0.3337		0	8.4024	8.9912	7.3140	6.5571	8.2527	9.3059	7.5235	7.9348	8.0512	7.8373	7.5801	7.7017
hsa-miR-2276	76.6235	90.3277	85.4701	101.9051	0.3396	0.5344	0.1196	0.0656		0	88.5453	119.4549	90.6501	123.3745	87.3525	79.5427	89.4186	114.9559	69.6564	99.4449	95.0689	84.7366
hsa-miR-141	5.7387	5.4017	5.4306	5.3328	0.3401	0.3853	0.2754	0.5476		0	5.1679	4.6265	5.1297	4.9501	5.2848	5.1710	5.8141	5.4677	5.1564	5.5738	5.5693	5.5321
hsa-miR-1225-5p	1307.3709	########	1332.9875	1600.3846	0.3494	0.8170	0.0279	0.0012		1	1309.1926	1596.4456	1517.2308	1982.5204	1323.3304	1611.6634	1275.3810	1555.2876	1250.5295	1427.9710	1417.9667	1560.8809
hsa-miR-186*	8.3208	7.0252	7.2798	6.4182	0.3545	0.4548	0.1858	0.0026		1	7.1244	6.2322	7.2050	6.7156	8.5370	6.8380	6.7396	6.5800	6.3274	5.5904	7.4748	6.6620
hsa-miR-92b	9.6674	11.0669	10.6412	12.0814	0.3570	0.5199	0.1563	0.1210		0	10.5770	12.1220	10.0565	11.6524	9.7957	12.5578	10.5340	12.2850	11.8781	13.4742	10.6482	12.7923
hsa-miR-629	4.7364	4.3837	4.3422	4.4826	0.3618	0.3146	0.5368	0.5035		0	3.9523	4.5023	4.7385	5.5289	4.7500	3.7379	4.1919	5.1790	3.6690	4.5316	4.6916	4.0953
hsa-miR-29b	25.9742	28.2635	28.9180	26.7038	0.3693	0.2646	0.8063	0.3224		0	22.8524	16.5680	27.3385	34.3173	31.2570	27.5528	29.6766	28.2470	32.4423	22.5758	30.3820	31.1661
hsa-miR-340*	5.5748	5.9477	6.0461	5.7130	0.3704	0.2679	0.7453	0.0842		0	5.6502	5.5195	6.3450	6.4274	6.0800	5.8855	6.3714	5.7345	6.7453	6.1233	5.8012	5.7792
hsa-miR-557	56.1322	65.1469	59.9469	77.5401	0.3779	0.7040	0.0870	0.0319		0	47.2596	73.7612	62.0971	120.4620	68.7912	60.4448	68.7965	84.1418	53.1833	55.8668	65.8418	62.1800
hsa-miR-1307	11.4187	12.4077	12.3850	12.4618	0.3901	0.4057	0.4124	0.9176		1	10.9386	12.0837	14.5783	17.4822	12.8550	9.4381	13.9006	14.6795	10.4143	11.0722	12.4151	11.3745
hsa-miR-33b*	21.5691	18.7645	18.5265	19.3315	0.3947	0.3592	0.5170	0.5862		0	20.5930	17.5769	15.2050	16.9987	14.5468	21.4851	17.9936	17.4437	22.9801	24.1533	17.7382	19.7343
hsa-miR-602	18.6423	16.8494	16.6209	17.3939	0.3953	0.3422	0.5823	0.5043		1	19.0652	14.2315	13.9579	15.0290	13.1920	18.8018	17.0596	16.0962	19.5740	22.6432	15.9313	18.1649
hsa-miR-3149	9.4127	10.8551	10.9955	10.5204	0.3963	0.3563	0.5317	0.5293		0	12.0253	11.3971	10.1753	12.3228	10.3346	11.3049	11.3741	9.9657	10.3004	10.1862	10.9426	9.9488
hsa-miR-939	209.8281	227.1839	219.4017	245.7316	0.3972	0.6378	0.1232	0.0394		0	228.6976	230.6818	193.7228	198.4297	201.1894	229.2773	236.5546	248.4668	201.5625	272.6831	203.9438	256.3617
hsa-miR-3195	153.8743	167.5031	158.9573	187.8706	0.3977	0.7505	0.0771	0.0193		0	184.0235	221.8424	148.6972	167.7246	141.6747	188.5750	167.0098	188.1120	144.6185	188.5289	149.7390	164.4120
hsa-miR-4306	234.9667	206.1806	208.3746	200.9515	0.4026	0.4412	0.3452	0.6146		0	238.2607	195.8249	194.8126	232.2692	207.2615	188.8024	221.5029	236.4310	199.8778	221.5386	214.6720	172.8386
hsa-miR-335	9.6174	8.7756	9.0833	8.0424	0.4074	0.6058	0.1616	0.1371		0	8.3045	7.0161	9.6685	9.9511	9.6810	8.3226	9.1547	8.6381	9.4667	7.2457	9.1425	9.5995
hsa-miR-2278	5.0151	5.4637	5.5319	5.3012	0.4087	0.3470	0.6074	0.2949		0	5.3720	4.7184	5.0229	6.2221	5.4042	5.2656	6.1505	5.5111	5.3706	5.2858	5.4949	4.8315
hsa-miR-7-1*	5.1676	4.9472	4.9301	4.9878	0.4121	0.3824	0.5316	0.6975		0	4.7722	5.0916	5.4846	5.9388	5.0312	4.9189	4.5377	4.8154	4.9555	4.7139	5.0357	5.1965
hsa-miR-1229	8.6584	9.4353	9.3657	9.6012	0.4140	0.4570	0.3726	0.6826		0	10.2331	8.2653	8.9379	10.2142	8.7110	11.0966	9.1913	8.9017	10.4961	11.8941	8.5841	8.9933
hsa-miR-484	19.3499	20.5336	20.7232	20.0818	0.4170	0.3538	0.6475	0.4943		0	17.9700	14.9022	21.7581	24.4167	22.3150	19.0676	23.3324	21.7909	20.4451	21.9194	20.8864	19.8826
hsa-miR-630	567.7592	499.3581	520.8109	448.2288	0.4206	0.5812	0.1867	0.0660		4	700.2833	434.5156	453.8715	205.0130	419.8712	462.4153	501.7847	294.2877	528.0895	663.6949	422.5867	521.8937
hsa-miR-331-3p	31.8696	34.9854	37.5965	28.7624	0.4267	0.1630	0.4517	0.0003		2	30.1961	27.4967	45.3658	39.8475	44.0489	26.0760	36.7505	29.8008	43.4847	25.6735	37.3596	28.0227
hsa-miR-425	83.5406	92.0368	98.7059	76.1422	0.4286	0.1747	0.5015	0.0000		2	89.5393	67.2992	102.9550	84.3916	112.5660	68.0047	93.8014	83.5362	99.2188	75.4028	114.8048	79.9339
hsa-miR-513a-3p	5.7953	5.4489	5.4834	5.3666	0.4290	0.4783	0.3459	0.4980		0	5.7965	5.1036	5.8088	5.4156	5.4791	5.1618	5.5329	5.5794	4.9567	5.3428	5.2294	5.9177
hsa-miR-500a*	6.8568	7.1327	7.0607	7.3044	0.4293	0.5654	0.2820	0.4208		0	6.7252	6.3370	6.7398	7.7654	7.9248	7.1247	6.9957	8.0654	6.6856	7.6400	7.2862	7.3207
hsa-miR-3194	51.6226	47.5236	44.5596	54.5878	0.4308	0.1832	0.6359	0.0213		0	43.0901	53.3367	47.4251	61.5886	41.1578	46.4445	49.3693	49.4785	40.7000	63.5158	42.3263	52.9136
hsa-miR-32	3.6742	3.8059	3.8131	3.7886	0.4333	0.4272	0.5459	0.8620		0	3.6594	3.4958	3.9094	4.1295	4.0682	3.7628	3.6561	3.7794	4.0116	3.4693	3.8353	4.0103
hsa-miR-3945	20.4069	22.4142	20.8664	26.1034	0.4347	0.8570	0.0748	0.0202		0	18.0331	29.5639	24.1685	39.7078	21.0650	19.3754	20.8011	23.5435	17.4853	22.6091	23.2298	28.2899
hsa-miR-362-3p	5.9652	5.7413	5.6041	6.0684	0.4373	0.2231	0.7391	0.0077		1	5.7829	5.7768	5.6045	6.4398	5.9822	6.0238	5.4251	6.3083	5.5040	6.0662	5.5576	6.1401
hsa-miR-3188	119.8231	129.0715	126.9856	134.0428	0.4379	0.5488	0.2902	0.3690		0	146.0965	149.2987	115.6831	113.4662	118.3552	112.8438	129.9012	128.6770	119.9913	151.0921	126.5275	150.5170
hsa-miR-195	6.7997	6.4332	6.4931	6.2902	0.4421	0.5278	0.3344	0.5382		0	5.8959	5.3849	7.2136	7.8428	6.9460	6.2195	6.1242	6.8094	6.7614	6.0885	6.5176	6.5200
hsa-miR-3938	6.1356	6.4297	6.5857	6.0578	0.4503	0.2612	0.8506	0.0204		2	6.4540	5.4256	6.4659	6.3854	6.7965	6.0687	6.7742	6.1828	6.0441	6.2570	6.4631	6.0827
hsa-miR-106b	178.6710	207.9190	217.2253	185.7389	0.4529	0.3361	0.8554	0.0579		0	163.9883	129.0442	184.2145	173.1704	233.6948	168.7598	228.5783	215.8949	241.5146	216.0113	265.4913	179.9602
hsa-miR-1228*	3.5081	3.8471	3.9030	3.7140	0.4535	0.3897	0.6608	0.3740		0	3.4940	3.3909	3.6129	4.9780	3.9911	3.4200	4.3373	3.8597	3.4233	2.8961	4.1367	3.5918
hsa-miR-940	222.9117	200.3905	195.1686	212.8358	0.4548	0.3626	0.7569	0.2988		0	244.5236	198.4406	154.0408	124.1415	160.5265	176.0165	186.5350	186.8429	214.2851	324.1695	167.0230	263.5468
hsa-miR-1290	61.1831	52.6223	48.4752	62.5061	0.4552	0.2779	0.9101	0.0035		0	51.4116	79.1619	50.1479	65.4588	46.5540	68.4947	42.0514	65.5745	47.1871	42.2041	58.5103	63.6000

hsa-miR-18a	18.4508	19.9160	21.2379	16.7655	0.4567	0.1880	0.4375	0.0110		1	16.1951	10.9437	25.0111	25.7593	24.2556	14.6346	21.0419	18.9842	23.9931	14.5218	23.3354	16.7919
hsa-miR-371-3p	6.1217	5.7719	5.6464	6.0711	0.4568	0.3214	0.9214	0.1531		0	5.6355	7.6010	5.9334	5.7260	5.9701	5.3095	5.9629	5.5250	5.7871	6.1338	5.0787	6.1660
hsa-miR-30c-2*	7.1919	6.7800	6.5598	7.3049	0.4666	0.2733	0.8578	0.0501		0	6.2775	8.1850	7.1017	8.0751	6.3541	6.3281	6.3462	7.1136	5.9912	8.4090	7.0575	6.3365
hsa-miR-3185	5.6985	5.1987	5.2120	5.1672	0.4722	0.4870	0.4537	0.8489		1	4.5179	4.9236	5.6625	6.7556	5.7311	4.3800	5.1271	5.1437	4.3206	4.3327	5.0099	5.2063
hsa-miR-125b-1*	3.1439	3.3846	3.3915	3.3682	0.4774	0.4723	0.5291	0.8975		0	3.0054	3.4175	3.8179	4.0906	3.6092	3.1242	3.3266	3.2877	2.9547	2.8626	3.9562	3.6493
hsa-miR-101	50.3742	55.0455	57.2327	49.8328	0.4776	0.3205	0.9369	0.0975		1	48.7923	27.6635	50.4349	55.6837	66.5642	50.7445	56.8131	58.3430	59.0732	51.3870	64.0022	51.1359
hsa-miR-636	8.1093	8.8458	9.1198	8.1928	0.4778	0.3387	0.9398	0.1043		0	9.6410	7.6434	9.0349	7.7493	7.8113	7.9481	9.4257	7.4891	9.3811	9.3855	8.4468	8.7552
hsa-miR-374a	25.4400	27.9017	28.8568	25.6255	0.4817	0.3473	0.9608	0.1939		0	20.3476	18.0248	32.5832	37.1812	32.9499	24.7431	29.1214	27.2231	35.5720	22.7383	30.4008	28.1296
hsa-miR-1234	33.1266	36.3204	35.3740	38.5759	0.4823	0.6214	0.3004	0.3308		0	39.2011	36.5762	30.5158	38.7976	28.5288	40.9301	34.0480	36.0916	40.8657	48.4466	32.5865	37.2358
hsa-miR-181a	41.7537	46.3419	48.3887	41.4636	0.4858	0.3229	0.9657	0.0132		1	36.9201	31.1793	45.8045	42.4741	50.5469	33.0795	53.8226	45.6397	51.0498	42.7285	54.3698	49.4545
hsa-miR-151-3p	39.8161	36.8247	39.5179	30.4058	0.4909	0.9460	0.0525	0.0010		0	32.1762	23.4155	42.5134	36.4597	40.2498	28.1387	45.0294	35.0025	41.1254	27.7968	44.9476	30.7477
hsa-miR-454	4.5481	4.8102	4.7647	4.9188	0.4938	0.5821	0.3941	0.6133		0	3.6290	4.0480	4.8497	6.9769	5.5193	4.3308	4.8543	5.5926	4.9826	3.9541	4.9174	5.0129
hsa-miR-30c-1*	7.5448	8.0441	7.6492	8.9851	0.4973	0.8869	0.0884	0.0060		1	7.0832	9.8770	8.3868	11.7163	7.8129	7.4702	7.8868	8.6043	6.6070	8.7328	8.2828	8.3671
hsa-miR-134	256.6840	275.7268	273.3839	281.3105	0.4977	0.5550	0.4120	0.5885		0	324.7254	351.7259	273.1720	261.0102	251.2476	249.8891	272.2559	270.1624	230.4480	280.8246	283.5701	263.6193
hsa-miR-610	12.7310	12.0578	11.7948	12.6846	0.5075	0.3633	0.9683	0.2341		0	10.5826	11.2022	11.6244	15.5130	11.4606	10.8644	13.6152	12.5256	10.2084	13.5169	11.7001	12.7569
hsa-miR-582-5p	6.0130	5.6997	5.6061	5.9228	0.5078	0.3938	0.8600	0.2196		0	5.4487	5.4323	5.5462	7.4522	5.6761	5.4540	5.0603	6.3815	5.6865	5.4137	5.9825	6.5221
hsa-miR-1180	4.5763	4.8470	4.8967	4.7285	0.5098	0.4442	0.7271	0.4749		0	4.2130	4.7652	4.3839	5.5962	4.9317	4.1980	5.4136	4.8316	4.3774	3.8455	5.1849	4.7671
hsa-miR-1470	11.5909	10.7421	10.7238	10.7855	0.5111	0.5041	0.5593	0.9236		1	12.4313	9.1613	9.4000	9.9864	9.5680	11.7473	10.1743	10.3532	12.5305	13.4648	9.5609	10.9236
hsa-miR-1911	7.1248	6.2297	6.3879	5.8529	0.5134	0.5900	0.3628	0.0790		1	6.0370	6.8699	6.8750	6.2482	7.7019	5.9942	6.5656	6.3636	5.1779	4.5628	6.1808	5.2161
hsa-miR-26b	125.5162	106.9866	107.3078	106.2213	0.5178	0.5287	0.5132	0.9242		0	82.3526	92.0225	108.9198	155.5180	124.7068	106.9082	101.1975	108.8115	132.7990	85.3792	101.9593	111.9107
hsa-miR-3656	1884.5291	########	1823.9500	1806.2715	0.5187	0.5564	0.4694	0.7365		0	1923.4867	1871.4469	1708.3905	1500.2021	1772.4799	1785.0204	1853.2903	1883.7574	1725.2509	1811.8921	1711.0272	1768.5518
hsa-miR-98	8.9196	8.3292	8.3794	8.2094	0.5187	0.5663	0.4866	0.8072		0	5.8877	7.3504	10.4635	12.9755	9.3429	7.9624	8.3267	8.9599	9.0300	6.4503	8.2008	8.1807
hsa-miR-708	9.5079	8.6004	8.7783	8.1766	0.5256	0.6094	0.3639	0.1073		1	8.5015	9.0585	9.2968	8.3447	9.8160	8.2133	7.9714	8.6870	7.8627	6.7113	9.0172	8.1085
hsa-miR-1914*	45.5349	42.7606	41.2889	46.2682	0.5288	0.3403	0.8886	0.1587		0	37.8808	43.4575	42.9245	51.4383	35.9422	42.0349	45.7161	41.6916	39.9495	55.8516	38.6651	40.3214
hsa-miR-320d	212.0322	199.9630	199.9947	199.8873	0.5297	0.5312	0.5681	0.9925		1	207.2296	163.6484	179.7473	177.7613	201.5337	218.6982	224.4815	242.0410	188.7258	213.1442	201.9183	181.0548
hsa-miR-29c	147.2401	140.6954	143.0053	135.1900	0.5310	0.6951	0.2970	0.3248		0	134.1799	119.8098	131.5127	121.3368	146.1143	147.8256	135.1529	156.8741	143.3393	118.0600	160.6861	130.3697
hsa-miR-483-5p	297.0540	271.2100	260.8373	295.9317	0.5337	0.3896	0.9795	0.0762		0	296.2397	386.2163	298.3733	281.7413	241.0066	267.9047	244.3199	298.8904	203.9427	290.9885	286.2211	240.8449
hsa-miR-10a	7.0361	7.2453	7.1933	7.3691	0.5356	0.6538	0.3737	0.5070		0	7.0291	7.4078	7.0029	7.1770	7.5751	8.2819	7.6793	7.4171	7.7947	7.5948	6.7076	7.4769
hsa-miR-342-3p	44.8237	47.9269	51.2248	40.0669	0.5361	0.2203	0.3771	0.0004		2	50.5717	30.5119	44.1770	38.4254	50.7057	39.7922	52.9788	41.4666	57.7310	36.0676	51.6633	51.4767
hsa-miR-505*	3.6341	3.4791	3.4946	3.4422	0.5485	0.5912	0.4833	0.6709		0	3.1481	3.3575	3.7902	4.0414	3.7011	3.3791	3.4760	3.6916	3.4364	3.2168	3.6094	3.2805
hsa-miR-107	258.7467	238.4650	247.7212	216.4042	0.5590	0.7544	0.2500	0.0895		0	231.1258	197.9988	237.6965	267.3374	261.9073	212.5230	278.2406	228.2182	266.4097	201.5313	249.5603	222.3795
hsa-miR-3162	582.9482	562.3390	545.5841	602.2714	0.5611	0.3079	0.6522	0.0854		1	592.6663	612.1213	519.4652	461.8929	456.7717	641.9350	531.8107	620.1884	515.9322	639.8368	536.6677	566.6003
hsa-miR-99b	7.7069	7.1467	7.6010	6.0639	0.5681	0.9142	0.1190	0.0002		2	6.2989	5.1460	7.9040	6.8390	8.3249	5.7630	7.3745	6.0428	8.0403	5.6188	8.3714	6.8186
hsa-miR-15a	262.6199	239.1893	238.2767	241.3644	0.5723	0.5630	0.6221	0.8755		0	221.2295	201.0848	210.2530	291.6628	249.4495	258.7079	249.7783	261.1190	255.2207	206.4065	248.3452	260.4635
hsa-miR-532-5p	6.2643	6.5183	6.5205	6.5131	0.5735	0.5821	0.6219	0.9830		0	5.9176	5.1739	6.5530	7.5992	7.5641	5.9881	6.2511	7.0884	6.4154	6.2666	6.6773	6.7340
hsa-miR-762	1737.8575	########	1423.5742	1918.8081	0.5820	0.3104	0.5797	0.0011		1	1707.7378	2108.1974	1414.4107	2303.4391	1549.2057	1995.5381	1552.0206	1840.9265	1218.1072	1656.7106	1424.1964	1568.3564
hsa-miR-659	15.2676	14.2582	13.7299	15.5173	0.5897	0.4185	0.9083	0.2106		0	11.6680	17.3133	14.5903	22.0029	14.8284	13.4651	13.9330	14.8576	12.0231	13.7092	15.0468	13.1101
hsa-miR-769-5p	5.3960	5.5532	5.5699	5.5133	0.5928	0.5575	0.6980	0.6178		0	5.2599	4.9841	5.7228	5.5427	5.5209	5.3337	5.5365	5.3966	5.7109	6.0009	5.6588	5.6597
hsa-miR-3614-5p	19.8730	20.6921	20.5498	21.0313	0.5980	0.6672	0.5293	0.7096		0	21.7090	21.4130	19.5192	21.7721	18.2196	21.2508	20.5116	21.1368	22.1611	25.1699	21.2115	18.0790
hsa-miR-572	400.4877	426.5820	436.3100	403.3969	0.6011	0.4818	0.9566	0.2653		1	494.9982	394.9173	398.8739	270.5618	378.9795	356.1770	423.1891	337.0528	442.6509	541.3007	428.6028	517.9722
hsa-miR-2116*	19.3561	17.8016	17.7197	17.9968	0.6061	0.5898	0.6716	0.8527		0	18.6364	16.0689	15.1497	16.3549	14.4512	19.1682	17.6818	15.8004	21.8860	23.0185	16.3605	20.1297
hsa-miR-103	241.6976	260.3910	285.2967	201.0325	0.6111	0.2609	0.2949	0.0007		1	212.1106	180.1039	314.4005	266.7250	335.6405	183.5214	275.2186	197.0126	364.5950	179.6009	312.7287	217.2104
hsa-miR-125a-5p	15.6628	13.9049	14.9785	11.3463	0.6150	0.8453	0.2357	0.0026		1	11.3458	10.9617	16.0832	15.7704	20.6709	9.2571	14.6479	11.8680	15.6522	10.1344	14.6780	12.2655
hsa-miR-374c	4.1302	4.2775	4.2797	4.2723	0.6178	0.6169	0.6471	0.9600		1	3.6855	4.4322	4.8491	5.1037	4.6760	4.2915	4.2975	4.3237	4.5354	3.6385	4.1446	4.0838
hsa-miR-4322	31.9554	30.2843	29.6794	31.7260	0.6324	0.5181	0.9531	0.3574		0	28.2769	32.9532	30.5703	39.0940	29.1927	27.0012	32.1569	31.3317	26.6604	30.9857	28.6519	29.0158
hsa-miR-4310	20.1466	18.4474	18.2245	18.9786	0.6345	0.5929	0.7566	0.6432		0	19.6308	16.8590	14.9668	16.8665	14.8880	20.8333	17.0580	17.1601	22.4801	23.5084	16.7034	21.1186
hsa-miR-132	4.7553	4.5887	4.5618	4.6527	0.6431	0.5935	0.7987	0.6995		0	4.0673	4.4330	4.8139	5.0573	4.9657	4.9814	4.5294	5.3947	4.2011	3.7874	4.6247	4.4591
hsa-miR-563	14.6282	13.6697	13.3463	14.4407	0.6503	0.5470	0.9340	0.3062		0	14.0284	12.0410	11.7724	13.0335	10.5432	16.0415	13.5033	12.9846	16.4203	18.7466	12.8058	15.9416
hsa-miR-326	9.7532	9.3163	9.6526	8.5147	0.6525	0.9179	0.2238	0.0022		0	9.7397	7.8734	10.3315	9.7881	10.1676	8.4069	9.8988	8.6299	10.0084	8.3485	9.4384	8.4183
hsa-miR-3190	13.6496	13.1096	12.8710	13.6784	0.6618	0.5354	0.9829	0.2952		0	15.4507	12.6967	10.8908	9.2939	11.4441	15.0351	11.5000	13.2335	12.3330	16.0063	13.0162	12.6074
hsa-miR-1207-5p	2029.7231	########	2058.4093	2178.3687	0.6622	0.8464	0.3507	0.1467		0	2152.2051	2453.5578	2015.5919	2191.3353	1957.8587	1877.5164	2038.9734	2095.8478	1916.4120	2076.5364	2044.0435	2149.9469
hsa-miR-3652	142.5320	135.0502	134.2478	136.9624	0.6660	0.6345	0.7654	0.7643		0	134.5925	135.6890	138.8809	113.4638	119.4751	122.3347	142.2190	136.1398	114.0152	157.3735	129.1318	128.7025
hsa-let-7i	213.9619	202.9983	205.8869	196.1140	0.6710	0.7617	0.5255	0.5928		0	165.1736	174.2192	211.5925	276.3656	230.7797	200.9421	190.1456	197.9004	243.5909	167.2363	228.3194	186.4871
hsa-miR-3667-5p	52.8922	55.4309	52.9184	61.4190	0.6772	0.9966	0.2342	0.0667		0	53.2486	69.8164	56.9994	74.2098	48.4426	45.6140	55.4034	53.6997	43.9933	69.4633	56.1409	61.6402
hsa-miR-215	7.2899	7.0303	6.9360	7.2551	0.6803	0.5791	0.9580	0.2881		0	6.3755	6.0267	6.8313	8.8450	7.6131	7.5871	6.4039	7.5197	6.7863	6.8758	6.9020	7.6447
hsa-let-7d*	24.1751	25.1395	26.0172	23.0478	0.6936	0.4617	0.6809	0.0851		1	26.9096	32.1751	24.2152	16.5451	33.0818	22.8319	26.8886	21.3593	24.5936	26.5881	25.4806	20.4710
hsa-miR-23b	22.7300	23.9049	25.4349	20.2584	0.6970	0.3892	0.4445	0.0102		0	19.4120	20.0442	30.8441	28.2253	26.0048	19.3310	24.5945	20.3760	31.2390	18.0131	26.1952	21.3174
hsa-miR-654-5p	7.1716	6.9701	7.0303	6.8264	0.6981	0.7878	0.5279	0.4151		1	6.0059	6.7250	8.1039	7.9764	7.9820	6.4810	6.8011	7.2903	6.1202	6.2635	6.9336	6.2375
hsa-miR-1285	4.9486	4.7708	4.6205	5.1288	0.6982	0.4803	0.7144	0.0377		0	4.4072	5.2536	5.1286	5.9454	4.9437	4.8083	4.1116	5.5276	3.9126	4.5707	5.4304	4.7891
hsa-miR-142-5p	69.9006	73.4102	75.1635	69.2316	0.7026	0.5760	0.9451	0.2607		0	59.3889	41.8406	68.5763	72.9917	78.1136	69.6551	77.1679	85.6618	78.1694	60.8800	91.2395	77.2299
hsa-miR-660	12.9521	12.4202	12.0331	13.3427	0.7070	0.5255	0.7983	0.1353		0	11.5174	10.2622	11.5203	15.6688	14.0164	13.6735	12.0705	15.5846	11.6179	13.1925	12.4078	13.2435
hsa-miR-1237	16.4844	17.2049	17.2932	16.9946	0.7097	0.6785	0.8117	0.8048		0	18.2175	14.8701	15.3285	16.8205	14.8352	17.4141	16.9149	16.5967	20.4725	20.1724	16.7399	17.2193
hsa-miR-1268	452.3741	438.0017	422.7889	474.2587	0.7164	0.4612	0.6071	0.0149		0	459.1621	536.0911	422.2524	472.8679	399.8420	452.4127	422.8276	447.9749	367.7688	470.1017	444.7809	431.4146
hsa-miR-4269	5.8001	5.6526	5.6643	5.6247	0.7176	0.7417	0.6824	0.8393		0	5.3918	5.7849	5.6791	5.8186	6.2929	5.3847	5.6321	5.7228	5.2760	5.1072	5.6015	5.5146

hsa-miR-23c	17.2845	18.2662	18.1204	18.6139	0.7179	0.7601	0.6514	0.7444		0	20.0559	16.2021	15.3762	16.5763	14.2292	20.4791	18.5214	17.3349	21.9045	24.0747	16.8904	19.6802
hsa-miR-20b	60.9464	66.2761	68.1774	61.7448	0.7227	0.6349	0.9583	0.2621		0	53.9818	46.1450	70.0362	82.0960	76.8629	62.0035	68.2691	67.4796	78.2347	57.2456	67.9554	61.4265
hsa-let-7g	156.5704	146.9753	148.0896	144.3196	0.7343	0.7683	0.6790	0.7995		0	115.5278	134.1992	154.9432	198.5998	162.3176	145.5964	144.1106	142.9058	181.6864	117.1790	150.0936	152.1910
hsa-miR-30b	57.4745	54.2947	56.0684	50.0674	0.7346	0.8826	0.4509	0.1949		1	45.4537	46.9488	62.8260	69.6312	62.0202	47.9251	53.3308	48.8416	67.8592	41.6908	57.6295	53.3923
hsa-miR-548c-5p	5.9403	6.0578	6.0684	6.0325	0.7371	0.7171	0.8047	0.8439		1	6.3363	6.0553	6.1354	7.1954	5.7264	5.6189	5.9124	5.7582	6.1194	6.5363	5.9765	5.9519
hsa-miR-373*	8.8588	9.0713	8.7376	9.8668	0.7388	0.8498	0.2143	0.0654		0	8.0940	8.7597	8.0594	12.6980	9.0697	8.6902	9.6578	11.1625	7.8804	10.3061	8.6359	7.7962
hsa-miR-202	52.4956	50.1732	49.2818	52.2976	0.7403	0.6502	0.9793	0.4694		0	55.2308	59.9195	57.2073	39.6191	40.0941	36.6850	43.7774	51.6040	43.8983	54.4664	53.7717	64.1477
hsa-miR-3663-5p	12.0391	11.6188	11.2151	12.5809	0.7441	0.5293	0.7075	0.1227		0	10.2071	12.4822	13.6564	14.6047	9.7724	10.3466	10.9970	11.8467	10.1011	13.9588	11.5966	11.9463
hsa-miR-887	13.7384	14.1977	14.3434	13.8504	0.7448	0.6708	0.9424	0.5595		0	15.1972	14.4248	14.6563	15.2672	14.7411	12.4428	15.0058	14.7846	11.4447	13.7502	14.8463	11.7670
hsa-miR-486-3p	7.2845	6.9855	7.0452	6.8432	0.7455	0.7962	0.6475	0.6117		0	6.0463	6.2272	8.0288	9.6035	7.7000	7.2665	7.4351	7.3620	7.0389	5.8743	6.8892	5.9381
hsa-miR-551b	6.2504	6.3720	6.5924	5.8468	0.7473	0.3816	0.3112	0.0005		2	6.0743	5.4167	6.3285	6.0545	7.2442	5.6492	6.5193	5.4613	6.8837	6.0738	6.3262	6.1246
hsa-miR-221	44.6933	43.2157	45.3424	38.1470	0.7473	0.8905	0.1996	0.0282		0	41.6328	32.5764	53.5844	46.6260	46.4229	39.3679	44.6230	36.9828	49.4974	33.0221	45.0941	42.8040
hsa-miR-301b	3.6189	3.5676	3.6908	3.2738	0.7481	0.6585	0.0621	0.0004		1	3.4306	3.0631	3.9422	3.7764	3.7713	3.0243	3.7223	3.5038	3.8192	3.1802	3.8008	3.4586
hsa-miR-18b*	8.5438	8.8409	8.6578	9.2773	0.7503	0.9028	0.4852	0.2873		1	9.8952	7.7762	8.0581	9.7226	7.4435	8.8010	8.5941	9.9699	9.7556	10.8464	8.2224	9.9806
hsa-miR-127-3p	6.9601	6.7484	6.7937	6.6405	0.7531	0.8056	0.6598	0.6664		1	6.1088	5.8659	7.6024	7.6615	6.7329	7.0778	7.5404	6.8469	7.4272	5.8233	6.8191	6.3617
hsa-miR-642b	199.4314	188.2209	176.1960	216.8802	0.7541	0.5194	0.6537	0.0329		0	156.5550	265.7587	182.8216	270.2651	200.5434	203.2578	169.4023	233.2283	157.9208	156.3970	199.2564	193.3853
hsa-miR-25	262.2609	244.7949	243.7378	247.3143	0.7541	0.7417	0.7932	0.8565		0	240.6679	242.3937	235.4404	278.7899	253.1590	226.8104	248.5101	295.3672	239.4655	231.7874	250.6782	257.9739
hsa-miR-125b	4.7375	4.5942	4.5965	4.5889	0.7560	0.7612	0.7555	0.9663		0	4.2227	3.8452	4.8759	5.0787	4.9644	4.5744	4.4132	4.9596	4.5910	4.3161	4.4360	4.8417
hsa-miR-126	264.4661	252.0950	264.2275	223.1793	0.7595	0.9955	0.3381	0.1446		0	234.5320	202.3442	325.7689	289.4095	314.0287	236.2783	240.5749	208.2109	329.1937	177.2356	237.4752	227.9823
hsa-let-7f	171.3941	162.0541	167.6368	148.7485	0.7603	0.9056	0.4884	0.3581		0	104.7872	149.6537	206.6666	234.8364	191.6980	156.0707	178.1137	149.7001	209.0467	114.3941	156.7452	142.8965
hsa-miR-301a	15.1403	15.7115	16.5797	13.6422	0.7619	0.4608	0.4569	0.0138		1	12.6557	9.5888	17.4100	19.1618	19.6408	12.4610	16.1778	15.6554	18.9675	12.9475	16.5256	13.7125
hsa-miR-1225-3p	31.5944	32.5100	32.0029	33.7185	0.7652	0.8952	0.5446	0.4414		0	36.2679	34.6919	28.3085	32.2432	27.9130	32.1274	33.3216	33.6673	34.6528	39.9017	31.0208	30.4834
hsa-miR-424	14.9146	14.3859	13.8857	15.5781	0.7679	0.5719	0.7357	0.1298		0	12.3783	13.6611	15.4072	19.7180	16.0675	16.4981	12.2039	18.9990	12.8996	11.7163	15.3658	16.5608
hsa-miR-342-5p	4.3592	4.4429	4.5086	4.2864	0.7685	0.6033	0.8100	0.1205		1	4.2499	4.1475	4.0624	4.5132	4.5305	4.4563	5.0121	4.6227	4.6408	3.8022	4.3711	4.3945
hsa-miR-4284	42.9786	41.3372	40.9142	42.3453	0.7724	0.7176	0.9151	0.5515		1	43.1927	42.2979	40.3857	35.8838	36.0043	47.4032	37.6373	38.4108	48.1938	49.3479	41.4124	42.0700
hsa-miR-933	11.2789	11.6575	11.6723	11.6224	0.7729	0.7659	0.8118	0.9492		1	12.1555	9.3017	9.9398	10.5444	9.9935	12.6563	11.1799	12.2684	13.5566	14.7636	11.8571	11.2502
hsa-miR-1267	9.9853	9.7328	9.7368	9.7231	0.7785	0.7842	0.7965	0.9825		1	10.7057	8.1893	8.4101	8.6773	7.9018	10.6118	9.0703	8.7064	11.7930	11.5596	9.5937	11.6375
hsa-miR-625*	8.1823	8.3663	8.2676	8.6015	0.7797	0.8971	0.5798	0.4710		0	8.4545	7.5936	8.1171	9.1338	7.4305	8.8961	7.4958	8.1770	9.0972	10.0417	8.7802	8.1134
hsa-miR-151-5p	45.7818	44.3080	47.3876	36.9681	0.7854	0.7779	0.1434	0.0184		1	34.9929	29.3571	56.8270	58.4195	50.0297	37.2673	45.3267	38.5617	57.6715	29.2909	51.4913	38.8344
hsa-miR-765	67.9104	65.1913	64.0938	67.8072	0.7858	0.7056	0.9924	0.5056		0	69.4079	84.8208	72.1222	68.5426	60.9036	55.0053	65.8971	65.8467	49.1434	81.2209	65.5492	57.4270
hsa-miR-1275	313.9228	325.0201	308.7581	363.7778	0.7861	0.8999	0.2708	0.0199		0	312.0101	424.7788	338.6902	404.1117	317.6482	283.6652	294.2165	370.2572	245.0849	352.0321	343.5803	357.0313
hsa-miR-4298	228.4375	222.7883	215.2379	240.7836	0.7872	0.5340	0.6038	0.0825		0	251.5128	296.8345	215.3359	210.6850	199.6241	235.9740	209.9130	243.5549	188.1434	241.9368	220.1583	206.4641
hsa-miR-874	21.9794	21.4940	20.6300	23.5533	0.7897	0.4665	0.4412	0.0170		0	19.6270	21.9940	20.5539	25.4981	20.3100	21.4731	21.9982	24.5117	18.8073	24.5237	20.7491	21.9088
hsa-miR-145	8.2740	8.5431	8.8582	7.7920	0.7968	0.5802	0.6543	0.0046		1	8.2246	6.8784	10.8952	9.0150	9.4410	6.9404	8.3343	8.3435	9.0106	7.4528	8.8316	8.5955
hsa-miR-1238	32.4076	33.7610	33.5576	34.2457	0.8001	0.8312	0.7528	0.8273		0	38.1431	28.0338	27.0599	31.1790	26.4423	37.9111	33.8086	32.6788	39.9702	43.0787	30.3700	35.0501
hsa-miR-146b-5p	7.0904	7.2102	7.4432	6.6549	0.8043	0.4840	0.4332	0.0555		1	6.6743	5.3142	7.5549	8.7043	7.6651	6.8432	7.5980	6.8297	8.0429	5.6312	7.3919	7.4724
hsa-miR-4271	157.9746	154.0532	141.0337	185.0828	0.8089	0.3015	0.1748	0.0012		0	154.3622	209.4732	136.4850	170.3092	126.1156	166.7354	140.3885	192.3880	124.4575	199.2814	149.7877	170.7803
hsa-miR-1539	20.3605	21.1233	21.1086	21.1583	0.8100	0.8152	0.8179	0.9788		0	22.7667	19.5791	17.4752	19.3559	17.3750	22.3127	18.5184	19.7804	26.0949	26.1299	19.5797	21.9734
hsa-miR-196a	7.9804	8.2548	8.4418	7.8090	0.8129	0.6925	0.8837	0.0303		0	8.5801	8.0102	8.3546	8.1279	8.6300	8.6012	8.8079	7.6471	7.5325	6.9078	7.9265	7.6901
hsa-miR-3663-3p	712.7474	728.9678	696.1490	807.1858	0.8166	0.8131	0.2459	0.0271		1	816.1810	944.4941	639.4531	660.5145	648.2309	1054.8150	730.0393	719.7252	662.1578	676.2175	672.5908	725.9627
hsa-miR-185	109.7647	114.5523	119.4775	102.8140	0.8168	0.6452	0.7410	0.0577		0	106.0817	78.0982	125.8667	119.1383	130.8704	94.4719	119.2976	110.6588	126.2503	118.9670	136.3720	97.8959
hsa-miR-19a	221.7640	214.8304	224.8103	191.0447	0.8187	0.9212	0.3291	0.0141		0	200.4893	168.1456	229.9756	185.0261	234.7355	213.9607	225.4411	216.8897	238.1115	191.3378	242.1116	180.6457
hsa-miR-564	15.8769	15.2973	15.0841	15.8057	0.8213	0.7600	0.9785	0.4894		0	17.6577	20.7432	15.7207	19.3387	16.4142	16.6432	16.2523	13.7610	11.5374	12.3543	15.0103	13.5768
hsa-miR-93	133.2697	138.7247	143.9137	126.3576	0.8228	0.6659	0.7814	0.0493		0	126.8344	102.4891	140.1388	123.6969	153.0679	111.7743	145.4736	145.9087	153.9240	151.7841	162.2649	124.4066
hsa-let-7c	29.1604	30.2372	30.6750	29.1938	0.8236	0.7598	0.9947	0.5896		0	23.3648	21.6406	32.4632	40.5386	34.2690	29.0963	30.4339	32.4092	37.4590	24.6071	30.1809	30.3873
hsa-miR-3137	23.9193	23.5171	22.9359	24.9021	0.8254	0.5999	0.6220	0.1114		0	24.0739	26.7956	23.6481	24.8653	20.4870	22.8272	23.5991	24.1006	19.9588	25.4885	23.6762	24.6394
hsa-miR-4257	33.6140	34.2059	30.8210	42.2731	0.8270	0.2921	0.0398	0.0019		1	33.2637	36.0450	25.8115	34.7695	24.1725	43.2683	35.6146	49.7724	29.5451	52.8787	29.0224	37.2112
hsa-miR-4313	31.8245	30.4813	30.4755	30.4950	0.8285	0.8287	0.8403	0.9948		0	34.1995	24.5591	24.6232	27.2197	23.4760	36.3787	28.4297	28.5824	38.5301	38.0875	28.0177	32.1536
hsa-miR-769-3p	10.6974	10.4322	10.0530	11.3361	0.8290	0.6055	0.6358	0.0960		0	9.3872	12.9906	10.7765	14.0669	10.2445	9.1471	10.2649	11.9178	9.2545	9.3298	10.9561	11.8633
hsa-miR-191*	26.5156	27.5147	26.6126	29.6646	0.8296	0.9834	0.5360	0.2585		0	28.9620	26.7791	22.5797	27.0203	19.6865	32.2135	26.8308	27.8050	31.9987	37.6603	24.0285	30.5323
hsa-miR-744	6.4741	6.3261	6.6390	5.5803	0.8325	0.8198	0.2524	0.0433		0	5.5842	5.0278	8.2246	8.0299	6.9841	4.7143	6.6950	5.7020	6.5284	4.8480	7.0606	6.0784
hsa-miR-665	9.1970	9.0547	8.9116	9.3958	0.8357	0.6813	0.8043	0.3693		0	9.4317	8.6543	8.5335	10.2864	7.8700	9.0460	9.3515	9.5668	8.8958	9.2074	9.0737	9.7264
hsa-miR-654-3p	8.1236	8.3215	8.2092	8.5892	0.8382	0.9301	0.6573	0.4708		0	8.0434	11.0923	8.7798	8.7111	9.3910	8.8024	8.4097	8.3800	8.8090	7.4347	7.7535	7.4336
hsa-miR-320b	147.9446	151.0086	148.5240	156.9302	0.8425	0.9700	0.6097	0.4088		1	143.6546	110.6857	138.1042	170.4826	147.9399	161.0301	168.2049	192.4558	149.6583	180.6445	153.1035	139.5327
hsa-miR-223	3065.7043	########	3106.5730	2610.6875	0.8427	0.9402	0.4212	0.1037		1	2451.8669	2328.4850	3956.3633	3639.7549	3331.7883	2426.3027	2468.9584	2902.2554	3907.4450	1945.2696	2793.9040	2710.3164
hsa-miR-1915	2369.8681	########	2328.3656	2560.6895	0.8459	0.7691	0.2199	0.0069		2	2563.3416	2718.9218	2207.6623	2167.2552	2321.3509	2312.1937	2401.5386	2680.6027	2217.2171	2817.6307	2451.1979	2348.1390
hsa-miR-1226*	48.4240	47.3469	46.2424	49.9794	0.8481	0.6998	0.8102	0.3525		0	47.7542	58.2419	47.1083	60.5022	44.2340	38.1005	51.9733	47.3078	39.8685	50.3052	48.5572	42.1824
hsa-miR-3923	6.7593	6.9659	7.2272	6.3430	0.8504	0.6711	0.7080	0.0016		1	7.1176	6.6828	7.6517	6.9808	7.7723	6.7603	6.7267	6.8027	6.4395	5.0611	7.0381	6.0414
hsa-miR-2861	6530.1289	########	6703.2927	6360.0352	0.8614	0.6865	0.7343	0.4070		1	7799.1217	5758.6265	5881.9382	5086.5807	6480.1408	5652.8450	6189.6351	5582.6749	6238.4214	6383.4301	6897.7416	9380.1608
hsa-miR-3196	343.0726	348.1496	331.9558	386.7450	0.8625	0.7062	0.1871	0.0024		0	378.0087	433.8782	342.5033	356.6234	311.7166	352.4064	325.1267	381.6679	299.7665	403.7811	345.3152	369.2392
hsa-miR-1182	36.3491	37.1145	35.5504	40.8424	0.8638	0.8594	0.3637	0.0559		0	34.6834	46.5694	38.5568	49.4693	36.6932	33.4348	38.5456	43.9326	26.7790	40.8237	38.4387	31.9897
hsa-miR-3665	2729.6276	########	2708.0470	2662.4142	0.8679	0.9193	0.7657	0.6852		0	2871.5638	2533.8183	2540.4300	2331.5413	2553.0476	2851.2340	2752.3113	2612.8714	2692.5964	3079.2337	2636.4503	2555.4255
hsa-miR-520b	8.4661	8.6509	8.6996	8.5351	0.8681	0.8346	0.9514	0.6077		0	8.4966	8.7387	8.5138	9.4616	9.6067	8.8199	8.6278	8.9030	8.2174	7.9854	7.9245	8.1757
hsa-miR-3180-3p	8.5386	8.3997	8.0057	9.3389	0.8759	0.5541	0.4596	0.0840		0	6.5903	8.4907	9.0131	13.0650	7.5703	8.1672	8.3297	8.7323	8.0035	9.4893	7.9315	8.2781

hsa-miR-3131	12.2995	12.4873	11.7340	14.2827	0.8774	0.6376	0.3009	0.1401		0	10.9538	12.5036	11.0888	17.7487	9.9689	14.5527	11.8275	14.3919	12.5937	16.7053	12.6569	11.2143
hsa-miR-381	10.7036	10.4881	10.4493	10.5805	0.8820	0.8615	0.9362	0.8432		2	11.2162	9.0314	11.1012	8.9098	10.6541	10.8731	11.4891	12.9653	9.0577	10.6697	10.9387	8.2690
hsa-miR-663b	5.8729	5.7678	5.7302	5.8573	0.8872	0.8494	0.9842	0.7447		0	5.1642	6.4727	6.5374	7.8974	7.1851	5.4616	6.3803	5.9231	4.3215	4.5989	5.8072	4.8296
hsa-miR-140-3p	57.6435	58.9539	60.5745	55.0915	0.8883	0.7565	0.7904	0.1555		0	55.7966	37.3445	55.1384	54.4385	61.2197	52.3578	64.7495	63.7445	59.3181	66.7705	71.6752	56.5747
hsa-miR-28-5p	8.1136	8.0207	8.0632	7.9193	0.8945	0.9442	0.8102	0.8019		0	6.7160	6.9464	9.7785	12.0596	8.8577	7.7799	7.7214	8.0752	9.3218	6.5515	7.7107	8.1887
hsa-miR-1915*	8.1774	8.0836	8.0619	8.1353	0.8976	0.8759	0.9568	0.8572		2	8.5861	8.1529	8.7737	9.4067	8.2307	8.2476	7.6828	8.3476	7.6252	6.8630	8.0517	7.7818
hsa-miR-1972	15.3733	15.1737	14.7587	16.1627	0.8996	0.7014	0.6527	0.1660		0	14.8062	18.8205	16.9037	17.6084	14.2222	13.6986	13.2528	16.2694	12.3012	15.0795	16.6481	14.6236
hsa-miR-338-3p	11.1268	11.3031	11.5038	10.8247	0.9038	0.7972	0.8460	0.3411		1	9.4575	8.0850	11.8907	13.7284	12.4750	10.6057	11.6635	12.3010	11.6058	9.1708	12.4557	12.0383
hsa-miR-3934	14.1575	13.9868	13.7024	14.6645	0.9063	0.7569	0.7568	0.3395		0	12.6163	14.1176	13.9396	16.3603	12.9660	11.8704	14.8203	15.3566	11.7346	15.5331	14.9013	13.9193
hsa-miR-17*	4.7711	4.8272	4.9209	4.6039	0.9071	0.7605	0.7441	0.2638		0	3.7805	3.3067	5.1757	5.6695	5.4778	4.3323	5.0174	5.1030	4.6043	4.3164	5.4795	4.2392
hsa-miR-451	13125.1092	########	12917.0618	13152.6050	0.9080	0.8706	0.9838	0.8326		0	10907.5778	12311.7630	14560.8888	12786.9889	14137.4598	14840.3797	12000.9293	13539.1985	12663.9220	13013.2351	12472.8830	12016.6336
hsa-miR-422a	7.5586	7.6479	7.8080	7.2662	0.9112	0.7593	0.7446	0.3306		1	6.6991	6.2880	8.1191	9.0699	7.5759	5.3219	8.6162	6.8710	7.5230	8.2027	7.3015	7.0522
hsa-miR-192	12.5961	12.7911	12.6212	13.1960	0.9140	0.9890	0.7515	0.5105		0	12.6173	10.6242	12.5630	18.7923	14.6249	13.1042	11.8042	13.8203	11.8074	12.8796	11.5527	12.2497
hsa-let-7d	65.8233	64.7420	67.8277	57.3877	0.9155	0.8500	0.4422	0.1471		1	44.4148	56.8590	80.5736	90.5871	75.2931	54.9649	62.9947	58.6709	86.5792	46.4191	69.5847	57.2543
hsa-miR-3074	5.8957	5.8183	5.8588	5.7216	0.9198	0.9619	0.8299	0.6796		2	6.0728	6.0373	5.7139	6.4367	6.5430	6.4819	5.9683	5.6353	5.4537	4.9302	5.8087	4.3584
hsa-miR-135a*	92.8948	93.9316	89.7075	103.9992	0.9206	0.7601	0.3494	0.0372		0	89.8478	111.3594	95.1957	118.9712	90.2888	94.9937	100.7694	113.6864	82.5847	97.6113	92.0023	85.0332
hsa-miR-19b	751.7980	739.2795	783.3735	634.1888	0.9210	0.8080	0.3619	0.0134		0	659.3677	481.2172	678.5581	658.1562	837.2647	626.8925	705.6049	709.5211	923.4540	652.3950	986.1075	650.6413
hsa-miR-198	39.3148	38.6689	35.0011	47.4108	0.9247	0.5321	0.3076	0.0116		1	31.9455	58.1626	39.6989	73.0969	36.7243	34.7046	38.3809	44.6239	26.3399	41.8760	40.5960	41.9520
hsa-miR-224	5.1925	5.2337	5.0719	5.6195	0.9286	0.7958	0.4362	0.1506		0	4.4322	4.6723	6.2705	6.2736	5.0855	5.2295	4.9998	6.0364	5.0838	5.1820	4.7274	6.2245
hsa-miR-320a	106.9064	105.8722	102.6095	113.6482	0.9291	0.7113	0.6176	0.1706		0	94.6990	81.9679	97.5639	124.3813	102.3705	112.7984	114.6162	136.8854	108.5697	129.5499	99.4177	98.6475
hsa-miR-758	4.9345	4.8985	4.9687	4.7310	0.9293	0.9332	0.6339	0.1915		0	4.7650	5.2454	4.8777	4.5043	5.3539	4.6826	4.8359	4.3905	5.6607	4.8678	4.9180	4.4346
hsa-miR-3180-5p	12.7576	12.5841	12.4372	12.9342	0.9294	0.8706	0.9332	0.6129		0	12.5918	10.2461	11.1176	12.0913	10.0214	12.3260	12.6693	13.6259	14.1384	16.3305	12.3377	13.7180
hsa-miR-16	2419.1261	########	2382.8573	2318.4220	0.9321	0.9560	0.8803	0.8151		0	1960.3286	1570.9085	1949.5937	3004.5876	2606.4023	2593.1488	2818.1954	3001.5641	2677.9516	1949.9735	2284.9205	2273.9512
hsa-miR-20a	317.1065	322.6633	334.7906	293.7598	0.9324	0.7901	0.7320	0.1764		0	264.6928	250.2271	348.4095	380.9187	377.5924	294.2238	322.0886	311.3040	394.6621	263.5912	332.0417	298.9338
hsa-miR-188-5p	102.5095	101.5742	96.0043	114.8490	0.9372	0.5866	0.3752	0.0330		0	103.5468	122.7309	89.9009	108.0248	82.0735	108.3036	101.7903	105.0716	85.5651	131.6918	99.8289	112.6873
hsa-miR-34a	7.1936	7.2424	7.1395	7.4877	0.9386	0.9318	0.6877	0.4190		0	7.1395	6.6585	6.8823	7.5695	7.1746	10.4339	7.2449	6.8041	7.6083	7.0223	6.6680	7.4115
hsa-miR-374b	22.4626	22.2583	22.9701	20.5619	0.9494	0.8778	0.5843	0.2357		0	16.9760	18.0110	28.2021	32.0575	24.9645	18.7023	22.1686	20.9377	28.3137	17.5359	24.7191	21.3382
hsa-miR-4270	584.3460	580.2463	549.7419	652.9486	0.9522	0.6169	0.3762	0.0239		0	540.6099	789.5225	619.1428	769.8761	562.3109	650.8894	527.9647	613.6135	463.5173	564.9819	621.4195	597.2702
hsa-miR-324-3p	46.9677	46.8353	46.5792	47.4457	0.9539	0.8674	0.8471	0.5275		1	47.6670	47.3922	45.9372	39.1857	44.9637	48.9116	49.7408	48.4398	49.8396	50.0969	46.2273	51.7090
hsa-miR-425*	23.0416	22.8774	22.5117	23.7490	0.9666	0.8934	0.8664	0.5429		0	24.8729	21.7649	19.2461	20.6018	16.8103	25.0454	22.4166	21.8079	27.5286	30.3818	21.2774	25.4759
hsa-miR-136	9.9120	9.8631	9.9945	9.5499	0.9671	0.9451	0.7772	0.4994		0	9.6293	7.8583	9.1216	8.1989	9.3238	10.2046	11.3760	10.9466	10.8607	9.8850	10.9652	8.5966
hsa-let-7a	188.9140	187.7716	191.8851	177.9679	0.9732	0.9325	0.7655	0.5536		0	126.8428	189.4448	224.5448	270.7639	229.5979	186.0119	191.6104	176.9901	241.8145	136.2264	188.5555	174.1980
hsa-miR-574-5p	80.2570	79.8713	77.1849	86.2737	0.9737	0.7936	0.6404	0.1670		0	75.7918	90.7564	66.0452	103.8798	69.5289	89.6180	87.0130	90.1335	82.4165	73.6625	80.3146	71.0196
hsa-miR-638	4795.8979	########	4841.3238	4718.2646	0.9740	0.8722	0.7928	0.4192		0	5321.8926	4858.1776	4605.0317	3957.9173	4647.6879	4494.1297	4825.6041	4394.3240	4781.8827	5083.2967	4630.7215	5249.7058
hsa-miR-3676	21.2460	21.3317	20.8983	22.3647	0.9744	0.8970	0.7090	0.3948		0	21.2878	19.9299	18.8431	22.2087	16.4504	22.9348	20.5416	21.0652	24.2000	27.9768	20.4070	22.9961
hsa-miR-320e	150.5304	150.9046	152.3691	147.4140	0.9757	0.8819	0.8218	0.5379		1	153.9300	117.8396	140.5228	142.9405	159.5285	152.3879	171.7854	180.1715	145.6920	159.0375	151.4358	136.4604
hsa-miR-183	5.3974	5.4179	5.1435	6.0718	0.9759	0.7110	0.3553	0.0130		0	4.5271	5.5414	4.8905	7.5436	5.9839	5.5677	5.0138	6.8995	5.2493	5.7207	5.0642	5.6796
hsa-miR-196b	8.8636	8.8302	9.0059	8.4113	0.9789	0.9108	0.7249	0.0946		1	8.9980	9.4867	8.4901	9.5042	9.4990	8.4674	9.1349	8.3225	8.9235	7.3515	8.6435	7.6329
hsa-let-7b	237.0610	235.9980	232.6806	243.9043	0.9795	0.9172	0.8737	0.5866		0	185.9336	221.1842	225.7592	332.1819	250.7065	259.8851	217.5311	245.8917	290.1115	193.4605	263.0737	251.2507
hsa-miR-3202	27.3938	27.4882	25.3932	32.4811	0.9809	0.6160	0.2804	0.0310		0	22.3806	40.6084	30.1585	43.0886	23.2674	26.6152	24.9852	30.4937	21.1576	34.2238	29.2663	25.7371
hsa-miR-17	157.7628	156.9892	166.3385	134.7066	0.9812	0.7968	0.4950	0.0126		0	148.1599	133.2121	185.1111	152.8368	177.1069	121.1267	160.2516	139.4294	192.1880	130.0320	176.5164	142.4978
hsa-miR-139-3p	16.0938	16.1319	16.5257	15.1932	0.9820	0.8006	0.6156	0.1291		1	16.1724	15.3897	17.2041	19.1274	17.9939	12.8175	18.4930	17.8577	14.7975	14.5699	16.4629	12.7146
hsa-miR-15b	403.7225	404.8242	419.4960	369.8565	0.9847	0.7944	0.5778	0.2322		1	299.9671	346.4772	425.6834	491.8103	490.0717	376.6027	355.2219	428.3842	543.6159	278.4322	473.4460	368.6562
hsa-miR-718	226.2929	226.7021	215.8691	252.5207	0.9866	0.6711	0.3250	0.0081		1	256.6587	311.1931	206.7903	210.1125	213.0941	250.1234	221.3443	250.2423	183.6058	232.7223	234.9008	248.6355
hsa-miR-154	6.7994	6.8100	6.7464	6.9615	0.9882	0.9416	0.8296	0.4831		0	6.3108	7.4765	7.1682	6.6541	6.8158	7.3618	7.5521	6.8002	7.1131	6.6779	6.4117	6.7637
hsa-miR-142-3p	212.2445	211.8047	227.8952	173.4558	0.9902	0.6730	0.3010	0.0064		1	165.8504	131.6138	207.6772	192.5747	235.7501	178.6045	254.8875	164.0561	264.5182	143.6601	247.1527	209.0647

X	Y	Z	AA	AB	AC	AD	AE	AF	AG	AH	AI
				p-values for the comparison metastases versus no metastases							
mean TP7 no metastases	mean TP7 metastases	mean TP8 no metastases	mean TP8 metastases	TP 1 metastases vs no metastases p-value	TP 2 meastases vs no metastases p-value	TP 3 metastases vs no metastases p-value	TP 4 metastases vs no metastases p-value	TP 5 metastases vs no metastases p-value	TP 6 metastases vs no metastases p-value	TP 7 metastases vs no metastases p-value	TP 8 metastases vs no metastases p-value
5.0621	3.9887	6.1880	3.7643	0.0202	0.0052	0.4675	0.1559	0.9662	0.1820	0.0234	0.0698
301.5122	369.1086	306.1181	483.1503	0.5186	0.3895	0.4624	0.2741	0.9175	0.6781	0.3716	0.0911
6.0391	6.1557	6.2306	7.8962	0.4532	0.6873	0.8378	0.7019	0.5020	0.4878	0.9498	0.4136
13.4109	9.9723	11.5587	11.6359	0.9060	0.9246	0.4914	0.7248	0.7554	0.4133	0.0657	0.9599
11.3189	9.6979	10.7755	14.1131	0.2665	0.7481	0.5044	0.8710	0.0963	0.3891	0.1748	0.1003
6.4372	7.1949	6.8388	9.8103	0.4108	0.5798	0.9870	0.7389	0.3658	0.8175	0.6495	0.2546
17.7805	14.6604	17.1361	13.4280	0.5175	0.5292	0.7021	0.7209	0.7171	0.1840	0.3204	0.1512
4.8440	5.9857	4.4144	5.0147	0.7340	0.6336	0.0319	0.2710	0.0709	0.7354	0.1712	0.1202
42.0017	39.3571	41.5722	38.1126	0.4446	0.9130	0.7222	0.9517	0.2652	0.5437	0.5629	0.5647
21.2667	22.8048	21.1207	17.7522	0.1995	0.5932	0.4073	0.5520	0.2830	0.9252	0.7008	0.3721
11.9057	10.2698	11.6505	8.9981	0.5365	0.3852	0.7529	0.7567	0.5454	0.3195	0.2459	0.2148
16.8207	26.6479	14.6337	16.2086	0.6223	0.4245	0.0446	0.9101	0.8292	0.2417	0.2771	0.5207
7.8505	8.4719	8.2841	10.4806	0.1661	0.0773	0.3114	0.1540	0.3368	0.5965	0.6740	0.4437
6.3956	5.5283	5.9448	5.6333	0.4054	0.7901	0.2333	0.7452	0.6424	0.1733	0.3620	0.7028
9.5688	13.5246	10.4826	11.9840	0.7972	0.5432	0.0908	0.3559	0.1114	0.5047	0.0508	0.2365
6.3583	7.8469	6.4932	7.2144	0.4289	0.4370	0.0137	0.5224	0.0330	0.3271	0.3183	0.4944
6.7950	7.3733	7.3547	6.6620	0.7008	0.5898	0.1808	0.5182	0.7239	0.4425	0.5104	0.3120
12.3253	22.6976	12.5860	17.1028	0.5361	0.4922	0.0342	0.5075	0.0190	0.6653	0.0338	0.1985
170.1384	172.2513	173.6107	183.2875	0.6322	0.3153	0.5300	0.7115	0.5534	0.3818	0.9631	0.8584
12.7982	13.1553	11.6671	12.0007	0.6147	0.4678	0.2685	0.7786	0.2950	0.4319	0.8688	0.8026
8.6677	11.1602	8.9849	8.6017	0.4218	0.1960	0.0504	0.1601	0.9338	0.6067	0.0939	0.7183
8.7832	6.4052	8.2735	7.7199	0.1349	0.3976	0.8249	0.7802	0.6004	0.3216	0.0259	0.7151
5.7044	5.1940	5.6135	5.2167	0.7737	0.0731	0.6297	0.6652	0.7192	0.0916	0.4832	0.5894
25.9981	23.8018	27.7776	25.5179	0.4111	0.6843	0.4897	0.2132	0.9780	0.0174	0.5149	0.4386
7.2254	8.1454	7.1177	7.3074	0.7508	0.7845	0.1875	0.9604	0.5946	0.6906	0.3553	0.8829
32.2917	30.9514	32.3609	31.2064	0.6411	0.5265	0.3993	0.9870	0.2848	0.8046	0.8410	0.8776
5.2708	6.4471	5.2979	5.4252	0.6761	0.4977	0.0261	0.0239	0.3720	0.4364	0.1362	0.7403
6.1435	6.9860	7.7181	7.1932	0.8717	0.1167	0.0735	0.7332	0.1458	0.0198	0.4138	0.6907
17.2012	16.3956	15.3076	14.4993	0.6599	0.6369	0.3262	0.9701	0.8140	0.6378	0.7871	0.6995
22.2368	19.2637	26.0685	28.1328	0.9358	0.3129	0.1355	0.3101	0.7105	0.2437	0.2863	0.5768
35.8832	38.8694	43.4120	37.6033	0.6747	0.3804	0.9912	0.9167	0.1832	0.2210	0.5859	0.3379
5.0735	5.8272	5.4453	5.2150	0.7092	0.5775	0.1512	0.9702	0.3012	0.8856	0.1323	0.6343
4.2831	4.7417	3.8102	4.6411	0.4483	0.7076	0.0463	0.2526	0.3956	0.9606	0.3378	0.1088
5.7590	6.5389	6.5356	7.5748	0.6201	0.2478	0.9333	0.2716	0.1697	0.6682	0.3551	0.2247
14.6677	13.2829	14.5237	12.9534	0.9804	0.9051	0.0649	0.9503	0.1272	0.5733	0.5207	0.5561
46.5654	31.9036	37.6844	39.5210	0.8614	0.8612	0.3256	0.9052	0.9498	0.9624	0.1002	0.8762
35.3166	37.6826	36.4286	36.4369	0.4311	0.5025	0.1040	0.6151	0.0734	0.3300	0.7283	0.9981
7.9315	6.5616	7.8146	7.3240	0.4236	0.7732	0.5999	0.7488	0.9868	0.7988	0.1410	0.6407
225.2534	179.2131	224.5476	181.3371	0.5945	0.0429	0.2935	0.2032	0.2721	0.5861	0.1825	0.2100
17.9662	15.1269	14.7962	14.9191	0.4060	0.5226	0.0632	0.2945	0.1292	0.4741	0.2686	0.9608
5.6857	6.3406	5.7043	6.2676	0.5833	0.5270	0.0831	0.4170	0.1871	0.8223	0.4732	0.6207
116.1396	107.6654	114.9954	97.1054	0.3107	0.4901	0.3126	0.8293	0.3622	0.3718	0.7437	0.4476
4.2448	4.3584	4.3082	3.9206	0.1347	0.9509	0.3027	0.7965	0.2457	0.8420	0.8712	0.5684
5.8826	6.5349	6.6525	7.0788	0.9882	0.7515	0.7722	0.2760	0.1944	0.7179	0.1232	0.3673
3.5211	3.9867	3.6457	3.9327	0.3838	0.3322	0.0383	0.6387	0.5289	0.6369	0.3573	0.5351
9.9862	11.6578	11.2513	10.4489	0.1271	0.3046	0.7610	0.8353	0.2362	0.8961	0.3453	0.6362
4.6862	5.6881	5.0501	5.4607	0.3274	0.9699	0.4923	0.9656	0.1124	0.6350	0.2574	0.6772
37.0479	25.9792	30.3611	31.0495	0.9936	0.8768	0.5051	0.8952	0.8806	0.9275	0.1329	0.9395
5.2546	5.1440	5.5909	6.1781	0.9758	0.5798	0.1426	0.1397	0.5589	0.8516	0.8307	0.6054
47.7249	59.5802	57.9916	55.0365	0.4876	0.3643	0.4622	0.6852	0.8400	0.5748	0.2947	0.7513
13.4090	18.6718	12.5554	12.7364	0.5518	0.0458	0.3153	0.3585	0.0682	0.1297	0.4289	0.9206
7.8886	7.5270	8.9876	8.2273	0.7967	0.4359	0.8924	0.5194	0.2927	0.3823	0.7145	0.5484
12.6039	10.9224	11.1295	10.6254	0.1663	0.9226	0.1992	0.9063	0.8965	0.8369	0.4388	0.8311
7.2169	7.0170	7.3643	7.2374	0.9411	0.6442	0.5004	0.2286	0.5539	0.5339	0.5556	0.8868
3.8339	4.4376	3.9939	4.2831	0.5470	0.3664	0.4029	0.6999	0.6054	0.8330	0.4134	0.6341

4.1312	4.4495	4.6138	4.0776	0.4660	0.0982	0.1143	0.2452	0.5049	0.9011	0.5102	0.2231
361.4219	410.4305	315.2237	310.1296	0.8498	0.7807	0.9002	0.8330	0.6189	0.5355	0.5427	0.9359
6343.6249	8405.7315	5915.6793	4522.6983	0.4665	0.4494	0.5198	0.7796	0.3465	0.0703	0.5427	0.1362
10.3339	10.4469	10.2013	9.8419	0.5864	0.6577	0.2199	0.9887	0.4441	0.7163	0.9354	0.7382
274.4747	312.3535	228.6704	226.3419	0.4833	0.5348	0.7979	0.8182	0.3887	0.9487	0.5338	0.9638
9.2896	5.8737	8.6834	6.8680	0.6952	0.7461	0.6307	0.7352	0.9143	0.8523	0.0004	0.0513
9.4083	12.5739	9.3499	11.2638	0.7709	0.5503	0.0106	0.2946	0.1291	0.3545	0.2440	0.3924
7.5963	6.9940	7.9014	8.4405	0.6837	0.0114	0.2096	0.4851	0.2177	0.7987	0.3867	0.5176
3.0691	2.4661	3.0992	2.0765	0.8661	0.9622	0.9296	0.6941	0.5527	0.2269	0.1571	0.0382
15.3556	13.3029	15.2115	12.6610	0.9347	0.6499	0.1331	0.4462	0.5896	0.1939	0.4169	0.2082
50.8982	67.6966	55.2715	59.5338	0.1576	0.3651	0.0984	0.5373	0.2041	0.5518	0.2104	0.7119
5.4126	5.9284	5.6796	5.7935	0.9494	0.5880	0.1079	0.9710	0.5653	0.7979	0.3200	0.8039
90.0991	87.4117	93.5623	67.7714	0.8017	0.9925	0.4199	0.8996	0.0640	0.8454	0.9279	0.2775
10.6373	11.6986	12.5962	9.7948	0.0871	0.0392	0.4087	0.9672	0.2303	0.0358	0.5855	0.1889
7.2592	9.1200	9.4091	11.7021	0.9102	0.0330	0.3793	0.0129	0.2287	0.2389	0.1039	0.0491
4.4232	4.6637	4.6007	4.7499	0.8481	0.9587	0.5041	0.6747	0.7083	0.1529	0.6261	0.7909
12.7792	15.6436	11.8582	11.7593	0.3568	0.4892	0.9673	0.7852	0.1236	0.3449	0.5051	0.9828
17.6129	21.4538	18.6794	18.3166	0.7543	0.2933	0.1016	0.6547	0.0240	0.3377	0.2168	0.8992
8.3745	8.6219	8.6961	9.8025	0.6703	0.0647	0.2836	0.7219	0.4061	0.3425	0.8722	0.5452
5.9910	6.3477	5.7107	5.1290	0.8148	0.9372	0.1856	0.8113	0.2630	0.2608	0.6760	0.3069
8.8908	10.8304	8.6713	9.7885	0.1880	0.3529	0.0133	0.1662	0.0784	0.5681	0.2509	0.4462
41.1343	40.2201	44.6697	30.1998	0.1384	0.1918	0.0564	0.1278	0.2005	0.1876	0.9021	0.0346
6.0758	7.3319	6.3362	7.1126	0.4708	0.2127	0.4053	0.6547	0.1852	0.3232	0.4102	0.6501
2.9313	3.7242	3.1406	3.2274	0.0065	0.6209	0.2452	0.2781	0.2392	0.7964	0.0879	0.8367
20.1698	23.0410	20.9533	21.2241	0.1145	0.2852	0.2108	0.6572	0.4053	0.6346	0.4551	0.9308
6.1893	7.3513	7.1420	7.4984	0.7219	0.7121	0.8082	0.9193	0.0940	0.6002	0.2520	0.7850
14.0542	14.7844	15.2983	18.4380	0.6377	0.2273	0.8169	0.3543	0.0398	0.6769	0.7586	0.1409
13.1549	12.5327	13.4876	17.5124	0.9026	0.0722	0.3642	0.4010	0.2941	0.6254	0.8687	0.5966
14.9808	16.3828	17.1373	14.4744	0.5311	0.2484	0.4336	0.4162	0.2561	0.8289	0.5482	0.2618
5.2949	6.8489	6.6069	6.2738	0.5732	0.6117	0.8968	0.0687	0.1833	0.9384	0.0678	0.6786
12.9023	9.8245	11.8046	10.7380	0.8912	0.8469	0.8788	0.8249	0.9212	0.6946	0.0721	0.6068
7.1095	7.6555	7.1630	7.4712	0.2308	0.8906	0.0944	0.6717	0.1354	0.7573	0.6618	0.8047
7.7192	8.0953	7.9868	7.6792	0.0840	0.4097	0.3286	0.1536	0.5014	0.9562	0.7268	0.7834
4.7536	4.8647	4.9800	5.2094	0.6556	0.7757	0.3316	0.2814	0.3705	0.3345	0.7804	0.6415
5.5578	5.8904	5.8034	5.5568	0.6807	0.7952	0.6251	0.5091	0.4326	0.8340	0.7006	0.8055
6.1008	6.3298	6.9116	6.8456	0.3412	0.1561	0.0423	0.2577	0.5911	0.7547	0.7785	0.9704
19.1423	21.4910	17.9033	16.3108	0.2076	0.5806	0.6187	0.6332	0.0677	0.3742	0.7136	0.8145
5.3466	7.0043	6.1468	7.9707	0.2814	0.1778	0.4719	0.0921	0.1124	0.2946	0.2052	0.2780
38.3279	41.8233	34.7391	27.9219	0.3019	0.5633	0.7278	0.3138	0.7558	0.9754	0.7205	0.4109
20.7407	19.6852	20.0271	16.6245	0.4293	0.8698	0.3182	0.7833	0.1959	0.7715	0.7913	0.4191
16.8551	19.9868	17.0323	19.0195	0.2592	0.3867	0.7140	0.8324	0.1277	0.4125	0.3316	0.5201
6.6074	8.0392	7.1588	5.6493	0.2627	0.2869	0.9829	0.2506	0.1435	0.7645	0.1119	0.1759
8.3264	9.6206	8.8842	7.7786	0.4247	0.4659	0.9830	0.5000	0.0284	0.1848	0.4488	0.6568
69.5729	59.6449	67.7760	54.5416	0.5551	0.0186	0.0027	0.2118	0.0077	0.1093	0.1712	0.1448
643.5258	631.6992	747.4878	764.1901	0.0916	0.8168	0.1944	0.8877	0.4827	0.4915	0.9407	0.9323
120.7315	166.9952	120.7927	169.1220	0.3523	0.6401	0.2571	0.5965	0.2138	0.6025	0.3657	0.1512
6.3173	6.8868	5.9797	6.6085	0.7405	0.4005	0.3972	0.7283	0.5954	0.4552	0.7009	0.7038
6.6604	6.5104	6.0560	5.0393	0.5790	0.8180	0.8792	0.2629	0.1733	0.8641	0.9105	0.1374
4.6904	5.1538	4.8448	4.6934	0.4279	0.8564	0.9096	0.9083	0.1479	0.7854	0.5708	0.8801
9.7450	8.0106	8.4391	8.7450	0.2620	0.7715	0.3447	0.8260	0.9806	0.6318	0.1190	0.8169
7.6199	6.9903	8.6852	6.6675	0.0151	0.0073	0.3926	0.3961	0.3250	0.1417	0.5284	0.1296
6.3113	6.1703	7.1881	8.7976	0.1826	0.0048	0.0744	0.0078	0.5594	0.1384	0.7569	0.2090
15.7251	13.8219	15.7997	14.0047	0.6034	0.4024	0.0187	0.9199	0.0190	0.6512	0.4907	0.5070
29.1725	20.9850	26.4180	22.6375	0.6460	0.5650	0.5521	0.0565	0.2200	0.0292	0.0039	0.3356
50.0913	57.1756	59.2257	41.7820	0.4285	0.2850	0.6835	0.7313	0.3142	0.5722	0.6354	0.0092
21.4081	20.5310	18.3214	28.0726	0.3826	0.1592	0.6996	0.4139	0.1447	0.4748	0.8558	0.0323
5.6214	5.2020	6.2884	4.9538	0.1475	0.5700	0.8518	0.0115	0.2008	0.2696	0.2496	0.0481
42.0981	38.2823	42.6186	34.1557	0.3305	0.8629	0.4998	0.7194	0.1286	0.8017	0.6244	0.2875
3.7617	3.5442	3.6514	3.0187	0.8112	0.6468	0.0417	0.4776	0.1238	0.2772	0.6153	0.2320
6.8762	5.8694	6.6074	7.9734	0.7168	0.9788	0.9990	0.8491	0.8603	0.6845	0.1168	0.4561
151.1283	150.4336	152.0516	144.5471	0.0293	0.3487	0.1853	0.6123	0.8730	0.1149	0.9843	0.8063
8.8235	9.8266	8.9551	8.6024	0.8708	0.3743	0.4905	0.7791	0.4234	0.3996	0.5353	0.7609
358.4253	341.3030	364.3430	443.1348	0.9205	0.7414	0.3757	0.1532	0.1784	0.3242	0.8005	0.2350
531.5746	666.4103	551.5306	511.0172	0.2204	0.7854	0.4729	0.5292	0.3121	0.9156	0.2289	0.6204

20.1501	22.0621	25.6138	28.3697	0.4763	0.8192	0.8057	0.0354	0.3325	0.3641	0.5414	0.5229
70.8960	91.8799	90.3098	76.3922	0.9201	0.3604	0.4986	0.4045	0.1776	0.3134	0.6161	0.4598
11.3425	11.0273	13.5031	11.6501	0.1965	0.7538	0.1323	0.6424	0.5445	0.2390	0.8705	0.4351
5.0390	5.6684	5.2718	4.0791	0.9725	0.6190	0.3359	0.2074	0.9825	0.9049	0.2301	0.0564
13.1808	14.3191	11.1277	11.8908	0.4026	0.8223	0.8389	0.8479	0.3257	0.2120	0.7080	0.7944
5.9522	6.4960	6.2301	5.5561	0.5520	0.7062	0.4326	0.3885	0.2066	0.3433	0.4846	0.4997
9.7626	9.4498	9.2387	11.0987	0.1291	0.0566	0.6499	0.0983	0.6257	0.8887	0.8767	0.3150
6.2398	5.5099	5.7564	5.8318	0.9816	0.0783	0.8975	0.1283	0.2967	0.0534	0.0568	0.8100
3.7237	3.9082	3.9219	3.3628	0.2086	0.6190	0.6382	0.4359	0.0699	0.7321	0.5516	0.1318
4.3722	3.3594	4.4556	3.4119	0.2763	0.2913	0.2916	0.6425	0.5791	0.7920	0.0030	0.0220
4.6302	4.7211	4.8896	3.6798	0.1972	0.8159	0.5864	0.2922	0.1385	0.2665	0.9137	0.1085
11.2723	11.4220	10.4733	9.1309	0.8974	0.3796	0.2678	0.2553	0.4069	0.8434	0.9486	0.5673
7.0866	7.3426	7.4008	5.3228	0.9058	0.1103	0.8967	0.7191	0.0984	0.5150	0.7549	0.0035
4.7450	5.7206	4.9758	4.6728	0.6564	0.4546	0.3067	0.1515	0.4514	0.4535	0.0261	0.5559
7.0160	6.8571	6.6905	5.7448	0.9060	0.2567	0.0730	0.5402	0.2972	0.7010	0.7804	0.0643
9.0969	8.2580	8.9163	7.4419	0.4002	0.3617	0.5146	0.4110	0.3831	0.4497	0.3815	0.0684
13.3424	11.1339	13.5963	9.7998	0.1531	0.3747	0.7286	0.4223	0.1664	0.6965	0.1346	0.0249
8.5139	10.3630	9.2846	10.8161	0.5440	0.6239	0.1593	0.1753	0.1427	0.6838	0.2281	0.3437
32.3309	33.1966	41.4963	35.2108	0.1298	0.6229	0.9285	0.2695	0.3115	0.6678	0.8235	0.1040
7.0704	7.2223	6.9466	5.6505	0.0531	0.6981	0.1335	0.6692	0.0530	0.6899	0.9227	0.0677
84.4190	91.0420	84.9612	77.2870	0.0400	0.9410	0.2373	0.2249	0.8587	0.2506	0.4899	0.4824
22.0919	28.5759	20.9711	26.5608	0.4719	0.6255	0.3835	0.2490	0.0446	0.9692	0.1647	0.2711
11.1108	12.7375	10.2769	9.3742	0.2599	0.5015	0.8554	0.6554	0.1033	0.2384	0.6049	0.7883
54.9870	74.6825	63.7923	67.5324	0.2242	0.6256	0.1639	0.5524	0.1734	0.2742	0.2124	0.7604
7.5146	9.1258	6.8431	8.0137	0.2936	0.4908	0.5615	0.5318	0.9346	0.3841	0.3681	0.6341
21.3291	20.6320	25.5630	21.5542	0.8028	0.0850	0.0122	0.0029	0.3163	0.0499	0.8782	0.4970
10.6032	11.9862	11.3335	10.1824	0.1290	0.6486	0.2561	0.3789	0.4460	0.4964	0.3506	0.4322
13.8928	14.1013	13.7923	12.4920	0.2214	0.0558	0.4855	0.2681	0.6268	0.0608	0.9303	0.4781
7.8767	8.2355	7.4297	7.7970	0.2626	0.7406	0.4313	0.0089	0.4700	0.3869	0.7523	0.8232
9.7162	9.1942	10.2078	9.0921	0.1282	0.4345	0.1153	0.8746	0.1227	0.7553	0.7379	0.5459
6.8853	6.8134	7.3833	7.8834	0.4634	0.8078	0.0221	0.5288	0.2091	0.1073	0.9285	0.5924
2.9694	3.3602	3.2567	2.9933	0.3717	0.9960	0.4615	0.3489	0.3148	0.9397	0.3252	0.6418
15.7899	12.5759	14.9596	14.5068	0.2262	0.9222	0.4388	0.6389	0.6497	0.8795	0.1601	0.8709
6.7170	5.9488	6.7554	6.8662	0.3920	0.1265	0.8062	0.4143	0.2977	0.5640	0.1316	0.9034
4.3698	4.2102	4.2812	4.1362	0.7461	0.3833	0.3097	0.4712	0.0989	0.2387	0.8261	0.8657
4.8731	5.3190	5.0069	5.0033	0.0775	0.5425	0.3208	0.5741	0.8111	0.7136	0.3855	0.9938
20.9023	16.5200	18.9582	20.5816	0.9088	0.8244	0.4787	0.6724	0.7363	0.4041	0.2655	0.7057
4.3548	4.8892	4.3353	5.2167	0.0733	0.3860	0.6628	0.1804	0.4919	0.9974	0.6064	0.4907
4.9818	5.2336	5.2668	4.6142	0.2988	0.7229	0.3253	0.9017	0.5402	0.3622	0.5409	0.0906
59.4227	94.9538	66.3166	48.9184	0.5973	0.7110	0.3835	0.5497	0.6428	0.0456	0.4228	0.2095
750.8775	728.3929	692.2947	818.8872	0.2496	0.3546	0.3119	0.6382	0.1196	0.6643	0.8326	0.3470
7.3963	6.4300	7.2341	7.4621	0.8187	0.8515	0.8787	0.5801	0.4471	0.6278	0.2112	0.8248
804.0843	501.7764	731.3327	565.6264	0.2135	0.0229	0.2777	0.2540	0.0496	0.4984	0.0158	0.4107
3.8266	4.5694	4.0623	3.6990	0.8135	0.5904	0.4878	0.6076	0.2168	0.2663	0.2199	0.6205
18.2067	15.4153	14.8655	14.4137	0.1267	0.7692	0.2200	0.5839	0.8822	0.5526	0.3716	0.9140
5.0883	5.2800	5.2285	4.7851	0.4299	0.6897	0.6203	0.5280	0.8583	0.9474	0.7723	0.3603
6.5464	5.9874	5.4541	6.1610	0.0339	0.5534	0.4263	0.4508	0.5180	0.4017	0.5038	0.6693
20.9037	21.5561	19.5590	21.4776	0.3518	0.1838	0.0869	0.7444	0.4917	0.4331	0.8777	0.6814
28.3123	24.4953	30.4918	28.5240	0.3693	0.2158	0.2276	0.7137	0.4219	0.8102	0.5136	0.8182
351.1578	348.6493	304.2306	242.2654	0.9492	0.8775	0.5090	0.7706	0.2218	0.7732	0.9768	0.3956
53.9993	40.1041	48.2425	50.2248	0.2478	0.9218	0.2553	0.7866	0.8510	0.2693	0.1915	0.8844
25.9979	19.1538	21.4937	22.3224	0.8588	0.7617	0.6256	0.9562	0.9387	0.6206	0.1711	0.9018
6.1821	5.7190	6.3098	5.4039	0.8414	0.0996	0.2984	0.8262	0.8879	0.5777	0.4539	0.4163
5.6662	5.9496	6.3508	6.3337	0.9431	0.2154	0.8493	0.0813	0.1501	0.3503	0.6141	0.9852
5.2682	5.5217	4.6313	4.9159	0.4737	0.6158	0.5091	0.8414	0.8219	0.3070	0.6841	0.6715
40.4248	44.5659	39.0903	43.1929	0.5372	0.5741	0.3069	0.9617	0.2847	0.6408	0.6436	0.6761
43.7355	38.0806	58.1180	52.2508	0.9543	0.1291	0.5652	0.2120	0.4803	0.3395	0.5221	0.6379
49.1516	65.4756	53.6917	60.1588	0.2253	0.5691	0.4112	0.6508	0.2273	0.8034	0.1465	0.5632
6.2588	6.9333	7.2166	6.9869	0.0847	0.0975	0.1223	0.2331	0.4927	0.6338	0.3075	0.7490
6.3967	6.4618	7.3566	5.8839	0.8554	0.2116	0.4840	0.4246	0.6472	0.2524	0.9381	0.1028
5.8849	8.0872	6.3012	7.1198	0.5025	0.4124	0.1830	0.0805	0.0017	0.6119	0.0236	0.3597
10.8202	8.2405	9.5923	8.5817	0.5899	0.8140	0.2621	0.8478	0.8628	0.8116	0.0872	0.4716
194.7191	195.3624	203.4923	170.9124	0.2598	0.4169	0.7227	0.4938	0.3012	0.7168	0.9864	0.5259
5.8646	5.7689	7.3440	5.1777	0.2005	0.7866	0.0169	0.6508	0.2286	0.0373	0.8885	0.0117

104.0163	74.3395	94.7552	74.4848	0.0305	0.3450	0.7073	0.3662	0.1130	0.4298	0.1984	0.3715
21.3427	15.7748	17.2734	20.1686	0.4970	0.8543	0.2125	0.9192	0.9412	0.7321	0.1498	0.5445
5.7030	6.1409	6.0550	5.2066	0.0182	0.0120	0.5988	0.5395	0.5404	0.0120	0.5839	0.2925
6.4377	6.9417	6.5109	5.4265	0.8491	0.4923	0.2090	0.8509	0.0140	0.8109	0.7811	0.2558
34.1149	36.6368	41.5590	52.8274	0.6539	0.8814	0.7797	0.0566	0.1034	0.3713	0.7233	0.1975
3.4441	3.8257	3.2097	3.1340	0.9869	0.4736	0.6678	0.9674	0.4751	0.4425	0.6297	0.9270
3.9914	4.0823	3.8157	4.3265	0.4000	0.6693	0.0092	0.8682	0.3706	0.7926	0.7700	0.2848
9.5159	10.1589	10.1284	9.3463	0.8118	0.5472	0.6722	0.2184	0.9416	0.5074	0.6175	0.5163
11.6790	11.6510	11.7810	11.1554	0.0082	0.1940	0.2753	0.4997	0.7175	0.9870	0.9830	0.6590
6.3303	6.9365	6.9890	6.7461	0.3143	0.2777	0.2865	0.2429	0.7368	0.3095	0.3845	0.7615
425.3454	562.8456	467.6891	413.5595	0.0615	0.4329	0.2147	0.3064	0.3896	0.8823	0.1140	0.4761
20.0612	20.4290	21.2325	21.5049	0.4829	0.8801	0.2031	0.2014	0.0606	0.5250	0.8706	0.9101
12.8853	9.9386	11.2408	9.0385	0.0694	0.9726	0.5612	0.4977	0.8566	0.7304	0.1416	0.0474
7.2586	8.2268	7.8103	6.8802	0.3995	0.3195	0.6848	0.5590	0.4463	0.9132	0.2380	0.0777
1346.1909	1498.3180	1398.8673	1847.7916	0.4472	0.5725	0.1441	0.4806	0.1205	0.2517	0.5913	0.1281
30.8502	32.8141	33.6969	29.3708	0.7516	0.9666	0.5872	0.6018	0.3247	0.8935	0.5974	0.3925
5.6812	5.7143	5.7390	4.9934	0.1816	0.3108	0.6454	0.2154	0.6972	0.8457	0.9679	0.2792
8.7139	10.0900	10.7201	8.2147	0.5384	0.9131	0.7165	0.2586	0.1289	0.2414	0.2870	0.0740
151.2426	135.2295	124.0388	116.0490	0.9029	0.9320	0.7072	0.1236	0.3927	0.4218	0.4838	0.7061
64.6268	57.2703	66.3959	46.1259	0.1794	0.6154	0.3257	0.4662	0.1860	0.7166	0.5905	0.1390
5.9113	5.4466	5.5117	4.8684	0.6106	0.5220	0.0906	0.9849	0.0740	0.9721	0.5851	0.2705
6.8925	7.1088	7.4847	5.9971	0.8492	0.3367	0.5808	0.5240	0.6292	0.3356	0.8660	0.1713
13.1625	12.6538	12.2631	11.1071	0.2754	0.7995	0.0874	0.8263	0.0872	0.3347	0.8462	0.6158
489.8904	473.6462	537.5166	426.0320	0.0672	0.4734	0.4664	0.9595	0.8636	0.5989	0.8917	0.1418
10.9088	11.6179	10.3627	10.4662	0.0276	0.5284	0.1490	0.5666	0.3641	0.4722	0.7370	0.9359
6.6097	6.4019	7.4769	6.6374	0.0314	0.8051	0.1631	0.5327	0.4934	0.5895	0.7017	0.1275
10.6868	10.2816	11.5218	12.1830	0.9087	0.6388	0.1613	0.9499	0.3700	0.5552	0.7447	0.5246
61.3595	53.1769	53.7764	56.5766	0.9871	0.6006	0.1080	0.8006	0.3139	0.1990	0.5118	0.8179
7.7135	8.1750	9.7706	9.0944	0.2893	0.1994	0.6385	0.5850	0.3039	0.0489	0.7388	0.7182
36.2344	32.5748	36.2733	28.8615	0.2085	0.8574	0.2711	0.3049	0.1298	0.1641	0.6702	0.3298
4.3545	4.2408	3.9114	3.7858	0.1887	0.1353	0.9160	0.5012	0.2177	0.2699	0.7938	0.7720
5.6862	6.9994	5.8733	4.7405	0.3529	0.2063	0.5561	0.6302	0.5829	0.4778	0.4186	0.1379
218.8442	200.9496	192.2262	146.7450	0.2079	0.5548	0.3148	0.2117	0.0320	0.2224	0.7271	0.3247
47.3884	69.0441	52.7001	58.2733	0.3040	0.5438	0.2816	0.8554	0.2259	0.8945	0.0827	0.6795
19.9121	25.0388	20.6197	23.1829	0.7190	0.5268	0.0164	0.3935	0.0373	0.5979	0.1433	0.3837
11.1325	6.9180	11.4530	6.0979	0.9916	0.0740	0.1940	0.3375	0.2736	0.2039	0.0878	0.0418
6.7581	7.3581	7.5411	6.1471	0.5072	0.1542	0.5693	0.8999	0.7854	0.1207	0.2814	0.0601
7.9450	6.7682	7.2608	6.6896	0.8182	0.1362	0.5250	0.4912	0.5075	0.1933	0.0772	0.3877
9.6174	8.4537	11.2697	9.9182	0.8668	0.3280	0.5459	0.4372	0.9945	0.3902	0.3178	0.4689
7.5147	6.9326	7.4801	7.2261	0.9697	0.3009	0.8654	0.2741	0.7667	0.1830	0.2682	0.5610
13.8998	14.4097	13.2959	10.8922	0.2586	0.7836	0.4002	0.5988	0.3719	0.9316	0.8575	0.3637
4.4632	4.6642	4.3410	3.6351	0.4309	0.9018	0.6540	0.8322	0.1251	0.4611	0.7450	0.1629
69.3415	94.3234	70.0678	87.3185	0.2983	0.4128	0.8472	0.7774	0.1146	0.7555	0.2260	0.3271
1058.3413	1268.1834	1047.9516	1029.7326	0.1119	0.0863	0.2906	0.1564	0.1062	0.4805	0.1895	0.8922
22.6879	22.6307	22.0073	21.2231	0.9764	0.8521	0.6739	0.3971	0.1664	0.9754	0.9894	0.8406
5.3515	5.9110	6.2339	5.2318	0.4861	0.4769	0.6505	0.6543	0.3258	0.2301	0.3630	0.0083
170.9398	232.8555	147.2702	192.4886	0.2679	0.6832	0.0928	0.3843	0.1078	0.8795	0.3839	0.2860
32.5500	42.0571	32.4041	43.9780	0.7393	0.8307	0.4798	0.4933	0.2027	0.7059	0.4842	0.4681
5.8613	5.1464	5.9275	6.2291	0.9203	0.5623	0.0069	0.5496	0.4213	0.0809	0.0539	0.7163
41.1117	41.8298	43.5031	72.1538	0.1896	0.2466	0.3134	0.8672	0.2163	0.8261	0.9582	0.0592
8.0093	8.6532	7.8922	8.0502	0.5035	0.4175	0.8398	0.2820	0.4311	0.2499	0.6836	0.9233
691.9973	677.3456	700.7742	585.5115	0.3364	0.7086	0.4692	0.7686	0.7627	0.6755	0.8722	0.1330
6.9346	6.5749	7.5430	7.5018	0.9864	0.5510	0.2552	0.0931	0.3890	0.4319	0.6791	0.9717
9.6828	8.0464	9.3349	5.7213	0.6956	0.8117	0.2664	0.8248	0.1689	0.4056	0.4519	0.0553
4.6514	4.8535	4.9485	4.4278	0.7737	0.8290	0.2159	0.5431	0.2700	0.7195	0.6198	0.3315
4.7313	5.2839	5.1203	4.3985	0.2434	0.8893	0.8337	0.2802	0.1644	0.5031	0.3810	0.2207
20.9492	25.7767	28.4991	27.5137	0.4216	0.7034	0.7212	0.5734	0.1604	0.7983	0.2017	0.8609
4.7044	4.9511	4.4886	4.8520	0.6225	0.0314	0.0749	0.0829	0.3294	0.1761	0.5099	0.4115
15.4884	14.3797	18.6236	17.3425	0.9022	0.0610	0.6844	0.2017	0.2023	0.8726	0.6373	0.7020
126.4073	100.1283	122.4499	120.0701	0.4081	0.5176	0.5089	0.0123	0.3278	0.5573	0.3136	0.9319
12.3303	10.0810	11.6528	11.4089	0.7723	0.9517	0.5386	0.7573	0.8267	0.9142	0.1262	0.8746
9.7095	9.3337	9.6718	8.8906	0.5765	0.2045	0.7692	0.4843	0.8378	0.5355	0.8281	0.4731
19.2156	22.5175	22.3673	16.8836	0.8962	0.4658	0.3170	0.5938	0.9289	0.5821	0.2024	0.0698
26.5890	25.6795	24.5790	19.2928	0.3579	0.4296	0.1774	0.8062	0.1184	0.2134	0.8327	0.2376

230.7687	293.0217	232.4921	277.4794	0.2981	0.8921	0.1780	0.4280	0.0593	0.9611	0.1908	0.3240
12.9965	9.9008	10.7780	11.9750	0.1722	0.9682	0.2290	0.9105	0.9973	0.5631	0.1309	0.6151
57.8198	53.3831	65.1719	72.4218	0.8734	0.0478	0.8694	0.0858	0.2601	0.4448	0.6264	0.5271
3.6318	3.2652	3.4604	2.6504	0.7923	0.3158	0.0122	0.5936	0.1189	0.0972	0.5638	0.1017
9.9969	9.0080	10.2290	8.6683	0.6237	0.2478	0.4876	0.8724	0.7707	0.0477	0.6044	0.4249
24.3689	31.1590	25.8186	25.6257	0.3818	0.2796	0.4195	0.2257	0.2614	0.5703	0.0776	0.9345
7.6132	7.1900	8.5952	7.1989	0.5468	0.3461	0.9018	0.0912	0.9706	0.8003	0.6966	0.1510
4.2733	4.4160	4.2946	4.2948	0.9989	0.4034	0.0040	0.7253	0.2826	0.0453	0.7244	0.9995
1202.9888	995.0297	1006.1148	807.1475	0.5738	0.7332	0.5365	0.6917	0.0479	0.7969	0.3953	0.3206
15.6629	15.4211	19.5615	19.1651	0.3767	0.6390	0.3708	0.5696	0.1178	0.8883	0.8588	0.8847
3.5884	3.7389	3.7523	3.6346	0.6585	0.8488	0.0031	0.7390	0.1929	0.8357	0.5165	0.7610
8.7787	6.7219	8.5204	7.7606	0.8215	0.8905	0.7367	0.1915	0.3589	0.3505	0.0064	0.2017
12.6736	13.8460	16.8986	19.5621	0.6928	0.3278	0.9454	0.0854	0.1958	0.4594	0.6781	0.5539
49.6505	67.2953	56.1926	55.9459	0.1695	0.5436	0.1497	0.9442	0.4140	0.5353	0.1388	0.9686
22.2500	29.8704	24.2909	22.8426	0.1436	0.5545	0.4854	0.2564	0.1821	0.5896	0.0995	0.7670
10.6223	10.3769	9.3861	8.7400	0.0855	0.8881	0.7659	0.3441	0.0926	0.9766	0.8874	0.6710
6.2175	5.9320	6.2187	6.3023	0.5117	0.6854	0.5718	0.5104	0.6095	0.7355	0.8021	0.9550
4.5072	4.6175	5.0335	4.2381	0.3460	0.2359	0.1177	0.3146	0.3482	0.0950	0.8403	0.0306
14.4182	15.8045	17.2529	14.5429	0.2616	0.2621	0.4308	0.6813	0.1348	0.8224	0.4997	0.3220
4.9583	5.1016	5.1795	4.3982	0.1015	0.9469	0.0290	0.0782	0.5915	0.1928	0.7897	0.1699
5.6434	6.0061	5.7759	5.2063	0.5370	0.5995	0.1290	0.3378	0.3681	0.5597	0.3700	0.3330
11.3862	12.0943	12.4881	12.4469	0.1890	0.8022	0.7139	0.1137	0.4987	0.8689	0.7129	0.9863
3.9532	3.9190	3.7822	3.7496	0.9414	0.5689	0.2326	0.6373	0.1967	0.6082	0.9447	0.9332
77.6586	104.5381	76.3677	93.1549	0.2819	0.9300	0.1519	0.6751	0.1894	0.6678	0.1058	0.3897
5.6045	5.7250	6.0425	5.7283	0.3254	0.2765	0.8281	0.9248	0.2325	0.5484	0.8029	0.4876
498.1501	516.2727	398.7969	505.5439	0.0116	0.6017	0.2160	0.2049	0.9927	0.7478	0.8731	0.4245
7.5855	7.6679	7.6626	9.3724	0.7103	0.2301	0.3870	0.5140	0.7395	0.8522	0.8671	0.2337
78.1865	105.2665	84.8481	81.0727	0.2511	0.3805	0.5559	0.3595	0.3060	0.6104	0.3601	0.7833
5.6508	6.1261	5.6861	5.3029	0.0966	0.6738	0.6609	0.3628	0.5420	0.9402	0.3973	0.3267
1245.4380	1614.7114	1324.3508	1368.3892	0.2020	0.2068	0.1853	0.1682	0.4404	0.5576	0.0345	0.7076
7.2672	6.8678	7.5802	5.6137	0.3107	0.5766	0.1216	0.8421	0.2852	0.1800	0.5856	0.0369
11.4867	10.5955	10.1245	10.3295	0.5859	0.6814	0.3497	0.5101	0.5531	0.2565	0.6672	0.9429
4.2318	4.3220	4.5227	3.6201	0.3830	0.3090	0.1083	0.0639	0.0884	0.2807	0.8436	0.1733
29.3762	27.7845	27.9658	24.8638	0.1021	0.3993	0.4320	0.8319	0.0748	0.9334	0.7714	0.4257
5.5352	4.6547	5.8286	5.2891	0.8239	0.8817	0.7277	0.1797	0.3530	0.9579	0.0618	0.2563
51.9247	102.2781	61.7830	56.3212	0.0579	0.1701	0.5894	0.5057	0.8607	0.8229	0.1229	0.6867
10.4529	11.4800	13.5918	11.6619	0.6429	0.3565	0.0319	0.6942	0.7105	0.4599	0.4241	0.1556
21.7667	17.0301	17.3219	20.3086	0.2368	0.7789	0.1599	0.8726	0.8013	0.5571	0.1808	0.5666
18.6985	15.9526	15.4224	18.4471	0.0077	0.8458	0.0735	0.7159	0.4394	0.3318	0.2739	0.4003
11.4797	9.0090	11.3520	9.4314	0.7258	0.5396	0.6929	0.5496	0.9421	0.5610	0.1110	0.3578
251.5499	259.1366	239.0865	288.5474	0.9299	0.9177	0.1611	0.7650	0.1123	0.2298	0.8318	0.2130
168.5422	189.7628	167.7616	198.0674	0.2032	0.7328	0.1366	0.5643	0.1904	0.5146	0.6064	0.3669
182.6557	183.6612	207.9288	157.9646	0.1897	0.6132	0.4346	0.6264	0.7274	0.2060	0.9629	0.0631
8.5627	6.8294	8.6622	5.7110	0.4807	0.8984	0.5127	0.8483	0.2234	0.7976	0.2665	0.1526
5.2466	5.1714	6.2327	5.4399	0.2151	0.1267	0.8245	0.3268	0.8826	0.3022	0.9080	0.1671
4.9677	4.5723	4.6404	4.3720	0.3487	0.4118	0.7580	0.5801	0.5033	0.6147	0.3380	0.3872
9.7205	8.2379	9.0333	8.6973	0.0850	0.6096	0.1766	0.8214	0.5675	0.6191	0.1350	0.8119
19.8220	20.0820	19.1703	17.6992	0.1763	0.2925	0.2873	0.5747	0.5918	0.6832	0.9167	0.5581
581.4012	422.5388	560.8221	656.2791	0.0272	0.0013	0.6888	0.0054	0.2289	0.3224	0.0402	0.6051
31.1894	25.1962	32.0699	26.8063	0.6515	0.4897	0.0086	0.3819	0.0148	0.1564	0.3105	0.3740
88.3809	76.5181	87.7734	72.8712	0.1217	0.2606	0.0073	0.5465	0.1108	0.0419	0.3726	0.3642
5.3379	5.3622	5.7398	4.8590	0.1655	0.5234	0.5030	0.9172	0.4992	0.2113	0.9457	0.0662
6.8902	7.2278	7.2484	6.7289	0.5959	0.4886	0.3221	0.1334	0.1961	0.9721	0.5685	0.3541
42.8033	55.1112	49.9018	54.2541	0.4040	0.4956	0.5154	0.9898	0.1687	0.3002	0.2698	0.6562
3.8158	4.1276	3.5337	3.4492	0.5769	0.6111	0.4578	0.8042	0.1380	0.7205	0.3809	0.8401
19.1163	23.0033	23.1590	20.0923	0.1637	0.1457	0.6567	0.5177	0.2177	0.4805	0.2388	0.5715
5.6156	6.1394	5.3466	5.4339	0.9764	0.0408	0.9323	0.1643	0.2485	0.2240	0.2742	0.8898
129.0503	128.9506	130.4735	138.5511	0.8830	0.9375	0.6536	0.9565	0.1592	0.4602	0.9956	0.6013
6.2044	5.8951	6.2696	5.0451	0.4332	0.5799	0.3580	0.6233	0.4596	0.9974	0.6715	0.1816
6.5500	6.3276	7.1705	5.5915	0.0368	0.9335	0.1895	0.3265	0.7821	0.5193	0.6522	0.0339
211.7893	195.9825	208.0198	221.9462	0.2139	0.7376	0.0503	0.7426	0.6793	0.3139	0.6361	0.6785
3.5777	3.6171	4.6946	4.0859	0.8768	0.0694	0.2511	0.3452	0.2560	0.4269	0.9327	0.4090
221.9572	174.4102	213.4749	272.8034	0.3469	0.3537	0.6040	0.9944	0.0633	0.1147	0.2002	0.1938
49.1145	59.9447	42.4928	50.9601	0.0681	0.2127	0.0621	0.2333	0.7146	0.7010	0.3236	0.5258

17.5222	16.4662	18.3907	15.5171	0.0901	0.8894	0.0612	0.7411	0.0288	0.2731	0.8016	0.5040
5.1930	5.4101	5.6080	6.9409	0.1270	0.8072	0.3581	0.4589	0.6637	0.2377	0.7550	0.2009
6.3284	7.4589	7.0489	6.1012	0.1408	0.5423	0.9718	0.2578	0.1240	0.4052	0.1178	0.1637
4.8507	5.5221	6.5502	5.0882	0.6486	0.0543	0.0158	0.9752	0.9761	0.7610	0.1568	0.1194
3.1803	3.5951	3.2750	2.6939	0.3098	0.6032	0.3508	0.9200	0.6891	0.7348	0.2822	0.1700
52.1021	52.9933	60.2469	51.8714	0.0119	0.6650	0.2410	0.9151	0.4665	0.4885	0.9264	0.4894
9.9192	7.2459	9.3086	9.8161	0.1149	0.5713	0.9335	0.2686	0.9981	0.7547	0.1294	0.7607
25.5479	23.5989	24.0649	21.6029	0.5770	0.5922	0.2205	0.8400	0.0634	0.7525	0.7331	0.6996
42.1845	33.0660	35.0436	35.6937	0.6321	0.6218	0.1481	0.8020	0.5224	0.3197	0.2668	0.9403
48.1652	44.2607	46.3157	44.3092	0.4150	0.6498	0.0233	0.2675	0.3810	0.6057	0.5390	0.8161
35.1913	33.2259	34.6390	27.8564	0.2021	0.4625	0.1174	0.2254	0.1675	0.1141	0.7718	0.2818
4.6659	4.6206	4.6956	4.6916	0.2310	0.0861	0.1896	0.5141	0.0909	0.8817	0.9405	0.9971
6.8761	8.5477	8.2943	8.2264	0.1361	0.0855	0.7463	0.4277	0.0881	0.9518	0.0415	0.9684
281.1026	292.2347	270.3832	283.0272	0.4834	0.8096	0.9628	0.9652	0.2730	0.6516	0.7620	0.7351
11.6910	12.4011	13.5750	12.6474	0.7816	0.1445	0.5873	0.4819	0.1936	0.7525	0.5538	0.5003
5.8987	5.2161	5.5461	5.1220	0.9770	0.0667	0.7399	0.0930	0.6229	0.5923	0.1237	0.2757
4.5787	5.1104	6.1611	4.7812	0.4808	0.1157	0.2115	0.4355	0.2901	0.5161	0.3525	0.0639
11.5913	10.0890	10.5231	10.2835	0.0080	0.8316	0.2739	0.9209	0.6958	0.2352	0.2621	0.8300
6.0046	6.0619	6.5702	5.3399	0.3939	0.5303	0.0406	0.8422	0.3273	0.1772	0.9435	0.1539
98.5585	95.1082	108.0074	84.6241	0.6735	0.3155	0.5748	0.8560	0.0932	0.6935	0.9029	0.4747
1952.8904	1898.5291	1951.8919	2023.9246	0.7268	0.0596	0.9000	0.8353	0.5734	0.7556	0.7223	0.6745
7.9835	6.8317	7.7665	5.9416	0.2828	0.3910	0.4409	0.8015	0.1068	0.9882	0.4143	0.2365
8.3542	8.3113	9.4432	7.8865	0.7077	0.3961	0.1820	0.4794	0.0407	0.2942	0.9627	0.3394
40.0877	47.3921	49.6070	49.1975	0.5329	0.6047	0.4049	0.5361	0.2398	0.8364	0.4626	0.9612
191.7814	213.3678	204.8074	185.7764	0.0356	0.9466	0.6325	0.6850	0.4514	0.3965	0.6251	0.5120
156.3516	153.4831	136.3353	136.5619	0.3188	0.6413	0.9512	0.3809	0.3070	0.1732	0.9110	0.9894
254.7704	312.3066	261.8829	287.4137	0.2690	0.7990	0.5097	0.3407	0.0912	0.4174	0.1490	0.6375
6.8512	6.3068	6.8898	7.0308	0.6696	0.8204	0.3920	0.7492	0.7972	0.2313	0.3644	0.8301
52.7252	43.3960	49.1293	39.6640	0.0287	0.5150	0.2191	0.2373	0.0401	0.9839	0.2521	0.2908
3.3237	3.2143	3.4706	3.2592	0.4330	0.5082	0.2582	0.6552	0.5615	0.3011	0.7462	0.5450
215.6762	202.1205	240.7672	185.9005	0.4999	0.5962	0.2551	0.4779	0.2532	0.6201	0.7612	0.3049
655.3160	641.9943	556.6582	660.3447	0.7122	0.5965	0.0113	0.5251	0.1455	0.6921	0.9131	0.2409
7.2162	6.7935	7.2592	5.2908	0.2971	0.4226	0.0634	0.2845	0.0377	0.2630	0.6565	0.0264
225.6938	218.2274	246.7123	223.7437	0.6143	0.2942	0.8156	0.8673	0.4557	0.8378	0.8651	0.6815
6.2322	6.8964	6.5553	6.3417	0.3473	0.3951	0.1282	0.4613	0.8532	0.9573	0.4508	0.8619
1098.2874	2232.5226	1424.6901	1543.0964	0.2645	0.2388	0.4435	0.4392	0.0857	0.4753	0.0088	0.7039
13.1923	16.2862	14.6066	12.2741	0.2509	0.2799	0.5848	0.7686	0.6798	0.6174	0.2665	0.4123
5.5733	5.7878	5.5765	5.3883	0.3918	0.5170	0.5554	0.5856	0.4746	0.9978	0.4626	0.5945
20.9817	18.9410	20.0570	19.7438	0.9055	0.7387	0.3428	0.8048	0.5115	0.2508	0.4358	0.9102
473.6480	358.6659	450.3153	469.8599	0.1727	0.0343	0.7810	0.1288	0.3311	0.4279	0.1034	0.7970
20.4728	15.4340	17.0837	17.4893	0.3468	0.8541	0.3102	0.5912	0.8202	0.2423	0.1752	0.9290
237.6206	196.7741	226.8097	178.2276	0.5897	0.5274	0.0681	0.3097	0.0272	0.1703	0.4725	0.4504
12.8496	10.5202	13.8367	9.0159	0.8843	0.9363	0.0131	0.4516	0.0826	0.4984	0.3935	0.0774
4.0318	4.0877	4.0023	4.1478	0.0613	0.5491	0.3578	0.9509	0.0209	0.8842	0.8871	0.7368
28.9512	33.8527	33.1684	28.7079	0.5133	0.4657	0.6055	0.8666	0.5148	0.9298	0.3036	0.2494
21.7521	15.9636	18.3220	19.2413	0.4248	0.7882	0.1885	0.9773	0.8516	0.2333	0.2013	0.8670
4.5537	4.7282	4.7492	4.2318	0.5432	0.6808	0.9815	0.4779	0.3438	0.7326	0.7426	0.2528
14.8563	12.5379	12.8106	13.6731	0.2563	0.7889	0.0934	0.8297	0.5234	0.2823	0.3200	0.7511
9.0552	8.4007	8.5183	8.0713	0.0613	0.6696	0.1563	0.2018	0.1468	0.3477	0.4495	0.5759
13.5489	16.1254	14.8964	15.3690	0.3495	0.2548	0.0536	0.4736	0.1208	0.7751	0.2461	0.8323
2189.1845	2283.0183	2158.5701	2392.6137	0.2983	0.3882	0.6822	0.8061	0.3931	0.6985	0.7095	0.4551
149.7707	139.1807	146.5825	178.7706	0.9657	0.2622	0.9309	0.8087	0.1227	0.9864	0.6716	0.2707
183.0882	182.2975	193.7296	173.1100	0.7803	0.3754	0.5192	0.9036	0.1051	0.4869	0.9849	0.6365
48.4463	57.2442	61.1287	57.7763	0.3373	0.3931	0.6795	0.8537	0.0853	0.6930	0.3065	0.7416
7.3927	6.5697	7.1974	6.6655	0.4930	0.1073	0.9776	0.1789	0.8620	0.2845	0.1533	0.7408
21.7763	23.4005	25.1432	19.8599	0.5237	0.0139	0.0747	0.0550	0.7006	0.1716	0.5930	0.1418
24.1546	18.0559	20.7758	14.1309	0.9269	0.6916	0.1825	0.4861	0.0545	0.3010	0.1727	0.1037
7.2048	7.0293	7.0946	6.5185	0.4106	0.8882	0.0229	0.4552	0.8187	0.2568	0.7447	0.3337
4.4299	5.2473	4.5990	4.7675	0.3483	0.2114	0.8472	0.0755	0.2738	0.3564	0.1206	0.8551
75.5280	78.3074	73.0041	67.9342	0.0683	0.7916	0.5812	0.6387	0.1903	0.4539	0.8229	0.6006
11.3314	12.3221	11.7681	12.2618	0.5564	0.1362	0.8673	0.3239	0.5272	0.7617	0.6226	0.8174
20.0045	15.4793	15.7464	17.3154	0.0507	0.8073	0.3089	0.9106	0.9516	0.8073	0.1175	0.5890
422.2292	495.2492	444.6637	500.3749	0.1838	0.5177	0.2163	0.6858	0.1632	0.7984	0.2307	0.4568
5.4185	5.9982	6.0439	5.7667	0.5981	0.7227	0.1423	0.8933	0.6823	0.8865	0.1958	0.6384

19.4404	15.5014	18.5702	18.7087	0.2490	0.8485	0.2155	0.7466	0.6697	0.3899	0.2255	0.9763
61.2844	54.5380	68.8306	62.3510	0.3760	0.5292	0.3271	0.9693	0.1544	0.7546	0.6178	0.7173
130.2464	129.4600	145.6561	125.5174	0.5248	0.4214	0.6810	0.9831	0.0822	0.9562	0.9838	0.6522
47.6546	44.6541	51.5203	44.8049	0.8945	0.6805	0.2846	0.7814	0.0351	0.7210	0.7899	0.6077
6.1290	5.6369	6.2199	5.1130	0.6333	0.1004	0.7603	0.6863	0.3787	0.9585	0.3903	0.0313
9.0742	10.1908	9.4698	9.0738	0.6701	0.1401	0.7039	0.3809	0.2135	0.3731	0.4700	0.7394
52.7652	54.5412	47.4055	60.9070	0.7616	0.0562	0.7064	0.2892	0.4238	0.5509	0.8607	0.0744
11.6415	12.2039	11.7800	13.5884	0.5422	0.7349	0.8285	0.6762	0.1908	0.8561	0.7418	0.3727
13.9811	14.4696	14.9059	14.0485	0.7916	0.7656	0.3344	0.9372	0.3068	0.2164	0.8580	0.7365
6.2434	6.2147	6.9760	5.7839	0.8670	0.3291	0.6386	0.9553	0.1904	0.2777	0.9667	0.2227
6.6530	5.7181	6.7171	6.5080	0.2564	0.6903	0.0294	0.0769	0.1797	0.7343	0.0383	0.7753
40.9422	38.6995	40.6833	33.3775	0.3289	0.5114	0.5086	0.4371	0.1077	0.8060	0.7740	0.3596
3.5775	3.1087	3.4495	2.9236	0.1624	0.6581	0.0022	0.5666	0.0645	0.3095	0.1918	0.1621
9.0725	8.0225	8.1951	8.7412	0.0292	0.5388	0.2287	0.4259	0.6435	0.2009	0.3089	0.6801
5.9828	7.4407	6.0969	5.8502	0.8378	0.9459	0.7916	0.5522	0.0404	0.5076	0.2715	0.7467
166.5939	235.0119	176.4912	157.8784	0.0906	0.3094	0.9496	0.2495	0.9658	0.9126	0.1645	0.6021
223.0460	210.1800	259.8295	220.5231	0.9688	0.5504	0.5555	0.5071	0.9043	0.8798	0.7772	0.5762
4.7362	4.5016	4.5288	4.5792	0.3969	0.7264	0.3539	0.3978	0.6150	0.4359	0.6381	0.8998
223.5305	259.0881	205.4504	169.0911	0.6249	0.6991	0.4828	0.7088	0.0552	0.8707	0.6863	0.5096
139.2710	123.6061	154.0090	95.8513	0.3353	0.7228	0.5672	0.7101	0.0547	0.7338	0.7485	0.2697
15.5072	12.8731	15.7046	12.0409	0.1826	0.6310	0.0334	0.9038	0.0548	0.4716	0.3095	0.2575
33.6252	32.2173	30.8488	34.5343	0.6961	0.7412	0.3651	0.9557	0.4498	0.8730	0.7928	0.5673
13.9003	14.4721	12.8031	11.2314	0.5790	0.2999	0.8919	0.1032	0.5508	0.7417	0.8119	0.5150
4.4658	4.1516	4.7495	4.1266	0.8474	0.2596	0.7278	0.4576	0.0408	0.9540	0.4274	0.1567
43.5267	43.2636	36.7280	38.9123	0.8876	0.3390	0.0379	0.8791	0.8870	0.9230	0.9825	0.7486
13.5507	10.3976	11.1143	11.6562	0.0215	0.8576	0.1155	0.6855	0.6494	0.6681	0.0945	0.8368
10.9439	9.0075	9.4607	9.0555	0.0209	0.9214	0.1495	0.8056	0.8710	0.2657	0.2593	0.8569
9.0771	9.1228	7.6542	7.3167	0.3759	0.6288	0.1812	0.5214	0.6206	0.4005	0.9701	0.6897
40.8132	32.6567	41.6287	27.1289	0.5151	0.9190	0.3339	0.6537	0.0194	0.3341	0.3869	0.1311
62.5206	65.6882	67.3892	61.1421	0.4695	0.8723	0.5817	0.9967	0.1200	0.5711	0.7278	0.5930
302.8542	380.3376	316.4049	325.8589	0.2348	0.4236	0.4484	0.2473	0.0960	0.8504	0.2155	0.8560
219.3722	253.5150	217.9964	237.7630	0.2198	0.9456	0.2884	0.4617	0.1589	0.7063	0.2969	0.5650
19.6889	25.3683	23.4632	23.2688	0.5029	0.3168	0.6666	0.5280	0.1513	0.6420	0.1435	0.9403
8.6729	7.5642	7.3730	7.3533	0.2641	0.1753	0.0016	0.9938	0.0901	0.8294	0.1392	0.9867
39.1514	29.6638	33.5127	36.7290	0.0965	0.7733	0.2264	0.8791	0.7713	0.3975	0.2606	0.7653
7.4060	6.3904	7.1987	5.6407	0.0832	0.4498	0.4031	0.5991	0.0289	0.9503	0.2857	0.0622
156.3465	193.2018	140.2853	176.1635	0.0998	0.5631	0.1687	0.2827	0.0595	0.5400	0.3761	0.1307
25.0866	17.5185	22.0230	22.7638	0.4855	0.8218	0.3813	0.7556	0.9955	0.4919	0.0978	0.9051
8.4438	7.6975	9.3074	7.7571	0.5393	0.7768	0.9802	0.1867	0.2526	0.7131	0.3939	0.0519
714.3715	956.6979	685.5804	696.0870	0.3950	0.8310	0.0498	0.9323	0.8681	0.4922	0.3023	0.9267
99.3408	106.2471	111.2855	94.2536	0.0996	0.7611	0.1020	0.7176	0.8089	0.3350	0.7031	0.4364
212.8399	192.5713	214.1882	173.3282	0.2821	0.2365	0.4635	0.8562	0.2934	0.2656	0.5531	0.2939
14.0323	16.3785	13.9867	12.4711	0.4384	0.1185	0.9491	0.4605	0.5635	0.5901	0.3331	0.4241
130.0482	125.7912	139.3015	124.0879	0.2698	0.4626	0.0502	0.9857	0.9550	0.2113	0.8096	0.5622
25.9116	25.2982	31.3554	29.0220	0.7087	0.3873	0.4700	0.8489	0.0748	0.9815	0.9170	0.7853
23.3219	25.4471	24.8277	25.2528	0.6526	0.7120	0.4563	0.8566	0.0673	0.7804	0.5060	0.8760
35.4872	40.8855	33.8176	43.7253	0.6011	0.4807	0.0207	0.4248	0.0568	0.1508	0.5945	0.3395
37.8605	25.1968	28.5606	31.4952	0.0768	0.8407	0.1946	0.9837	0.9657	0.4802	0.1073	0.7767
8.6760	10.7406	10.9117	10.0915	0.2030	0.2117	0.4580	0.4724	0.9674	0.7291	0.1860	0.7400
32.1986	24.7749	26.6162	30.0736	0.6818	0.7195	0.1282	0.8805	0.5271	0.1963	0.2937	0.6951
6.3302	5.2872	5.6501	4.5205	0.6842	0.9221	0.1054	0.5551	0.2377	0.4878	0.3791	0.3378
8.9493	9.1249	9.2038	9.5948	0.5538	0.5162	0.2854	0.8854	0.7938	0.7040	0.8953	0.7656
7.1230	8.3197	7.3143	8.4558	0.2356	0.9404	0.7285	0.9860	0.1404	0.7459	0.4057	0.4124
134.3977	159.1181	153.3996	132.6667	0.0384	0.2002	0.6800	0.5754	0.2883	0.4714	0.5224	0.3370
2938.6803	2711.8020	2997.5190	2007.9133	0.8374	0.7963	0.2611	0.6577	0.0210	0.9161	0.7590	0.2530
2305.1262	2709.8657	2149.5560	2862.8736	0.4765	0.8097	0.9643	0.3417	0.0164	0.6959	0.2035	0.0002
42.9924	56.9767	47.5221	45.3620	0.4015	0.4942	0.2869	0.6202	0.4508	0.4360	0.1898	0.7517
7.3092	6.6602	7.7941	5.4657	0.6060	0.4090	0.2280	0.9323	0.0676	0.0854	0.4366	0.0029
7070.2334	5759.3175	7090.6277	7706.4695	0.0114	0.0749	0.3574	0.3148	0.8185	0.2549	0.1557	0.7515
312.7450	405.0466	340.9646	397.7204	0.2994	0.8065	0.1907	0.3565	0.0620	0.6488	0.1257	0.2953
33.4233	42.6976	37.3850	36.3812	0.2938	0.2857	0.5459	0.4521	0.0561	0.4336	0.1104	0.8668
2767.5009	2522.3437	2858.8543	2875.0909	0.2898	0.5161	0.4787	0.6384	0.1806	0.7875	0.4272	0.9679
8.9910	8.0337	9.2492	7.8389	0.7005	0.4704	0.3423	0.8092	0.7670	0.7645	0.2644	0.1531
7.9721	9.5610	8.6721	8.7248	0.2012	0.2777	0.6492	0.7873	0.6444	0.8456	0.3655	0.9728

11.6653	15.4359	13.1984	10.3954	0.5723	0.4608	0.2144	0.4571	0.5942	0.5999	0.3306	0.2095
9.0696	13.7465	10.0455	10.5716	0.2119	0.0827	0.9395	0.5660	0.1150	0.0257	0.0206	0.7778
4.7874	6.0138	5.6544	5.5754	0.4044	0.2235	0.1585	0.6262	0.7280	0.3494	0.1868	0.9282
57.1831	54.7227	59.4526	54.5176	0.0787	0.9387	0.3621	0.9179	0.6458	0.2361	0.7132	0.6615
7.2128	7.0927	7.1354	5.7400	0.8247	0.3472	0.4010	0.8475	0.0630	0.7559	0.9107	0.1482
6.6729	9.2003	8.9198	6.6634	0.7624	0.7100	0.9860	0.4255	0.2619	0.8145	0.0478	0.0293
14.9529	17.1750	14.9952	16.1475	0.3305	0.7394	0.8506	0.2730	0.4258	0.4805	0.3917	0.6455
11.7254	10.5372	10.7127	9.6571	0.1577	0.5186	0.3217	0.7715	0.0431	0.8834	0.4926	0.3982
13.8021	15.7565	14.9058	14.4631	0.6537	0.4528	0.6977	0.8408	0.1970	0.7512	0.4608	0.8935
4.9587	5.4874	4.8709	4.4170	0.2171	0.5090	0.2630	0.9117	0.6889	0.2062	0.5289	0.5720
13218.1138	13975.2950	13401.6409	12652.7292	0.4545	0.5852	0.8385	0.6353	0.9161	0.8930	0.8456	0.8379
7.2357	7.6062	9.4868	8.0569	0.7726	0.6860	0.0494	0.1426	0.7491	0.8717	0.6864	0.3287
11.5681	11.2593	14.5385	12.2369	0.2765	0.0950	0.5006	0.4553	0.5832	0.7571	0.8158	0.4988
59.7367	46.5082	63.1867	39.9329	0.4750	0.7052	0.3274	0.8597	0.0260	0.4921	0.4438	0.2428
5.1975	6.9079	6.1276	4.7805	0.9745	0.6078	0.9445	0.6082	0.4423	0.0309	0.1669	0.0142
79.8065	115.9711	87.0150	90.9828	0.2049	0.4760	0.7336	0.6007	0.2974	0.4513	0.1073	0.8184
727.9925	689.3875	746.5953	599.0060	0.1202	0.8767	0.0903	0.9793	0.1477	0.3035	0.7790	0.2761
28.8880	46.7811	37.5781	32.3703	0.1473	0.1484	0.7976	0.6206	0.1746	0.9217	0.0391	0.5414
4.7553	5.8465	5.2294	5.4594	0.7473	0.9981	0.8741	0.4937	0.8939	0.1406	0.5011	0.8092
93.2253	120.5979	110.8730	100.1721	0.1637	0.3649	0.6033	0.5099	0.3507	0.9210	0.4198	0.5125
4.7189	4.9442	4.5993	4.8498	0.5356	0.0753	0.2332	0.3666	0.1808	0.2294	0.6527	0.7144
14.6757	12.5337	11.9171	12.3227	0.0971	0.8212	0.2979	0.7916	0.4832	0.5169	0.3796	0.8773
2085.7746	1930.1224	2697.1523	2088.2782	0.4460	0.2473	0.9804	0.8840	0.2794	0.9894	0.7831	0.4772
309.9008	257.7192	328.5927	285.5922	0.8015	0.7594	0.3498	0.9241	0.1147	0.7153	0.4616	0.6245
104.4371	115.4940	101.1793	114.8807	0.4007	0.6338	0.1774	0.8787	0.1735	0.6055	0.6343	0.4542
7.1915	7.1826	7.2108	6.3567	0.4926	0.2716	0.2704	0.5178	0.3622	0.2961	0.9858	0.1572
19.7971	17.9624	18.3635	15.8629	0.8369	0.5840	0.2235	0.8585	0.0695	0.5299	0.6968	0.5770
536.8103	668.4770	524.7726	521.6697	0.0775	0.3757	0.4273	0.4675	0.4175	0.8801	0.2717	0.9703
44.9146	45.9259	43.1530	47.8757	0.9541	0.0160	0.2777	0.7549	0.9553	0.1344	0.7555	0.1533
26.5070	20.3713	21.3712	24.3443	0.4752	0.8771	0.1502	0.9085	0.6850	0.3076	0.2367	0.6454
9.8496	11.3659	8.7615	9.5831	0.1303	0.4119	0.7321	0.8771	0.5152	0.1927	0.4665	0.6615
152.9327	140.6446	178.4347	124.8965	0.3010	0.6069	0.5521	0.8630	0.0521	0.7773	0.8141	0.3934
79.1170	80.9883	77.2565	91.3895	0.4030	0.2152	0.4697	0.8669	0.4554	0.3050	0.8941	0.3337
5007.5498	4681.5252	4914.2731	5204.9583	0.3373	0.1163	0.7142	0.3028	0.4803	0.2230	0.2183	0.5354
23.7547	19.6804	21.7492	21.4453	0.5491	0.6989	0.1133	0.9146	0.5277	0.3893	0.3464	0.9473
139.5103	153.3905	156.7940	132.0818	0.0306	0.9133	0.7791	0.7794	0.5292	0.3760	0.6658	0.1105
5.1544	5.2076	5.2720	6.4476	0.3177	0.0615	0.6964	0.1446	0.5364	0.5584	0.9477	0.4399
8.5065	8.0055	9.9016	8.5039	0.6678	0.3516	0.4132	0.4831	0.0069	0.2062	0.5788	0.2603
199.1471	200.0498	228.9764	240.6152	0.2503	0.1896	0.8563	0.6726	0.0975	0.8744	0.9839	0.8535
24.6433	32.0755	27.3984	23.6401	0.1223	0.3734	0.6139	0.4623	0.2278	0.6952	0.1419	0.5562
139.8270	127.5121	150.6775	127.3470	0.6209	0.3671	0.1483	0.6442	0.0857	0.4434	0.7068	0.4580
14.7643	15.1386	16.3054	13.1611	0.7868	0.5689	0.0043	0.8011	0.9291	0.0948	0.8329	0.0679
374.9537	341.9020	391.4500	295.0347	0.5615	0.6191	0.2767	0.6110	0.0213	0.5134	0.7062	0.4186
202.5282	286.2088	207.5699	224.7090	0.0976	0.9308	0.3179	0.5489	0.0437	0.7631	0.0891	0.6694
6.5326	6.8530	6.0274	7.1689	0.4297	0.4715	0.5522	0.4079	0.5000	0.6337	0.7024	0.2223
243.9119	201.8268	201.9734	167.5942	0.4909	0.8198	0.1421	0.2089	0.0287	0.5933	0.4411	0.4137
