# Supplementary material for: Longitudinal study on circulating miRNAs in patients after lung cancer resection
Source: Oncotarget. 2015 May 29;6(18):16674–85. doi: 10.18632/oncotarget.4322 (PMC4599298; doi:10.18632/oncotarget.4322)
Supplement: Supplementary file 4 [file oncotarget-06-16674-s004.pdf]

A	B	C	D	E	F	G	H	I	J	K	L	M	N	O	P
	sum of comparisons with significant p-value	# of comparisons with significant p-value	# of comparisons with significant p-value	without metastases								with me			
miRNA	p 0.05	without metastases p 0.05	with metastases p 0.05	non-cancer control vs TP1 p-value	non-cancer control vs TP2 p-value	non-cancer control vs TP3 p-value	non-cancer control vs TP4 p-value	non-cancer control vs TP5 p-value	non-cancer control vs TP6 p-value	non-cancer control vs TP7 p-value	non-cancer control vs TP8 p-value	non-cancer control vs TP1 p-value	non-cancer control vs TP2 p-value	non-cancer control vs TP3 p-value	non-cancer control vs TP4 p-value
hsa-let-7a	0	0	0	0.08981	0.51210	0.50175	0.96588	0.30814	0.99440	0.44704	0.84790	0.99343	0.34315	0.96209	0.86957
hsa-let-7b	0	0	0	0.24782	0.80425	0.78798	0.68021	0.40620	0.74912	0.40632	0.86992	0.73509	0.26631	0.68446	0.90379
hsa-let-7b*	1	1	0	0.07287	0.95834	0.47510	0.34841	0.04562	0.45334	0.13307	0.45013	0.34498	0.77247	0.26387	0.57814
hsa-let-7c	0	0	0	0.28559	0.58279	0.49353	0.84425	0.26575	0.91073	0.55254	0.74967	0.21127	0.24402	0.99164	0.75417
hsa-let-7d	0	0	0	0.05678	0.41042	0.61220	0.86836	0.21142	0.82892	0.69697	0.87656	0.63593	0.31035	0.49950	0.74685
hsa-let-7d*	1	0	1	0.31197	0.98977	0.09434	0.33980	0.88160	0.69546	0.36317	0.79227	0.35058	0.01980	0.72876	0.37769
hsa-let-7e	1	0	1	0.05657	0.71456	0.54162	0.24290	0.38206	0.30156	0.19555	0.17841	0.12301	0.90596	0.10516	0.23381
hsa-let-7f	1	1	0	0.04486	0.51014	0.68933	0.91256	0.42798	0.72559	0.47690	0.72563	0.67532	0.38418	0.77676	0.72412
hsa-let-7f-1*	2	2	0	0.03861	0.58311	0.84292	0.46127	0.03917	0.23615	0.13933	0.60328	0.55675	0.91611	0.25176	0.73453
hsa-let-7g	0	0	0	0.17399	0.96571	0.88344	0.76236	0.50214	0.87396	0.46774	0.79151	0.55711	0.43996	0.78174	0.80312
hsa-let-7i	0	0	0	0.11223	0.94756	0.69660	0.55459	0.42289	0.80287	0.36565	0.60742	0.27868	0.38636	0.73003	0.79067
hsa-miR-1	15	8	7	0.00470	0.04155	0.01531	0.01390	0.01612	0.01914	0.01062	0.01194	0.00472	0.05620	0.02186	0.01290
hsa-miR-100	1	0	1	0.42596	0.69345	0.23805	0.71533	0.97476	0.75625	0.24998	0.58216	0.80251	0.05608	0.01361	0.05705
hsa-miR-101	1	0	1	0.86370	0.99407	0.18677	0.51721	0.28958	0.43071	0.83145	0.34930	0.00430	0.66730	0.97116	0.55706
hsa-miR-103	0	0	0	0.47831	0.17477	0.23832	0.57679	0.07119	0.26276	0.92996	0.78487	0.33994	0.72750	0.26470	0.50871
hsa-miR-106b	0	0	0	0.72708	0.89129	0.24858	0.29881	0.17157	0.32613	0.45873	0.51816	0.24614	0.90614	0.80148	0.40698
hsa-miR-107	0	0	0	0.53777	0.61779	0.94718	0.73926	0.85781	0.85645	0.27956	0.71938	0.23871	0.88404	0.27314	0.61610
hsa-miR-10a	1	0	1	0.98640	0.95338	0.46770	0.31141	0.13277	0.51228	0.73678	0.78792	0.67863	0.83496	0.04326	0.58130
hsa-miR-10b	1	0	1	0.12700	0.98799	0.72789	0.17900	0.06420	0.77559	0.62128	0.28909	0.62345	0.78779	0.01400	0.18643
hsa-miR-10b*	6	6	0	0.00178	0.00441	0.00156	0.03554	0.01010	0.01501	0.25712	0.43349	0.30293	0.99068	0.32334	0.17819
hsa-miR-1180	1	1	0	0.48981	0.66860	0.49541	0.17063	0.69157	0.23823	0.99647	0.01819	0.81134	0.20555	0.53006	0.71537
hsa-miR-1181	2	2	0	0.00455	0.12472	0.37824	0.05998	0.23574	0.31385	0.61562	0.00471	0.32086	0.78202	0.45788	0.40002
hsa-miR-1182	0	0	0	0.75189	0.65451	0.95246	0.71894	0.07820	0.77538	0.58697	0.86078	0.37560	0.22490	0.60641	0.29029
hsa-miR-1183	1	0	1	0.29547	0.22004	0.15382	0.92877	0.13531	0.21887	0.27255	0.77829	0.38666	0.01206	0.14114	0.11692
hsa-miR-1202	1	1	0	0.09834	0.07399	0.00194	0.09745	0.11030	0.10223	0.19034	0.32521	0.92637	0.69153	0.70053	0.67448
hsa-miR-1207-5p	0	0	0	0.44902	0.94216	0.71756	0.96411	0.51522	0.94034	0.38706	0.51339	0.17872	0.43425	0.44014	0.76915
hsa-miR-1208	15	8	7	0.00066	0.00005	0.00014	0.00014	0.00005	0.00008	0.00010	0.00032	0.00312	0.00004	0.02073	0.00178
hsa-miR-122	5	2	3	0.63286	0.09823	0.03103	0.24604	0.15269	0.03210	0.59199	0.65008	0.77721	0.02221	0.82341	0.08749
hsa-miR-1224-3p	13	8	5	0.00005	0.00272	0.00093	0.02946	0.00137	0.00125	0.00044	0.01262	0.00603	0.05559	0.01169	0.02826
hsa-miR-1224-5p	1	1	0	0.09565	0.11859	0.05470	0.33591	0.03835	0.21746	0.16132	0.17855	0.90387	0.81423	0.09984	0.53092
hsa-miR-1225-3p	0	0	0	0.25998	0.46257	0.33610	0.69724	0.50730	0.87159	0.65180	0.85079	0.44351	0.95595	0.91185	0.72985
hsa-miR-1225-5p	0	0	0	0.98941	0.16258	0.90909	0.81913	0.74132	0.45436	0.62272	0.91293	0.20891	0.08046	0.17114	0.22776
hsa-miR-1226*	0	0	0	0.91947	0.83066	0.54682	0.69049	0.28450	0.98501	0.40196	0.90131	0.44729	0.54496	0.12179	0.89081
hsa-miR-1227	8	8	0	0.00024	0.02206	0.01400	0.03695	0.00011	0.00714	0.01347	0.04005	0.08637	0.42958	0.16531	0.26387
hsa-miR-1228	0	0	0	0.16753	0.95993	0.59791	0.26425	0.26952	0.86437	0.13980	0.38090	0.26625	0.60900	0.18413	0.35962
hsa-miR-1228*	0	0	0	0.97894	0.83431	0.39369	0.18005	0.87624	0.25577	0.89631	0.05089	0.87201	0.07132	0.87156	0.48870
hsa-miR-1229	0	0	0	0.19676	0.80323	0.96221	0.61068	0.16073	0.94518	0.32748	0.72545	0.74001	0.54541	0.18893	0.86868
hsa-miR-1234	0	0	0	0.29377	0.66108	0.36920	0.86975	0.21723	0.91414	0.21462	0.73604	0.55310	0.73481	0.37610	0.72520
hsa-miR-1236	2	2	0	0.00188	0.17024	0.07656	0.09465	0.04250	0.17338	0.24757	0.12130	0.26327	0.31211	0.15009	0.07898
hsa-miR-1237	0	0	0	0.45863	0.63958	0.45520	0.85894	0.13150	0.91296	0.21064	0.73580	0.43314	0.95648	0.74413	0.97005
hsa-miR-1238	0	0	0	0.40626	0.42906	0.31505	0.82286	0.32354	0.73422	0.40099	0.86025	0.49273	0.93246	0.58332	0.97376
hsa-miR-124	12	8	4	0.00811	0.00017	0.00103	0.00186	0.00033	0.00115	0.00509	0.00051	0.00214	0.00016	0.06452	0.08992
hsa-miR-1246	0	0	0	0.39480	0.41097	0.26397	0.11428	0.13393	0.85091	0.42514	0.10805	0.30826	0.79294	0.99319	0.99504
hsa-miR-1249	2	0	2	0.05244	0.08048	0.31699	0.11332	0.49459	0.11821	0.27349	0.10068	0.03339	0.16897	0.03186	0.07073
hsa-miR-1254	12	8	4	0.03346	0.03232	0.02667	0.01995	0.00710	0.03903	0.01109	0.02136	0.38514	0.06771	0.01723	0.06775
hsa-miR-125a-3p	9	7	2	0.00198	0.00036	0.05929	0.00526	0.02669	0.00216	0.00120	0.01247	0.08733	0.09243	0.19240	0.03103
hsa-miR-125a-5p	0	0	0	0.23285	0.91074	0.34200	0.79545	0.99781	0.80609	0.44295	0.65399	0.27107	0.98248	0.09992	0.41141
hsa-miR-125b	0	0	0	0.32871	0.80806	0.66585	0.54831	0.77411	0.57353	0.99800	0.69249	0.13006	0.59612	0.76642	0.75691

hsa-miR-125b-1*	0	0	0	0.73653	0.16232	0.32378	0.67440	0.59622	0.14795	0.93114	0.73439	0.53890	0.07542	0.96868	0.72570
hsa-miR-126	0	0	0	0.51951	0.41770	0.63265	0.72013	0.34171	0.59794	0.42370	0.27339	0.38152	0.74831	0.66842	0.46770
hsa-miR-126*	1	0	1	0.18305	0.79714	0.41501	0.20870	0.77094	0.13942	0.12850	0.15940	0.15166	0.25506	0.46133	0.99471
hsa-miR-1260	11	8	3	0.00960	0.01323	0.02468	0.00185	0.00041	0.01666	0.00579	0.01530	0.10373	0.08375	0.02426	0.16979
hsa-miR-1260b	11	7	4	0.00902	0.00213	0.03375	0.00155	0.00062	0.07496	0.03045	0.00679	0.15839	0.04436	0.25111	0.40225
hsa-miR-1267	0	0	0	0.53228	0.14522	0.05657	0.40304	0.14574	0.73437	0.50723	0.65173	0.10978	0.63822	0.73680	0.41840
hsa-miR-1268	0	0	0	0.88346	0.50413	0.26311	0.54797	0.08739	0.88059	0.53155	0.87459	0.18866	0.80404	0.99938	0.94659
hsa-miR-127-3p	0	0	0	0.23795	0.47021	0.79154	0.46799	0.57197	0.86715	0.18582	0.24307	0.41299	0.44133	0.93001	0.92723
hsa-miR-1273d	2	2	0	0.13622	0.53157	0.15367	0.02560	0.02090	0.90478	0.41124	0.36086	0.85005	0.71105	0.29478	0.63663
hsa-miR-1273e	2	2	0	0.07558	0.12607	0.00600	0.05985	0.00349	0.09966	0.10012	0.07152	0.86194	0.07247	0.31206	0.59387
hsa-miR-1274a	3	3	0	0.12696	0.00647	0.31953	0.00876	0.06444	0.05732	0.08182	0.04702	0.94506	0.16043	0.22983	0.31248
hsa-miR-1274b	9	7	2	0.00622	0.00425	0.06476	0.00103	0.00172	0.00596	0.00154	0.01122	0.08992	0.23861	0.02111	0.36666
hsa-miR-1275	0	0	0	0.96799	0.62422	0.93800	0.68724	0.17698	0.60879	0.81477	0.96023	0.25837	0.29228	0.56605	0.41791
hsa-miR-128	8	5	3	0.00439	0.98779	0.62394	0.02477	0.04054	0.11378	0.02639	0.01413	0.02961	0.36883	0.08224	0.65656
hsa-miR-1280	1	1	0	0.12491	0.61182	0.88169	0.12410	0.01383	0.36904	0.07986	0.14533	0.82299	0.69717	0.24815	0.48142
hsa-miR-1281	6	6	0	0.00386	0.31356	0.15272	0.02616	0.00346	0.03951	0.00701	0.01716	0.19434	0.50915	0.10222	0.20694
hsa-miR-1285	1	1	0	0.32061	0.73941	0.99287	0.12119	0.04836	0.44405	0.36410	0.50250	0.74383	0.16917	0.85392	0.47457
hsa-miR-1287	4	3	1	0.55386	0.00198	0.01896	0.43611	0.16968	0.02150	0.40187	0.21325	0.18477	0.00050	0.77726	0.47084
hsa-miR-1288	12	7	5	0.01943	0.01710	0.01166	0.04359	0.00714	0.00274	0.00477	0.12752	0.02952	0.00135	0.00492	0.00265
hsa-miR-129-3p	2	2	0	0.02555	0.44822	0.89758	0.30760	0.00967	0.50180	0.08974	0.37001	0.33850	0.85942	0.26666	0.50343
hsa-miR-129*	2	2	0	0.00521	0.26085	0.37787	0.14556	0.00175	0.16591	0.05398	0.36677	0.28662	0.76412	0.15784	0.37092
hsa-miR-1290	0	0	0	0.42822	0.36967	0.25319	0.19467	0.38531	0.86616	0.34891	0.13218	0.29272	0.77935	0.61011	0.82541
hsa-miR-1295	10	5	5	0.11779	0.02538	0.04267	0.05421	0.03050	0.04306	0.05607	0.04750	0.01931	0.01191	0.07048	0.01904
hsa-miR-1299	3	3	0	0.29479	0.00994	0.12132	0.90099	0.02889	0.03937	0.47870	0.65331	0.05189	0.07601	0.78315	0.81481
hsa-miR-1303	3	1	2	0.72841	0.16001	0.03615	0.12019	0.83658	0.24595	0.70243	0.07304	0.13970	0.03135	0.88731	0.02163
hsa-miR-1305	8	5	3	0.05867	0.04223	0.01064	0.13158	0.03991	0.00951	0.02572	0.36441	0.04987	0.09296	0.01724	0.00984
hsa-miR-1306	0	0	0	0.70303	0.51295	0.66968	0.10593	0.28745	0.32542	0.30766	0.05105	0.42851	0.24894	0.85039	0.82026
hsa-miR-1307	1	1	0	0.72503	0.02958	0.30350	0.17281	0.47971	0.50776	0.49599	0.14738	0.79394	0.07749	0.22685	0.07688
hsa-miR-130a	1	0	1	0.26621	0.40104	0.23237	0.17339	0.42331	0.24750	0.05531	0.10754	0.07003	0.17496	0.13159	0.06245
hsa-miR-130b	7	2	5	0.03220	0.49650	0.47112	0.39772	0.13230	0.48639	0.02369	0.23202	0.00321	0.06833	0.00865	0.02540
hsa-miR-132	0	0	0	0.11378	0.89265	0.61367	0.70071	0.17249	0.77586	0.64419	0.98967	0.61544	0.63028	0.75329	0.58465
hsa-miR-1323	0	0	0	0.31117	0.25191	0.70890	0.27710	0.77334	0.16223	0.83902	0.51820	0.20864	0.15070	0.94007	0.14541
hsa-miR-133b	3	0	3	0.22127	0.34950	0.25271	0.22796	0.47456	0.10584	0.19335	0.07030	0.63758	0.07508	0.14091	0.13436
hsa-miR-134	1	0	1	0.05075	0.62519	0.87091	0.66976	0.42925	0.46527	0.48162	0.68305	0.03618	0.93514	0.84229	0.78374
hsa-miR-135a*	0	0	0	0.78316	0.85960	0.83796	0.58223	0.46510	0.93846	0.30124	0.64078	0.32083	0.44074	0.88879	0.40249
hsa-miR-135b*	0	0	0	0.23015	0.50704	0.62921	0.23320	0.13651	0.40308	0.37870	0.30924	0.27726	0.26378	0.11108	0.16335
hsa-miR-136	0	0	0	0.82989	0.56134	0.67159	0.38723	0.51795	0.58841	0.96403	0.36732	0.17101	0.23606	0.91326	0.70627
hsa-miR-139-3p	0	0	0	0.96904	0.58004	0.37614	0.30581	0.55625	0.87264	0.52028	0.91650	0.81898	0.39671	0.09374	0.48298
hsa-miR-139-5p	3	3	0	0.46947	0.01005	0.06330	0.04809	0.04482	0.15399	0.17478	0.26078	0.19251	0.06071	0.66813	0.32648
hsa-miR-140-3p	0	0	0	0.88485	0.80591	0.74687	0.52255	0.87317	0.32465	0.96240	0.87082	0.06017	0.78822	0.64803	0.60223
hsa-miR-140-5p	2	1	1	0.02953	0.54960	0.40017	0.11870	0.46807	0.24011	0.28981	0.26966	0.04370	0.78986	0.30139	0.82602
hsa-miR-141	1	0	1	0.16008	0.16084	0.25973	0.84500	0.19904	0.68816	0.84158	0.89893	0.01304	0.10957	0.13955	0.56125
hsa-miR-142-3p	0	0	0	0.26627	0.93020	0.59888	0.56044	0.27513	0.55860	0.54345	0.82472	0.15585	0.75552	0.42659	0.28977
hsa-miR-142-5p	1	0	1	0.35033	0.90567	0.48522	0.59314	0.44493	0.24425	0.63015	0.78397	0.01578	0.85936	0.98751	0.36606
hsa-miR-144	12	7	5	0.01137	0.01307	0.01900	0.00227	0.00309	0.13270	0.00695	0.00170	0.11560	0.09270	0.04003	0.01215
hsa-miR-144*	0	0	0	0.48879	0.42994	0.11196	0.14399	0.17406	0.41008	0.29520	0.25245	0.55775	0.05112	0.12405	0.12673
hsa-miR-145	0	0	0	0.96507	0.05126	0.30557	0.96203	0.53437	0.62048	0.71479	0.42477	0.34812	0.61967	0.25372	0.95946
hsa-miR-1469	3	2	1	0.03339	0.10335	0.16161	0.15423	0.04926	0.19875	0.51568	0.46123	0.32017	0.56390	0.10111	0.32918
hsa-miR-146a	1	0	1	0.40556	0.58064	0.74631	0.29887	0.67407	0.29725	0.11930	0.20683	0.06511	0.32602	0.07586	0.10933
hsa-miR-146b-5p	1	0	1	0.54322	0.59909	0.52910	0.60665	0.26267	0.67798	0.65399	0.87761	0.02086	0.25873	0.73115	0.83505
hsa-miR-1470	0	0	0	0.57305	0.13553	0.17660	0.31653	0.55149	0.16736	0.99981	0.47754	0.11074	0.58641	0.94204	0.55488
hsa-miR-1471	0	0	0	0.99473	0.07194	0.09575	0.49488	0.08347	0.46910	0.24292	0.20286	0.34521	0.33289	0.52339	0.94926
hsa-miR-148a	7	5	2	0.00826	0.41044	0.03416	0.00455	0.01263	0.19377	0.05298	0.03116	0.18037	0.93744	0.11516	0.28856
hsa-miR-148b	7	3	4	0.02037	0.22277	0.29295	0.06270	0.37892	0.07985	0.02442	0.02354	0.01216	0.37366	0.03089	0.25850
hsa-miR-149	2	2	0	0.03320	0.77807	0.56110	0.42301	0.02888	0.49875	0.15554	0.30978	0.26485	0.71779	0.26471	0.37566
hsa-miR-149*	0	0	0	0.72829	0.25114	0.37002	0.25055	0.43930	0.62126	0.69676	0.13475	0.34231	0.14955	0.98036	0.11441
hsa-miR-150	1	1	0	0.03524	0.66726	0.34074	0.25273	0.07439	0.32711	0.10647	0.24701	0.82773	0.49690	0.21840	0.83866

hsa-miR-150*	3	1	2	0.03048	0.41704	0.22999	0.31954	0.66496	0.17795	0.75660	0.38401	0.00193	0.24326	0.03955	0.09682
hsa-miR-151-3p	1	0	1	0.18133	0.65542	0.95016	0.40648	0.83197	0.47096	0.41215	0.37979	0.02673	0.67339	0.08433	0.53409
hsa-miR-151-5p	1	0	1	0.09426	0.29756	0.72099	0.96301	0.21822	0.60292	0.53259	0.64378	0.08660	0.36453	0.34567	0.59250
hsa-miR-152	14	7	7	0.00208	0.06784	0.03091	0.00431	0.00445	0.00431	0.00154	0.00263	0.00033	0.14246	0.00303	0.01695
hsa-miR-1539	0	0	0	0.51928	0.47161	0.39656	0.59283	0.23991	0.82445	0.31441	0.67177	0.87570	0.90568	0.74657	0.90347
hsa-miR-154	0	0	0	0.52072	0.68702	0.98465	0.40984	0.70600	0.61293	0.73077	0.32778	0.66802	0.85709	0.59025	0.99932
hsa-miR-155	2	1	1	0.85971	0.23849	0.15244	0.08046	0.07779	0.05539	0.23841	0.03428	0.83941	0.02749	0.28753	0.34884
hsa-miR-15a	0	0	0	0.41204	0.27626	0.80151	0.82824	0.88646	0.80423	0.43480	0.77836	0.20457	0.71962	0.93024	0.98164
hsa-miR-15b	0	0	0	0.10278	0.78013	0.35727	0.57906	0.18486	0.66051	0.70563	0.90252	0.52611	0.50275	0.75478	0.85622
hsa-miR-16	0	0	0	0.50988	0.49326	0.81077	0.68197	0.73511	0.88411	0.63864	0.74821	0.26989	0.57531	0.80081	0.62722
hsa-miR-16-2*	2	0	2	0.46234	0.34545	0.06647	0.20745	0.18479	0.53080	0.19208	0.20314	0.24597	0.06788	0.01799	0.14556
hsa-miR-17	0	0	0	0.78485	0.47521	0.65113	0.95211	0.37177	0.69928	0.61939	0.85737	0.55369	0.90825	0.37090	0.70447
hsa-miR-17*	1	0	1	0.08800	0.51649	0.29700	0.71067	0.77614	0.44262	0.76398	0.86795	0.00937	0.24941	0.65548	0.66198
hsa-miR-181a	0	0	0	0.52772	0.60019	0.29897	0.12786	0.23798	0.12727	0.42483	0.53289	0.21682	0.93311	0.27966	0.64906
hsa-miR-181b	0	0	0	0.57082	0.19313	0.11309	0.31436	0.65946	0.06042	0.11646	0.07139	0.84662	0.11471	0.37056	0.05646
hsa-miR-181c	2	1	1	0.01964	0.43811	0.91708	0.44930	0.20451	0.74128	0.17232	0.51720	0.02805	0.79018	0.19227	0.94550
hsa-miR-181d	2	1	1	0.74066	0.74830	0.21710	0.45261	0.33128	0.07274	0.25942	0.04746	0.66890	0.20316	0.58528	0.50162
hsa-miR-1825	4	4	0	0.00918	0.35570	0.11532	0.05087	0.01203	0.05231	0.01571	0.04054	0.18219	0.54963	0.12315	0.23944
hsa-miR-183	0	0	0	0.30875	0.47772	0.58355	0.62755	0.85409	0.75152	0.76035	0.88477	0.89131	0.13860	0.85212	0.26067
hsa-miR-184	6	5	1	0.00921	0.00746	0.00276	0.06018	0.00096	0.01838	0.23119	0.27044	0.00345	0.20550	0.99245	0.24851
hsa-miR-185	0	0	0	0.87512	0.49167	0.43200	0.71385	0.48830	0.50756	0.63488	0.95137	0.17674	0.73029	0.51650	0.97278
hsa-miR-186	2	0	2	0.99129	0.29933	0.06738	0.06933	0.24631	0.20933	0.23637	0.27649	0.04597	0.09420	0.59096	0.02954
hsa-miR-186*	0	0	0	0.41874	0.44449	0.88319	0.28034	0.16765	0.56544	0.46690	0.60901	0.18060	0.30374	0.36330	0.25856
hsa-miR-187*	4	3	1	0.02614	0.05043	0.08516	0.02789	0.14553	0.06254	0.34073	0.02732	0.02601	0.14981	0.12374	0.06850
hsa-miR-188-5p	0	0	0	0.94140	0.36916	0.14179	0.96558	0.26560	0.87640	0.90348	0.92801	0.39737	0.88607	0.77519	0.90250
hsa-miR-18a	1	0	1	0.37186	0.08256	0.20550	0.50153	0.08832	0.35150	0.73415	0.98560	0.01915	0.14087	0.24843	0.92282
hsa-miR-18b	2	1	1	0.76472	0.04283	0.06161	0.22337	0.06241	0.36831	0.58632	0.43870	0.07454	0.04179	0.84373	0.33342
hsa-miR-18b*	0	0	0	0.22062	0.65473	0.27693	0.96357	0.30695	0.75136	0.68086	0.75016	0.49639	0.66951	0.84721	0.43529
hsa-miR-191*	0	0	0	0.67220	0.49155	0.17738	0.95198	0.40375	0.62700	0.40680	0.98578	0.96424	0.96801	0.50873	0.86058
hsa-miR-1911	0	0	0	0.44419	0.86055	0.68762	0.69798	0.17540	0.50398	0.43474	0.69872	0.87267	0.58415	0.44696	0.63436
hsa-miR-1914*	0	0	0	0.13321	0.62452	0.06360	0.97770	0.40559	0.16805	0.30565	0.54203	0.82385	0.72086	0.65402	0.51145
hsa-miR-1915	1	0	1	0.30680	0.34657	0.80980	0.86758	0.36110	0.66586	0.69428	0.21577	0.12367	0.28284	0.76410	0.29063
hsa-miR-1915*	0	0	0	0.67358	0.55975	0.95317	0.62164	0.51859	0.89779	0.08591	0.47931	0.98645	0.46944	0.94579	0.83392
hsa-miR-192	0	0	0	0.99262	0.98651	0.43395	0.71168	0.68219	0.67706	0.59788	0.47818	0.33680	0.11407	0.80994	0.67612
hsa-miR-193a-5p	3	0	3	0.05638	0.15742	0.14412	0.07800	0.42707	0.31300	0.09859	0.18671	0.24895	0.07890	0.02936	0.07905
hsa-miR-193b*	5	5	0	0.09596	0.04096	0.00624	0.03682	0.00777	0.02899	0.07623	0.17777	0.08579	0.30910	0.18122	0.30485
hsa-miR-194	2	1	1	0.55145	0.17433	0.01797	0.12545	0.19430	0.46810	0.18075	0.10168	0.10292	0.07037	0.55216	0.03942
hsa-miR-195	0	0	0	0.12022	0.56918	0.85407	0.35248	0.95555	0.69059	0.32537	0.43297	0.05606	0.34155	0.34875	0.99423
hsa-miR-196a	0	0	0	0.65773	0.76166	0.60195	0.53034	0.70730	0.96508	0.70052	0.30333	0.98140	0.91057	0.68848	0.79417
hsa-miR-196b	0	0	0	0.92456	0.77747	0.64106	0.86485	0.96383	0.87418	0.78070	0.47642	0.68542	0.68396	0.81158	0.69350
hsa-miR-197	7	7	0	0.02302	0.01591	0.01381	0.03282	0.01716	0.01892	0.01962	0.09623	0.53903	0.56376	0.34337	0.74381
hsa-miR-1972	0	0	0	0.76328	0.44284	0.56605	0.26872	0.10547	0.62407	0.84625	0.85258	0.41134	0.32819	0.56215	0.75257
hsa-miR-1973	7	2	5	0.08800	0.21154	0.05358	0.22933	0.02774	0.34171	0.03146	0.06170	0.00782	0.24024	0.02805	0.04606
hsa-miR-198	0	0	0	0.35601	0.96105	0.75383	0.92296	0.12643	0.90682	0.17728	0.84143	0.29547	0.14961	0.60291	0.66448
hsa-miR-199a-3p	12	5	7	0.01229	0.18478	0.18119	0.00462	0.12672	0.00935	0.00644	0.01050	0.01911	0.24200	0.01141	0.01340
hsa-miR-199a-5p	2	0	2	0.07054	0.93465	0.69319	0.29470	0.54312	0.45855	0.12192	0.16226	0.01422	0.91657	0.08766	0.07731
hsa-miR-199b-5p	5	2	3	0.43920	0.10709	0.04969	0.31850	0.26206	0.13496	0.01627	0.10987	0.49413	0.01754	0.18722	0.03156
hsa-miR-19a	0	0	0	0.53696	0.82094	0.72670	0.93187	0.64702	0.71069	0.79151	0.84887	0.16123	0.38406	0.81538	0.91460
hsa-miR-19b	0	0	0	0.51170	0.60358	0.59787	0.75152	0.26872	0.47254	0.86537	0.97276	0.07851	0.56603	0.35039	0.80840
hsa-miR-200b*	11	5	6	0.00095	0.05171	0.01697	0.08704	0.00436	0.00533	0.01004	0.07250	0.01592	0.00251	0.00553	0.00918
hsa-miR-202	0	0	0	0.73141	0.62351	0.14777	0.27782	0.29813	0.90521	0.97713	0.55836	0.64834	0.15139	0.12883	0.92026
hsa-miR-204	2	2	0	0.03835	0.16005	0.10577	0.05180	0.03021	0.31980	0.13285	0.30041	0.27355	0.25543	0.11228	0.22367
hsa-miR-20a	0	0	0	0.45285	0.68740	0.49089	0.95498	0.31682	0.87551	0.92465	0.89475	0.41154	0.57927	0.79950	0.95938
hsa-miR-20b	0	0	0	0.66923	0.58358	0.39605	0.68460	0.33998	0.76490	0.98366	0.65759	0.34796	0.35403	0.95153	0.77433
hsa-miR-21	1	0	1	0.08507	0.72200	0.94656	0.33379	0.68176	0.34899	0.40488	0.11177	0.07161	0.95243	0.25870	0.22385
hsa-miR-21*	2	1	1	0.04514	0.51004	0.18428	0.68899	0.06699	0.40708	0.14269	0.63261	0.02434	0.86120	0.16749	0.46899
hsa-miR-210	4	3	1	0.53805	0.02878	0.14250	0.00092	0.10220	0.24943	0.73621	0.03961	0.07724	0.03907	0.70363	0.05527

hsa-miR-2116*	0	0	0	0.83026	0.24914	0.15409	0.62077	0.51054	0.37789	0.77807	0.52645	0.36482	0.66157	0.97049	0.40020
hsa-miR-212	6	4	2	0.02964	0.00016	0.11438	0.04006	0.05138	0.01929	0.09779	0.05588	0.09455	0.02786	0.89264	0.09906
hsa-miR-215	0	0	0	0.20869	0.51245	0.72928	0.25778	0.46816	0.63622	0.88348	0.90955	0.08468	0.22882	0.72816	0.79550
hsa-miR-22	2	0	2	0.11154	0.27994	0.27785	0.43884	0.37900	0.67402	0.22744	0.27706	0.02633	0.56256	0.94962	0.71121
hsa-miR-22*	8	6	2	0.04048	0.19876	0.03294	0.03981	0.10050	0.02231	0.04619	0.01624	0.12473	0.26001	0.51610	0.20560
hsa-miR-221	0	0	0	0.62406	0.23351	0.85491	0.99174	0.50152	0.95157	0.51014	0.54303	0.19303	0.84151	0.49786	0.41040
hsa-miR-222	3	2	1	0.74502	0.14363	0.02056	0.09136	0.12145	0.02507	0.18231	0.08222	0.18134	0.68512	0.63049	0.02519
hsa-miR-223	0	0	0	0.29756	0.28212	0.73394	0.41792	0.31333	0.68478	0.84427	0.93688	0.31955	0.62270	0.38499	0.86500
hsa-miR-223*	2	2	0	0.00788	0.61346	0.34955	0.06948	0.01865	0.47702	0.19212	0.21536	0.24697	0.54347	0.24469	0.90653
hsa-miR-224	0	0	0	0.12361	0.30118	0.87251	0.74243	0.84285	0.43314	0.41945	0.95344	0.53357	0.30012	0.96594	0.57721
hsa-miR-2276	0	0	0	0.47800	0.38308	0.54131	0.50682	0.67563	0.33668	0.92104	0.63549	0.13816	0.23449	0.84882	0.17744
hsa-miR-2278	0	0	0	0.56652	0.98922	0.50485	0.09448	0.57787	0.42417	0.72760	0.09067	0.64905	0.17350	0.74479	0.49413
hsa-miR-23a	2	1	1	0.14297	0.37576	0.31340	0.03601	0.27983	0.13181	0.14737	0.07451	0.19979	0.41218	0.11599	0.11192
hsa-miR-23a*	2	2	0	0.04431	0.42473	0.11868	0.26061	0.01537	0.35257	0.47208	0.82846	0.86275	0.39711	0.24668	0.71729
hsa-miR-23b	1	0	1	0.33226	0.11759	0.53202	0.70648	0.12821	0.39091	0.73075	0.62512	0.70934	0.35379	0.34352	0.65597
hsa-miR-23c	0	0	0	0.39311	0.57810	0.31243	0.70327	0.21573	0.90275	0.54402	0.70422	0.77138	0.91209	0.54625	0.99008
hsa-miR-24	11	6	5	0.00901	0.09691	0.01997	0.00842	0.10884	0.03641	0.00481	0.00141	0.02770	0.17317	0.01730	0.00853
hsa-miR-25	0	0	0	0.73236	0.66119	0.88863	0.83316	0.72055	0.86220	0.52157	0.97326	0.75064	0.84641	0.56141	0.68205
hsa-miR-26a	1	1	0	0.63310	0.18527	0.23205	0.24987	0.02706	0.12064	0.32561	0.76233	0.35650	0.70146	0.96276	0.80414
hsa-miR-26b	0	0	0	0.15878	0.62535	0.98193	0.48941	0.83248	0.48878	0.41706	0.62653	0.33500	0.54371	0.59909	0.71252
hsa-miR-27a	15	8	7	0.01947	0.02350	0.03436	0.00412	0.03031	0.02180	0.02131	0.00489	0.00838	0.01614	0.01286	0.01007
hsa-miR-27b	9	3	6	0.04975	0.46439	0.24141	0.03819	0.31412	0.08128	0.03388	0.05927	0.01485	0.45380	0.02603	0.04831
hsa-miR-28-5p	1	0	1	0.07754	0.20991	0.53177	0.72195	0.34145	0.65197	0.34324	0.34586	0.32031	0.09995	0.74580	0.98239
hsa-miR-2861	1	0	1	0.09823	0.17475	0.94929	0.51874	0.64914	0.62034	0.56681	0.55935	0.15144	0.00652	0.20282	0.11916
hsa-miR-296-5p	1	0	1	0.14367	0.07182	0.27465	0.08250	0.46612	0.21756	0.19582	0.08216	0.03582	0.10392	0.05557	0.08673
hsa-miR-29a	1	1	0	0.11393	0.24064	0.28036	0.02844	0.24166	0.71637	0.62885	0.11684	0.08343	0.36469	0.69209	0.67961
hsa-miR-29b	1	0	1	0.32184	0.72200	0.22552	0.45077	0.08003	0.35729	0.41988	0.59397	0.02288	0.30541	0.67459	0.69142
hsa-miR-29c	1	0	1	0.38813	0.29960	0.94539	0.56886	0.79985	0.45604	0.62135	0.50437	0.04086	0.22818	0.98231	0.60567
hsa-miR-29c*	5	4	1	0.01366	0.15318	0.03910	0.02357	0.00599	0.05376	0.05984	0.11339	0.43470	0.15502	0.04004	0.07189
hsa-miR-300	2	2	0	0.02210	0.17519	0.11922	0.33923	0.39228	0.04265	0.09568	0.07306	0.74595	0.45249	0.38309	0.69138
hsa-miR-301a	1	0	1	0.23948	0.39620	0.17565	0.71863	0.16206	0.67842	0.87841	0.83425	0.04591	0.26635	0.27937	0.89976
hsa-miR-301b	1	0	1	0.32785	0.19442	0.53133	0.65148	0.37557	0.46212	0.84075	0.51529	0.05285	0.65622	0.00422	0.75516
hsa-miR-3074	0	0	0	0.83279	0.83240	0.48451	0.93504	0.59653	0.92263	0.39921	0.79578	0.91186	0.72301	0.56759	0.76462
hsa-miR-30a	1	1	0	0.32728	0.64420	0.96134	0.01483	0.22873	0.60068	0.20631	0.40715	0.09034	0.33959	0.15812	0.10378
hsa-miR-30b	0	0	0	0.23610	0.66590	0.73627	0.74526	0.40110	0.99070	0.38874	0.63200	0.44759	0.47133	0.43743	0.59412
hsa-miR-30c	4	3	1	0.31573	0.01159	0.00690	0.19809	0.01023	0.09117	0.36652	0.39857	0.80857	0.03031	0.55233	0.39814
hsa-miR-30c-1*	1	0	1	0.61152	0.31265	0.75421	0.72419	0.32061	0.50803	0.43942	0.43744	0.21191	0.04226	0.94858	0.25414
hsa-miR-30c-2*	0	0	0	0.20816	0.88741	0.22680	0.24253	0.15418	0.87371	0.17827	0.84220	0.43106	0.58979	0.28936	0.91206
hsa-miR-30d	3	3	0	0.10450	0.60264	0.03935	0.00238	0.05740	0.04656	0.13767	0.07301	0.26169	0.65067	0.88260	0.35895
hsa-miR-30e	4	2	2	0.89333	0.12439	0.01817	0.10359	0.06666	0.01498	0.11909	0.13522	0.04115	0.31889	0.59705	0.00882
hsa-miR-30e*	5	1	4	0.00366	0.87174	0.64135	0.32894	0.61867	0.53467	0.47343	0.14776	0.00303	0.43341	0.02352	0.56228
hsa-miR-3124	8	7	1	0.20387	0.00008	0.00099	0.01237	0.00338	0.00998	0.01297	0.00899	0.22128	0.00015	0.99771	0.80906
hsa-miR-3125	10	6	4	0.00854	0.01842	0.00837	0.10426	0.00494	0.00669	0.04619	0.13356	0.10940	0.00397	0.02810	0.03123
hsa-miR-3127	1	0	1	0.74778	0.21608	0.13959	0.42055	0.10856	0.23487	0.21496	0.71831	0.32304	0.17441	0.04539	0.20100
hsa-miR-3131	0	0	0	0.33488	0.39205	0.09858	0.80015	0.92721	0.87525	0.75100	0.62938	0.94100	0.54387	0.52968	0.52014
hsa-miR-3132	4	4	0	0.01704	0.09973	0.07339	0.01648	0.00229	0.05998	0.03335	0.07811	0.17072	0.23892	0.08003	0.08912
hsa-miR-3137	0	0	0	0.94882	0.91371	0.20032	0.91194	0.09866	0.93853	0.81586	0.74422	0.63468	0.76973	0.71032	0.93645
hsa-miR-3138	3	3	0	0.34796	0.19370	0.02313	0.18989	0.03188	0.55429	0.02875	0.14954	0.48358	0.80612	0.49046	0.21243
hsa-miR-3141	1	1	0	0.89646	0.16628	0.07347	0.17460	0.01702	0.21473	0.19678	0.23598	0.40543	0.38732	0.84863	0.80576
hsa-miR-3147	12	8	4	0.00109	0.00001	0.00004	0.00105	0.00002	0.00012	0.00036	0.00045	0.00211	0.02718	0.04516	0.48578
hsa-miR-3148	0	0	0	0.45127	0.52200	0.39723	0.12097	0.32586	0.05842	0.15769	0.07664	0.42498	0.23485	0.31928	0.45122
hsa-miR-3149	0	0	0	0.17942	0.70496	0.62951	0.36524	0.63976	0.41391	0.31508	0.35903	0.37332	0.42931	0.49551	0.82326
hsa-miR-3152	16	8	8	0.00030	0.00010	0.00009	0.00011	0.00013	0.00011	0.00013	0.00010	0.00020	0.00008	0.00068	0.00011
hsa-miR-3154	6	5	1	0.01439	0.02603	0.00135	0.00921	0.00360	0.24526	0.13880	0.25738	0.51742	0.52415	0.43587	0.28116
hsa-miR-3156	6	6	0	0.03033	0.02511	0.00903	0.18188	0.01487	0.01890	0.03425	0.18353	0.44939	0.51791	0.32502	0.62120
hsa-miR-3162	1	1	0	0.82442	0.26079	0.01004	0.31007	0.20651	0.33073	0.41038	0.59954	0.58970	0.25959	0.35923	0.78441
hsa-miR-3173	13	8	5	0.00169	0.00015	0.00030	0.00009	0.00018	0.00109	0.00276	0.00458	0.01871	0.00010	0.08511	0.00167

hsa-miR-3176	3	3	0	0.09944	0.00848	0.11477	0.04571	0.00240	0.19105	0.07002	0.37432	0.62561	0.06156	0.83144	0.48582
hsa-miR-3177	4	3	1	0.04090	0.05140	0.30561	0.01872	0.00389	0.19695	0.05755	0.11614	0.20999	0.17524	0.06384	0.64669
hsa-miR-3180-3p	0	0	0	0.05486	0.66392	0.34648	0.86627	0.76379	0.58425	0.68396	0.91693	0.97546	0.23170	0.79418	0.89241
hsa-miR-3180-5p	0	0	0	0.93603	0.45657	0.20057	0.96708	0.56089	0.85632	0.47035	0.69954	0.26180	0.88388	0.87454	0.82585
hsa-miR-3181	5	0	5	0.33694	0.46234	0.33965	0.26150	0.05707	0.26023	0.18606	0.88337	0.04840	0.37529	0.00406	0.18939
hsa-miR-3185	0	0	0	0.13312	0.96411	0.96636	0.46802	0.06959	0.37782	0.28209	0.33105	0.46360	0.17171	0.09782	0.47081
hsa-miR-3186-3p	11	8	3	0.00168	0.00001	0.00002	0.00035	0.00014	0.00019	0.00048	0.00072	0.00373	0.01053	0.21599	0.13756
hsa-miR-3187	4	3	1	0.03933	0.06887	0.03868	0.07413	0.02132	0.22550	0.11209	0.09755	0.21767	0.84458	0.02710	0.14164
hsa-miR-3188	0	0	0	0.11747	0.77604	0.91789	0.56340	0.99098	0.63535	0.49739	0.49093	0.18373	0.82635	0.62257	0.67869
hsa-miR-3189	16	8	8	0.00049	0.00055	0.00064	0.00249	0.00024	0.00112	0.00248	0.00690	0.00175	0.00037	0.01721	0.00362
hsa-miR-3190	1	0	1	0.29967	0.07408	0.16356	0.13341	0.41590	0.69086	0.95202	0.52156	0.73821	0.01077	0.45325	0.86973
hsa-miR-3194	0	0	0	0.14660	0.52407	0.09829	0.76964	0.13672	0.13661	0.16972	0.82254	0.89124	0.63008	0.54824	0.78382
hsa-miR-3195	1	0	1	0.17379	0.77897	0.56371	0.57397	0.66559	0.82632	0.46777	0.52407	0.03025	0.80641	0.26186	0.33710
hsa-miR-3196	0	0	0	0.31235	0.98716	0.38938	0.63200	0.23053	0.95117	0.36452	0.95456	0.12316	0.82199	0.78820	0.53325
hsa-miR-3197	2	0	2	0.96029	0.59692	0.57162	0.18823	0.42270	0.98191	0.31246	0.55309	0.11070	0.75989	0.01678	0.00716
hsa-miR-3198	4	2	2	0.17474	0.11109	0.01418	0.35222	0.13819	0.03119	0.09455	0.57675	0.17307	0.45821	0.03368	0.03263
hsa-miR-32	0	0	0	0.94860	0.46053	0.21060	0.94624	0.19610	0.68209	0.51057	0.57727	0.52592	0.22955	0.79672	0.82337
hsa-miR-32*	0	0	0	0.16044	0.74149	0.77562	0.23308	0.34762	0.17570	0.23857	0.23837	0.28144	0.20509	0.43006	0.32946
hsa-miR-3200-5p	14	8	6	0.01802	0.00935	0.00545	0.00615	0.00104	0.00924	0.00480	0.00909	0.01171	0.00300	0.00282	0.04468
hsa-miR-3202	0	0	0	0.28327	0.56430	0.38308	0.66749	0.31306	0.81844	0.54469	0.99934	0.25330	0.28897	0.91055	0.66421
hsa-miR-320a	0	0	0	0.34142	0.47731	0.75501	0.56432	0.91180	0.56412	0.31129	0.77829	0.06813	0.56413	0.77746	0.39210
hsa-miR-320b	1	0	1	0.82661	0.60369	0.99981	0.25137	0.92372	0.78477	0.42735	0.76808	0.03949	0.39039	0.68826	0.32844
hsa-miR-320c	2	2	0	0.02692	0.39544	0.57750	0.08398	0.00832	0.44670	0.18092	0.16504	0.93405	0.25480	0.11626	0.60973
hsa-miR-320d	1	0	1	0.83809	0.18416	0.65933	0.53853	0.29426	0.65671	0.32650	0.74922	0.04352	0.28403	0.85750	0.51773
hsa-miR-320e	0	0	0	0.83331	0.57855	0.59725	0.14288	0.72411	0.95150	0.42608	0.68666	0.05569	0.72014	0.94116	0.35126
hsa-miR-324-3p	1	0	1	0.83313	0.68787	0.51184	0.33316	0.34217	0.82638	0.51373	0.20192	0.92689	0.01803	0.59117	0.73417
hsa-miR-324-5p	3	1	2	0.02728	0.73453	0.77359	0.23800	0.96659	0.65024	0.16774	0.11414	0.00647	0.60496	0.02901	0.23625
hsa-miR-326	0	0	0	0.99040	0.61698	0.73901	0.90153	0.81905	0.77864	0.51546	0.29544	0.12516	0.98045	0.30565	0.33653
hsa-miR-328	3	3	0	0.02135	0.12463	0.03094	0.04575	0.05208	0.15640	0.32577	0.10130	0.49309	0.88691	0.60007	0.94293
hsa-miR-330-3p	8	2	6	0.00815	0.35105	0.26584	0.05162	0.04883	0.11482	0.11090	0.08427	0.01263	0.25360	0.01153	0.03583
hsa-miR-331-3p	1	1	0	0.69402	0.01840	0.07467	0.38557	0.05825	0.36935	0.88447	0.97083	0.51097	0.31307	0.23453	0.78397
hsa-miR-335	0	0	0	0.28908	0.97454	0.97181	0.79815	0.91394	0.72691	0.41675	0.54806	0.18176	0.86948	0.44570	0.68659
hsa-miR-335*	5	4	1	0.00750	0.17872	0.07076	0.02579	0.00098	0.09074	0.02806	0.05932	0.07751	0.25919	0.03389	0.08514
hsa-miR-337-3p	0	0	0	0.06318	0.26195	0.19479	0.09821	0.09080	0.66213	0.26938	0.37898	0.23508	0.29721	0.10913	0.39524
hsa-miR-337-5p	5	4	1	0.00943	0.09233	0.03848	0.05982	0.02866	0.08329	0.03092	0.05349	0.14014	0.10798	0.16768	0.08754
hsa-miR-338-3p	0	0	0	0.29243	0.66232	0.46102	0.77539	0.76080	0.42432	0.73248	0.80166	0.06887	0.38900	0.79770	0.60986
hsa-miR-339-3p	10	6	4	0.06271	0.10866	0.04608	0.01897	0.00165	0.02011	0.00879	0.01305	0.06957	0.00759	0.01369	0.05041
hsa-miR-33a	4	1	3	0.04523	0.89608	0.47835	0.30736	0.34600	0.60535	0.28949	0.18883	0.01877	0.81693	0.36166	0.53177
hsa-miR-33b*	0	0	0	0.79060	0.08361	0.05578	0.32926	0.72596	0.28456	0.96217	0.25885	0.27375	0.51287	0.98760	0.34360
hsa-miR-340	2	0	2	0.06669	0.63470	0.96278	0.26040	0.78751	0.52630	0.16837	0.16415	0.00707	0.95783	0.35972	0.70841
hsa-miR-340*	1	1	0	0.87339	0.14147	0.38343	0.15727	0.03459	0.63467	0.93640	0.62315	0.93266	0.16498	0.56647	0.73560
hsa-miR-342-3p	0	0	0	0.36572	0.92039	0.42652	0.32750	0.09307	0.30836	0.26506	0.56892	0.11030	0.47538	0.53715	0.68471
hsa-miR-342-5p	0	0	0	0.75660	0.33465	0.61256	0.08368	0.38874	0.97183	0.77095	0.20835	0.70343	0.73432	0.73729	0.62243
hsa-miR-34a	0	0	0	0.94015	0.65064	0.98383	0.94600	0.59091	0.45189	0.99768	0.97993	0.52468	0.63933	0.27013	0.62327
hsa-miR-34b	14	8	6	0.00008	0.01210	0.00421	0.00586	0.00147	0.02760	0.00231	0.00246	0.03095	0.00870	0.01114	0.04533
hsa-miR-3605-5p	8	5	3	0.02764	0.03094	0.03880	0.02688	0.00166	0.06018	0.71380	0.16874	0.03224	0.07862	0.05309	0.38428
hsa-miR-361-3p	4	3	1	0.66017	0.05890	0.00295	0.02903	0.21748	0.07419	0.04145	0.22078	0.90924	0.01961	0.28136	0.08253
hsa-miR-361-5p	1	0	1	0.11756	0.86295	0.89421	0.41711	0.85171	0.78869	0.43677	0.27719	0.06334	0.48555	0.15545	0.39035
hsa-miR-3610	1	1	0	0.02755	0.24776	0.18526	0.10254	0.38863	0.27175	0.26635	0.11520	0.15080	0.43080	0.14043	0.48322
hsa-miR-3613-3p	1	1	0	0.06063	0.86337	0.94705	0.55601	0.03076	0.35297	0.15555	0.77866	0.69226	0.95258	0.24832	0.63255
hsa-miR-3614-5p	0	0	0	0.43773	0.87206	0.42505	0.78836	0.28645	0.55175	0.62525	0.94151	0.48584	0.77677	0.65921	0.56895
hsa-miR-3617	16	8	8	0.00824	0.00427	0.00442	0.00329	0.00160	0.00261	0.00248	0.00653	0.00236	0.00161	0.01218	0.00296
hsa-miR-362-3p	0	0	0	0.60218	0.32654	0.96402	0.14747	0.16543	0.32805	0.38345	0.13218	0.72619	0.24647	0.90576	0.57911
hsa-miR-3620	8	8	0	0.00552	0.04751	0.04050	0.00839	0.00196	0.01634	0.01113	0.01410	0.15030	0.65147	0.25543	0.17625
hsa-miR-3621	0	0	0	0.09752	0.95532	0.23819	0.08255	0.07396	0.47958	0.46177	0.83055	0.80131	0.86092	0.24144	0.97307
hsa-miR-3622a-5p	4	3	1	0.03512	0.34198	0.17573	0.03930	0.00247	0.13519	0.09667	0.28024	0.56339	0.41323	0.10563	0.27469
hsa-miR-3622b-5p	16	8	8	0.00055	0.00028	0.00020	0.00028	0.00006	0.00037	0.00042	0.00128	0.00119	0.00039	0.01154	0.00060

hsa-miR-363	0	0	0	0.67826	0.22533	0.08640	0.22297	0.08681	0.29068	0.25769	0.17785	0.67398	0.09245	0.43776	0.22098
hsa-miR-3646	7	4	3	0.00015	0.25275	0.05027	0.05167	0.00836	0.04695	0.00977	0.05303	0.03940	0.20606	0.05619	0.11387
hsa-miR-3647-5p	15	8	7	0.00025	0.00002	0.00001	0.00009	0.00001	0.00000	0.00000	0.00022	0.00014	0.00111	0.00220	0.00002
hsa-miR-3648	10	8	2	0.03573	0.03392	0.02099	0.04759	0.01559	0.02672	0.01953	0.01994	0.26494	0.15451	0.01063	0.02754
hsa-miR-365	8	4	4	0.00776	0.54164	0.24650	0.16516	0.00246	0.00613	0.02809	0.27883	0.04545	0.34638	0.02245	0.05688
hsa-miR-3651	3	3	0	0.00710	0.56765	0.05554	0.01242	0.00611	0.27952	0.08535	0.11251	0.87472	0.63401	0.08529	0.53587
hsa-miR-3652	0	0	0	0.69216	0.85902	0.26559	0.98766	0.15027	0.51781	0.72312	0.84786	0.80938	0.25355	0.56608	0.81821
hsa-miR-3654	12	8	4	0.00115	0.00230	0.00121	0.00713	0.00033	0.00572	0.01295	0.03381	0.04265	0.00626	0.02822	0.00743
hsa-miR-3656	1	0	1	0.72444	0.16429	0.34105	0.82191	0.21372	0.18950	0.56910	0.64279	0.93826	0.00490	0.42629	0.99576
hsa-miR-3659	10	8	2	0.03017	0.00756	0.00806	0.01670	0.01918	0.03125	0.01576	0.04368	0.07903	0.33755	0.00160	0.02494
hsa-miR-3660	1	1	0	0.28010	0.49014	0.44387	0.08364	0.01601	0.74833	0.32845	0.41138	0.92436	0.92672	0.29180	0.42556
hsa-miR-3663-3p	0	0	0	0.24451	0.38920	0.48507	0.85627	0.61286	0.64382	0.98440	0.75175	0.14662	0.62519	0.09005	0.95363
hsa-miR-3663-5p	0	0	0	0.27076	0.40326	0.15901	0.56698	0.23682	0.81066	0.80189	0.88554	0.90524	0.34575	0.53922	0.92130
hsa-miR-3665	0	0	0	0.58036	0.48062	0.47702	0.93305	0.88416	0.75088	0.88382	0.63441	0.56275	0.24624	0.78176	0.70746
hsa-miR-3667-5p	0	0	0	0.96154	0.57026	0.53498	0.79162	0.26379	0.73439	0.52478	0.37654	0.33462	0.30217	0.35802	0.91742
hsa-miR-3675-3p	1	1	0	0.09138	0.48720	0.76462	0.41104	0.00667	0.36132	0.06843	0.35113	0.89870	0.73492	0.44811	0.94209
hsa-miR-3676	0	0	0	0.98903	0.49749	0.10822	0.83056	0.41537	0.79241	0.48732	0.88354	0.66073	0.91180	0.69979	0.97167
hsa-miR-3679-3p	4	3	1	0.00544	0.26513	0.19687	0.06064	0.02065	0.06643	0.04258	0.08569	0.05476	0.37620	0.05583	0.15408
hsa-miR-3679-5p	0	0	0	0.98379	0.12248	0.94853	0.71052	0.56729	0.43507	0.06149	0.21465	0.24235	0.91095	0.27357	0.44332
hsa-miR-3680*	7	6	1	0.19313	0.00039	0.00547	0.01088	0.00898	0.00221	0.02999	0.09335	0.19124	0.00009	0.73337	0.25485
hsa-miR-3682	3	3	0	0.54010	0.02047	0.01042	0.77129	0.04584	0.20932	0.25276	0.61448	0.31745	0.06055	0.17686	0.07461
hsa-miR-3692*	2	1	1	0.10358	0.08696	0.15640	0.41453	0.05742	0.40321	0.03981	0.33825	0.86602	0.64027	0.07549	0.74734
hsa-miR-370	8	4	4	0.00116	0.05559	0.01317	0.05255	0.00309	0.03023	0.08369	0.44365	0.03851	0.02852	0.02386	0.07374
hsa-miR-371-3p	0	0	0	0.35696	0.79361	0.84235	0.81536	0.54485	0.08535	0.06946	0.36769	0.24877	0.61028	0.16152	0.26391
hsa-miR-371-5p	0	0	0	0.96537	0.82642	0.55832	0.06501	0.61418	0.54422	0.10834	0.18702	0.57478	0.60713	0.15400	0.24927
hsa-miR-3713	1	0	1	0.05135	0.22992	0.09096	0.74130	0.28309	0.11545	0.43839	0.98363	0.27838	0.85052	0.05066	0.02624
hsa-miR-373*	0	0	0	0.31396	0.22823	0.80590	0.43293	0.41602	0.78331	0.85243	0.47006	0.95024	0.21533	0.86183	0.16936
hsa-miR-374a	0	0	0	0.18965	0.19298	0.23692	0.54179	0.07085	0.44797	0.98042	0.77207	0.13808	0.16577	0.88937	0.83549
hsa-miR-374b	0	0	0	0.12793	0.23391	0.61912	0.95074	0.25775	0.63935	0.52447	0.33940	0.43215	0.17172	0.39277	0.81758
hsa-miR-374c	1	0	1	0.17969	0.05557	0.13122	0.63989	0.28666	0.97252	0.76765	0.73575	0.48867	0.04608	0.72139	0.67602
hsa-miR-376a	14	7	7	0.00864	0.02230	0.01901	0.02263	0.02446	0.07611	0.01073	0.00764	0.00410	0.00992	0.03084	0.02495
hsa-miR-376b	2	1	1	0.04593	0.53102	0.16061	0.18018	0.08137	0.30621	0.15608	0.07831	0.08369	0.20389	0.46593	0.31967
hsa-miR-376c	12	5	7	0.01874	0.05219	0.03321	0.04246	0.07300	0.11956	0.02153	0.01613	0.00525	0.02533	0.01815	0.03224
hsa-miR-377	8	3	5	0.04707	0.12353	0.10801	0.12161	0.19705	0.17967	0.03540	0.02250	0.01498	0.04839	0.27106	0.09119
hsa-miR-378	5	4	1	0.00869	0.01339	0.08347	0.04073	0.00068	0.08229	0.05061	0.16037	0.48591	0.39949	0.24958	0.36669
hsa-miR-378b	8	7	1	0.01041	0.03837	0.01065	0.00760	0.00377	0.04675	0.01127	0.36580	0.18281	0.01944	0.17498	0.08592
hsa-miR-379	3	1	2	0.01846	0.31573	0.14678	0.39692	0.12272	0.26935	0.05721	0.11152	0.02500	0.16007	0.08019	0.24474
hsa-miR-381	0	0	0	0.75094	0.81772	0.97715	0.67609	0.28471	0.88426	0.30112	0.68303	0.41925	0.26864	0.95561	0.39797
hsa-miR-382	5	2	3	0.00654	0.12172	0.10923	0.33510	0.05836	0.32958	0.03303	0.06819	0.02073	0.09941	0.04428	0.12381
hsa-miR-3907	2	1	1	0.75107	0.00295	0.86893	0.40069	0.10596	0.07308	0.56831	0.79404	0.96698	0.00994	0.17620	0.41832
hsa-miR-3911	0	0	0	0.67003	0.16240	0.57824	0.40824	0.79335	0.39239	0.61914	0.31553	0.17118	0.33206	0.25429	0.24321
hsa-miR-3917	1	1	0	0.25847	0.43698	0.15739	0.22127	0.02524	0.47688	0.15032	0.35269	0.84969	0.58430	0.38501	0.97649
hsa-miR-3923	0	0	0	0.75929	0.45759	0.40503	0.97757	0.78843	0.80852	0.63601	0.38060	0.95265	0.85903	0.99935	0.97396
hsa-miR-3926	3	3	0	0.07372	0.18190	0.03012	0.00278	0.00923	0.59084	0.13184	0.06042	0.84116	0.54441	0.47047	0.50091
hsa-miR-3934	0	0	0	0.38191	0.90491	0.53985	0.74762	0.19575	0.72465	0.85398	0.72872	0.99072	0.50142	0.42311	0.64379
hsa-miR-3937	1	0	1	0.12529	0.97396	0.88214	0.27360	0.89056	0.95658	0.99859	0.28454	0.02109	0.55412	0.18474	0.36778
hsa-miR-3938	0	0	0	0.48892	0.52210	0.20952	0.25473	0.84579	0.50492	0.44866	0.08595	0.18165	0.79648	0.90167	0.93312
hsa-miR-3945	0	0	0	0.44143	0.23124	0.83801	0.91483	0.40526	0.52741	0.65281	0.47731	0.26277	0.08164	0.79114	0.42899
hsa-miR-409-3p	8	5	3	0.04495	0.03897	0.04492	0.09017	0.11233	0.12397	0.04860	0.01251	0.03111	0.06208	0.12946	0.12559
hsa-miR-410	9	3	6	0.01646	0.08417	0.06015	0.07610	0.07309	0.18518	0.01482	0.02714	0.00977	0.03365	0.10733	0.03828
hsa-miR-422a	0	0	0	0.38758	0.63017	0.98508	0.40144	0.97722	0.80042	0.73440	0.12842	0.39631	0.51625	0.07255	0.47777
hsa-miR-423-3p	1	0	1	0.17482	0.39061	0.23155	0.40432	0.12039	0.36397	0.12202	0.35964	0.06369	0.69077	0.09011	0.38328
hsa-miR-423-5p	1	1	0	0.61032	0.18708	0.05787	0.00379	0.25815	0.06782	0.49847	0.05046	0.74452	0.09274	0.45229	0.19613
hsa-miR-424	0	0	0	0.22222	0.83227	0.67127	0.22088	0.33444	0.86637	0.66755	0.35655	0.63423	0.25565	0.58945	0.31766
hsa-miR-425	0	0	0	0.64506	0.15305	0.07124	0.49919	0.23399	0.06525	0.69714	0.75982	0.28804	0.95987	0.27440	0.99978
hsa-miR-425*	0	0	0	0.68696	0.42771	0.15455	0.88817	0.40931	0.68637	0.52111	0.71005	0.80507	0.78852	0.75003	0.83883
hsa-miR-4253	3	3	0	0.79362	0.00357	0.01217	0.41382	0.01384	0.06731	0.09334	0.39075	0.80015	0.59140	0.32931	0.56785

hsa-miR-4257	2	2	0	0.91309	0.01665	0.00743	0.63130	0.28273	0.14044	0.71841	0.95959	0.65147	0.92661	0.19038	0.36175
hsa-miR-4259	12	8	4	0.00053	0.00028	0.00030	0.00054	0.00007	0.00134	0.00056	0.00496	0.20345	0.01073	0.40809	0.11574
hsa-miR-4261	15	8	7	0.00002	0.00000	0.00001	0.00001	0.00000	0.00001	0.00036	0.00000	0.00259	0.00000	0.00547	0.02703
hsa-miR-4265	0	0	0	0.30152	0.89160	0.81193	0.31577	0.14777	0.35983	0.24385	0.58169	0.14162	0.36454	0.25164	0.13493
hsa-miR-4269	0	0	0	0.37852	0.79449	0.37252	0.74910	0.26992	0.69874	0.44408	0.64095	0.98488	0.97018	0.49531	0.91196
hsa-miR-4270	0	0	0	0.58343	0.68289	0.79955	0.52776	0.19055	0.70012	0.59501	0.46858	0.15179	0.28915	0.56296	0.80585
hsa-miR-4271	0	0	0	0.83936	0.24413	0.11153	0.40954	0.10439	0.70329	0.94009	0.36477	0.13805	0.83431	0.76746	0.46994
hsa-miR-4274	0	0	0	0.13920	0.24275	0.13250	0.17063	0.30967	0.21266	0.30607	0.05921	0.18514	0.16251	0.05315	0.07134
hsa-miR-4281	4	3	1	0.03501	0.07090	0.03834	0.09576	0.05309	0.03754	0.09181	0.16334	0.04567	0.14752	0.17949	0.40568
hsa-miR-4284	0	0	0	0.97316	0.69795	0.26554	0.40220	0.41615	0.81529	0.93571	0.31843	0.93006	0.26243	0.52712	0.50771
hsa-miR-4286	8	7	1	0.04648	0.01208	0.05683	0.00039	0.00220	0.01689	0.02689	0.03278	0.21935	0.22147	0.02985	0.22208
hsa-miR-4290	13	8	5	0.00000	0.00822	0.00506	0.00008	0.00007	0.00060	0.00028	0.00228	0.07549	0.09429	0.00691	0.04450
hsa-miR-4291	2	0	2	0.35435	0.49649	0.51693	0.29150	0.31365	0.83610	0.59810	0.18640	0.01883	0.76745	0.92237	0.95811
hsa-miR-4294	9	2	7	0.14170	0.07702	0.06836	0.05855	0.03648	0.04170	0.08732	0.07206	0.06873	0.03486	0.04090	0.02723
hsa-miR-4297	10	7	3	0.00037	0.01843	0.01445	0.00476	0.05639	0.00791	0.03392	0.00858	0.12813	0.11930	0.15537	0.05849
hsa-miR-4298	0	0	0	0.39944	0.62421	0.30374	0.53835	0.12729	0.75547	0.74566	0.71440	0.07783	0.79616	0.82350	0.73134
hsa-miR-4299	7	5	2	0.00099	0.00419	0.05332	0.10453	0.02575	0.00320	0.02080	0.05932	0.00938	0.08530	0.07208	0.19202
hsa-miR-4306	1	0	1	0.94087	0.34455	0.47991	0.73353	0.34514	0.62262	0.15090	0.48901	0.27117	0.97170	0.20047	0.97033
hsa-miR-431	4	1	3	0.01988	0.55352	0.35689	0.13936	0.18240	0.15520	0.06940	0.05689	0.03044	0.20340	0.09898	0.07420
hsa-miR-4310	0	0	0	0.89697	0.20399	0.17736	0.42725	0.60111	0.38673	0.72748	0.65285	0.46345	0.66620	0.89646	0.52549
hsa-miR-4312	1	1	0	0.06144	0.60107	0.82213	0.09737	0.03173	0.20865	0.06884	0.17400	0.43689	0.70824	0.38427	0.47525
hsa-miR-4313	0	0	0	0.74009	0.33041	0.21497	0.61056	0.40404	0.58083	0.48050	0.63700	0.30573	0.73478	0.67409	0.72219
hsa-miR-4314	1	0	1	0.15129	0.70616	0.08672	0.88406	0.05771	0.19232	0.26924	0.87158	0.45753	0.04919	0.07758	0.13909
hsa-miR-4317	1	0	1	0.15673	0.97498	0.78775	0.27100	0.18291	0.80307	0.33661	0.20726	0.28957	0.58267	0.22899	0.74692
hsa-miR-432	16	8	8	0.00052	0.00348	0.00049	0.00172	0.00060	0.00052	0.00062	0.00052	0.00481	0.00240	0.00965	0.01052
hsa-miR-4322	0	0	0	0.34206	0.73065	0.51019	0.96524	0.27845	0.42257	0.50051	0.78728	0.89478	0.54669	0.30412	0.90048
hsa-miR-4323	1	1	0	0.26002	0.99828	0.71585	0.55080	0.04729	0.32875	0.14778	0.26442	0.74775	0.95105	0.34519	0.93822
hsa-miR-4327	2	1	1	0.02379	0.20661	0.10199	0.11095	0.94027	0.05934	0.31448	0.21585	0.01039	0.32358	0.06199	0.08705
hsa-miR-449b*	10	6	4	0.00020	0.07849	0.01134	0.00120	0.00544	0.09317	0.00530	0.01508	0.15060	0.01798	0.00254	0.01744
hsa-miR-449c*	7	5	2	0.00079	0.05699	0.02683	0.03475	0.17831	0.00937	0.05915	0.01420	0.11975	0.75657	0.13674	0.15352
hsa-miR-451	0	0	0	0.21395	0.45810	0.73165	0.58143	0.81917	0.73022	0.97113	0.93186	0.61537	0.91034	0.47246	0.88776
hsa-miR-454	1	1	0	0.03262	0.57155	0.27451	0.61446	0.44424	0.56697	0.81770	0.78709	0.27088	0.05399	0.67509	0.34069
hsa-miR-483-3p	13	8	5	0.00010	0.01113	0.00775	0.00027	0.00030	0.00063	0.00023	0.00348	0.07938	0.12069	0.00848	0.04409
hsa-miR-483-5p	0	0	0	0.98689	0.97796	0.25019	0.30142	0.05053	0.83213	0.38841	0.47289	0.29444	0.83064	0.56424	0.97620
hsa-miR-484	1	1	0	0.46685	0.18538	0.19509	0.04255	0.55602	0.43015	0.76681	0.92434	0.06183	0.06605	0.91881	0.38434
hsa-miR-485-3p	2	2	0	0.01824	0.12591	0.03474	0.05884	0.11228	0.14402	0.13753	0.17861	0.64644	0.65989	0.35160	0.68741
hsa-miR-486-3p	0	0	0	0.23813	0.52972	0.70503	0.88801	0.81375	0.71337	0.30895	0.77348	0.42107	0.17329	0.98710	0.95780
hsa-miR-486-5p	4	3	1	0.12075	0.39077	0.05347	0.00772	0.04312	0.09665	0.13226	0.03735	0.95549	0.37468	0.27768	0.04370
hsa-miR-487b	4	4	0	0.02317	0.12481	0.08404	0.05098	0.04801	0.07459	0.04192	0.02485	0.19810	0.27496	0.21518	0.20851
hsa-miR-493*	9	7	2	0.00645	0.07579	0.00907	0.04206	0.00663	0.00677	0.01166	0.02685	0.02364	0.08310	0.05321	0.05848
hsa-miR-494	1	1	0	0.80814	0.30312	0.50297	0.08533	0.75026	0.35647	0.15705	0.03874	0.77355	0.25541	0.39682	0.15168
hsa-miR-495	7	5	2	0.02866	0.13261	0.05004	0.02442	0.09439	0.03190	0.01454	0.00855	0.03980	0.03556	0.23600	0.13080
hsa-miR-497	1	0	1	0.17866	0.15107	0.09266	0.12952	0.13597	0.05296	0.07650	0.17367	0.00784	0.07000	0.41197	0.43498
hsa-miR-498	3	1	2	0.00375	0.35283	0.72942	0.07801	0.50804	0.22924	0.21620	0.15787	0.03650	0.38982	0.07441	0.09037
hsa-miR-500a	15	8	7	0.00190	0.00100	0.00142	0.00221	0.00033	0.00115	0.00204	0.01401	0.00332	0.00031	0.00510	0.00066
hsa-miR-500a*	0	0	0	0.81121	0.77543	0.13628	0.76000	0.70093	0.52252	0.93754	0.45756	0.43544	0.54069	0.65430	0.09321
hsa-miR-501-3p	2	1	1	0.37623	0.54977	0.53071	0.38955	0.02302	0.36850	0.16461	0.41126	0.24078	0.84689	0.03823	0.67749
hsa-miR-501-5p	13	7	6	0.00108	0.00071	0.00075	0.03026	0.00298	0.00231	0.00200	0.06226	0.00366	0.00115	0.00500	0.00768
hsa-miR-502-3p	1	0	1	0.12164	0.57403	0.67855	0.48111	0.07511	0.85990	0.24013	0.46080	0.04440	0.73732	0.15812	0.59434
hsa-miR-505	2	1	1	0.73007	0.02149	0.25779	0.88572	0.13221	0.18431	0.86062	0.94651	0.80673	0.00482	0.15090	0.17471
hsa-miR-505*	0	0	0	0.10265	0.63451	0.84064	0.58805	0.48505	0.93705	0.30487	0.59317	0.40977	0.31296	0.40417	0.91153
hsa-miR-513a-3p	0	0	0	0.99807	0.97806	0.52921	0.62152	0.12895	0.22857	0.36360	0.91056	0.24054	0.58563	0.27381	0.68035
hsa-miR-513a-5p	11	7	4	0.10922	0.02991	0.00814	0.00210	0.00520	0.00520	0.04045	0.04551	0.19252	0.02369	0.14094	0.02219
hsa-miR-513b	4	4	0	0.19546	0.00905	0.13899	0.05779	0.45688	0.02017	0.00441	0.00325	0.90827	0.16115	0.73984	0.29529
hsa-miR-514b-5p	0	0	0	0.08167	0.89178	0.07723	0.21500	0.07253	0.56104	0.11978	0.39104	0.48135	0.59234	0.51413	0.91744
hsa-miR-516a-5p	1	0	1	0.11772	0.13371	0.06110	0.52606	0.33014	0.10405	0.29789	0.71673	0.52552	0.93367	0.29402	0.03182
hsa-miR-516b	5	4	1	0.01409	0.11506	0.02323	0.06770	0.00531	0.05083	0.04751	0.14023	0.47537	0.38239	0.07460	0.19820

hsa-miR-518e*	11	8	3	0.00249	0.00019	0.00085	0.00481	0.00119	0.00168	0.00552	0.00518	0.00214	0.09877	0.04235	0.57509
hsa-miR-520b	0	0	0	0.97947	0.96676	0.36033	0.89785	0.83862	0.64245	0.67874	0.51643	0.81782	0.54758	0.77686	0.76274
hsa-miR-520e	0	0	0	0.25225	0.11986	0.12069	0.20087	0.42921	0.57480	0.23379	0.29823	0.22962	0.30267	0.25880	0.86489
hsa-miR-526b	7	6	1	0.00551	0.00136	0.00025	0.01067	0.00567	0.21291	0.07499	0.03555	0.92193	0.92232	0.69318	0.64350
hsa-miR-532-3p	3	2	1	0.73311	0.01957	0.00415	0.25352	0.20563	0.27292	0.41332	0.72247	0.64132	0.04361	0.85515	0.50046
hsa-miR-532-5p	0	0	0	0.62749	0.63005	0.17449	0.98280	0.76814	0.64854	0.95673	0.69417	0.11935	0.28184	0.69400	0.46376
hsa-miR-539	5	5	0	0.18001	0.14576	0.28072	0.00246	0.02578	0.01391	0.03617	0.00143	0.63353	0.20832	0.37609	0.32664
hsa-miR-542-5p	3	3	0	0.02281	0.54883	0.28365	0.04504	0.00364	0.39595	0.20716	0.34774	0.56356	0.42954	0.25779	0.37980
hsa-miR-543	2	2	0	0.06288	0.32939	0.21158	0.11854	0.48907	0.03500	0.16218	0.00779	0.54247	0.12717	0.67718	0.13048
hsa-miR-548c-5p	0	0	0	0.33640	0.63362	0.61647	0.94927	0.66347	0.93392	0.69778	0.53780	0.85585	0.06966	0.43876	0.66833
hsa-miR-548q	5	3	2	0.02267	0.00540	0.52585	0.06124	0.21827	0.08620	0.15191	0.02772	0.16723	0.12655	0.52558	0.04183
hsa-miR-550a	4	3	1	0.16619	0.03573	0.01929	0.04506	0.51887	0.12158	0.29399	0.05520	0.03678	0.39553	0.33358	0.12136
hsa-miR-550a*	1	1	0	0.51338	0.06553	0.01680	0.30722	0.10144	0.26237	0.47237	0.40319	0.72117	0.09138	0.70686	0.63585
hsa-miR-551b	0	0	0	0.66835	0.89209	0.16975	0.60537	0.20267	0.86916	0.45037	0.37769	0.19910	0.75585	0.22259	0.17086
hsa-miR-551b*	8	7	1	0.00225	0.02909	0.04576	0.01662	0.00272	0.02897	0.02001	0.05461	0.14870	0.06383	0.01028	0.13439
hsa-miR-557	0	0	0	0.42874	0.61543	0.30431	0.36975	0.83045	0.41094	0.70822	0.67305	0.24493	0.13802	0.79242	0.23011
hsa-miR-563	0	0	0	0.80201	0.24730	0.08356	0.62899	0.49951	0.44132	0.93082	0.45000	0.28365	0.74733	0.68606	0.57904
hsa-miR-564	0	0	0	0.55352	0.95925	0.86643	0.91880	0.12688	0.76629	0.55220	0.50586	0.27113	0.23628	0.84318	0.50789
hsa-miR-566	1	0	1	0.13633	0.09357	0.75913	0.68024	0.46351	0.15040	0.95323	0.22366	0.06166	0.01305	0.21055	0.15473
hsa-miR-572	1	0	1	0.16213	0.98081	0.75112	0.70591	0.53751	0.69188	0.27505	0.47925	0.93819	0.03443	0.58703	0.31522
hsa-miR-574-3p	11	8	3	0.00042	0.00837	0.00297	0.02206	0.00026	0.01075	0.01229	0.03480	0.03983	0.06422	0.00039	0.07295
hsa-miR-574-5p	0	0	0	0.73182	0.31054	0.41156	0.61923	0.87559	0.99676	0.93778	0.82055	0.59744	0.43944	0.74564	0.62959
hsa-miR-575	2	1	1	0.16180	0.04223	0.38144	0.05234	0.49618	0.23721	0.11673	0.09856	0.38985	0.10683	0.14529	0.39467
hsa-miR-582-5p	0	0	0	0.36332	0.37102	0.58046	0.07596	0.52367	0.95800	0.83603	0.35644	0.33573	0.16910	0.42708	0.65210
hsa-miR-583	3	1	2	0.20178	0.17332	0.02654	0.38900	0.05475	0.05213	0.10509	0.60309	0.39770	0.03206	0.05639	0.02690
hsa-miR-584	1	1	0	0.29680	0.01802	0.08393	0.68213	0.14604	0.05224	0.14307	0.12595	0.25408	0.16223	0.88166	0.55285
hsa-miR-590-5p	10	6	4	0.00360	0.06671	0.12718	0.01762	0.01257	0.01637	0.00245	0.01019	0.00055	0.45397	0.00642	0.38975
hsa-miR-595	0	0	0	0.48811	0.78614	0.36575	0.09659	0.16143	0.17676	0.24749	0.09275	0.46621	0.27999	0.31261	0.52699
hsa-miR-598	3	2	1	0.03023	0.93379	0.61173	0.10789	0.11166	0.07462	0.20772	0.02543	0.63090	0.13302	0.55197	0.43733
hsa-miR-601	1	0	1	0.57931	0.31856	0.06120	0.88837	0.11405	0.06040	0.09964	0.49349	0.51553	0.69343	0.01895	0.46277
hsa-miR-602	2	2	0	0.86192	0.04314	0.02477	0.51844	0.74312	0.26127	0.98417	0.19081	0.06242	0.53660	0.96169	0.40128
hsa-miR-605	12	8	4	0.00018	0.00156	0.04235	0.01448	0.00078	0.00564	0.00046	0.00285	0.04982	0.06752	0.19081	0.01675
hsa-miR-609	4	4	0	0.00270	0.10590	0.08126	0.04323	0.20812	0.03544	0.05157	0.01574	0.40783	0.41962	0.37462	0.18436
hsa-miR-610	0	0	0	0.10173	0.37174	0.30590	0.58158	0.05765	0.41028	0.37584	0.56406	0.50507	0.29041	0.14057	0.87219
hsa-miR-615-3p	6	6	0	0.00471	0.09468	0.01989	0.00511	0.00171	0.07636	0.04038	0.02994	0.11756	0.22269	0.29630	0.05075
hsa-miR-617	0	0	0	0.21869	0.08759	0.48867	0.33931	0.71635	0.84570	0.39992	0.08415	0.86796	0.08857	0.82139	0.35367
hsa-miR-622	8	7	1	0.03671	0.01173	0.00714	0.08283	0.00795	0.03524	0.02049	0.02711	0.64548	0.24392	0.02465	0.08143
hsa-miR-623	1	1	0	0.48171	0.10566	0.65467	0.19165	0.30722	0.73374	0.24003	0.04784	0.77401	0.99535	0.81017	0.91191
hsa-miR-625	4	1	3	0.02694	0.75149	0.47145	0.11361	0.22539	0.08748	0.05225	0.06441	0.02942	0.34628	0.06054	0.14638
hsa-miR-625*	0	0	0	0.71867	0.93214	0.32277	0.39126	0.32815	0.48066	0.34755	0.51329	0.57914	0.65691	0.53427	0.99618
hsa-miR-627	6	3	3	0.00503	0.03556	0.55123	0.15098	0.00128	0.18797	0.09571	0.24084	0.00885	0.31104	0.12205	0.99259
hsa-miR-629	1	1	0	0.10677	0.99626	0.97999	0.21923	0.01672	0.93309	0.30927	0.64738	0.72026	0.33024	0.10283	0.43709
hsa-miR-629*	2	1	1	0.84010	0.50671	0.12612	0.49203	0.44203	0.27575	0.65191	0.03281	0.02132	0.72671	0.62810	0.18599
hsa-miR-630	2	0	2	0.22338	0.26417	0.14129	0.48503	0.71011	0.12566	0.89144	0.94922	0.26886	0.00087	0.39009	0.00917
hsa-miR-631	0	0	0	0.11132	0.92751	0.33166	0.80138	0.26677	0.78385	0.72977	0.61812	0.33005	0.25878	0.09442	0.45090
hsa-miR-634	6	6	0	0.01235	0.02953	0.17883	0.00466	0.00478	0.07945	0.00992	0.03653	0.07017	0.49286	0.25007	0.24182
hsa-miR-636	0	0	0	0.23851	0.49157	0.79534	0.38796	0.30492	0.78221	0.18496	0.35150	0.72871	0.87471	0.92918	0.70201
hsa-miR-638	0	0	0	0.27802	0.59581	0.65280	0.92768	0.96848	0.62480	0.53698	0.74665	0.86825	0.05518	0.51057	0.37610
hsa-miR-642b	0	0	0	0.27723	0.68872	0.97896	0.46904	0.32202	0.99678	0.40947	0.57789	0.32247	0.42312	0.93838	0.57213
hsa-miR-648	12	8	4	0.00083	0.00705	0.01014	0.00849	0.00024	0.00545	0.00513	0.01166	0.06810	0.00281	0.00069	0.00994
hsa-miR-652	6	5	1	0.04976	0.00123	0.00387	0.05626	0.00216	0.05154	0.03569	0.05710	0.41336	0.03904	0.29769	0.16710
hsa-miR-654-3p	0	0	0	0.94116	0.57112	0.39364	0.79812	0.53239	0.75614	0.31571	0.51467	0.26210	0.60212	0.66453	0.88850
hsa-miR-654-5p	0	0	0	0.07323	0.16463	0.21857	0.57719	0.08496	0.69607	0.95827	0.89822	0.62764	0.39935	0.31267	0.86427
hsa-miR-659	0	0	0	0.11205	0.76288	0.84876	0.61108	0.25271	0.94321	0.35938	0.80494	0.67398	0.32737	0.49825	0.89213
hsa-miR-660	0	0	0	0.52791	0.37714	0.63176	0.65672	0.38870	0.80902	0.32794	0.55238	0.13129	0.33906	0.67150	0.44951
hsa-miR-663	3	0	3	0.87183	0.08790	0.06275	0.47137	0.25320	0.13297	0.52870	0.44628	0.42522	0.04826	0.03617	0.03305
hsa-miR-663b	0	0	0	0.41676	0.55107	0.17486	0.60120	0.07192	0.94366	0.23577	0.80858	0.71130	0.05383	0.73636	0.95898

hsa-miR-664	2	2	0	0.10614	0.27573	0.11517	0.15274	0.00443	0.25367	0.01301	0.05439	0.40622	0.62963	0.12913	0.40198
hsa-miR-665	0	0	0	0.80124	0.41600	0.15928	0.88163	0.78595	0.88361	0.77582	0.99508	0.67442	0.68700	0.88740	0.79337
hsa-miR-670	1	1	0	0.67810	0.63819	0.26725	0.08595	0.23266	0.07638	0.14099	0.02262	0.59046	0.16828	0.21612	0.31755
hsa-miR-671-3p	5	4	1	0.20922	0.01099	0.02510	0.02594	0.44612	0.18482	0.31317	0.03766	0.02116	0.30870	0.18046	0.17848
hsa-miR-671-5p	0	0	0	0.10719	0.37309	0.28669	0.78121	0.50348	0.70818	0.27836	0.84493	0.08052	0.85786	0.21657	0.34030
hsa-miR-7	1	0	1	0.79651	0.24404	0.10471	0.05236	0.50465	0.43111	0.27307	0.16538	0.64173	0.03670	0.28402	0.17870
hsa-miR-7-1*	1	0	1	0.17255	0.41545	0.69947	0.06375	0.50257	0.69132	0.52787	0.12201	0.84915	0.16254	0.50533	0.50171
hsa-miR-708	0	0	0	0.49553	0.88843	0.83954	0.30682	0.26974	0.75307	0.45758	0.96557	0.82001	0.49366	0.45192	0.62032
hsa-miR-711	3	2	1	0.33525	0.37282	0.06694	0.10580	0.02258	0.58655	0.04780	0.50410	0.97168	0.70215	0.13747	0.72925
hsa-miR-718	1	0	1	0.30411	0.47919	0.66120	0.87739	0.13949	0.78829	0.41668	0.55869	0.02437	0.70273	0.54066	0.62240
hsa-miR-720	3	3	0	0.09393	0.16679	0.37105	0.03501	0.00990	0.09549	0.04058	0.12050	0.78305	0.51111	0.12493	0.65594
hsa-miR-744	0	0	0	0.38599	0.17577	0.69080	0.84893	0.95956	0.58432	0.87842	0.42189	0.27446	0.39580	0.10284	0.61364
hsa-miR-758	0	0	0	0.70294	0.89401	0.46036	0.83120	0.13337	0.97348	0.61868	0.50485	0.70987	0.31167	0.64484	0.34991
hsa-miR-760	9	5	4	0.06771	0.00519	0.02672	0.22040	0.00737	0.01310	0.12447	0.04667	0.02606	0.01378	0.04329	0.07512
hsa-miR-762	0	0	0	0.93111	0.36207	0.58194	0.60170	0.12295	0.36523	0.05764	0.35162	0.37968	0.45905	0.67746	0.80962
hsa-miR-764	12	8	4	0.00031	0.00860	0.00458	0.00364	0.00070	0.00122	0.00071	0.01228	0.06448	0.02388	0.00364	0.09268
hsa-miR-765	0	0	0	0.90111	0.71563	0.60651	0.87452	0.11565	0.85357	0.64590	0.96827	0.44561	0.97836	0.23902	0.87513
hsa-miR-766	5	5	0	0.04392	0.09361	0.05386	0.02913	0.00227	0.03822	0.02271	0.16040	0.47628	0.47176	0.28985	0.61365
hsa-miR-769-3p	0	0	0	0.36735	0.95421	0.77090	0.78766	0.44685	0.89801	0.19386	0.88440	0.42125	0.22202	0.33028	0.59861
hsa-miR-769-5p	0	0	0	0.66159	0.34418	0.70010	0.67366	0.39913	0.45035	0.61315	0.60507	0.31690	0.67501	0.87457	0.99858
hsa-miR-874	0	0	0	0.30197	0.48333	0.54459	0.99428	0.17265	0.57163	0.32744	0.58850	0.99677	0.48351	0.83174	0.51342
hsa-miR-877	0	0	0	0.37331	0.45477	0.22954	0.97610	0.11759	0.08743	0.18276	0.92538	0.73743	0.68197	0.05724	0.53806
hsa-miR-877*	6	4	2	0.02098	0.09315	0.17270	0.05848	0.00392	0.02306	0.02360	0.05948	0.14832	0.34411	0.04631	0.26777
hsa-miR-885-5p	11	7	4	0.00032	0.04690	0.02314	0.00101	0.00113	0.05401	0.00082	0.01845	0.14310	0.08380	0.02990	0.03181
hsa-miR-887	0	0	0	0.38893	0.63614	0.57046	0.49516	0.15554	0.53444	0.87806	0.52336	0.82202	0.45328	0.59758	0.71360
hsa-miR-892b	8	3	5	0.05224	0.05492	0.07455	0.23235	0.03325	0.00390	0.02139	0.19098	0.01322	0.00307	0.00544	0.00619
hsa-miR-92a	1	1	0	0.15318	0.82147	0.19307	0.04776	0.16438	0.20315	0.30183	0.12677	0.59036	0.63731	0.51935	0.10743
hsa-miR-92b	0	0	0	0.58892	0.83114	0.94453	0.64055	0.33316	0.58111	0.32732	0.79158	0.42307	0.61837	0.34488	0.34976
hsa-miR-93	0	0	0	0.81499	0.79165	0.49034	0.67771	0.46228	0.41137	0.90031	0.83161	0.29569	0.75471	0.42867	0.66642
hsa-miR-933	0	0	0	0.57499	0.34298	0.40470	0.94764	0.19591	0.71509	0.26323	0.91298	0.18606	0.83544	0.45893	0.72709
hsa-miR-936	2	2	0	0.05365	0.77603	0.10729	0.23179	0.01450	0.17849	0.04133	0.99534	0.92607	0.57417	0.58585	0.67766
hsa-miR-937	9	6	3	0.00108	0.04505	0.04226	0.05353	0.01919	0.09376	0.02103	0.01687	0.08038	0.47072	0.04915	0.04070
hsa-miR-939	0	0	0	0.39664	0.49566	0.70676	0.29717	0.71974	0.80206	0.12988	0.27523	0.45622	0.81077	0.43255	0.35575
hsa-miR-940	1	0	1	0.54787	0.05261	0.08753	0.30683	0.81262	0.10569	0.98087	0.79976	0.63569	0.01896	0.20176	0.45771
hsa-miR-96	5	1	4	0.07756	0.19506	0.05837	0.06162	0.04879	0.35898	0.21496	0.13585	0.83410	0.04639	0.01711	0.04235
hsa-miR-98	1	1	0	0.00441	0.44463	0.79312	0.66783	0.94047	0.56951	0.42578	0.35483	0.30597	0.11963	0.50102	0.98672
hsa-miR-99a	1	1	0	0.10484	0.33255	0.14001	0.83999	0.09945	0.03899	0.24732	0.28281	0.12581	0.98264	0.32667	0.64539
hsa-miR-99b	1	0	1	0.18495	0.85391	0.66651	0.77381	0.76508	0.59880	0.65340	0.69353	0.07392	0.57117	0.12504	0.23999

Q	R	S	T	U	V	W	X	Y	Z	AA	AB	AC	AD	AE	AF	AG	AH	AI	AJ
**tastases**				**without metastases**								**with metastases**							
**non-cancer control vs TP5 p-value**	**non-cancer control vs TP6 p-value**	**non-cancer control vs TP7 p-value**	**non-cancer control vs TP8 p-value**	**TP1 direction**	**TP2 direction**	**TP3 direction**	**TP4 direction**	**TP5 direction**	**TP6 direction**	**TP7 direction**	**TP8 direction**	**TP1 direction**	**TP2 direction**	**TP3 direction**	**TP4 direction**	**TP5 direction**	**TP6 direction**	**TP7 direction**	**TP8 direction**
0.25668	0.74022	0.34401	0.25141	2	1	1	1	1	2	2	2	1	1	2	2	2	2	2	2
0.37105	0.75816	0.50863	0.95835	2	2	1	2	1	1	2	2	2	1	1	1	2	1	2	1
0.13633	0.26524	0.92629	0.60605	1	2	1	1	1	1	1	1	1	1	1	1	1	1	1	1
0.45253	0.84301	0.57374	0.98660	2	1	1	1	1	1	2	1	2	1	2	1	2	1	2	2
0.18369	0.54100	0.21489	0.14141	2	1	1	2	1	1	2	2	2	1	2	2	2	2	2	2
0.65674	0.29789	0.82837	0.17175	1	1	1	1	1	1	2	1	1	2	2	2	1	2	2	2
0.09083	0.12697	0.11107	0.02934	2	2	2	2	2	2	2	2	2	2	2	2	2	2	2	2
0.16968	0.47010	0.29311	0.10145	2	1	1	1	1	2	2	2	2	1	2	2	2	2	2	2
0.09022	0.20183	0.97943	0.38423	1	1	2	1	1	1	1	1	1	1	1	1	1	1	2	1
0.29027	0.90139	0.50883	0.46259	2	2	1	2	1	2	2	2	2	1	2	2	2	2	2	2
0.28604	0.47257	0.45294	0.30557	2	2	1	2	1	1	2	2	2	1	2	2	2	2	2	2
0.01028	0.00581	0.00371	0.00181	2	2	2	2	2	2	2	2	2	2	2	2	2	2	2	2
0.34872	0.09379	0.10892	0.23694	1	2	1	1	2	1	1	1	1	1	1	1	1	1	1	1
0.92486	0.94502	0.79308	0.88579	2	1	1	1	1	1	1	1	2	1	1	1	1	1	1	1
0.34615	0.65758	0.43936	0.29178	2	1	1	1	1	1	2	2	2	1	2	2	2	2	2	2
0.57837	0.97754	0.69082	0.31931	2	1	1	1	1	1	1	1	2	2	2	1	1	1	1	1
0.33287	0.47737	0.27603	0.15830	2	2	1	1	1	2	2	2	2	1	2	2	2	2	2	2
0.45750	0.45162	0.15303	0.99250	2	2	1	1	1	2	2	2	1	1	1	1	1	1	2	2
0.05348	0.14084	0.68080	0.09777	1	1	1	1	1	2	1	1	1	1	1	1	1	1	1	1
0.74964	0.87036	0.75783	0.89317	2	2	2	2	2	2	2	2	2	1	2	2	1	2	1	1
0.18943	0.77584	0.36716	0.74237	2	2	1	1	2	1	1	1	1	1	2	1	2	1	1	1
0.13878	0.58243	0.60731	0.25023	1	1	1	1	1	1	1	1	1	1	1	1	1	1	1	1
0.54419	0.53926	0.30990	0.99580	2	1	1	1	2	1	2	1	1	1	2	1	1	2	1	1
0.98948	0.94153	0.20775	0.73155	2	2	2	1	2	2	2	2	2	2	2	2	1	2	2	1
0.48247	0.99411	0.77080	0.33918	2	2	2	2	2	2	2	2	2	2	2	2	1	2	2	1
0.82206	0.66555	0.34329	0.26233	1	2	2	1	2	1	1	1	1	1	2	1	1	1	1	1
0.00590	0.00388	0.09193	0.00621	2	2	2	2	2	2	2	2	2	2	2	2	2	2	2	2
0.50807	0.18791	0.04988	0.03463	2	2	2	2	2	2	2	2	2	2	2	2	2	2	2	2
0.13202	0.36201	0.04716	0.00651	1	1	1	1	1	1	1	1	1	1	1	1	1	1	1	1
0.74907	0.54273	0.71371	0.97566	2	2	2	2	2	2	2	2	1	1	2	2	1	2	1	1
0.22138	0.77691	0.90131	0.64969	1	2	2	1	1	2	1	2	1	1	1	1	1	2	1	1
0.56818	0.30628	0.09000	0.56607	1	1	1	2	2	1	2	1	1	1	1	1	1	1	1	1
0.88877	0.46609	0.43439	0.66769	2	2	2	1	2	1	2	2	1	1	2	2	1	2	1	2
0.06935	0.17501	0.50592	0.21440	1	1	1	1	1	1	1	1	1	1	1	1	1	1	1	1
0.12974	0.17976	0.57758	0.43906	1	2	2	1	1	1	1	1	1	1	1	1	1	1	1	1
0.26087	0.90843	0.84347	0.44346	2	1	1	1	2	1	1	1	2	1	2	1	2	1	1	1
0.20114	0.75397	0.72087	0.98014	1	1	1	1	1	2	1	1	2	1	1	1	1	1	2	1
0.20837	0.47301	0.99317	0.77362	1	2	2	1	1	2	1	1	1	1	1	1	1	1	2	1
0.08962	0.65351	0.23210	0.10274	1	1	1	1	1	1	1	1	1	1	1	1	1	1	1	1
0.46379	0.74816	0.70402	0.79431	1	2	2	1	1	1	1	2	2	1	1	1	1	1	2	1
0.31709	0.69798	0.71820	0.70270	1	2	2	1	1	2	1	1	2	2	1	1	1	1	2	1
0.00237	0.00919	0.09097	0.07125	2	2	2	2	2	2	2	2	2	2	2	2	2	2	2	2
0.20365	0.56580	0.53289	0.54024	2	2	2	2	2	2	2	2	1	2	2	1	2	2	2	2
0.13435	0.13000	0.07487	0.16943	1	1	1	1	1	1	1	1	1	1	1	1	1	1	1	1
0.02716	0.01444	0.04834	0.10779	2	2	2	2	2	2	2	2	2	2	2	2	2	2	2	2
0.06246	0.08340	0.01886	0.07092	1	1	1	1	1	1	1	1	1	1	1	1	1	1	1	1
0.19511	0.44411	0.22276	0.08662	2	1	1	2	2	2	2	2	2	1	2	2	2	2	2	2
0.52506	0.86596	0.70387	0.76714	2	1	1	2	2	2	2	2	2	1	2	1	2	1	2	2

0.44652	0.55641	0.28639	0.33915	2	1	1	1	2	1	1	1	1	1	2	1	2	1	1	2
0.18156	0.54302	0.95156	0.09761	2	1	1	2	1	2	2	2	2	1	2	2	2	2	2	2
0.67407	0.49466	0.26963	0.03229	2	2	2	2	2	2	2	2	2	1	2	2	2	2	2	2
0.01669	0.00965	0.13476	0.15971	1	1	1	1	1	1	1	1	1	1	1	1	1	1	1	1
0.00747	0.04996	0.43068	0.00669	1	1	1	1	1	1	1	1	1	1	1	1	1	1	1	1
0.27857	0.37056	0.51578	0.68353	1	2	2	2	1	2	1	2	2	2	1	2	1	1	2	2
0.81027	0.70579	0.50290	0.53441	1	2	2	2	2	2	2	2	1	1	1	2	1	2	1	1
0.18791	0.44573	0.72835	0.25174	2	1	2	1	1	2	2	2	2	1	1	2	2	2	1	2
0.53816	0.09763	0.75976	0.50850	2	1	2	2	2	2	2	2	2	2	2	2	2	2	2	2
0.96479	0.12083	0.79490	0.87777	2	2	2	2	2	2	2	2	2	2	2	2	2	2	1	2
0.64745	0.18066	0.58854	0.75107	1	1	1	1	1	1	1	1	1	1	1	1	1	1	1	2
0.03283	0.14545	0.33031	0.23915	1	1	1	1	1	1	1	1	1	1	1	1	1	1	1	1
0.56023	0.53738	0.32619	0.83529	2	1	1	2	2	1	2	1	1	1	2	1	1	1	1	1
0.01979	0.13368	0.11119	0.02125	2	2	2	2	2	2	2	2	2	1	2	2	2	2	2	2
0.09487	0.11767	0.79142	0.38361	1	1	1	1	1	1	1	1	1	1	1	1	1	1	1	1
0.05060	0.12297	0.32657	0.25792	1	1	1	1	1	1	1	1	1	1	1	1	1	1	1	1
0.58731	0.81576	0.60232	0.85334	2	1	2	2	2	1	2	2	1	1	2	1	2	2	1	2
0.43830	0.38320	0.62508	0.64386	2	2	2	2	2	2	2	2	2	2	1	2	2	2	2	2
0.17503	0.04436	0.11356	0.98725	2	2	2	2	2	2	2	2	2	2	2	2	2	2	2	2
0.14333	0.38024	0.88466	0.91753	1	1	1	1	1	1	1	1	1	1	1	1	1	1	2	1
0.09645	0.09203	0.66011	0.39832	1	1	1	1	1	1	1	1	1	1	1	1	1	1	1	1
0.15795	0.85092	0.92937	0.53398	2	2	2	2	2	2	2	2	1	1	1	1	2	1	2	2
0.04518	0.04235	0.05169	0.08565	2	2	2	2	2	2	2	2	2	2	2	2	2	2	2	2
0.95531	0.05248	0.62769	0.11973	2	2	2	2	2	2	2	2	2	2	2	2	1	2	2	1
0.41080	0.48259	0.19366	0.21446	2	1	1	1	1	1	1	1	1	1	1	1	1	1	1	1
0.27100	0.13948	0.10852	0.75310	2	2	2	2	2	2	2	2	2	2	2	2	2	2	2	2
0.43072	0.59816	0.63031	0.55819	2	1	1	1	1	1	1	1	2	1	1	1	1	1	1	1
0.85183	0.97528	0.96504	0.85983	2	1	1	1	2	1	2	1	1	1	2	1	2	2	1	1
0.14887	0.23422	0.07735	0.04443	2	2	2	2	2	2	2	2	2	2	2	2	2	2	2	2
0.03360	0.07921	0.13919	0.00123	2	2	2	2	2	2	2	2	2	2	2	2	2	2	2	2
0.07072	0.56110	0.96193	0.26164	2	1	1	2	2	2	2	2	2	1	1	1	2	2	2	2
0.33102	0.29571	0.29702	0.28813	1	1	1	1	1	1	2	1	1	1	2	1	1	1	1	2
0.78489	0.03872	0.03517	0.03581	2	2	2	2	2	2	2	2	2	2	2	2	1	2	2	2
0.61815	0.87761	0.37822	0.52535	1	1	2	1	2	1	1	1	1	1	2	1	1	1	1	1
0.74081	0.52201	0.29780	0.91675	2	1	2	1	2	2	2	2	1	1	1	1	1	2	1	2
0.05252	0.11073	0.25892	0.09919	2	2	2	2	2	2	2	2	2	2	2	2	2	2	2	2
0.98680	0.35938	0.51295	0.87517	2	2	2	1	1	1	2	2	2	2	1	1	2	2	1	2
0.56603	0.13067	0.64117	0.14828	1	1	1	1	2	1	2	1	2	1	2	1	2	2	2	2
0.67822	0.51832	0.45972	0.53908	1	1	1	1	1	1	1	1	2	1	1	1	2	1	1	2
0.60643	0.92271	0.78402	0.80741	2	2	1	1	1	1	2	1	2	2	2	1	1	2	2	2
0.07710	0.38589	0.44288	0.23529	2	2	2	2	2	2	2	2	2	2	2	2	2	2	2	2
0.81299	0.70264	0.50708	0.33608	2	2	2	1	2	2	2	2	2	2	2	2	2	2	1	2
0.19810	0.95903	0.83634	0.31218	2	2	1	1	1	1	1	2	2	2	2	2	2	2	2	2
0.52326	0.57810	0.52143	0.85582	2	2	1	1	1	1	1	1	2	1	2	1	2	1	1	2
0.12156	0.02358	0.00054	0.00761	1	1	1	1	1	1	1	1	1	1	1	1	1	1	1	1
0.60241	0.07861	0.45944	0.33675	2	1	1	1	1	1	1	1	2	1	1	1	1	1	1	1
0.50139	0.82056	0.55179	0.53732	2	1	1	1	1	1	1	2	2	1	2	1	2	1	2	2
0.29973	0.89338	0.74472	0.04811	2	2	2	2	2	2	2	2	2	1	2	2	2	2	1	2
0.12472	0.23267	0.08025	0.00875	2	2	2	2	2	2	2	2	2	2	2	2	2	2	2	2
0.11320	0.75839	0.42938	0.06322	2	1	1	1	1	1	1	1	2	1	2	2	2	1	2	2
0.45850	0.66047	0.32331	0.37271	1	2	2	2	1	2	1	2	2	2	1	2	1	2	2	2
0.61473	0.84005	0.46907	0.96711	1	2	2	2	2	2	2	2	1	2	2	2	2	2	1	1
0.02111	0.07330	0.23496	0.00629	2	2	2	2	2	2	2	2	2	2	2	2	2	2	2	2
0.05145	0.08369	0.03736	0.01005	2	2	2	2	2	2	2	2	2	2	2	2	2	2	2	2
0.07765	0.17054	0.85651	0.29118	1	1	1	1	1	1	1	1	1	1	1	1	1	1	1	1
0.33601	0.55839	0.33204	0.71827	2	1	1	1	2	1	1	1	1	1	2	1	1	1	1	1
0.94364	0.13254	0.95278	0.94710	1	2	1	1	1	1	1	1	2	1	1	1	2	1	1	1

0.70822	0.43250	0.09917	0.91974	1	1	1	1	2	1	1	1	1	1	1	1	1	1	1	1
0.19909	0.24803	0.34587	0.06764	2	1	1	1	1	1	2	2	2	2	2	2	2	2	2	2
0.09439	0.48598	0.13912	0.02531	2	1	1	2	1	1	2	2	2	1	2	2	2	2	2	2
0.00155	0.00575	0.00726	0.00157	2	2	2	2	2	2	2	2	2	2	2	2	2	2	2	2
0.33268	0.70373	0.48574	0.71030	1	2	2	2	1	2	1	1	2	2	1	2	1	1	2	1
0.88490	0.97050	0.95857	0.72678	2	1	1	1	1	2	2	2	1	2	1	1	2	2	1	1
0.69745	0.14419	0.16666	0.09333	1	1	1	1	1	1	1	1	1	1	1	1	2	1	1	2
0.41473	0.97023	0.41246	0.48767	2	2	2	2	2	2	2	2	2	1	2	2	2	2	2	2
0.13863	0.65054	0.47705	0.28520	2	1	1	2	1	1	2	2	2	1	2	1	2	2	2	2
0.56057	0.85385	0.53304	0.70481	2	2	1	1	1	2	2	1	2	1	1	1	2	2	2	2
0.02837	0.17495	0.95512	0.33152	1	1	1	1	1	1	1	1	1	1	1	1	1	1	2	1
0.51128	0.70559	0.47888	0.42969	2	1	1	1	1	1	2	2	2	2	2	2	2	2	2	2
0.55446	0.46013	0.40526	0.66999	2	1	1	1	2	1	1	1	2	1	2	1	2	2	1	2
0.92466	0.45437	0.73875	0.79802	2	1	1	1	1	1	1	1	2	1	2	1	1	1	1	1
0.68077	0.06978	0.11680	0.20944	2	1	1	1	1	1	1	1	2	1	1	1	1	1	1	1
0.06645	0.53103	0.39514	0.14290	2	2	2	2	2	2	2	2	2	2	2	1	2	2	2	2
0.42069	0.66702	0.02803	0.98230	1	2	1	1	1	1	1	1	2	1	1	1	1	1	1	1
0.05647	0.12647	0.42652	0.31572	1	1	1	1	1	1	1	1	1	1	1	1	1	1	1	1
0.71170	0.75711	0.83781	0.49439	2	2	1	2	2	2	2	2	1	1	1	1	1	1	2	1
0.94992	0.10028	0.77234	0.66320	2	2	2	2	2	2	2	2	2	2	2	2	2	2	2	2
0.78443	0.66657	0.88937	0.54130	2	1	1	1	1	1	2	1	2	1	2	1	1	2	2	2
0.26600	0.31678	0.38897	0.57253	2	1	1	1	1	1	1	1	2	1	1	1	1	1	1	1
0.08207	0.24502	0.33174	0.08842	2	2	1	2	2	2	2	2	2	2	2	2	2	2	2	2
0.20836	0.07245	0.13098	0.15684	1	1	1	1	1	1	1	1	1	1	1	1	1	1	1	1
0.37830	0.67128	0.57727	0.51756	1	2	2	2	2	2	1	2	1	1	1	1	1	1	1	1
0.27594	0.66082	0.62349	0.43117	2	1	1	1	1	1	2	2	2	1	2	1	2	2	2	2
0.90822	0.57747	0.97409	0.97237	2	1	1	1	1	1	1	1	2	1	1	1	2	1	1	2
0.34649	0.34879	0.60335	0.89205	1	2	2	1	1	2	1	2	2	1	1	1	1	1	2	1
0.21614	0.51230	0.78823	0.69910	1	2	2	1	1	2	1	1	1	1	1	1	1	1	2	1
0.09225	0.20349	0.48136	0.24162	2	2	1	2	2	2	2	2	2	2	2	2	2	2	2	2
0.42374	0.54764	0.85353	0.64125	2	2	2	1	2	2	2	1	2	1	2	2	1	2	1	1
0.07239	0.93415	0.29346	0.00804	1	2	2	1	2	1	2	2	1	2	2	1	1	2	1	1
0.12828	0.73244	0.41484	0.10965	1	1	1	2	2	2	2	1	2	1	1	1	2	2	1	2
0.90857	0.87526	0.51102	0.90958	1	2	1	2	2	2	2	1	2	1	1	1	1	2	2	2
0.01536	0.00914	0.24259	0.23899	1	2	1	1	1	1	1	1	1	1	1	1	1	1	1	1
0.95055	0.22098	0.61400	0.83479	2	2	2	2	2	2	2	2	2	2	2	2	1	2	2	2
0.69078	0.40136	0.19357	0.34335	2	1	1	1	1	1	1	1	2	1	1	1	1	1	1	1
0.41286	0.67226	0.23971	0.06384	2	1	1	2	2	2	2	2	2	1	2	1	2	2	2	2
0.38671	0.81380	0.83682	0.85807	1	1	1	1	2	2	1	1	1	1	1	2	2	2	2	2
0.25412	0.36515	0.56796	0.81698	1	2	1	1	1	2	2	1	1	1	2	2	2	2	2	2
0.61301	0.72373	0.70123	0.76203	1	1	1	1	1	1	1	1	1	2	2	1	2	1	1	2
0.93454	0.75927	0.48042	0.76311	2	1	2	2	2	1	2	2	1	1	2	1	2	2	1	1
0.00479	0.07708	0.06135	0.00476	2	2	2	2	2	2	2	2	2	2	2	2	2	2	2	2
0.82583	0.83265	0.42830	0.44387	2	1	2	2	2	1	2	2	1	1	2	1	1	1	1	2
0.00412	0.00921	0.01545	0.00149	2	2	2	2	2	2	2	2	2	2	2	2	2	2	2	2
0.06650	0.05840	0.12570	0.03117	2	1	2	2	2	2	2	2	2	2	2	2	2	2	2	2
0.11967	0.01193	0.33471	0.09439	1	1	1	1	1	1	1	1	1	1	1	1	1	1	1	1
0.52789	0.31975	0.48108	0.23326	2	1	1	1	1	1	2	2	2	2	2	2	2	2	2	2
0.60926	0.54338	0.70943	0.32417	2	2	1	2	1	1	2	2	2	2	2	2	2	2	2	2
0.01416	0.08457	0.07010	0.01448	2	2	2	2	2	2	2	2	2	2	2	2	2	2	2	2
0.88640	0.48682	0.84063	0.30971	1	1	2	2	2	1	1	2	1	2	2	2	1	1	1	1
0.10827	0.48238	0.63310	0.19155	1	1	1	1	1	1	1	1	1	1	1	1	1	1	1	1
0.56024	0.82945	0.48385	0.72641	2	1	1	1	1	1	2	1	2	1	2	2	2	2	2	2
0.83337	0.97821	0.72534	0.94550	2	1	1	1	1	1	1	1	2	1	1	1	2	1	2	1
0.08769	0.31980	0.12430	0.01482	2	1	2	2	1	2	2	2	2	2	2	2	2	2	2	2
0.25649	0.10904	0.39725	0.07392	2	2	2	2	2	2	2	2	2	2	2	2	2	2	2	2
0.06785	0.77052	0.97107	0.75402	2	1	1	1	1	1	1	1	2	1	2	1	1	2	1	1

0.48378	0.84352	0.30759	0.70735	2	2	2	2	1	2	1	2	2	2	2	2	1	1	2	2
0.75287	0.20829	0.60475	0.04552	1	1	1	1	1	1	1	1	1	1	1	1	1	1	1	1
0.57094	0.62925	0.33980	0.70173	2	2	1	2	2	2	1	2	2	1	1	1	2	1	2	2
0.38804	0.43126	0.23394	0.02122	2	2	2	2	2	2	2	2	2	2	2	2	2	2	2	2
0.02844	0.43082	0.37659	0.00325	2	2	2	2	2	2	2	2	2	2	2	2	2	2	2	2
0.21331	0.83255	0.46168	0.15050	2	1	1	2	1	1	2	2	2	1	2	2	2	2	2	2
0.79526	0.26841	0.05550	0.38121	1	1	1	1	1	1	1	1	2	1	1	1	1	1	1	1
0.11503	0.67000	0.65794	0.14981	2	1	1	2	1	2	2	2	2	1	2	2	2	2	2	2
0.15802	0.52555	0.31002	0.05099	2	2	2	2	2	2	2	2	2	1	2	1	2	2	2	2
0.98924	0.30015	0.68722	0.77588	2	1	2	2	2	2	2	1	2	1	1	1	2	1	1	1
0.44788	0.68936	0.35934	0.78020	1	1	1	1	2	1	1	1	1	1	1	1	1	1	1	1
0.69186	0.80911	0.82881	0.48605	1	1	1	1	1	1	1	1	2	1	1	1	1	2	1	1
0.05190	0.09957	0.22786	0.01184	2	2	2	2	2	2	2	2	2	2	2	2	2	2	2	2
0.61840	0.18279	0.99019	0.24937	2	2	2	2	2	2	2	2	2	2	2	2	2	2	1	2
0.39055	0.75906	0.27535	0.04174	2	1	1	1	1	1	1	2	2	1	2	2	2	2	2	2
0.19651	0.51560	0.59610	0.77033	1	2	2	1	1	2	1	1	2	2	1	1	1	1	2	1
0.15253	0.02981	0.14502	0.00477	2	2	2	2	2	2	2	2	2	2	2	2	2	2	2	2
0.69200	0.94450	0.42981	0.57571	2	2	2	2	2	2	2	2	2	1	2	1	2	2	2	2
0.56003	0.85127	0.66635	0.43346	1	1	1	1	1	1	1	1	2	1	1	2	2	1	1	2
0.23343	0.67077	0.39229	0.26413	2	2	2	2	1	2	2	2	2	1	2	2	2	2	2	2
0.02167	0.02952	0.11091	0.00559	2	2	2	2	2	2	2	2	2	2	2	2	2	2	2	2
0.02884	0.10053	0.02187	0.00464	2	2	2	2	2	2	2	2	2	2	2	2	2	2	2	2
0.18722	0.96121	0.33735	0.00971	2	1	1	2	1	2	2	2	2	1	2	2	2	1	2	2
0.77036	0.18739	0.08742	0.52013	1	2	2	2	2	1	1	1	2	2	2	2	2	1	2	1
0.23805	0.18113	0.08614	0.14846	1	1	1	1	1	1	1	1	1	1	1	1	1	1	1	1
0.09601	0.16705	0.27920	0.06808	2	2	2	2	2	2	2	2	2	2	2	2	2	2	2	2
0.49831	0.55788	0.70754	0.74435	2	1	1	1	1	1	1	1	2	1	1	1	2	1	1	2
0.22404	0.36515	0.78229	0.46722	2	2	2	2	2	1	1	2	2	2	1	1	2	2	1	2
0.05975	0.10071	0.09645	0.24749	1	1	1	1	1	1	1	1	1	1	1	1	1	1	1	1
0.23017	0.87049	0.08776	0.38507	1	1	1	1	1	1	1	1	1	2	1	1	1	2	1	1
0.44024	0.65774	0.39654	0.31268	2	1	1	1	1	1	1	1	2	1	2	1	2	2	2	2
0.17861	0.60265	0.16137	0.06428	2	1	1	1	1	1	2	2	2	1	2	2	2	2	2	2
0.31201	0.08629	0.45050	0.16251	1	2	1	1	2	2	2	1	1	1	1	2	2	2	1	2
0.14462	0.26971	0.36682	0.56448	1	2	2	1	1	1	1	1	2	2	1	1	1	1	1	1
0.20498	0.71554	0.31829	0.34981	2	1	1	2	1	1	2	2	2	1	2	2	2	2	2	2
0.92754	0.25202	0.97952	0.91936	1	1	1	1	1	1	1	1	1	1	1	1	2	1	1	1
0.33506	0.52986	0.27404	0.69495	2	1	1	1	2	1	2	1	1	1	2	1	1	1	1	1
0.40758	0.27491	0.73703	0.13269	2	2	2	2	2	2	2	2	1	1	2	2	1	2	1	2
0.35153	0.69504	0.47408	0.53825	1	1	1	1	1	1	1	1	2	2	2	1	1	1	1	1
0.36016	0.28206	0.07269	0.63723	1	1	1	1	1	1	1	1	2	1	1	1	1	1	1	1
0.02508	0.72701	0.30663	0.02290	2	1	2	2	2	2	2	2	2	1	2	2	2	2	2	2
0.58053	0.24425	0.95171	0.51782	2	2	2	2	2	2	2	2	2	2	1	1	2	2	2	2
0.26803	0.04255	0.11698	0.63300	2	2	2	2	2	2	2	2	2	2	2	2	2	2	2	2
0.82811	0.46337	0.21908	0.81781	2	2	2	2	2	2	2	1	2	2	2	2	1	2	2	1
0.54316	0.61294	0.38866	0.31938	2	2	2	2	1	1	2	1	1	1	1	1	1	2	1	2
0.07308	0.06687	0.13129	0.08955	2	2	2	2	2	2	2	2	2	2	2	2	2	2	2	2
0.59080	0.79779	0.61889	0.57367	1	2	2	2	2	2	2	1	1	1	2	1	1	1	1	1
0.94290	0.46683	0.82449	0.55640	2	2	2	2	2	2	2	2	1	1	2	2	1	2	1	2
0.90650	0.36758	0.71449	0.95672	2	2	2	2	2	2	2	2	1	2	2	2	1	2	1	1
0.66671	0.00256	0.63957	0.30854	2	2	2	2	2	2	2	2	2	2	2	2	2	2	1	2
0.57709	0.47557	0.27309	0.67906	1	1	1	1	1	1	1	1	1	1	1	1	1	1	1	1
0.70579	0.80509	0.82759	0.99335	1	1	1	1	1	1	1	1	1	1	1	1	1	1	2	1
0.00013	0.01586	0.00033	0.00012	2	2	2	2	2	2	2	2	2	2	2	2	2	2	2	2
0.01281	0.34053	0.48294	0.30899	2	2	2	2	2	2	2	2	1	2	2	2	2	2	2	2
0.58849	0.27211	0.72480	0.35864	2	2	2	2	2	2	2	2	2	2	2	2	2	2	2	2
0.46756	0.82576	0.55230	0.35885	1	2	2	2	2	2	1	2	1	2	1	1	1	2	1	1
0.02086	0.17823	0.38179	0.00385	2	2	2	2	2	2	2	2	2	2	2	2	2	2	2	2

0.38384	0.15415	0.13926	0.67798	2	2	2	2	2	2	2	2	2	2	2	1	1	2	1	1
0.01368	0.05734	0.88512	0.07940	2	2	2	2	2	2	2	2	2	2	2	2	2	2	2	2
0.74628	0.88592	0.51779	0.89870	2	1	2	2	2	2	2	1	2	1	2	1	1	2	1	1
0.29391	0.70289	0.92751	0.88632	2	2	2	2	1	2	1	2	2	2	2	1	1	1	2	2
0.00909	0.01008	0.09811	0.03791	2	2	2	2	2	2	2	1	2	2	2	2	2	2	2	2
0.08352	0.56369	0.80726	0.52783	2	2	1	2	2	2	2	1	2	1	2	2	2	2	2	2
0.11596	0.03439	0.26145	0.06022	2	2	2	2	2	2	2	2	2	2	2	2	2	2	2	2
0.31628	0.05947	0.35293	0.05438	2	2	2	2	2	2	2	2	2	2	2	2	2	2	2	2
0.16778	0.35942	0.64900	0.25071	1	2	2	1	1	1	1	1	1	2	2	1	1	1	1	1
0.01333	0.00269	0.03243	0.00729	2	2	2	2	2	2	2	2	2	2	2	2	2	2	2	2
0.31103	0.49023	0.26164	0.39932	1	2	2	2	2	2	2	1	2	2	1	2	1	2	1	1
0.45120	0.90087	0.75220	0.77327	2	2	2	2	2	2	2	2	1	1	2	2	1	1	1	1
0.29295	0.66529	0.39622	0.20009	1	2	2	1	2	2	1	1	1	1	1	1	1	1	1	1
0.27156	0.63208	0.30953	0.32549	1	2	2	2	2	1	2	2	1	1	1	1	1	1	1	1
0.98471	0.22314	0.51953	0.08795	2	2	2	2	2	1	2	2	2	2	2	2	1	2	2	2
0.75025	0.35553	0.30931	0.46489	2	2	2	2	2	2	2	2	2	2	2	2	1	2	2	1
0.52897	0.36820	0.21260	0.57756	2	1	1	2	1	1	1	2	2	1	1	1	2	1	1	2
0.29553	0.90235	0.58502	0.67240	1	1	1	1	1	1	1	1	1	1	1	1	1	2	1	1
0.01665	0.06886	0.05487	0.02196	2	2	2	2	2	2	2	2	2	2	2	2	2	2	2	2
0.49904	0.79790	0.39416	0.53675	2	1	2	2	2	1	2	1	1	1	2	1	1	2	1	2
0.32477	0.49501	0.68890	0.70180	2	2	2	1	1	2	2	1	2	1	1	1	1	2	1	2
0.29009	0.68521	0.77942	0.51083	2	2	2	1	1	1	2	1	2	1	1	1	1	2	1	2
0.08232	0.99073	0.90221	0.12291	2	2	2	2	2	2	2	2	2	1	2	2	2	1	2	2
0.97443	0.26548	0.97705	0.40810	2	2	2	1	2	2	2	2	2	2	1	1	1	2	1	2
0.71104	0.44370	0.93125	0.25606	1	2	1	1	2	1	2	1	2	2	1	1	1	2	1	2
0.50524	0.16298	0.74522	0.78169	1	2	2	1	1	2	2	2	1	2	1	1	1	1	2	1
0.06055	0.16194	0.27547	0.05273	2	1	2	2	2	2	2	2	2	1	2	2	2	2	2	2
0.29207	0.30607	0.25536	0.11669	2	1	1	1	1	2	2	2	2	1	2	2	2	2	2	2
0.61075	0.57178	0.58008	0.50335	1	1	1	1	1	1	1	1	1	2	2	2	1	2	1	1
0.00494	0.03350	0.06439	0.01694	2	2	2	2	2	2	2	2	2	2	2	2	2	2	2	2
0.31688	0.49423	0.28815	0.36953	2	1	1	1	1	1	2	1	2	1	2	2	2	2	2	2
0.18464	0.99184	0.09008	0.05420	2	1	1	2	2	2	2	2	2	1	2	2	2	2	2	2
0.06100	0.10072	0.29536	0.06778	1	1	1	1	1	1	1	1	1	1	1	1	1	1	1	1
0.10182	0.34526	0.89318	0.28355	1	1	1	1	1	1	1	1	1	1	1	1	1	1	1	1
0.00433	0.05712	0.19977	0.13749	2	2	2	2	2	2	2	2	2	2	2	2	2	2	2	2
0.25216	0.76543	0.76462	0.38618	2	1	1	1	1	1	1	2	2	1	2	1	2	1	2	2
0.05856	0.00663	0.02766	0.10242	2	2	2	2	2	2	2	2	2	2	2	2	2	2	2	2
0.03266	0.21827	0.62360	0.02498	2	2	2	2	2	2	2	2	2	2	2	2	2	2	2	2
0.60878	0.67303	0.24364	0.82407	2	2	2	2	1	2	1	2	2	2	2	2	1	2	2	2
0.26571	0.68591	0.43451	0.03087	2	2	1	2	2	2	2	2	2	2	2	2	2	2	2	2
0.42559	0.68927	0.08672	0.57748	1	1	1	1	1	1	2	1	2	1	1	1	1	1	2	2
0.34988	0.47543	0.85200	0.52455	1	2	1	1	1	1	1	1	2	2	2	2	2	1	2	2
0.20380	0.93720	0.61561	0.61419	2	2	1	1	1	1	1	1	2	1	1	1	2	1	2	2
0.81810	0.79978	0.98721	0.28451	2	2	2	1	1	2	2	1	2	1	1	2	2	1	2	2
0.03265	0.00840	0.13603	0.05527	1	1	1	1	1	1	1	1	1	1	1	1	1	1	1	1
0.04217	0.11249	0.57001	0.01211	2	2	2	2	2	2	2	2	2	2	2	2	2	2	2	2
0.88776	0.34357	0.26759	0.31222	2	1	1	1	1	1	1	1	2	1	1	1	2	1	1	2
0.07688	0.27821	0.45673	0.02227	2	1	1	2	2	1	2	2	2	2	2	2	2	2	2	2
0.78598	0.64251	0.27281	0.77602	1	1	1	1	1	1	1	1	1	1	1	1	1	2	1	1
0.15312	0.21626	0.83017	0.51921	1	1	1	1	1	1	1	1	1	1	1	1	1	1	2	1
0.25607	0.47764	0.69811	0.95650	1	2	2	1	1	1	1	1	1	1	1	1	1	2	2	2
0.00062	0.00244	0.01044	0.00373	2	2	2	2	2	2	2	2	2	2	2	2	2	2	2	2
0.84332	0.69422	0.70366	0.40500	2	2	1	2	2	2	2	2	2	1	1	1	1	1	1	2
0.17093	0.81552	0.42067	0.07231	1	1	1	1	1	1	1	1	1	1	1	1	1	1	1	1
0.39771	0.58487	0.67280	0.82528	2	1	2	2	2	2	2	2	1	2	2	2	2	2	2	2
0.04311	0.19997	0.39804	0.22665	2	2	2	2	2	2	2	2	2	2	2	2	2	2	2	2
0.00013	0.00954	0.00302	0.00040	2	2	2	2	2	2	2	2	2	2	2	2	2	2	2	2

0.40837	0.22444	0.66181	0.42922	1	1	1	1	1	1	1	1	2	1	1	1	1	1	1	1
0.01133	0.00631	0.12200	0.09962	1	1	1	1	1	1	1	1	1	1	1	1	1	1	1	1
0.00079	0.00425	0.00001	0.05587	1	1	1	1	1	1	1	1	1	1	1	1	1	1	1	1
0.05148	0.17582	0.11705	0.08440	2	2	2	2	2	2	2	2	2	2	2	2	2	2	2	2
0.01135	0.02428	0.37147	0.51449	1	1	1	1	1	1	1	1	1	1	1	1	1	1	1	1
0.23902	0.35335	0.37419	0.58792	2	2	2	2	2	2	2	2	1	1	2	2	2	2	2	2
0.61614	0.61865	0.90290	0.24195	2	2	2	2	2	2	1	1	2	2	2	2	1	2	2	1
0.05947	0.09384	0.33969	0.30221	2	2	2	2	2	2	2	2	2	2	2	2	2	2	2	2
0.65538	0.54365	0.93292	0.40710	1	2	2	2	2	2	1	1	2	2	2	2	2	2	1	1
0.15618	0.08148	0.16810	0.09980	2	2	2	2	2	2	2	2	2	2	2	2	2	2	2	2
0.35912	0.85857	0.33121	0.57641	2	1	2	2	2	2	2	2	1	1	2	2	2	1	2	2
0.63840	0.87675	0.30533	0.88949	1	2	2	1	2	2	1	2	1	2	1	1	2	1	1	2
0.50816	0.95913	0.92817	0.42814	2	1	2	2	2	2	2	2	1	1	2	2	1	2	1	1
0.26612	0.55516	0.52950	0.72618	1	2	2	1	2	2	1	1	2	2	1	2	1	2	2	1
0.24562	0.50435	0.64141	0.59612	1	1	2	1	2	1	2	1	1	1	2	1	1	1	1	1
0.11406	0.20996	0.95775	0.48979	1	1	1	1	1	1	1	1	2	1	1	1	1	1	1	2
0.27049	0.61057	0.71414	0.96633	1	2	2	2	1	2	1	1	2	1	1	2	1	1	2	1
0.06706	0.04744	0.15326	0.13949	1	1	1	1	1	1	1	1	1	1	1	1	1	1	1	1
0.16593	0.37833	0.19034	0.12419	2	1	2	1	2	1	1	1	1	1	1	1	1	1	1	1
0.77907	0.06985	0.65359	0.15744	2	2	2	2	2	2	2	2	2	2	2	2	2	2	2	2
0.56911	0.88232	0.40791	0.68052	2	2	2	2	2	2	2	2	2	2	2	2	1	1	2	2
0.54038	0.01098	0.09633	0.25732	2	2	2	2	2	2	2	2	1	1	2	2	2	2	2	2
0.07742	0.02841	0.60831	0.77538	2	2	2	2	2	2	2	2	2	2	2	2	2	2	2	2
0.98861	0.96130	0.37878	0.42192	2	2	2	2	2	2	2	2	1	2	2	2	1	1	2	1
0.36046	0.35075	0.18204	0.28436	2	2	1	1	2	1	1	1	1	1	1	1	1	1	1	1
0.82511	0.98222	0.30675	0.98354	2	2	2	2	2	2	2	1	2	1	2	2	1	1	2	2
0.40286	0.26982	0.32265	0.85486	2	2	1	1	2	2	1	1	2	1	2	1	1	2	1	1
0.64731	0.62796	0.75198	0.54235	2	1	1	1	1	1	1	2	2	1	2	1	2	1	2	2
0.33206	0.81910	0.35700	0.16143	2	1	1	2	1	1	2	2	2	1	2	2	2	2	2	2
0.21283	0.90755	0.92465	0.96875	2	1	1	1	1	1	2	2	1	1	1	1	2	2	2	1
0.00471	0.01483	0.05126	0.02050	2	2	2	2	2	2	2	2	2	2	2	2	2	2	2	2
0.04023	0.12105	0.61559	0.24403	2	2	2	2	2	2	2	2	2	2	2	2	2	2	2	2
0.00749	0.02670	0.06537	0.02726	2	2	2	2	2	2	2	2	2	2	2	2	2	2	2	2
0.02313	0.03168	0.16450	0.04897	2	2	2	2	2	2	2	2	2	2	2	2	2	2	2	2
0.50920	0.10947	0.65341	0.03019	2	2	2	2	2	2	2	2	2	2	2	2	2	2	2	2
0.17872	0.09643	0.56992	0.21743	2	2	2	2	2	2	2	2	2	2	2	2	2	2	2	2
0.02671	0.05614	0.44065	0.11790	2	2	2	2	2	2	2	2	2	2	2	2	2	2	2	2
0.98348	0.14802	0.15947	0.95183	1	1	2	1	2	1	2	2	2	2	1	1	2	2	1	2
0.01042	0.07477	0.18241	0.06475	2	2	2	2	2	2	2	2	2	2	2	2	2	2	2	2
0.64855	0.25052	0.43138	0.37505	1	1	1	1	1	1	2	1	1	1	2	1	1	1	2	1
0.25531	0.59080	0.08646	0.19568	1	1	1	1	2	1	1	1	1	1	1	1	1	1	1	1
0.22137	0.21683	0.53105	0.32161	2	2	2	2	2	2	2	2	1	1	2	2	2	2	1	2
0.16962	0.53659	0.93862	0.28124	1	1	1	2	2	1	1	1	2	1	1	1	2	2	2	2
0.28181	0.10669	0.37802	0.37393	2	2	2	2	2	2	2	2	1	2	2	2	2	2	2	2
0.63544	0.93636	0.54565	0.92350	2	2	2	1	2	1	2	1	2	1	2	1	1	2	1	1
0.45590	0.56265	0.14969	0.33509	1	2	2	1	2	1	2	1	1	1	1	1	1	1	1	1
0.87899	0.93086	0.68008	0.38898	1	1	1	1	2	1	1	1	2	1	2	1	1	2	1	2
0.57793	0.24326	0.47657	0.95017	2	1	1	1	2	1	2	1	1	1	2	1	1	1	1	2
0.04114	0.01810	0.19036	0.06559	2	2	2	2	2	2	2	2	2	2	2	2	2	2	2	2
0.00409	0.02207	0.07057	0.04159	2	2	2	2	2	2	2	2	2	2	2	2	2	2	2	2
0.75125	0.74839	0.96335	0.70149	2	1	1	1	2	2	2	1	2	1	2	2	1	2	1	1
0.10387	0.10922	0.44734	0.03184	2	2	2	2	2	2	2	2	2	2	2	2	2	2	2	2
0.09484	0.14419	0.47473	0.39365	1	1	1	1	1	1	1	1	2	1	1	1	1	1	1	2
0.18117	0.63282	0.85685	0.16249	2	1	1	2	2	1	2	2	2	1	1	1	2	1	2	2
0.60032	0.80032	0.63774	0.52090	1	1	1	1	1	1	1	1	2	1	2	2	2	2	2	2
0.30309	0.63362	0.60003	0.85231	1	2	2	2	1	2	1	2	2	2	1	2	1	1	2	1
0.33245	0.60257	0.37084	0.67582	2	2	2	2	2	2	2	2	1	2	1	1	1	2	1	1

0.10190	0.51979	0.44512	0.32346	2	2	2	1	2	2	1	1	1	1	1	1	1	1	1	1
0.00101	0.04820	0.56104	0.00640	2	2	2	2	2	2	2	2	2	2	2	2	2	2	2	2
0.00030	0.00319	0.07218	0.00012	2	2	2	2	2	2	2	2	2	2	2	2	2	2	2	2
0.07649	0.09510	0.34974	0.14339	2	2	2	2	2	2	2	2	2	2	2	2	2	2	2	2
0.18404	0.65844	0.69466	0.95666	2	2	1	2	2	2	2	1	2	1	2	2	2	2	1	2
0.87632	0.93471	0.48292	0.50644	2	1	2	2	2	1	2	2	1	1	1	1	2	1	1	2
0.27436	0.70747	0.39614	0.45295	2	2	2	2	2	2	2	2	1	1	1	1	1	1	1	1
0.09083	0.32514	0.15588	0.49942	1	1	1	1	1	1	1	1	1	1	1	1	1	1	1	1
0.57292	0.60529	0.28079	0.84326	1	1	1	1	1	1	1	1	1	1	1	1	1	1	1	2
0.49497	0.90815	0.98187	0.62333	1	2	2	2	1	2	1	2	2	2	1	2	1	2	1	2
0.15706	0.06498	0.27178	0.42822	1	1	1	1	1	1	1	1	1	1	1	1	1	1	1	1
0.03357	0.06891	0.03619	0.00272	1	1	1	1	1	1	1	1	1	1	1	1	1	1	1	1
0.02123	0.82664	0.56940	0.12150	2	2	2	2	2	2	2	2	2	2	2	1	2	2	2	2
0.01809	0.02154	0.03533	0.01250	2	2	2	2	2	2	2	2	2	2	2	2	2	2	2	2
0.03160	0.61267	0.03546	0.03117	1	1	1	1	1	1	1	1	1	1	1	1	1	1	1	1
0.72260	0.55800	0.44051	0.78201	1	2	2	2	2	2	2	2	1	2	1	1	1	2	1	1
0.12607	0.00386	0.12916	0.12340	1	1	1	1	1	1	1	1	1	1	1	1	1	1	1	1
0.84341	0.12589	0.18633	0.04882	1	2	2	2	2	2	2	2	2	2	2	1	2	2	2	2
0.01486	0.02128	0.10380	0.12730	2	2	2	2	2	2	2	2	2	2	2	2	2	2	2	2
0.56698	0.83323	0.38043	0.88184	2	2	2	2	1	2	1	2	2	2	1	2	1	1	2	2
0.07862	0.18164	0.66058	0.27321	1	1	1	1	1	1	1	1	1	1	1	1	1	1	1	1
0.55932	0.96570	0.38787	0.97683	1	2	2	2	1	2	1	2	2	2	1	2	1	1	2	2
0.91282	0.45421	0.20292	0.80703	2	2	2	2	2	2	2	1	2	2	2	2	1	2	2	2
0.03571	0.51876	0.42076	0.19202	2	1	2	2	2	2	2	2	2	1	2	2	2	2	2	2
0.00050	0.00078	0.01102	0.01281	2	2	2	2	2	2	2	2	2	2	2	2	2	2	2	2
0.88133	0.53739	0.70077	0.42790	2	2	2	1	2	2	2	1	1	1	2	2	2	2	1	2
0.15903	0.34278	0.86858	0.48684	1	1	1	1	1	1	1	1	1	1	1	1	1	1	2	1
0.31045	0.25949	0.07856	0.51283	1	1	1	1	1	1	1	1	1	1	1	1	1	1	1	1
0.05312	0.02097	0.07209	0.05100	1	1	1	1	1	1	1	1	1	1	1	1	1	1	1	1
0.03746	0.67906	0.03713	0.16808	1	1	1	1	1	1	1	1	1	1	1	1	1	1	1	1
0.97061	0.72945	0.79772	0.83931	2	1	1	2	2	2	1	1	2	2	1	1	2	2	1	2
0.26435	0.35153	0.90267	0.89568	2	1	1	1	1	1	1	1	2	1	2	1	2	1	1	1
0.02005	0.07343	0.04776	0.00375	1	1	1	1	1	1	1	1	1	1	1	1	1	1	1	1
0.91765	0.35660	0.75229	0.87355	2	1	2	2	2	2	2	2	1	2	2	1	2	2	1	2
0.36477	0.82804	0.78711	0.51722	2	1	1	1	1	1	1	2	2	1	2	1	1	1	1	2
0.36435	0.50573	0.24281	0.46241	1	1	1	1	1	1	1	1	1	2	1	1	1	2	1	1
0.21904	0.22681	0.30756	0.20637	2	1	1	1	2	2	2	2	2	1	2	1	2	2	2	2
0.11188	0.14596	0.32709	0.16208	1	1	1	1	1	1	1	1	1	1	1	1	1	1	1	1
0.06200	0.12952	0.19885	0.15564	2	2	2	2	2	2	2	2	2	2	2	2	2	2	2	2
0.03528	0.07468	0.07697	0.12617	2	2	2	2	2	2	2	2	2	2	2	2	2	2	2	2
0.13185	0.10514	0.36667	0.13719	1	1	1	1	1	1	1	1	1	1	1	1	1	1	1	1
0.06006	0.08364	0.10539	0.10817	2	2	2	2	2	2	2	2	2	2	2	2	2	2	2	2
0.47892	0.15990	0.47701	0.15085	2	2	2	2	2	2	2	2	2	2	2	2	2	2	2	2
0.02643	0.09153	0.36246	0.07559	1	1	1	1	1	1	1	1	1	1	1	1	1	1	1	1
0.00911	0.01331	0.02561	0.12129	2	2	2	2	2	2	2	2	2	2	2	2	2	2	2	2
0.28369	0.59312	0.54169	0.79138	2	2	1	1	2	1	1	1	2	1	1	1	1	1	1	2
0.47623	0.53480	0.34640	0.34715	2	2	1	2	2	2	2	2	2	2	2	2	2	2	2	2
0.30863	0.01790	0.04094	0.19372	2	2	2	2	2	2	2	2	2	2	2	2	2	2	2	2
0.89057	0.31013	0.70576	0.08833	2	2	1	2	2	2	2	2	2	1	2	1	1	2	2	2
0.51147	0.90453	0.28732	0.47003	1	1	1	1	1	1	1	1	1	1	1	1	1	2	2	2
0.33745	0.31966	0.28357	0.34032	2	1	1	2	2	2	2	2	2	1	2	1	2	2	2	2
0.46861	0.84962	0.37732	0.10229	1	1	2	2	2	2	2	2	2	2	2	2	2	1	2	2
0.11067	0.23586	0.02130	0.00374	2	2	2	2	2	2	2	2	2	2	2	2	2	2	2	2
0.27276	0.12527	0.92536	0.83699	1	1	1	1	1	1	1	1	1	1	1	1	1	1	1	1
0.75302	0.22992	0.72647	0.27288	2	2	2	2	2	2	2	2	1	1	2	1	1	2	1	2
0.47229	0.74501	0.57699	0.33895	2	2	2	2	2	2	2	1	2	1	2	2	1	2	2	1
0.26535	0.20978	0.15800	0.01745	2	2	2	2	2	2	2	2	2	2	2	2	2	2	2	2

0.37847	0.00504	0.08620	0.14636	2	2	2	2	2	2	2	2	2	2	2	2	2	2	2	2
0.69780	0.82607	0.72582	0.64003	1	1	1	1	2	2	1	1	1	1	1	1	2	2	2	2
0.93614	0.95715	0.51845	0.71791	1	1	1	1	1	1	1	1	1	1	1	1	1	2	2	1
0.52018	0.04644	0.78807	0.65594	2	2	2	2	2	2	2	2	1	2	1	1	1	2	1	2
0.83619	0.15422	0.21926	0.18838	1	1	1	1	1	1	1	1	2	1	1	1	1	1	1	1
0.99775	0.56460	0.47044	0.94626	2	1	1	2	1	1	2	1	2	1	2	1	1	1	1	1
0.12009	0.25600	0.34057	0.81843	1	1	1	1	1	1	1	1	2	1	1	1	1	1	1	1
0.06036	0.60855	0.60708	0.12724	2	2	2	2	2	2	2	2	2	2	2	2	2	2	2	2
0.43779	0.37156	0.47657	0.17582	2	2	2	2	2	2	2	2	2	2	2	2	2	2	2	2
0.26415	0.98190	0.58806	0.10610	1	1	2	2	1	1	1	1	1	1	2	2	1	1	2	2
0.04146	0.09986	0.17359	0.16080	1	1	1	1	1	1	1	1	1	1	1	1	1	1	1	1
0.53813	0.35635	0.07439	0.12065	2	2	2	2	2	2	2	2	2	2	2	2	2	2	2	2
0.33511	0.68241	0.52358	0.42234	1	1	1	1	1	1	1	1	1	1	2	1	1	2	2	1
0.76762	0.84095	0.19223	0.72048	2	1	1	1	1	1	1	1	2	2	2	2	2	2	2	1
0.10175	0.07257	0.41481	0.09314	2	2	2	2	2	2	2	2	2	2	2	2	2	2	2	2
0.98590	0.73154	0.15793	0.98890	2	1	1	1	2	1	2	1	1	1	1	1	2	1	1	1
0.28763	0.68929	0.42405	0.76112	2	2	2	2	1	2	1	2	2	2	1	2	1	1	2	2
0.19896	0.48889	0.86437	0.23958	1	2	1	1	2	2	2	2	1	1	1	2	2	2	1	2
0.43206	0.72211	0.38279	0.57293	1	1	1	1	1	1	1	1	1	1	1	1	1	1	1	1
0.17125	0.29315	0.54303	0.35223	1	2	2	1	1	1	1	1	2	2	2	2	1	1	2	1
0.02170	0.15475	0.05341	0.09402	1	1	1	1	1	1	1	1	1	1	1	1	1	1	1	1
0.64322	0.43397	0.96231	0.50752	2	2	2	1	1	1	2	2	1	1	1	1	2	2	1	1
0.06721	0.16267	0.32137	0.02167	1	1	1	1	1	1	1	1	1	1	1	1	1	1	1	1
0.37866	0.62446	0.15246	0.11937	2	2	2	2	2	2	2	2	2	1	2	1	2	1	2	2
0.90945	0.32426	0.31653	0.70264	2	2	2	2	2	2	2	2	2	2	2	2	2	2	2	2
0.53078	0.19000	0.82418	0.75320	2	2	2	2	2	2	2	2	2	2	2	1	1	2	1	1
0.01399	0.18028	0.11033	0.00680	2	2	2	2	2	2	2	2	2	2	2	2	2	2	2	2
0.29510	0.59004	0.62995	0.20006	1	1	1	1	1	1	1	1	1	1	1	1	1	1	1	1
0.02236	0.42833	0.26960	0.05672	2	1	2	2	2	2	2	2	2	1	2	2	2	2	2	2
0.79836	0.13734	0.33906	0.11697	2	2	2	2	2	2	2	2	2	2	2	2	1	2	2	2
0.31688	0.86149	0.28540	0.95856	1	2	2	2	1	2	1	2	2	2	1	2	1	2	2	2
0.00580	0.07054	0.07739	0.01186	1	1	1	1	1	1	1	1	1	1	1	1	1	1	1	1
0.10692	0.64578	0.12463	0.28016	1	1	1	1	1	1	1	1	1	2	1	1	1	1	1	1
0.74958	0.99387	0.80953	0.94663	2	2	2	1	2	2	2	1	2	1	2	2	1	1	2	2
0.06831	0.23239	0.10978	0.11681	1	1	1	1	1	1	1	1	1	1	1	1	1	1	1	1
0.31105	0.66873	0.26281	0.22395	2	1	1	1	2	1	1	1	1	1	1	1	1	2	1	1
0.15282	0.49433	0.14972	0.08781	2	2	2	2	2	2	2	2	2	2	2	2	2	2	2	2
0.17001	0.27108	0.78330	0.09631	1	1	1	1	1	1	1	1	1	2	2	2	1	1	1	1
0.00633	0.07601	0.32089	0.01745	2	1	2	2	2	2	2	2	2	1	2	2	2	2	2	2
0.33471	0.93373	0.43053	0.35395	1	2	2	2	1	1	1	2	2	1	1	2	1	2	1	2
0.00310	0.24231	0.72645	0.01267	2	2	2	2	2	2	2	2	2	2	2	2	2	2	2	2
0.71650	0.23424	0.39729	0.11435	2	1	1	2	2	2	2	2	2	1	2	1	2	2	2	2
0.93403	0.30640	0.99310	0.64235	1	1	1	1	1	1	1	1	1	1	1	1	2	1	1	1
0.41975	0.70139	0.13141	0.63615	1	2	2	2	2	2	1	2	2	2	2	2	1	2	2	1
0.54022	0.59079	0.63450	0.18276	1	2	1	1	1	1	1	1	1	2	1	1	1	1	1	1
0.11065	0.09954	0.84036	0.41926	1	1	1	1	1	1	1	1	1	1	1	1	1	1	2	1
0.52914	0.59124	0.61623	0.34281	1	1	2	1	1	1	1	1	2	2	2	2	1	1	2	1
0.51736	0.38644	0.70873	0.39568	1	2	2	1	2	2	1	1	1	2	2	2	1	1	2	1
0.33566	0.91678	0.51631	0.35486	2	2	1	2	2	2	2	2	1	1	1	1	2	2	1	2
0.00458	0.09289	0.10622	0.06888	2	2	2	2	2	2	2	2	2	2	2	2	2	2	2	2
0.31894	0.15133	0.22368	0.37859	1	1	1	1	1	1	1	1	1	1	1	1	1	1	1	1
0.55717	0.54711	0.90516	0.81760	2	1	1	1	1	2	2	2	1	1	1	1	2	2	1	1
0.22817	0.19225	0.81913	0.34911	2	1	1	2	2	2	1	2	2	1	2	1	2	2	2	2
0.68767	0.52898	0.72539	0.26840	2	2	2	2	2	2	2	2	1	1	2	2	2	2	1	2
0.92876	0.90629	0.77564	0.73735	2	2	1	2	2	2	2	2	2	1	1	1	1	1	2	2
0.94377	0.34013	0.18662	0.46660	2	2	2	2	2	2	2	2	2	2	2	2	2	2	2	2
0.20528	0.34832	0.88833	0.76237	2	1	1	1	2	2	2	2	1	1	2	1	2	2	1	2

0.12373	0.14824	0.60948	0.41498	1	1	1	1	1	1	1	1	1	1	1	1	1	1	1	1
0.99177	0.76168	0.95762	0.73155	1	2	2	1	2	2	2	1	2	1	2	1	1	1	2	1
0.49731	0.47271	0.22172	0.47759	1	1	1	1	1	1	1	1	1	1	1	1	1	1	1	1
0.97736	0.52161	0.16175	0.44528	2	2	2	2	2	2	2	2	2	2	2	2	2	2	2	2
0.15168	0.62756	0.25320	0.31114	1	2	2	1	2	1	1	2	1	1	1	1	1	1	1	1
0.62141	0.12097	0.78707	0.89720	2	1	1	1	1	1	1	1	2	1	1	1	1	1	1	2
0.26294	0.93367	0.18916	0.03047	2	1	2	2	2	2	2	2	2	1	2	2	2	1	2	2
0.07221	0.35617	0.44300	0.42251	2	2	1	2	2	2	2	2	2	2	2	2	2	2	2	2
0.64425	0.09483	0.28242	0.02280	2	2	2	2	2	2	2	2	2	2	2	2	2	2	2	2
0.82342	0.62962	0.22304	0.96895	1	2	2	2	2	1	2	2	1	2	1	1	1	1	1	2
0.29952	0.27633	0.99255	0.74650	1	1	1	1	1	1	1	1	1	2	1	1	1	1	1	1
0.21856	0.76017	0.31158	0.09193	2	1	1	1	1	1	2	2	2	1	2	2	2	2	2	2
0.91690	0.29675	0.98721	0.90636	2	2	1	2	1	2	2	2	1	2	2	2	2	2	1	2
0.19949	0.04874	0.10001	0.55121	2	2	2	2	2	2	2	2	2	2	2	2	2	2	2	2
0.82006	0.59021	0.25863	0.62531	2	2	2	2	2	2	2	2	1	1	1	1	2	2	1	2
0.03219	0.17732	0.09796	0.02803	1	1	1	1	1	1	1	1	1	1	1	1	1	1	1	1
0.51821	0.48693	0.84612	0.58057	1	1	2	2	2	2	2	2	1	1	2	2	1	2	2	2
0.23004	0.84950	0.67409	0.88919	1	1	1	1	1	1	1	1	1	1	1	1	1	1	2	1
0.39780	0.62185	0.97896	0.81321	2	1	2	2	2	1	2	1	1	1	2	1	2	1	1	2
0.17267	0.49466	0.27390	0.98477	2	1	1	1	1	1	1	1	2	1	2	1	1	1	1	2
0.51415	0.97928	0.37223	0.57513	2	2	2	1	2	2	2	1	1	1	2	1	1	2	1	1
0.18927	0.05128	0.97169	0.07058	2	2	2	2	2	2	2	2	2	1	2	2	2	2	2	2
0.13029	0.04308	0.18717	0.35850	1	1	1	1	1	1	1	1	1	1	1	1	1	1	1	1
0.01552	0.26197	0.01589	0.16697	1	1	1	1	1	1	1	1	1	1	1	1	1	1	1	1
0.99613	0.43627	0.80241	0.90493	1	1	1	1	2	1	1	1	1	1	2	1	1	2	1	1
0.08763	0.18700	0.01544	0.71458	2	2	2	2	2	2	2	2	2	2	2	2	2	2	2	1
0.36118	0.26637	0.62836	0.85086	1	1	1	1	1	1	1	1	2	2	1	1	1	1	1	1
0.14073	0.14677	0.67765	0.82755	1	1	1	1	1	1	1	1	1	1	1	1	1	1	1	1
0.66074	0.75105	0.78889	0.77069	2	1	1	1	1	1	2	1	2	2	2	1	1	2	2	2
0.20851	0.98597	0.57652	0.89189	1	2	2	2	1	1	1	2	2	2	1	1	1	2	2	1
0.77225	0.45970	0.52360	0.84490	2	1	2	2	2	2	2	1	2	1	2	2	2	2	2	2
0.05219	0.33250	0.10131	0.01603	1	1	1	1	1	1	1	1	1	1	1	1	1	1	1	1
0.17077	0.30260	0.18864	0.06780	1	2	2	1	2	2	1	1	1	2	1	1	1	1	1	1
0.09383	0.50854	0.20283	0.29229	1	2	2	2	2	2	2	2	2	2	2	2	1	1	2	1
0.01160	0.05817	0.14321	0.18557	1	1	1	1	1	1	1	1	1	1	1	1	1	1	1	1
0.06839	0.56835	0.15489	0.06436	2	1	1	2	1	2	2	2	2	1	2	1	2	2	2	2
0.91873	0.75909	0.58254	0.23629	2	2	2	1	2	2	2	2	2	1	1	1	1	2	2	2
0.12039	0.53644	0.45804	0.03850	2	1	1	2	1	1	2	2	2	2	2	2	2	2	2	2
