# Supplementary material for: Longitudinal study on circulating miRNAs in patients after lung cancer resection
Source: Oncotarget. 2015 May 29;6(18):16674–85. doi: 10.18632/oncotarget.4322 (PMC4599298; doi:10.18632/oncotarget.4322)
Supplement: Supplementary file 5 [file oncotarget-06-16674-s005.pdf]

A	B	C	D	E	F	G	H	I	J	K	L	M	N
	sum of comparisons with significant p-value	# of comparisons with significant p-value	# of comparisons with significant p-value	without metastases									w
**miRNA**	**p 0.05**	**without metastases p 0.05**	**with metastases p 0.05**	**TP1 vs TP2 p-value**	**TP1 vs TP3 p-value**	**TP1 vs TP4 p-value**	**TP1 vs TP5 p-value**	**TP1 vs TP6 p-value**	**TP1 vs TP7 p-value**	**TP1 vs TP8 p-value**	**TP1 vs TP2 p-value**	**TP1 vs TP3 p-value**	**TP1 vs TP4 p-value**
hsa-miR-101	7	0	7	0.85335	0.16390	0.44747	0.25062	0.38922	0.70868	0.30347	0.03290	0.02677	0.03560
hsa-miR-186	8	2	6	0.14587	0.04675	0.02689	0.10486	0.15825	0.10796	0.18637	0.01476	0.06943	0.00064
hsa-miR-30e	7	2	5	0.18944	0.03400	0.15434	0.11738	0.02578	0.18491	0.19760	0.02624	0.06436	0.00020
hsa-miR-194	6	2	4	0.06037	0.01019	0.05454	0.10537	0.31883	0.06372	0.04828	0.02669	0.06889	0.01007
hsa-miR-142-5p	4	0	4	0.35358	0.08012	0.16243	0.05103	0.07570	0.12719	0.17936	0.08104	0.07702	0.01965
hsa-miR-629*	4	0	4	0.67229	0.16838	0.60885	0.55989	0.38263	0.81993	0.05953	0.04533	0.02830	0.38568
hsa-miR-30a	4	0	4	0.18291	0.29075	0.31459	0.98718	0.58202	0.92670	0.68705	0.61355	0.03948	0.02796
hsa-miR-17*	8	5	3	0.01260	0.00696	0.04004	0.09950	0.05832	0.03386	0.03778	0.00499	0.27098	0.01901
hsa-miR-210	6	3	3	0.01470	0.07761	0.00059	0.05175	0.15214	0.42155	0.02014	0.00413	0.21339	0.00584
hsa-miR-144	5	2	3	0.70975	0.05145	0.03789	0.05254	0.24272	0.09027	0.04313	0.22007	0.08540	0.02398
hsa-miR-484	5	2	3	0.03453	0.05680	0.00583	0.17576	0.12899	0.23157	0.51581	0.00394	0.17742	0.03426
hsa-miR-106b	4	1	3	0.42750	0.05362	0.07779	0.02061	0.22447	0.13355	0.18153	0.21584	0.10750	0.01418
hsa-miR-29b	4	1	3	0.23490	0.05397	0.16401	0.00923	0.11589	0.11992	0.16530	0.04981	0.01851	0.07117
hsa-miR-140-3p	3	0	3	0.94825	0.62370	0.41926	0.73628	0.26490	0.88651	0.74183	0.08014	0.09956	0.00967
hsa-miR-144*	3	0	3	0.12957	0.06637	0.06051	0.09959	0.24052	0.12892	0.10821	0.03998	0.04254	0.09574
hsa-miR-215	3	0	3	0.36492	0.13143	0.96250	0.40412	0.42964	0.05172	0.21774	0.03453	0.04231	0.06286
hsa-miR-454	9	7	2	0.01075	0.03238	0.03007	0.00982	0.03417	0.01984	0.02940	0.02541	0.55163	0.16606
hsa-miR-718	5	3	2	0.03304	0.09820	0.21528	0.00355	0.44114	0.03342	0.08668	0.03456	0.14447	0.23313
hsa-miR-21*	5	3	2	0.04281	0.20443	0.03509	0.81866	0.05269	0.46476	0.00973	0.00870	0.43076	0.15470
hsa-miR-671-3p	5	3	2	0.00117	0.02886	0.02850	0.50845	0.84804	0.74705	0.06882	0.65129	0.35907	0.36918
hsa-miR-627	5	3	2	0.29020	0.04224	0.04200	0.70681	0.05595	0.07152	0.02937	0.26387	0.39880	0.01764
hsa-miR-150*	4	2	2	0.06907	0.20674	0.21293	0.00376	0.41350	0.01986	0.13816	0.13484	0.09291	0.18518
hsa-miR-769-5p	3	1	2	0.04505	0.18203	0.19122	0.09963	0.09028	0.18988	0.18127	0.13184	0.38650	0.24749
hsa-miR-222	2	0	2	0.34309	0.06881	0.21277	0.26388	0.06711	0.36655	0.18215	0.19727	0.16067	0.00342
hsa-miR-1246	2	0	2	0.98976	0.64478	0.24184	0.28026	0.55476	0.99002	0.20066	0.18457	0.25306	0.43829
hsa-miR-320b	2	0	2	0.75022	0.81116	0.12539	0.71312	0.58777	0.54414	0.56657	0.02493	0.12353	0.08501
hsa-miR-4291	2	0	2	0.73936	0.77353	0.86551	0.95616	0.42720	0.65656	0.65261	0.16623	0.16862	0.04259
hsa-miR-199b-5p	2	0	2	0.41685	0.22157	0.84342	0.83605	0.53085	0.07766	0.44042	0.04911	0.50850	0.15335
hsa-miR-98	7	6	1	0.02281	0.02436	0.04598	0.02180	0.03121	0.02704	0.06761	0.04767	0.71596	0.53567
hsa-miR-30e*	6	5	1	0.02788	0.03630	0.12925	0.03401	0.01575	0.03399	0.15091	0.04955	0.61005	0.28378
hsa-miR-630	5	4	1	0.01088	0.00331	0.02464	0.08765	0.00194	0.19882	0.17435	0.03269	0.82309	0.17599
hsa-miR-3147	4	3	1	0.00164	0.03425	0.92466	0.01470	0.12732	0.37414	0.45755	0.90022	0.21469	0.40455
hsa-miR-18b	4	3	1	0.03047	0.04794	0.16734	0.04263	0.30200	0.43223	0.33740	0.00784	0.12795	0.07611
hsa-miR-3656	4	3	1	0.02569	0.07095	0.53129	0.04434	0.04167	0.73336	0.81216	0.03421	0.58785	0.94393
hsa-miR-301a	4	3	1	0.04332	0.02582	0.16994	0.01013	0.21043	0.14956	0.19380	0.01875	0.27056	0.16355
hsa-miR-1915	4	3	1	0.04237	0.23304	0.39465	0.04186	0.55009	0.12105	0.02256	0.02130	0.08195	0.90006
hsa-miR-374a	4	3	1	0.01640	0.03739	0.11905	0.00404	0.10498	0.15565	0.35955	0.03697	0.21420	0.30460
hsa-miR-3680*	4	3	1	0.00112	0.04338	0.09195	0.07442	0.01234	0.25809	0.60334	0.01594	0.47178	0.88693
hsa-miR-30c	4	3	1	0.03753	0.02071	0.48530	0.03267	0.25030	0.96513	0.94162	0.04713	0.80589	0.52072
hsa-miR-760	3	2	1	0.01007	0.40241	0.45449	0.03488	0.10779	0.71867	0.68851	0.70857	0.65793	0.71954
hsa-miR-501-5p	3	2	1	0.78111	0.80738	0.03887	0.55498	0.63404	0.55709	0.03832	0.69690	0.90980	0.64730
hsa-miR-532-3p	3	2	1	0.01262	0.00276	0.31762	0.24347	0.34540	0.53910	0.94538	0.02419	0.53141	0.31712
hsa-miR-134	3	2	1	0.07566	0.01255	0.10437	0.00149	0.20213	0.14383	0.06018	0.12349	0.02004	0.13493
hsa-miR-602	3	2	1	0.00273	0.00141	0.30755	0.83494	0.10000	0.88012	0.06356	0.88546	0.13323	0.45438
hsa-miR-18a	3	2	1	0.02389	0.08599	0.21935	0.02085	0.18095	0.63636	0.51891	0.01045	0.30977	0.17938
hsa-miR-28-5p	3	2	1	0.01790	0.05581	0.30945	0.03262	0.18077	0.53294	0.64048	0.04608	0.50534	0.54806
hsa-miR-381	3	2	1	0.92677	0.65546	0.85171	0.02444	0.79933	0.03689	0.28320	0.94181	0.55281	0.15876
hsa-miR-128	3	2	1	0.00926	0.04093	0.69845	0.31090	0.12685	0.43862	0.84444	0.02854	0.51552	0.22326

hsa-miR-1267	3	2	1	0.02622	0.00715	0.11785	0.35427	0.31460	0.86465	0.26447	0.85761	0.19638	0.72637
hsa-miR-3124	3	2	1	0.00137	0.02319	0.21051	0.06990	0.17115	0.19384	0.15843	0.03834	0.27043	0.43451
hsa-miR-1299	2	1	1	0.01053	0.49388	0.30951	0.07024	0.12249	0.72283	0.59033	0.86688	0.16719	0.40851
hsa-miR-4327	2	1	1	0.20548	0.51197	0.72709	0.01538	0.83990	0.13879	0.23082	0.19094	0.38502	0.59288
hsa-miR-892b	2	1	1	0.97787	0.87018	0.15629	0.60643	0.01250	0.38363	0.33412	0.88503	0.88554	0.96460
hsa-miR-564	2	1	1	0.42630	0.63225	0.65939	0.00413	0.24029	0.14754	0.08911	0.71439	0.38004	0.10595
hsa-miR-141	2	1	1	0.90891	0.69248	0.02650	0.97457	0.22796	0.17755	0.11213	0.43516	0.07012	0.05062
hsa-miR-1911	2	1	1	0.17577	0.01236	0.41741	0.09562	0.79618	0.95707	0.39963	0.61322	0.41328	0.68035
hsa-miR-320a	2	1	1	0.74965	0.48897	0.03950	0.24153	0.59139	0.87524	0.12890	0.16717	0.12773	0.12741
hsa-miR-124	2	1	1	0.00857	0.24356	0.42887	0.05273	0.26777	0.85461	0.10355	0.02491	0.09297	0.25783
hsa-miR-139-5p	2	1	1	0.02456	0.13818	0.13419	0.12598	0.38213	0.44713	0.59025	0.01301	0.11692	0.07685
hsa-miR-933	2	1	1	0.04891	0.09352	0.43099	0.35520	0.82203	0.44523	0.40090	0.71520	0.04824	0.28054
hsa-miR-324-5p	2	1	1	0.02884	0.11555	0.41669	0.05976	0.11655	0.30003	0.68723	0.02876	0.56047	0.25193
hsa-miR-99a	2	1	1	0.38044	0.85296	0.03893	0.96850	0.53066	0.60446	0.56167	0.18306	0.00747	0.16392
hsa-miR-3907	2	1	1	0.00418	0.91431	0.54632	0.15757	0.10851	0.36381	0.96917	0.04474	0.35294	0.64430
hsa-miR-625	2	1	1	0.01013	0.09765	0.16270	0.11106	0.29460	0.47606	0.47044	0.01723	0.68885	0.49183
hsa-miR-7	2	1	1	0.16078	0.07747	0.03164	0.36268	0.34128	0.17762	0.09894	0.02489	0.12536	0.11931
hsa-miR-33a	2	1	1	0.03402	0.14490	0.22930	0.19975	0.13949	0.25875	0.39827	0.04325	0.37847	0.12883
hsa-miR-1288	1	0	1	0.88453	0.64413	0.40205	0.37049	0.06447	0.19186	0.17557	0.13730	0.39009	0.26752
hsa-miR-1295	1	0	1	0.20393	0.43925	0.57910	0.27526	0.44459	0.60683	0.50081	0.24028	0.18392	0.98267
hsa-miR-4281	1	0	1	0.71674	0.86453	0.86894	0.71942	0.95278	0.84314	0.51089	0.91852	0.28618	0.92163
hsa-miR-500a	1	0	1	0.55365	0.78934	0.91719	0.11910	0.62744	0.93725	0.21393	0.60292	0.72718	0.93706
hsa-miR-497	1	0	1	0.92952	0.66453	0.91113	0.97388	0.50272	0.70929	0.96650	0.28418	0.03010	0.05687
hsa-miR-1290	1	0	1	0.86148	0.54234	0.39032	0.74179	0.57475	0.77999	0.19947	0.41287	0.49973	0.51780
hsa-miR-125b	1	0	1	0.14223	0.05439	0.62962	0.30238	0.58344	0.15724	0.42100	0.04896	0.13962	0.11174
hsa-miR-135b*	1	0	1	0.12234	0.09061	0.96922	0.32957	0.29049	0.40802	0.65064	0.87574	0.18966	0.47173
hsa-miR-520e	1	0	1	0.37959	0.38820	0.64946	0.53675	0.22214	0.80851	0.88630	0.87981	0.88205	0.21349
hsa-miR-486-5p	1	0	1	0.29864	0.72234	0.23441	0.60578	0.61917	0.61560	0.79773	0.34614	0.21192	0.03796
hsa-miR-96	1	0	1	0.32800	0.37472	0.76869	0.73763	0.93061	0.69399	0.95352	0.05361	0.10239	0.05289
hsa-miR-660	1	0	1	0.99888	0.32866	0.81308	0.95943	0.72924	0.92798	0.91481	0.06571	0.03708	0.13690
hsa-miR-10b	1	0	1	0.15934	0.29821	0.64415	0.98963	0.08378	0.38355	0.80402	0.85468	0.03610	0.39836
hsa-miR-100	1	0	1	0.20165	0.68812	0.65855	0.35455	0.64984	0.71867	0.77726	0.08574	0.02553	0.08177
hsa-miR-92a	1	0	1	0.16622	0.53004	0.88863	0.59666	0.95474	0.35213	0.59958	0.97243	0.19954	0.03431
hsa-miR-638	1	0	1	0.13226	0.13774	0.26856	0.24965	0.13316	0.49009	0.39091	0.04264	0.42857	0.30878
hsa-miR-502-3p	1	0	1	0.24833	0.10166	0.30407	0.71106	0.26766	0.46477	0.39181	0.20116	0.39149	0.04922
hsa-miR-192	1	0	1	0.97499	0.40885	0.67517	0.63248	0.64894	0.54148	0.45786	0.03856	0.13176	0.23288
hsa-miR-146b-5p	1	0	1	0.34478	0.30248	0.36990	0.12959	0.35940	0.33736	0.49060	0.03432	0.06751	0.25526
hsa-miR-195	1	0	1	0.05876	0.16941	0.73372	0.17924	0.34730	0.56640	0.54634	0.04480	0.23452	0.31298
hsa-miR-126*	1	0	1	0.37302	0.95006	0.91440	0.29575	0.79235	0.75833	0.76250	0.03582	0.36521	0.35404
hsa-miR-3152	7	7	0	0.00847	0.00673	0.02029	0.04261	0.01633	0.03592	0.01380	0.12230	0.33580	0.24971
hsa-miR-181b	6	6	0	0.02583	0.00394	0.04386	0.23787	0.00566	0.01524	0.01654	0.12015	0.33961	0.08915
hsa-miR-617	5	5	0	0.00078	0.02434	0.01910	0.52100	0.08395	0.04115	0.00342	0.12647	0.95426	0.57619
hsa-miR-361-3p	5	5	0	0.01768	0.00038	0.00823	0.05931	0.01710	0.00865	0.08101	0.05068	0.42957	0.23815
hsa-miR-370	4	4	0	0.03441	0.17200	0.03664	0.66529	0.02942	0.08769	0.00549	0.50335	0.85114	0.36837
hsa-miR-1470	4	4	0	0.00709	0.01440	0.03367	0.93735	0.01124	0.52042	0.10744	0.76682	0.20060	0.52517
hsa-miR-223*	4	4	0	0.01355	0.03749	0.21824	0.39249	0.03357	0.05484	0.04053	0.13399	0.88740	0.36188
hsa-miR-374c	4	4	0	0.00015	0.00055	0.02696	0.00641	0.19020	0.14070	0.28822	0.17925	0.76977	0.82469
hsa-miR-1303	4	4	0	0.02962	0.00422	0.03186	0.52911	0.12807	0.40394	0.01289	0.32312	0.20596	0.62957
hsa-miR-654-5p	4	4	0	0.00076	0.00114	0.17395	0.81389	0.07688	0.03319	0.03705	0.26685	0.78483	0.53654
hsa-miR-1181	4	4	0	0.30788	0.02210	0.54732	0.04447	0.02543	0.00384	0.81416	0.82485	0.68444	0.82146
hsa-miR-498	3	3	0	0.01913	0.01320	0.68940	0.07288	0.03947	0.06918	0.14257	0.56765	0.53134	0.66562
hsa-miR-181a	3	3	0	0.15192	0.05671	0.00865	0.02714	0.01143	0.08839	0.09723	0.17344	0.79403	0.08504
hsa-miR-338-3p	3	3	0	0.05079	0.02792	0.12278	0.02505	0.00731	0.06871	0.23804	0.06464	0.14363	0.05305
hsa-miR-940	3	3	0	0.00247	0.00726	0.05578	0.33201	0.00720	0.52025	0.33416	0.15644	0.64206	0.83990
hsa-miR-3646	3	3	0	0.02011	0.06858	0.05675	0.52938	0.01278	0.35490	0.04162	0.45767	0.81647	0.26873
hsa-miR-3190	3	3	0	0.00615	0.01811	0.01129	0.06968	0.14868	0.27815	0.78128	0.24063	0.43324	0.87464

hsa-miR-140-5p	3	3	0	0.02544	0.05692	0.41547	0.04202	0.11921	0.04321	0.18857	0.17072	0.43476	0.23624
hsa-miR-18b*	3	3	0	0.03805	0.00215	0.14605	0.88565	0.03470	0.45798	0.05759	0.47636	0.40013	0.21839
hsa-miR-125a-5p	3	3	0	0.01183	0.03256	0.12548	0.03648	0.15389	0.30987	0.30220	0.27906	0.55946	0.81898
hsa-miR-1471	3	3	0	0.01206	0.02886	0.40551	0.02147	0.34966	0.14297	0.10934	0.11335	0.10480	0.44447
hsa-miR-1287	3	3	0	0.00108	0.03262	0.87211	0.36598	0.03672	0.76872	0.44646	0.05496	0.22246	0.81912
hsa-miR-671-5p	3	3	0	0.02719	0.02273	0.31654	0.03998	0.26700	0.71332	0.11668	0.38111	0.49250	0.99069
hsa-miR-3196	3	3	0	0.22378	0.03182	0.09852	0.01046	0.28636	0.01604	0.23523	0.28887	0.14433	0.47462
hsa-miR-551b*	3	3	0	0.09611	0.03638	0.14893	0.93580	0.03343	0.08274	0.03647	0.50227	0.07309	0.82155
hsa-miR-1236	3	3	0	0.24196	0.75338	0.01855	0.14745	0.09698	0.00298	0.02761	0.95084	0.62064	0.68300
hsa-miR-663	3	3	0	0.00617	0.00182	0.35574	0.09078	0.01331	0.44948	0.32328	0.36107	0.30446	0.30618
hsa-miR-3185	3	3	0	0.04706	0.02369	0.25556	0.63702	0.34346	0.52411	0.00401	0.05952	0.54408	0.80459
hsa-miR-99b	3	3	0	0.01563	0.08944	0.17222	0.01921	0.03290	0.17673	0.20189	0.28476	0.62537	0.52888
hsa-miR-331-3p	3	3	0	0.00254	0.02816	0.17695	0.01588	0.18725	0.78517	0.68653	0.17106	0.82219	0.78752
hsa-miR-4253	3	3	0	0.00448	0.01695	0.51874	0.01932	0.09392	0.13472	0.49738	0.54855	0.68332	0.73053
hsa-miR-1180	3	3	0	0.68189	0.15029	0.04438	0.72799	0.04967	0.46636	0.00336	0.39131	0.49520	0.94085
hsa-miR-127-3p	3	3	0	0.03137	0.32146	0.01085	0.02814	0.24289	0.75189	0.97686	0.18754	0.46096	0.52941
hsa-miR-598	2	2	0	0.01400	0.02401	0.48418	0.31464	0.64788	0.10674	0.75701	0.07949	0.97630	0.81372
hsa-miR-762	2	2	0	0.27345	0.53030	0.56576	0.03745	0.26809	0.00766	0.24061	0.79566	0.85526	0.53538
hsa-miR-1306	2	2	0	0.11492	0.21041	0.02166	0.14657	0.06257	0.10692	0.00906	0.12849	0.38061	0.26918
hsa-miR-155	2	2	0	0.25139	0.15705	0.08233	0.07341	0.04448	0.25622	0.02693	0.08135	0.55114	0.54761
hsa-miR-557	2	2	0	0.10717	0.02890	0.07603	0.60656	0.04214	0.56924	0.19345	0.26938	0.45057	0.65689
hsa-miR-2861	2	2	0	0.01262	0.17243	0.04069	0.06966	0.32873	0.50224	0.51971	0.18140	0.87511	0.76605
hsa-miR-3186-3p	2	2	0	0.00345	0.02898	0.48420	0.22014	0.29654	0.55885	0.70769	0.96045	0.08416	0.42498
hsa-let-7f	2	2	0	0.03981	0.05833	0.19768	0.01490	0.12316	0.35498	0.25356	0.28663	0.91846	0.99946
hsa-let-7e	2	2	0	0.01307	0.13577	0.16082	0.04701	0.09340	0.06090	0.18421	0.14453	0.98919	0.75013
hsa-let-7a	2	2	0	0.04532	0.06628	0.26048	0.01352	0.15901	0.49264	0.27960	0.40072	0.96407	0.88420
hsa-miR-122	2	2	0	0.16159	0.03375	0.44686	0.26909	0.03493	0.94182	0.97776	0.33736	0.92284	0.52192
hsa-miR-23b	2	2	0	0.01998	0.17809	0.25537	0.02740	0.05543	0.19151	0.68922	0.33154	0.91845	0.96657
hsa-let-7d	2	2	0	0.03216	0.07826	0.22633	0.00697	0.11463	0.26000	0.21919	0.22546	0.92708	0.94361
hsa-miR-1224-3p	2	2	0	0.03985	0.55737	0.03101	0.11719	0.12507	0.70605	0.71232	0.06350	0.58305	0.43937
hsa-miR-3682	2	2	0	0.02606	0.01201	0.83705	0.08125	0.38988	0.48528	0.98800	0.65791	0.88588	0.94993
hsa-miR-181c	2	2	0	0.05786	0.02174	0.05658	0.10644	0.01504	0.17558	0.06652	0.19020	0.61021	0.08070
hsa-miR-4257	2	2	0	0.01479	0.00649	0.56386	0.31063	0.14993	0.66417	0.88756	0.92275	0.37897	0.44482
hsa-miR-3141	2	2	0	0.10419	0.03171	0.13098	0.00226	0.16174	0.13546	0.18686	0.17705	0.35006	0.36450
hsa-miR-382	2	2	0	0.08533	0.11397	0.01415	0.16340	0.03157	0.12032	0.05669	0.29377	0.49246	0.30240
hsa-miR-539	2	2	0	0.91446	0.83065	0.02214	0.22806	0.13630	0.30892	0.01202	0.14509	0.25214	0.22783
hsa-miR-33b*	2	2	0	0.02999	0.01266	0.31293	0.44571	0.24106	0.72278	0.22658	0.92816	0.41760	0.96866
hsa-miR-15b	2	2	0	0.06646	0.03009	0.47150	0.01671	0.26452	0.24119	0.32067	0.30534	0.76291	0.57280
hsa-miR-431	2	2	0	0.01463	0.03665	0.17779	0.12853	0.22257	0.24777	0.36714	0.23571	0.45630	0.75421
hsa-miR-374b	2	2	0	0.00977	0.07615	0.20624	0.01704	0.06812	0.39779	0.68528	0.08014	0.90231	0.69588
hsa-miR-486-3p	2	2	0	0.03830	0.04760	0.07870	0.18129	0.28369	0.77227	0.23494	0.06032	0.37824	0.45287
hsa-miR-4297	2	2	0	0.55515	0.71336	0.50184	0.01098	0.26298	0.04062	0.96692	0.26280	0.27640	0.87174
hsa-miR-3613-3p	2	2	0	0.10030	0.02412	0.09724	0.69236	0.18006	0.70218	0.04938	0.86443	0.40638	0.86742
hsa-miR-132	2	2	0	0.04682	0.01215	0.39459	0.67321	0.16266	0.18970	0.10205	0.40935	0.50973	0.43950
hsa-miR-505*	2	2	0	0.01722	0.04272	0.11848	0.13988	0.05907	0.42139	0.16137	0.09034	0.93880	0.51248
hsa-miR-151-5p	2	2	0	0.03931	0.20133	0.27744	0.01856	0.12869	0.43428	0.43798	0.06757	0.45841	0.52939
hsa-miR-1307	2	2	0	0.00418	0.11582	0.08212	0.67180	0.27234	0.69410	0.04777	0.15975	0.31790	0.34077
hsa-miR-129*	2	2	0	0.18597	0.01154	0.13505	0.39851	0.10939	0.46580	0.02676	0.75324	0.73170	0.82210
hsa-miR-513b	2	2	0	0.06127	0.76827	0.33846	0.49674	0.15537	0.02911	0.02122	0.20763	0.83486	0.34949
hsa-miR-200b*	2	2	0	0.03745	0.06445	0.06204	0.71301	0.22875	0.26220	0.01257	0.64302	0.27604	0.82651
hsa-miR-330-3p	2	2	0	0.02283	0.01766	0.26258	0.27701	0.08248	0.08365	0.17697	0.24294	0.92431	0.84114
hsa-miR-103	2	2	0	0.03673	0.11000	0.25778	0.01995	0.09281	0.52242	0.76473	0.30213	0.95853	0.82925
hsa-miR-3198	1	1	0	0.69748	0.03033	0.54906	0.71290	0.12191	0.58502	0.36490	0.77815	0.63741	0.74242
hsa-miR-7-1*	1	1	0	0.03205	0.34428	0.32578	0.40422	0.28335	0.37520	0.58958	0.14399	0.67400	0.61460
hsa-miR-3937	1	1	0	0.05547	0.05830	0.80652	0.12009	0.05790	0.04350	0.68651	0.72816	0.61081	0.27604
hsa-miR-148a	1	1	0	0.04393	0.45471	0.54734	0.87776	0.41947	0.41580	0.86312	0.29977	0.86470	0.99551

hsa-miR-3663-3p	1	1	0	0.02793	0.05667	0.33901	0.10874	0.07924	0.18599	0.10519	0.09377	0.61482	0.19558
hsa-miR-939	1	1	0	0.04385	0.09152	0.68511	0.09815	0.14564	0.29145	0.61939	0.50407	0.95522	0.66750
hsa-let-7d*	1	1	0	0.28849	0.21050	0.99199	0.27179	0.60560	0.00690	0.57880	0.08692	0.29088	0.21539
hsa-miR-574-3p	1	1	0	0.21360	0.99594	0.12710	0.34282	0.16509	0.37396	0.04667	0.25073	0.84419	0.28907
hsa-miR-4298	1	1	0	0.16738	0.06054	0.16152	0.01499	0.22760	0.24076	0.23170	0.24844	0.15143	0.29103
hsa-miR-551b	1	1	0	0.60872	0.08208	0.30274	0.04539	0.47612	0.19725	0.14812	0.38833	0.70454	0.94700
hsa-miR-513a-5p	1	1	0	0.39715	0.14348	0.02767	0.09191	0.09384	0.58041	0.56865	0.38861	0.87168	0.48143
hsa-miR-30d	1	1	0	0.21582	0.50391	0.04315	0.53116	0.39729	0.76209	0.67032	0.46615	0.53492	0.17816
hsa-miR-483-5p	1	1	0	0.95665	0.16780	0.22702	0.01377	0.81709	0.30690	0.39778	0.27407	0.15729	0.32180
hsa-miR-320c	1	1	0	0.03647	0.07862	0.43276	0.42712	0.10953	0.27087	0.31255	0.27702	0.24523	0.75112
hsa-miR-765	1	1	0	0.77847	0.48122	0.75145	0.04222	0.72899	0.48540	0.86055	0.57213	0.16852	0.38856
hsa-miR-3945	1	1	0	0.03355	0.30406	0.42326	0.86513	0.22824	0.66839	0.16427	0.40805	0.22660	0.46395
hsa-miR-422a	1	1	0	0.19785	0.29338	0.11351	0.48592	0.51817	0.53202	0.02341	0.27822	0.53699	0.67897
hsa-miR-1915*	1	1	0	0.85177	0.69003	0.36154	0.25278	0.57921	0.02601	0.74602	0.52601	0.94867	0.88394
hsa-let-7c	1	1	0	0.06197	0.10176	0.19301	0.03593	0.41809	0.52553	0.17811	0.05947	0.18855	0.29697
hsa-miR-19b	1	1	0	0.84968	0.17603	0.67035	0.03177	0.29904	0.49819	0.45967	0.21461	0.16249	0.15114
hsa-miR-1	1	1	0	0.02163	0.21379	0.30116	0.17873	0.08303	0.16694	0.24453	0.11071	0.47079	0.74739
hsa-miR-129-3p	1	1	0	0.21155	0.00852	0.15691	0.61985	0.07026	0.72973	0.17025	0.63994	0.96247	0.96364
hsa-miR-518e*	1	1	0	0.01796	0.37385	0.59927	0.50666	0.73940	0.83661	0.79999	0.84767	0.14852	0.27842
hsa-miR-1228*	1	1	0	0.76616	0.29942	0.11849	0.87607	0.16373	0.84965	0.02202	0.08063	0.96557	0.47271
hsa-miR-1202	1	1	0	0.73342	0.01412	0.80077	0.80848	0.89313	0.86124	0.61074	0.80872	0.82995	0.82167
hsa-miR-1238	1	1	0	0.08580	0.03613	0.45743	0.80084	0.16602	0.89509	0.42937	0.82369	0.30535	0.53862
hsa-miR-3180-5p	1	1	0	0.26977	0.02822	0.95036	0.34118	0.86889	0.30440	0.60505	0.67004	0.36659	0.36568
hsa-miR-3189	1	1	0	0.90714	0.80716	0.10891	0.50346	0.40306	0.22809	0.01115	0.49762	0.27579	0.56651
hsa-miR-3176	1	1	0	0.10412	0.87308	0.48166	0.02531	0.80023	0.70682	0.49500	0.34524	0.75033	0.33537
hsa-miR-142-3p	1	1	0	0.36647	0.06602	0.20443	0.01966	0.14270	0.09629	0.36027	0.37870	0.35520	0.53738
hsa-miR-300	1	1	0	0.20867	0.25244	0.24369	0.04418	0.44217	0.32136	0.73205	0.18120	0.48942	0.89607
hsa-miR-4313	1	1	0	0.09885	0.02685	0.23079	0.50888	0.22847	0.61128	0.27513	0.83454	0.24165	0.60614
hsa-miR-610	1	1	0	0.36840	0.44702	0.05696	0.75685	0.34160	0.30900	0.03865	0.17947	0.87814	0.55634
hsa-miR-151-3p	1	1	0	0.08838	0.24411	0.04280	0.14695	0.07586	0.58387	0.66871	0.14879	0.52342	0.18470
hsa-miR-23a*	1	1	0	0.08376	0.44155	0.22374	0.51511	0.09176	0.09810	0.01251	0.68029	0.41348	0.96191
hsa-miR-563	1	1	0	0.21436	0.02896	0.74997	0.25590	0.46979	0.68896	0.48626	0.83204	0.21487	0.70194
hsa-miR-1254	1	1	0	0.97573	0.83191	0.53881	0.04256	0.88867	0.14849	0.60285	0.47304	0.17085	0.46424
hsa-miR-3651	1	1	0	0.01888	0.17192	0.68912	0.95261	0.06649	0.17240	0.26696	0.79757	0.15241	0.54204
hsa-miR-3162	1	1	0	0.18678	0.00415	0.21570	0.13955	0.22590	0.47160	0.46073	0.18074	0.66783	0.95356
hsa-miR-663b	1	1	0	0.17142	0.01559	0.14501	0.19323	0.40847	0.61082	0.50869	0.38449	0.56547	0.73138
hsa-miR-3200-5p	1	1	0	0.61173	0.38463	0.44038	0.02540	0.63789	0.33082	0.58759	0.54751	0.51019	0.70147
hsa-miR-23c	1	1	0	0.11410	0.01831	0.56922	0.56554	0.24047	0.83999	0.60406	0.95360	0.42411	0.78177
hsa-miR-3622a-5p	1	1	0	0.10399	0.22163	0.97810	0.03788	0.52328	0.54792	0.15541	0.95278	0.43225	0.80326
hsa-miR-379	1	1	0	0.06664	0.07510	0.03144	0.14765	0.06369	0.28390	0.10080	0.27854	0.52500	0.27510
hsa-miR-550a*	1	1	0	0.08288	0.02438	0.58645	0.14809	0.47038	0.94442	0.80060	0.20852	0.50370	0.99985
hsa-miR-378b	1	1	0	0.44029	0.79909	0.95281	0.69837	0.27897	0.78703	0.04853	0.67863	0.84040	0.77982
hsa-miR-423-5p	1	1	0	0.41484	0.13122	0.01037	0.50040	0.16883	0.86866	0.11894	0.07050	0.36424	0.17023
hsa-miR-223	1	1	0	0.04192	0.19182	0.97734	0.05117	0.51289	0.32587	0.47242	0.27585	0.89522	0.55757
hsa-miR-1268	1	1	0	0.29885	0.11594	0.37250	0.02458	0.73459	0.34974	0.71940	0.47507	0.16907	0.23421
hsa-miR-20a	1	1	0	0.11981	0.09917	0.39951	0.01546	0.38433	0.37287	0.33585	0.24198	0.59149	0.57281
hsa-miR-758	1	1	0	0.65852	0.20174	0.82063	0.00891	0.67180	0.86084	0.65278	0.33978	0.50002	0.32203
hsa-miR-3617	1	1	0	0.38832	0.37385	0.17923	0.01332	0.09974	0.07964	0.70016	0.63950	0.44413	0.80911
hsa-miR-648	1	1	0	0.10550	0.01867	0.10524	0.26269	0.17156	0.18464	0.13071	0.47987	0.27795	0.69817
hsa-miR-301b	1	1	0	0.03196	0.13884	0.17238	0.06650	0.11484	0.42977	0.93821	0.08278	0.87515	0.28289
hsa-miR-609	1	1	0	0.07275	0.08630	0.45753	0.01045	0.30394	0.38606	0.83458	0.07782	0.92508	0.55051
hsa-miR-199a-5p	1	1	0	0.03514	0.29666	0.37167	0.13727	0.21059	0.66929	0.73183	0.05249	0.49898	0.63963
hsa-miR-425*	1	1	0	0.14314	0.01321	0.46866	0.56354	0.27467	0.72018	0.31137	0.89688	0.59401	0.99418
hsa-miR-340*	1	1	0	0.10268	0.38746	0.12966	0.01714	0.68345	0.77231	0.67222	0.19964	0.56969	0.71453
hsa-miR-191*	1	1	0	0.21589	0.03426	0.64170	0.61478	0.26906	0.60989	0.64262	0.98447	0.51445	0.88337
hsa-miR-3676	1	1	0	0.40812	0.02563	0.77805	0.34077	0.72604	0.41703	0.87037	0.78834	0.44680	0.80922

hsa-miR-145	1	1	0	0.00659	0.06756	0.90167	0.29947	0.35597	0.45196	0.19225	0.17280	0.95818	0.30418
hsa-miR-21	1	1	0	0.14375	0.37842	0.63433	0.03741	0.39347	0.34129	0.97321	0.20704	0.40059	0.74725
hsa-miR-449c*	1	1	0	0.13049	0.26313	0.30106	0.00276	0.27623	0.06230	0.56427	0.11374	0.86929	0.69360
hsa-miR-4299	1	1	0	0.12677	0.54430	0.03679	0.69913	0.83140	0.61128	0.78538	0.93817	0.97851	0.34134
hsa-miR-1273d	1	1	0	0.04679	0.86609	0.52259	0.40732	0.31944	0.55232	0.61017	0.92164	0.52728	0.87225
hsa-miR-670	1	1	0	0.97700	0.36532	0.10938	0.31940	0.07456	0.16764	0.02158	0.24503	0.33236	0.56045
hsa-miR-26b	1	1	0	0.23536	0.09857	0.43103	0.03535	0.37613	0.43248	0.31276	0.18260	0.62101	0.68582
hsa-miR-150	1	1	0	0.01476	0.15893	0.23135	0.59390	0.10631	0.41138	0.23815	0.42502	0.18222	0.69542
hsa-miR-339-3p	1	1	0	0.68573	0.80797	0.35714	0.00490	0.37726	0.11603	0.22043	0.49797	0.58358	0.88954
hsa-let-7f-1*	1	1	0	0.24769	0.01923	0.11185	0.64540	0.27974	0.78196	0.08048	0.77460	0.60561	0.80950
hsa-let-7g	1	1	0	0.17953	0.13560	0.39169	0.02543	0.29583	0.57715	0.36908	0.24378	0.77241	0.87326
hsa-miR-1227	1	1	0	0.50701	0.10598	0.01453	0.50695	0.25881	0.32099	0.16356	0.71960	0.95436	0.58540
hsa-miR-631	1	1	0	0.04199	0.85614	0.07419	0.46528	0.22457	0.10884	0.10934	0.15559	0.86051	0.51992
hsa-miR-30b	1	1	0	0.07578	0.13821	0.43168	0.02314	0.25994	0.78271	0.52540	0.20822	0.94135	0.91084
hsa-miR-3180-3p	1	1	0	0.00829	0.20866	0.10670	0.39758	0.13723	0.26569	0.06720	0.23946	0.85326	0.89058
hsa-miR-887	1	1	0	0.75180	0.76200	0.90530	0.00552	0.81778	0.34755	0.85342	0.78701	0.56094	0.92198
hsa-miR-1234	1	1	0	0.12951	0.02864	0.33266	0.77740	0.15928	0.66806	0.43914	0.89358	0.61069	0.95312
hsa-miR-3622b-5p	1	1	0	0.54370	0.39463	0.55118	0.04841	0.69950	0.79828	0.52465	0.63188	0.32037	0.68072
hsa-miR-193a-5p	1	1	0	0.00620	0.66347	0.85674	0.32738	0.42020	0.88779	0.51797	0.18316	0.25953	0.24811
hsa-miR-202	1	1	0	0.80704	0.02677	0.06200	0.07918	0.87696	0.75452	0.26623	0.21870	0.17854	0.60340
hsa-miR-212	1	1	0	0.02673	0.93599	0.78371	0.44492	0.62078	0.51083	0.90850	0.58435	0.14365	0.35352
hsa-miR-1228	1	1	0	0.18307	0.04257	0.88666	0.93973	0.17837	0.65422	0.65469	0.91733	0.62310	0.87810
hsa-miR-342-5p	1	1	0	0.46326	0.34448	0.02522	0.16728	0.68097	0.51103	0.05683	0.54453	0.53687	0.47513
hsa-let-7i	1	1	0	0.15830	0.11085	0.50092	0.02385	0.26094	0.55009	0.43005	0.17480	0.50327	0.70141
hsa-miR-1225-3p	1	1	0	0.08193	0.03421	0.50959	0.72652	0.14838	0.55905	0.17960	0.83475	0.59153	0.86306
hsa-miR-936	1	1	0	0.01944	0.70780	0.52745	0.48486	0.52911	0.92873	0.12129	0.65466	0.75911	0.87589
hsa-miR-550a	1	1	0	0.13884	0.03432	0.18740	0.26422	0.73102	0.77744	0.27375	0.79556	0.34929	0.49411
hsa-miR-4306	0	0	0	0.27775	0.39632	0.65028	0.26195	0.54292	0.09271	0.40467	0.60514	0.64306	0.11417
hsa-miR-4270	0	0	0	0.27241	0.76623	0.86720	0.32562	0.34010	0.96007	0.81266	0.92001	0.36768	0.26724
hsa-miR-1305	0	0	0	0.77910	0.12034	0.53089	0.62390	0.09343	0.44347	0.23018	0.79409	0.95114	0.93196
hsa-miR-642b	0	0	0	0.35739	0.14299	0.64771	0.96161	0.17983	0.69178	0.47623	0.96283	0.35323	0.65950
hsa-miR-30c-2*	0	0	0	0.16912	0.90462	0.91879	0.71852	0.34047	0.92992	0.25981	0.95346	0.16291	0.38833
hsa-miR-634	0	0	0	0.72945	0.17542	0.88448	0.62465	0.23624	0.80227	0.47169	0.50001	0.15802	0.61477
hsa-miR-1323	0	0	0	0.89210	0.56593	0.70818	0.59790	0.50822	0.23790	0.71894	0.84615	0.20494	0.94460
hsa-miR-125b-1*	0	0	0	0.06809	0.16321	0.41159	0.86494	0.07429	0.64067	0.42172	0.18865	0.55318	0.74440
hsa-miR-184	0	0	0	0.57064	0.35133	0.32904	0.13897	0.70006	0.12529	0.12028	0.66325	0.06931	0.07836
hsa-miR-3125	0	0	0	0.57259	0.87795	0.05771	0.59319	0.84203	0.27150	0.12033	0.44049	0.74638	0.94876
hsa-miR-711	0	0	0	0.86472	0.29548	0.41171	0.09191	0.69273	0.21272	0.78242	0.76420	0.33438	0.82287
hsa-miR-23a	0	0	0	0.45933	0.67018	0.20678	0.69996	0.86024	0.91039	0.43084	0.76630	0.82572	0.63693
hsa-miR-2276	0	0	0	0.87039	0.93589	0.95861	0.17582	0.69531	0.41717	0.80026	0.92614	0.13900	0.89305
hsa-miR-548c-5p	0	0	0	0.53652	0.08534	0.24638	0.50875	0.31958	0.62076	0.75877	0.15071	0.46443	0.62049
hsa-miR-198	0	0	0	0.22353	0.48299	0.44912	0.41304	0.38655	0.60346	0.44298	0.57108	0.19679	0.48316
hsa-miR-3202	0	0	0	0.06826	0.82707	0.60951	0.82918	0.38285	0.55839	0.32406	0.88480	0.25663	0.40918
hsa-miR-193b*	0	0	0	0.49127	0.05162	0.45659	0.08220	0.36979	0.67843	0.75877	0.58898	0.55625	0.29074
hsa-miR-2278	0	0	0	0.40528	0.93877	0.14425	0.99766	0.78038	0.81361	0.14222	0.07663	0.43288	0.22478
hsa-miR-3923	0	0	0	0.42423	0.34507	0.52269	0.31135	0.89180	0.74977	0.27542	0.75366	0.93473	0.90960
hsa-miR-365	0	0	0	0.17973	0.08517	0.32686	0.62387	0.90130	0.77385	0.08760	0.63182	0.93230	0.69956
hsa-miR-516b	0	0	0	0.22489	0.67601	0.32881	0.55436	0.36761	0.26901	0.11193	0.94231	0.46092	0.80721
hsa-miR-516a-5p	0	0	0	0.83987	0.67504	0.31676	0.59961	0.95038	0.56911	0.10074	0.56791	0.69027	0.46366
hsa-miR-514b-5p	0	0	0	0.06525	0.93993	0.72175	0.61460	0.40990	0.79391	0.47501	0.98892	0.29813	0.53919
hsa-miR-3154	0	0	0	0.85442	0.25252	0.88409	0.30717	0.37622	0.35714	0.19645	0.32413	0.27687	0.23942
hsa-miR-3652	0	0	0	0.79138	0.34841	0.63008	0.16138	0.73776	0.34642	0.48028	0.45470	0.72666	0.98869
hsa-miR-566	0	0	0	0.87190	0.20287	0.23736	0.34322	0.86851	0.11778	0.79820	0.86386	0.37930	0.43040
hsa-miR-636	0	0	0	0.61997	0.07560	0.87880	0.81178	0.27552	0.82001	0.77238	0.96249	0.86374	0.92184
hsa-miR-187*	0	0	0	0.34011	0.41127	0.80082	0.13650	0.33098	0.11187	0.68255	0.97669	0.71776	0.22609
hsa-miR-3127	0	0	0	0.15384	0.08345	0.47764	0.06654	0.17540	0.11650	0.42735	0.89687	0.56874	0.84782

hsa-miR-3610	0	0	0	0.20462	0.33454	0.51568	0.11942	0.14367	0.21732	0.48273	0.90555	0.50436	0.56052
hsa-miR-29c	0	0	0	0.87100	0.50228	0.96497	0.58539	0.17223	0.26189	0.90236	0.93983	0.29725	0.05924
hsa-miR-296-5p	0	0	0	0.38858	0.63691	0.41770	0.74170	0.90734	0.83859	0.55019	0.60951	0.28929	0.58232
hsa-miR-885-5p	0	0	0	0.96455	0.62624	0.42689	0.80575	0.39103	0.62849	0.63001	0.30806	0.58987	0.38609
hsa-miR-4294	0	0	0	0.46362	0.38147	0.30294	0.11909	0.15370	0.55462	0.42613	0.62753	0.76353	0.53369
hsa-miR-3074	0	0	0	0.51866	0.47065	0.86260	0.22939	0.66699	0.07933	0.92858	0.81172	0.72235	0.72334
hsa-miR-378	0	0	0	0.79170	0.19625	0.45368	0.24992	0.28017	0.31791	0.09635	0.96004	0.73449	0.95004
hsa-miR-483-3p	0	0	0	0.91324	0.56253	0.86140	0.62207	0.88646	0.61489	0.85693	0.35555	0.58448	0.67804
hsa-miR-629	0	0	0	0.05134	0.11833	0.53049	0.41695	0.13985	0.53116	0.17708	0.25795	0.28882	0.33470
hsa-miR-1183	0	0	0	0.80659	0.55426	0.23331	0.50295	0.82207	0.99842	0.40755	0.27819	0.74234	0.73523
hsa-miR-1260b	0	0	0	0.44527	0.62181	0.42421	0.15374	0.52570	0.61593	0.37554	0.89454	0.40510	0.66376
hsa-miR-1225-5p	0	0	0	0.13873	0.91325	0.79467	0.72121	0.43158	0.57727	0.91758	0.32603	0.95473	0.87315
hsa-miR-764	0	0	0	0.94540	0.32918	0.62773	0.55425	0.70003	0.74126	0.68572	0.55625	0.33559	0.53546
hsa-miR-363	0	0	0	0.50649	0.17982	0.43304	0.20685	0.43504	0.51579	0.36095	0.06202	0.14546	0.13140
hsa-miR-125a-3p	0	0	0	0.40077	0.19893	0.51298	0.27182	0.96579	0.94951	0.89663	0.65651	0.19023	0.65317
hsa-miR-199a-3p	0	0	0	0.14570	0.54855	0.36389	0.22120	0.73272	0.50811	0.67830	0.26203	0.88684	0.68665
hsa-miR-623	0	0	0	0.21932	0.97359	0.35248	0.57617	0.72206	0.49269	0.08623	0.77766	0.67517	0.73535
hsa-miR-494	0	0	0	0.43544	0.68926	0.12681	0.89176	0.50833	0.24086	0.05663	0.29824	0.47062	0.19100
hsa-miR-1275	0	0	0	0.52219	0.88324	0.65370	0.10939	0.53221	0.80727	0.91414	0.85435	0.15325	0.59727
hsa-miR-1226*	0	0	0	0.89594	0.55143	0.60524	0.26739	0.89511	0.37028	0.97063	0.91825	0.12408	0.40991
hsa-miR-659	0	0	0	0.13625	0.12062	0.34079	0.88989	0.24820	0.43178	0.23138	0.54879	0.44111	0.63300
hsa-miR-654-3p	0	0	0	0.40152	0.29540	0.65660	0.33380	0.75328	0.14144	0.45982	0.34802	0.40023	0.34686
hsa-miR-20b	0	0	0	0.13240	0.10141	0.26783	0.06289	0.48239	0.48748	0.23685	0.07418	0.16441	0.26668
hsa-miR-3713	0	0	0	0.34111	0.61697	0.15160	0.71891	0.65649	0.19438	0.11974	0.44129	0.51571	0.89633
hsa-miR-520b	0	0	0	0.97511	0.12864	0.86419	0.68947	0.33115	0.52499	0.25701	0.58759	0.91295	0.87612
hsa-miR-3138	0	0	0	0.74442	0.14719	0.60722	0.16013	0.92305	0.17658	0.56020	0.78954	0.32773	0.25320
hsa-miR-4271	0	0	0	0.18363	0.06838	0.42149	0.06719	0.79541	0.91160	0.34893	0.54090	0.27607	0.74593
hsa-miR-572	0	0	0	0.15533	0.09147	0.23271	0.44130	0.34891	0.74573	0.52254	0.06910	0.65037	0.39910
hsa-miR-532-5p	0	0	0	0.38282	0.11459	0.64922	0.44969	0.44523	0.66184	0.45133	0.07252	0.30029	0.11798
hsa-miR-342-3p	0	0	0	0.30046	0.98486	0.76360	0.32404	0.86283	0.74800	0.84233	0.44701	0.34440	0.27303
hsa-miR-135a*	0	0	0	0.57953	0.96199	0.33893	0.51147	0.77388	0.26594	0.75363	0.82801	0.39423	0.93090
hsa-miR-1249	0	0	0	0.92033	0.28076	0.80153	0.30044	0.77957	0.51946	0.98937	0.86451	0.53575	0.57038
hsa-miR-877	0	0	0	0.94574	0.58180	0.45893	0.26813	0.20426	0.46442	0.38564	0.56579	0.36971	0.94946
hsa-miR-320e	0	0	0	0.44663	0.73506	0.19355	0.53037	0.86200	0.27707	0.84851	0.24145	0.18429	0.06699
hsa-miR-3149	0	0	0	0.23209	0.22739	0.70380	0.21189	0.41194	0.72714	0.68231	0.79569	0.97254	0.55178
hsa-miR-622	0	0	0	0.30891	0.12704	0.61473	0.19911	0.84327	0.62043	0.73406	0.54502	0.17084	0.34004
hsa-miR-1182	0	0	0	0.32831	0.68654	0.47231	0.07461	0.57740	0.77917	0.59927	0.83566	0.25500	0.82353
hsa-miR-424	0	0	0	0.12450	0.13124	0.92067	0.74531	0.21133	0.44277	0.82003	0.16899	0.36627	0.21061
hsa-miR-3692*	0	0	0	0.97863	0.80070	0.30983	0.54200	0.43106	0.49947	0.37180	0.79898	0.25574	0.71647
hsa-miR-133b	0	0	0	0.61086	0.92936	0.95873	0.37486	0.35851	0.83970	0.15015	0.51826	0.64284	0.63941
hsa-miR-1237	0	0	0	0.18592	0.07219	0.53756	0.32998	0.46356	0.47762	0.18171	0.74421	0.29297	0.49950
hsa-miR-769-3p	0	0	0	0.21547	0.51873	0.52740	0.93803	0.40014	0.58350	0.21982	0.75619	0.18501	0.73858
hsa-miR-136	0	0	0	0.60283	0.76366	0.21893	0.27612	0.43657	0.82829	0.30063	0.78426	0.37812	0.26223
hsa-miR-3926	0	0	0	0.57454	0.55323	0.06600	0.21272	0.31195	0.67921	0.86972	0.55363	0.49659	0.56940
hsa-miR-1469	0	0	0	0.44886	0.25274	0.36857	0.93915	0.17467	0.39896	0.09530	0.28818	0.54693	0.88141
hsa-miR-185	0	0	0	0.25238	0.25693	0.52459	0.25734	0.41532	0.65598	0.78840	0.07706	0.32965	0.12834
hsa-miR-575	0	0	0	0.29708	0.57908	0.54536	0.50097	0.68190	0.85136	0.76854	0.73016	0.80375	0.62750
hsa-miR-3917	0	0	0	0.65614	0.56863	0.75098	0.06484	0.75805	0.57835	0.84940	0.78085	0.46395	0.83270
hsa-miR-4274	0	0	0	0.75240	0.79296	0.72741	0.84242	0.97492	0.88853	0.31758	0.42906	0.91815	0.69672
hsa-miR-4290	0	0	0	0.47782	0.84885	0.05259	0.46811	0.13279	0.48236	0.05770	0.62916	0.63709	0.39826
hsa-miR-1208	0	0	0	0.05040	0.26723	0.22089	0.05411	0.13817	0.12247	0.60879	0.22741	0.31076	0.79777
hsa-miR-93	0	0	0	0.44199	0.21647	0.40042	0.18313	0.23454	0.84846	0.54862	0.41291	0.65828	0.08574
hsa-miR-139-3p	0	0	0	0.54050	0.32472	0.26394	0.47407	0.88657	0.42372	0.93780	0.36282	0.36747	0.44841
hsa-miR-24	0	0	0	0.25813	0.94712	0.62407	0.48381	0.50419	0.65158	0.19222	0.66911	0.81451	0.69909
hsa-miR-22*	0	0	0	0.59594	0.88221	0.87504	0.72749	0.88303	0.95785	0.64605	0.63944	0.28631	0.76605
hsa-miR-320d	0	0	0	0.19888	0.78654	0.30663	0.33026	0.78857	0.36590	0.90188	0.62457	0.13881	0.10315

hsa-miR-3665	0	0	0	0.16195	0.13526	0.61541	0.41477	0.37501	0.64316	0.95745	0.59404	0.50363	0.82455
hsa-miR-197	0	0	0	0.64715	0.30004	0.62084	0.67740	0.87978	0.93331	0.82254	0.35683	0.28164	0.70830
hsa-miR-196b	0	0	0	0.52954	0.56490	0.91005	0.92646	0.69988	0.50708	0.37241	0.98946	0.48032	0.28991
hsa-miR-371-5p	0	0	0	0.83694	0.50406	0.05269	0.61250	0.48892	0.08143	0.15126	0.75135	0.36954	0.43244
hsa-miR-4259	0	0	0	0.75117	0.85771	0.89715	0.42088	0.78303	0.87808	0.63773	0.78931	0.54240	0.85721
hsa-miR-1280	0	0	0	0.32532	0.09087	0.97818	0.22742	0.40434	0.56390	0.90840	0.80559	0.34493	0.59724
hsa-miR-584	0	0	0	0.29314	0.63283	0.44375	0.75335	0.46426	0.73751	0.70877	0.86865	0.53040	0.34076
hsa-miR-30c-1*	0	0	0	0.08153	0.34463	0.37074	0.57890	0.25671	0.78841	0.17635	0.43608	0.21791	0.47497
hsa-miR-224	0	0	0	0.06791	0.24258	0.21214	0.10788	0.51633	0.39923	0.12625	0.18029	0.58956	0.40026
hsa-miR-149*	0	0	0	0.09203	0.18301	0.12400	0.60269	0.39878	0.42037	0.05927	0.49537	0.35281	0.92666
hsa-miR-595	0	0	0	0.60731	0.66751	0.11942	0.21775	0.26911	0.40795	0.13931	0.47355	0.49475	0.93414
hsa-miR-543	0	0	0	0.37269	0.36780	0.70994	0.06341	0.66109	0.59794	0.12314	0.63868	0.80523	0.54608
hsa-miR-3131	0	0	0	0.91858	0.44585	0.62880	0.60805	0.44751	0.71433	0.21877	0.56939	0.62679	0.63214
hsa-miR-16	0	0	0	0.97715	0.23989	0.28820	0.17143	0.65819	0.76800	0.27080	0.14488	0.05988	0.21407
hsa-miR-10b*	0	0	0	0.76515	0.64673	0.32827	0.86264	0.54441	0.19441	0.09783	0.47781	0.97248	0.90163
hsa-miR-3667-5p	0	0	0	0.55813	0.44820	0.80900	0.19989	0.74765	0.43309	0.36531	0.85874	0.17695	0.35368
hsa-miR-3614-5p	0	0	0	0.38353	0.14927	0.65288	0.85277	0.84467	0.77635	0.55273	0.95741	0.95971	0.90665
hsa-miR-337-3p	0	0	0	0.70126	0.97608	0.67276	0.72882	0.18298	0.33579	0.29366	0.80064	0.90864	0.48445
hsa-miR-186*	0	0	0	0.91137	0.07673	0.58333	0.18621	0.64707	0.83911	0.52723	0.62626	0.58679	0.71368
hsa-miR-665	0	0	0	0.28699	0.10644	0.93961	0.63535	0.68020	0.59018	0.83945	0.56686	0.77947	0.58599
hsa-miR-505	0	0	0	0.06402	0.41786	0.90887	0.27190	0.35194	0.89543	0.84987	0.08484	0.49427	0.39693
hsa-miR-3195	0	0	0	0.07221	0.05725	0.48201	0.07978	0.08631	0.45709	0.46780	0.37088	0.35331	0.39796
hsa-miR-32*	0	0	0	0.14339	0.07964	0.88422	0.42647	0.94579	0.80485	0.95816	0.44677	0.80969	0.74919
hsa-miR-1260	0	0	0	0.45960	0.90354	0.90671	0.37177	0.58759	0.75908	0.79602	0.31741	0.75927	0.74940
hsa-miR-548q	0	0	0	0.60807	0.16844	0.71135	0.90999	0.61903	0.31754	0.48271	0.48452	0.27451	0.93684
hsa-miR-582-5p	0	0	0	0.84031	0.69630	0.42043	0.61835	0.33625	0.39205	0.83446	0.05991	0.97365	0.23893
hsa-miR-3654	0	0	0	0.72721	0.99540	0.25095	0.39345	0.33857	0.27441	0.05981	0.52522	0.81978	0.78758
hsa-miR-3679-5p	0	0	0	0.14464	0.96265	0.71827	0.60203	0.46136	0.07223	0.23743	0.36916	0.90484	0.73147
hsa-miR-1972	0	0	0	0.22473	0.73567	0.33495	0.10966	0.44508	0.93919	0.91493	0.77200	0.26911	0.57289
hsa-miR-152	0	0	0	0.13435	0.27143	0.76488	0.63716	0.70652	0.79460	0.92254	0.06483	0.21067	0.43552
hsa-miR-335	0	0	0	0.37244	0.42787	0.62521	0.37344	0.50706	0.82749	0.81178	0.23442	0.54145	0.55560
hsa-miR-4322	0	0	0	0.43189	0.76950	0.30102	0.68458	0.90064	0.84620	0.17513	0.63816	0.43805	0.83347
hsa-miR-493*	0	0	0	0.21034	0.88581	0.30072	0.99094	0.98396	0.64269	0.32309	0.40157	0.62959	0.97488
hsa-miR-708	0	0	0	0.24995	0.07576	0.38697	0.25661	0.52069	0.84664	0.17007	0.67534	0.62424	0.82331
hsa-miR-500a*	0	0	0	0.97757	0.12599	0.62713	0.94252	0.45068	0.75716	0.39673	0.36338	0.30978	0.05011
hsa-miR-1274b	0	0	0	0.76279	0.11855	0.61831	0.66976	0.98042	0.77193	0.83796	0.35743	0.85757	0.62582
hsa-miR-183	0	0	0	0.55459	0.15614	0.49202	0.32262	0.58946	0.38258	0.34981	0.18778	0.98005	0.33883
hsa-miR-376c	0	0	0	0.44731	0.71509	0.45317	0.22206	0.26120	0.85409	0.87853	0.21189	0.36491	0.33320
hsa-miR-4312	0	0	0	0.28498	0.05906	0.76644	0.42707	0.58623	0.82059	0.87541	0.96391	0.66307	0.86162
hsa-miR-766	0	0	0	0.84612	0.77173	0.94166	0.09853	0.89382	0.64848	0.58081	0.90587	0.90699	0.65181
hsa-miR-1914*	0	0	0	0.25000	0.61689	0.17848	0.72790	0.83433	0.60526	0.05659	0.65638	0.89308	0.84972
hsa-miR-3675-3p	0	0	0	0.30829	0.12079	0.29200	0.15851	0.39010	0.80657	0.39348	0.69358	0.39293	0.84761
hsa-miR-4314	0	0	0	0.21858	0.66270	0.22707	0.45784	0.88687	0.58228	0.11647	0.61199	0.56476	0.81410
hsa-miR-3188	0	0	0	0.05107	0.06867	0.36808	0.09812	0.18938	0.23384	0.33061	0.27561	0.09321	0.42419
hsa-miR-601	0	0	0	0.67178	0.15789	0.75956	0.22991	0.17013	0.26220	0.82564	0.99358	0.38602	0.94454
hsa-miR-542-5p	0	0	0	0.06367	0.10079	0.62022	0.34407	0.17624	0.14638	0.07356	0.90790	0.71435	0.91859
hsa-miR-376a	0	0	0	0.40878	0.57358	0.32151	0.24065	0.18317	0.78533	0.86022	0.39136	0.31501	0.35566
hsa-miR-3679-3p	0	0	0	0.25613	0.06156	0.72769	0.66266	0.30025	0.56718	0.52090	0.84360	0.62873	0.91596
hsa-miR-3660	0	0	0	0.09030	0.75525	0.46772	0.10935	0.61338	0.94722	0.95623	0.98612	0.41555	0.57040
hsa-miR-15a	0	0	0	0.78303	0.53839	0.59428	0.45297	0.60055	0.90950	0.61427	0.24666	0.09604	0.31360
hsa-miR-92b	0	0	0	0.71505	0.59081	0.97650	0.50951	0.95805	0.52960	0.72730	0.91706	0.90660	0.96309
hsa-miR-3648	0	0	0	0.92470	0.39710	0.82530	0.24824	0.65445	0.35594	0.38824	0.71114	0.12268	0.28404
hsa-miR-126	0	0	0	0.20660	0.43237	0.92211	0.14036	0.94718	0.80352	0.53731	0.33949	0.67137	0.94729
hsa-miR-513a-3p	0	0	0	0.97352	0.40842	0.53277	0.05887	0.08798	0.23174	0.87940	0.64714	0.91599	0.35329
hsa-miR-1539	0	0	0	0.12069	0.05401	0.11541	0.44295	0.25283	0.57526	0.81968	0.97981	0.67598	0.97030
hsa-miR-371-3p	0	0	0	0.63827	0.62363	0.57741	0.72238	0.24625	0.18935	0.95079	0.17080	0.08459	0.10996

hsa-let-7b*	0	0	0	0.13239	0.61724	0.30388	0.42673	0.29480	0.88969	0.26662	0.63934	0.96932	0.57288
hsa-miR-362-3p	0	0	0	0.58568	0.55965	0.27905	0.32452	0.55533	0.64689	0.24229	0.25636	0.68962	0.46560
hsa-miR-181d	0	0	0	0.46670	0.29391	0.63302	0.45267	0.09506	0.34927	0.06159	0.15780	0.42076	0.38085
hsa-miR-583	0	0	0	0.80477	0.09222	0.65600	0.25582	0.22965	0.55838	0.35217	0.40269	0.58916	0.40164
hsa-miR-615-3p	0	0	0	0.91870	0.46905	0.52198	0.45093	0.13585	0.38556	0.96848	0.53928	0.41001	0.55979
hsa-miR-605	0	0	0	0.33338	0.91961	0.27412	0.61957	0.68869	0.80034	0.45298	0.41022	0.14697	0.14599
hsa-miR-4284	0	0	0	0.56585	0.08667	0.20802	0.26265	0.71773	0.94729	0.12552	0.31094	0.46527	0.57091
hsa-miR-4310	0	0	0	0.09911	0.06040	0.32031	0.40755	0.28157	0.55893	0.64774	0.99918	0.42188	0.94318
hsa-miR-3621	0	0	0	0.06298	0.51728	0.89927	0.81746	0.45972	0.33459	0.14917	0.70044	0.25749	0.78532
hsa-miR-1285	0	0	0	0.10641	0.23336	0.47934	0.19543	0.06630	0.96148	0.64038	0.48577	0.66470	0.79492
hsa-miR-34a	0	0	0	0.60439	0.96542	0.85885	0.44290	0.35469	0.92408	0.88519	0.25179	0.20433	0.84937
hsa-miR-590-5p	0	0	0	0.44827	0.34156	0.89976	0.52682	0.74451	0.86146	0.91024	0.05273	0.13303	0.15348
hsa-miR-526b	0	0	0	0.55260	0.20441	0.81803	0.42709	0.42163	0.53737	0.71405	0.88719	0.86725	0.73959
hsa-miR-10a	0	0	0	0.96205	0.45439	0.29486	0.11405	0.50433	0.73830	0.79113	0.82167	0.37060	0.99274
hsa-miR-1274a	0	0	0	0.09303	0.41545	0.19086	0.68959	0.62877	0.73522	0.58765	0.17674	0.26875	0.33757
hsa-miR-3177	0	0	0	0.65746	0.21860	0.74154	0.28230	0.45860	0.84285	0.45218	0.79270	0.76064	0.48561
hsa-miR-409-3p	0	0	0	0.80849	0.92452	0.56770	0.37737	0.55588	0.91515	0.18272	0.42676	0.43928	0.44248
hsa-miR-149	0	0	0	0.12547	0.26429	0.10192	0.58134	0.12307	0.65040	0.24616	0.62097	0.94486	0.62502
hsa-miR-25	0	0	0	0.90085	0.79346	0.87038	0.97903	0.84157	0.67244	0.74030	0.62053	0.70289	0.43889
hsa-miR-744	0	0	0	0.05720	0.30644	0.37471	0.41872	0.20874	0.47656	0.95292	0.15033	0.81909	0.70196
hsa-miR-874	0	0	0	0.60448	0.79171	0.33666	0.69388	0.56646	0.97668	0.14355	0.53282	0.88401	0.58851
hsa-miR-340	0	0	0	0.21664	0.15385	0.64724	0.16449	0.25989	0.50877	0.76378	0.07779	0.33439	0.09810
hsa-miR-3148	0	0	0	0.87424	0.87620	0.23079	0.66673	0.07599	0.30829	0.13956	0.41353	0.62730	0.91702
hsa-miR-3647-5p	0	0	0	0.69210	0.18523	0.37924	0.18030	0.27116	0.43863	0.78652	0.59167	0.06852	0.31885
hsa-miR-3659	0	0	0	0.44031	0.49389	0.67383	0.43988	0.99814	0.75913	0.71479	0.84883	0.11164	0.70239
hsa-miR-146a	0	0	0	0.85165	0.81190	0.72864	0.63871	0.79365	0.39628	0.56291	0.45288	0.70461	0.84500
hsa-miR-432	0	0	0	0.35458	0.88029	0.35139	0.90744	0.96956	0.87352	0.97336	0.53693	0.28651	0.57092
hsa-miR-196a	0	0	0	0.79218	0.95410	0.81422	0.19364	0.44599	0.86627	0.42515	0.89263	0.62485	0.65593
hsa-miR-425	0	0	0	0.26193	0.11782	0.75892	0.40125	0.10693	0.91481	0.88721	0.33694	0.96169	0.34285
hsa-miR-410	0	0	0	0.23671	0.29452	0.31037	0.17735	0.12841	0.96442	0.68192	0.39314	0.28178	0.54094
hsa-miR-3911	0	0	0	0.26515	0.86330	0.58107	0.52757	0.55643	0.90646	0.47789	0.84479	0.66972	0.66801
hsa-let-7b	0	0	0	0.15549	0.07817	0.30362	0.05769	0.29846	0.62464	0.20829	0.17593	0.41565	0.71115
hsa-miR-2116*	0	0	0	0.20133	0.08032	0.68903	0.28425	0.33911	0.56397	0.56080	0.96513	0.51307	0.94189
hsa-miR-188-5p	0	0	0	0.25109	0.06467	0.90657	0.17585	0.81170	0.94963	0.85260	0.72615	0.58807	0.51597
hsa-miR-328	0	0	0	0.73325	0.49996	0.40484	0.41141	0.32486	0.10261	0.72806	0.45903	0.39234	0.47000
hsa-miR-361-5p	0	0	0	0.09148	0.18927	0.63520	0.26315	0.07781	0.42527	0.95870	0.44531	0.68699	0.46704
hsa-miR-487b	0	0	0	0.10490	0.24380	0.38487	0.35752	0.23031	0.33893	0.89686	0.66302	0.87311	0.97974
hsa-miR-29a	0	0	0	0.62130	0.51768	0.46312	0.63344	0.32115	0.20476	0.99696	0.75361	0.39370	0.26063
hsa-miR-130b	0	0	0	0.15993	0.20422	0.17167	0.42779	0.18339	0.74928	0.44316	0.24707	0.42681	0.17409
hsa-miR-3663-5p	0	0	0	0.07033	0.77067	0.65091	0.94486	0.43609	0.34176	0.36558	0.61541	0.61660	0.86798
hsa-miR-1229	0	0	0	0.21528	0.14275	0.27586	0.82819	0.10249	0.60095	0.22277	0.44466	0.12252	0.64934
hsa-miR-16-2*	0	0	0	0.84517	0.14629	0.52975	0.48019	0.98073	0.45432	0.45294	0.52772	0.98786	0.94800
hsa-miR-22	0	0	0	0.63604	0.53758	0.30288	0.39748	0.29892	0.60446	0.54349	0.14097	0.09881	0.08200
hsa-miR-3938	0	0	0	0.97897	0.45608	0.52130	0.31007	0.98275	0.84349	0.18642	0.33311	0.24581	0.19168
hsa-miR-29c*	0	0	0	0.60168	0.65529	0.47173	0.42994	0.96440	0.36816	0.70170	0.99213	0.86356	0.77891
hsa-miR-26a	0	0	0	0.32677	0.34044	0.35122	0.05599	0.21655	0.57653	0.90706	0.31912	0.44535	0.49386
hsa-miR-148b	0	0	0	0.31887	0.33564	0.88499	0.16104	0.60152	0.99620	0.83536	0.15347	0.58446	0.39011
hsa-miR-3137	0	0	0	0.86441	0.17889	0.86943	0.08332	0.89942	0.76866	0.78590	0.76149	0.52761	0.65311
hsa-miR-625*	0	0	0	0.58039	0.08954	0.14390	0.42760	0.64519	0.45326	0.22906	0.48933	0.31523	0.64312
hsa-miR-652	0	0	0	0.08039	0.09310	0.59388	0.07994	0.44786	0.78594	0.85789	0.41205	0.80554	0.80729
hsa-miR-937	0	0	0	0.47937	0.60061	0.08706	0.22896	0.19131	0.17154	0.99974	0.37401	0.79452	0.84181
hsa-miR-326	0	0	0	0.51276	0.67389	0.86452	0.75232	0.72508	0.38585	0.18901	0.15721	0.64379	0.44785
hsa-miR-107	0	0	0	0.86948	0.50404	0.41207	0.38872	0.70764	0.67634	0.84178	0.27359	0.75203	0.63550
hsa-miR-1825	0	0	0	0.16987	0.29847	0.38168	0.59748	0.29815	0.60198	0.47400	0.65276	0.98855	0.78127
hsa-miR-877*	0	0	0	0.59128	0.22922	0.53946	0.31400	0.72479	0.74456	0.57495	0.67528	0.17196	0.62171
hsa-miR-423-3p	0	0	0	0.75678	0.73062	0.62561	0.97802	0.57417	0.84044	0.50601	0.15259	0.67472	0.22564

hsa-miR-4265	0	0	0	0.08892	0.08339	0.94670	0.28724	0.77643	0.75587	0.24114	0.43571	0.61403	0.91308
hsa-miR-154	0	0	0	0.19524	0.36683	0.06167	0.13212	0.80173	0.60100	0.51017	0.57962	0.94290	0.66739
hsa-miR-3620	0	0	0	0.13289	0.30864	0.78274	0.45386	0.20351	0.23044	0.60997	0.56255	0.57626	0.66038
hsa-miR-377	0	0	0	0.51892	0.60447	0.45704	0.22905	0.37359	0.79913	0.44583	0.34191	0.27810	0.37260
hsa-miR-4323	0	0	0	0.24857	0.56093	0.55118	0.23159	0.87006	0.56771	0.95738	0.85901	0.68761	0.82592
hsa-miR-373*	0	0	0	0.94961	0.22081	0.10999	0.85230	0.46240	0.38004	0.08126	0.23554	0.96663	0.25594
hsa-miR-17	0	0	0	0.14894	0.36800	0.68893	0.08966	0.47452	0.70213	0.92635	0.61386	0.73894	0.88951
hsa-miR-27a	0	0	0	0.96315	0.93699	0.30769	0.76653	0.95084	0.96801	0.37944	0.98856	0.57937	0.96033
hsa-miR-204	0	0	0	0.95243	0.77640	0.98982	0.76564	0.36128	0.39708	0.34960	0.79010	0.84387	0.66320
hsa-miR-1207-5p	0	0	0	0.38458	0.23276	0.50597	0.07172	0.47766	0.79274	0.96787	0.39905	0.07630	0.27432
hsa-miR-337-5p	0	0	0	0.27314	0.24452	0.22137	0.38891	0.19419	0.17997	0.16663	0.86198	0.96736	0.89421
hsa-miR-130a	0	0	0	0.70948	0.84749	0.67327	0.62664	0.96453	0.20459	0.42786	0.65908	0.49654	0.84960
hsa-miR-19a	0	0	0	0.29942	0.24632	0.49692	0.17453	0.40868	0.61846	0.67500	0.65785	0.12563	0.25931
hsa-miR-451	0	0	0	0.09503	0.30142	0.62604	0.43195	0.46010	0.40124	0.46135	0.87665	0.31017	0.68295
hsa-miR-1973	0	0	0	0.45579	0.81745	0.51620	0.65800	0.33878	0.67681	0.92409	0.18842	0.63402	0.40150
hsa-miR-3156	0	0	0	0.86489	0.27514	0.24307	0.50478	0.67327	0.93543	0.27014	0.85029	0.80870	0.84542
hsa-miR-3181	0	0	0	0.75815	0.99300	0.84164	0.26400	0.90662	0.65554	0.21187	0.23872	0.30812	0.66161
hsa-miR-495	0	0	0	0.33101	0.82763	0.77328	0.28812	0.95582	0.49683	0.21106	0.70483	0.26928	0.69163
hsa-miR-3605-5p	0	0	0	0.91802	0.83939	0.84062	0.08177	0.56832	0.30966	0.37361	0.46854	0.61782	0.20289
hsa-miR-32	0	0	0	0.44872	0.20973	0.99066	0.19941	0.66210	0.49976	0.63667	0.13447	0.49215	0.57419
hsa-miR-324-3p	0	0	0	0.54302	0.41181	0.50333	0.50329	0.68848	0.41498	0.16253	0.09515	0.75989	0.84874
hsa-miR-3197	0	0	0	0.60118	0.57623	0.16702	0.42023	0.93880	0.29486	0.55494	0.53212	0.70605	0.48794
hsa-miR-720	0	0	0	0.77399	0.29493	0.59012	0.26293	0.87145	0.63107	0.93811	0.37304	0.19551	0.83499
hsa-miR-3132	0	0	0	0.29095	0.41288	0.85594	0.22564	0.62896	0.55232	0.41574	0.69855	0.75607	0.93939
hsa-miR-3173	0	0	0	0.15881	0.31731	0.05792	0.19158	0.91023	0.72672	0.71698	0.08792	0.35925	0.70860
hsa-miR-4261	0	0	0	0.22313	0.74044	0.47387	0.29687	0.68158	0.86458	0.42343	0.17844	0.29447	0.66848
hsa-miR-1224-5p	0	0	0	0.91808	0.65990	0.59371	0.40509	0.65462	0.85449	0.79308	0.89201	0.27681	0.59848
hsa-miR-335*	0	0	0	0.49733	0.96411	0.47705	0.24675	0.68455	0.83506	0.76657	0.78263	0.27684	0.66666
hsa-miR-3934	0	0	0	0.41418	0.84087	0.24540	0.59005	0.24259	0.49561	0.25403	0.59986	0.57204	0.74486
hsa-miR-376b	0	0	0	0.09285	0.31129	0.33941	0.71285	0.14314	0.16223	0.58760	0.41218	0.39341	0.60799
hsa-miR-221	0	0	0	0.12014	0.61841	0.66868	0.28566	0.61110	0.90676	0.88842	0.23838	0.50726	0.69628
hsa-miR-4317	0	0	0	0.08004	0.10960	0.71655	0.93069	0.11128	0.50795	0.93518	0.13809	0.98529	0.58770
hsa-miR-3194	0	0	0	0.42350	0.69098	0.35481	0.69783	0.87336	0.95474	0.31494	0.71872	0.61124	0.76786
hsa-miR-485-3p	0	0	0	0.70318	0.74500	0.83562	0.36439	0.13798	0.41432	0.41408	0.44094	0.63812	0.95157
hsa-miR-1273e	0	0	0	0.72852	0.18369	0.78018	0.11577	0.99298	0.77428	0.89514	0.43378	0.58963	0.88528
hsa-miR-34b	0	0	0	0.68703	0.47393	0.54237	0.26071	0.42853	0.70847	0.86893	0.23073	0.99104	0.22117
hsa-miR-449b*	0	0	0	0.83878	0.50638	0.90565	0.22841	0.45892	0.71529	0.93442	0.33364	0.55572	0.38229
hsa-miR-501-3p	0	0	0	0.72917	0.07329	0.98042	0.05381	0.86988	0.52311	0.98383	0.53814	0.31154	0.52061
hsa-miR-4286	0	0	0	0.81560	0.43661	0.28852	0.58401	0.92520	0.96219	0.91585	0.70635	0.22170	0.43986
hsa-miR-3187	0	0	0	0.64828	0.92961	0.79008	0.54944	0.54713	0.73370	0.61964	0.42551	0.57555	0.84553
hsa-miR-1281	0	0	0	0.15416	0.07355	0.45959	0.41972	0.29605	0.44833	0.55669	0.73683	0.90542	0.93733
hsa-miR-664	0	0	0	0.90291	0.70918	0.87216	0.09983	0.70093	0.21222	0.59882	0.87915	0.62522	0.90037
hsa-miR-574-5p	0	0	0	0.34141	0.48146	0.25518	0.51320	0.67230	0.76691	0.87465	0.68345	0.97059	0.97874
hsa-miR-27b	0	0	0	0.23855	0.71062	0.77864	0.28699	0.78997	0.89038	0.95445	0.19085	0.55401	0.82242
hsa-miR-4269	0	0	0	0.40051	0.05313	0.56883	0.73727	0.60522	0.94443	0.12548	0.96470	0.63244	0.94502

O	P	Q	R	S	T	U	V	W	X	Y	Z	AA	AB	AC	AD	AE	AF
ith metastases				without metastases							with metastases						
**TP1 vs TP5 p-value**	**TP1 vs TP6 p-value**	**TP1 vs TP7 p-value**	**TP1 vs TP8 p-value**	**TP2 direction**	**TP3 direction**	**TP4 direction**	**TP5 direction**	**TP6 direction**	**TP7 direction**	**TP8 direction**	**TP2 direction**	**TP3 direction**	**TP4 direction**	**TP5 direction**	**TP6 direction**	**TP7 direction**	**TP8 direction**
0.03149	0.03838	0.01672	0.03785	1	1	1	1	1	1	1	1	1	1	1	1	1	1
0.01233	0.03800	0.01978	0.03686	1	1	1	1	1	1	1	1	1	1	1	1	1	1
0.03838	0.01297	0.00327	0.06985	1	1	1	1	1	1	1	1	1	1	1	1	1	1
0.27306	0.02446	0.01893	0.14759	1	1	1	1	1	1	1	1	1	1	1	1	1	1
0.15142	0.00908	0.00829	0.01311	1	1	1	1	1	1	1	1	1	1	1	1	1	1
0.04597	0.77366	0.03217	0.05695	1	1	1	1	1	1	1	2	2	2	2	2	2	2
0.03716	0.03257	0.12633	0.11764	2	2	1	1	2	1	2	1	1	1	1	1	1	1
0.14591	0.13771	0.01944	0.17155	1	1	1	1	1	1	1	1	1	1	1	1	1	1
0.00729	0.22154	0.21407	0.19948	1	1	1	1	1	1	1	1	1	1	1	1	1	1
0.22317	0.06341	0.00164	0.01076	1	1	1	1	1	1	1	1	1	1	1	1	1	1
0.03271	0.08033	0.09555	0.32073	1	1	1	1	1	1	1	1	1	1	1	1	1	1
0.16668	0.14359	0.04065	0.00839	1	1	1	1	1	1	1	1	1	1	1	1	1	1
0.26913	0.12838	0.04744	0.05180	1	1	1	1	1	1	1	1	1	1	1	1	1	1
0.09192	0.02658	0.03132	0.13261	2	1	1	1	1	1	1	1	1	1	1	1	1	1
0.31551	0.03549	0.28046	0.27709	1	1	1	1	1	1	1	1	1	1	1	1	1	1
0.12510	0.00854	0.33618	0.68322	1	1	1	1	1	1	1	1	1	1	1	1	1	1
0.84413	0.04496	0.31478	0.55722	1	1	1	1	1	1	1	1	1	1	2	1	1	1
0.02339	0.20176	0.60851	0.06471	2	2	2	2	2	2	2	2	2	2	2	2	2	2
0.10766	0.37350	0.04957	0.19982	1	1	1	1	1	1	1	1	1	1	1	1	1	1
0.04991	0.03263	0.28911	0.53814	2	2	2	1	2	1	2	1	1	1	1	1	1	1
0.78229	0.18933	0.02690	0.78090	1	1	1	2	1	1	1	1	1	1	2	1	1	2
0.00381	0.05450	0.10046	0.00418	2	2	2	2	2	2	2	2	2	2	2	2	2	2
0.03176	0.09773	0.03953	0.33137	1	1	1	1	1	1	1	1	1	1	1	1	1	1
0.25057	0.05325	0.00821	0.06071	1	1	1	1	1	1	1	1	1	1	1	1	1	1
0.01893	0.03098	0.06439	0.12200	1	2	2	2	1	2	2	2	2	2	2	2	2	2
0.03021	0.10530	0.22966	0.28798	2	1	1	1	1	2	1	1	1	1	1	1	1	1
0.96450	0.03365	0.11834	0.42476	1	1	2	2	1	1	2	1	1	1	1	1	1	1
0.32286	0.04717	0.95892	0.46535	1	1	1	1	1	1	1	1	1	1	1	1	1	1
0.57002	0.60101	0.75708	0.41546	1	1	1	1	1	1	1	1	1	1	2	1	2	2
0.92970	0.21703	0.31141	0.61865	1	1	1	1	1	1	1	1	1	1	1	1	1	1
0.07518	0.48126	0.90253	0.26209	2	2	2	2	2	2	2	2	1	2	1	1	2	1
0.07102	0.94817	0.03778	0.20647	2	2	2	2	2	2	2	2	1	1	1	1	1	1
0.18131	0.11332	0.18451	0.22681	1	1	1	1	1	1	1	1	1	1	1	1	1	1
0.75296	0.62945	0.88802	0.43097	2	2	2	2	2	1	1	2	2	1	2	2	1	1
0.25940	0.22416	0.24399	0.43215	1	1	1	1	1	1	1	1	1	1	1	1	1	1
0.70186	0.20512	0.97811	0.47057	2	2	2	2	2	2	2	2	2	2	1	2	2	1
0.44402	0.09582	0.36342	0.58017	1	1	1	1	1	1	1	1	1	1	1	1	1	1
0.28751	0.64149	0.47378	0.85865	2	2	2	2	2	2	2	2	1	2	1	2	1	2
0.76600	0.40312	0.85418	0.89270	1	1	1	1	1	1	1	1	1	1	2	1	2	2
0.37211	0.74828	0.38020	0.01531	2	2	1	2	2	1	2	2	1	1	1	1	1	1
0.14235	0.55513	0.14352	0.01993	2	2	1	1	1	1	1	2	1	1	1	1	1	1
0.53434	0.08000	0.10651	0.10854	1	1	1	1	1	1	1	1	1	1	1	1	1	1
0.18288	0.08583	0.18923	0.14654	2	2	2	2	2	2	2	2	2	2	2	2	2	2
0.03857	0.08441	0.33756	0.24657	2	2	2	1	2	2	2	1	1	1	1	1	1	1
0.35791	0.16620	0.21523	0.26403	1	1	1	1	1	1	1	1	1	1	1	1	1	1
0.76814	0.46658	0.90714	0.27646	1	1	1	1	1	1	1	1	1	1	2	1	1	2
0.34827	0.65565	0.04296	0.49595	2	2	1	2	2	2	2	2	1	1	1	2	1	1
0.83365	0.48684	0.69855	0.61586	1	1	1	1	1	1	1	1	1	1	2	1	1	2

0.02421	0.07057	0.56261	0.69726	2	2	2	1	2	1	2	1	1	1	1	1	1	1
0.59093	0.88271	0.47547	0.76425	2	2	2	2	2	2	2	2	1	1	1	1	1	1
0.24850	0.87725	0.30724	0.00937	2	2	1	2	2	1	1	1	1	1	1	1	1	1
0.09572	0.27414	0.39326	0.03223	2	2	2	2	2	2	2	2	2	2	2	2	2	2
0.18179	0.22003	0.52090	0.03934	1	1	1	2	2	2	1	2	2	1	1	1	1	1
0.04695	0.10391	0.27597	0.05248	2	2	2	2	2	2	2	2	2	2	2	2	2	2
0.17128	0.08262	0.01726	0.09795	2	1	1	2	1	1	1	1	1	1	1	1	1	1
0.03933	0.12896	0.46409	0.17492	1	1	1	2	1	2	1	2	2	2	2	2	2	2
0.04374	0.05261	0.26492	0.25864	1	1	1	1	1	2	1	1	1	1	1	1	1	1
0.82312	0.87775	0.18089	0.26434	2	2	2	2	2	2	2	2	1	1	2	2	1	1
0.49024	0.12780	0.17610	0.32693	1	1	1	1	1	1	1	1	1	1	1	1	1	1
0.05139	0.14211	0.36676	0.38583	2	2	2	1	2	1	2	1	1	1	1	1	1	1
0.50571	0.26237	0.24973	0.43423	1	1	1	1	1	1	1	1	1	1	1	1	1	1
0.29781	0.16414	0.49213	0.53745	1	1	1	2	2	1	1	1	1	1	1	1	1	1
0.81385	0.30060	0.60651	0.66473	1	2	1	1	1	2	2	1	2	1	1	1	2	1
0.34880	0.49713	0.56677	0.67536	1	1	1	1	1	1	1	1	1	1	2	1	1	2
0.35634	0.06511	0.48056	0.90322	1	1	1	1	1	1	1	1	1	1	1	1	1	1
0.87479	0.23224	0.14086	0.93552	1	1	1	1	1	1	1	1	1	1	1	1	1	2
0.46513	0.87918	0.42513	0.01077	2	2	1	2	2	2	1	2	2	2	1	1	1	1
0.24800	0.36158	0.15201	0.01476	2	2	2	2	2	2	2	2	1	1	1	1	1	1
0.23174	0.06944	0.80189	0.03946	2	1	2	1	2	2	2	1	2	2	2	2	1	2
0.47911	0.60328	0.26203	0.04351	2	2	1	2	2	1	1	2	1	2	1	1	1	1
0.16750	0.15313	0.05026	0.07805	2	2	2	2	2	2	2	1	1	1	1	1	1	1
0.02498	0.29291	0.22607	0.12763	2	2	2	2	1	2	2	2	2	2	2	2	2	2
0.43759	0.08840	0.25025	0.12897	1	1	1	1	1	1	1	1	1	1	1	1	1	1
0.02699	0.25218	0.86384	0.14636	1	1	2	2	1	1	1	2	2	2	2	2	2	2
0.16000	0.08888	0.00757	0.15281	1	1	1	2	2	1	2	1	1	2	2	2	2	2
0.11153	0.10986	0.30806	0.15576	2	1	1	1	1	2	1	1	1	1	1	1	1	1
0.01911	0.08967	0.21291	0.20544	2	1	2	1	2	2	2	1	1	1	1	1	1	1
0.27091	0.22207	0.33097	0.31049	1	1	1	1	1	2	1	1	1	1	1	1	1	1
0.10874	0.35685	0.89113	0.32313	2	2	2	1	2	2	2	2	1	1	1	1	2	1
0.43285	0.17724	0.19080	0.34070	2	1	2	2	2	1	2	1	1	1	1	1	1	1
0.22850	0.09185	0.38739	0.42426	2	2	2	2	2	2	2	2	1	1	1	1	1	1
0.61048	0.45259	0.56583	0.46830	2	2	2	2	2	2	2	2	2	2	1	1	2	1
0.16363	0.18162	0.06375	0.50647	1	1	1	1	1	1	1	1	1	1	1	1	1	1
0.28159	0.34881	0.66242	0.57883	2	1	2	2	2	2	1	1	1	1	1	1	1	1
0.73270	0.11364	0.25928	0.67507	1	1	1	1	1	1	1	1	1	1	1	1	1	1
0.44272	0.13359	0.52949	0.70245	1	1	1	1	1	1	1	1	1	1	1	1	1	2
0.39902	0.41702	0.96516	0.79010	1	1	2	1	2	2	2	1	1	1	1	1	1	2
0.51923	0.42336	0.73962	0.30088	2	2	2	2	2	2	2	2	1	2	2	1	1	2
0.59246	0.06771	0.11269	0.20458	1	1	1	1	1	1	1	1	1	1	1	1	1	1
0.43706	0.62427	0.49930	0.47848	1	1	1	1	1	1	1	1	1	1	1	2	1	1
0.99547	0.43862	0.34889	0.55748	1	1	1	1	1	1	1	1	1	1	1	1	1	2
0.33482	0.72307	0.09919	0.14676	1	1	1	1	1	1	1	1	1	1	1	1	1	1
0.08874	0.14746	0.42496	0.31120	2	2	2	1	2	2	2	1	1	1	1	1	1	1
0.71193	0.44526	0.93305	0.43856	1	1	1	1	1	1	1	1	2	1	2	1	1	2
0.07564	0.42587	0.47586	0.55544	1	1	1	1	1	1	1	1	2	2	2	2	2	2
0.38242	0.36020	0.53077	0.58161	1	1	1	1	1	1	1	1	2	1	2	2	2	2
0.62465	0.59448	0.72281	0.81861	1	1	1	1	1	1	1	1	2	1	2	2	1	2
0.36373	0.56570	0.68210	0.99104	2	2	2	2	2	2	2	2	2	2	1	2	2	2
0.77790	0.21949	0.23249	0.10942	2	2	2	2	2	2	2	2	2	2	2	2	2	2
0.25377	0.07849	0.07433	0.19159	1	1	1	1	1	1	1	1	1	1	1	1	1	1
0.34835	0.18450	0.13776	0.19202	1	1	1	1	1	1	1	1	1	1	1	1	1	1
0.06805	0.34802	0.62501	0.19821	2	2	2	2	2	2	2	2	2	2	1	1	2	1
0.96298	0.19495	0.50715	0.22115	2	2	2	2	2	2	2	2	2	2	1	2	2	2
0.31387	0.97419	0.28519	0.38698	2	2	2	2	2	2	2	2	1	1	1	2	1	1

0.97497	0.37553	0.33760	0.41903	1	1	1	1	1	1	1	1	1	1	1	1	1	1
0.20786	0.13390	0.76023	0.47889	2	2	2	2	2	2	2	1	1	1	1	1	1	1
0.81153	0.72881	0.89723	0.49912	1	1	1	1	1	1	1	1	2	1	2	1	2	2
0.15129	0.28595	0.92316	0.52461	2	2	2	2	2	2	2	2	2	2	2	2	2	2
0.43993	0.78386	0.49559	0.53954	2	2	2	2	2	2	2	2	1	1	1	1	1	1
0.96068	0.13616	0.89063	0.55877	2	2	2	2	2	2	2	2	2	2	2	2	1	2
0.65063	0.34030	0.68386	0.58602	2	2	2	2	2	2	2	2	2	2	2	2	2	2
0.53943	0.37052	0.51796	0.63234	1	1	1	1	1	1	1	2	2	2	2	2	1	2
0.55896	0.36251	0.57908	0.83456	2	2	2	2	2	2	2	1	2	1	1	2	2	2
0.48016	0.98718	0.73570	0.85388	2	2	2	2	2	2	2	2	2	2	1	1	2	1
0.50613	0.76894	0.49022	0.87617	1	1	1	2	1	1	1	1	2	1	2	1	1	1
0.72237	0.26143	0.20362	0.89729	1	1	1	1	1	1	1	1	1	1	1	1	1	1
0.80343	0.93945	0.75502	0.91997	1	1	1	1	1	1	1	1	2	1	2	1	2	2
0.63917	0.56980	0.60314	0.92702	2	2	2	2	2	2	2	2	1	1	1	2	1	1
0.25641	0.99821	0.67226	0.98475	1	1	1	1	1	1	1	1	2	1	2	1	1	1
0.97318	0.68781	0.35081	0.99060	1	1	1	1	1	2	2	1	1	1	2	1	1	2
0.14724	0.85458	0.55234	0.16459	1	1	1	1	1	1	2	1	1	2	2	2	2	2
0.22172	0.11353	0.77241	0.17476	2	2	2	2	2	2	2	1	2	2	2	2	1	2
0.21149	0.23119	0.26138	0.18977	1	1	1	1	1	1	1	1	1	1	1	1	1	1
0.65212	0.24843	0.38157	0.19365	1	1	1	1	1	1	1	1	1	1	2	1	1	2
0.28027	0.53654	0.36965	0.25703	1	1	1	1	1	1	1	1	2	1	2	2	1	2
0.23396	0.10517	0.99875	0.30682	2	2	2	2	2	2	2	2	2	2	1	1	1	1
0.08810	0.36735	0.25140	0.32555	2	2	2	2	2	2	2	2	1	1	1	1	1	1
0.50016	0.89444	0.63711	0.32833	1	1	1	1	1	1	1	1	1	1	2	2	2	2
0.89557	0.87647	0.99832	0.34793	1	1	1	1	1	1	1	1	2	1	2	1	1	2
0.41976	0.81242	0.47832	0.36876	1	1	1	1	1	1	1	1	2	2	2	2	2	2
0.94325	0.65322	0.45004	0.37896	2	2	2	2	2	2	1	2	1	2	2	2	2	2
0.80000	0.86558	0.78547	0.41499	1	1	1	1	1	1	1	1	2	1	2	1	2	2
0.59327	0.98362	0.60808	0.42721	1	1	1	1	1	1	1	1	2	1	2	1	2	2
0.46194	0.06589	0.53666	0.45222	2	2	2	2	2	2	2	2	2	2	2	2	2	2
0.20361	0.49706	0.68367	0.46458	2	2	1	2	2	2	2	2	1	2	1	1	1	1
0.82809	0.06755	0.11096	0.46775	1	1	1	1	1	1	1	1	1	1	1	1	1	1
0.16245	0.86534	0.63458	0.47040	2	2	1	2	2	1	1	2	1	1	1	1	1	1
0.54769	0.12851	0.72659	0.50526	2	2	2	2	2	2	2	2	2	2	2	2	2	2
0.78352	0.26686	0.15178	0.50616	1	1	1	1	1	1	1	1	1	1	1	1	1	1
0.12061	0.17951	0.23143	0.56622	1	2	1	1	1	1	1	1	1	1	1	1	1	1
0.14820	0.53396	0.84121	0.59420	2	2	2	1	2	1	2	2	1	2	1	1	2	1
0.47985	0.80923	0.96319	0.63918	1	1	1	1	1	1	1	1	1	1	2	1	2	2
0.83606	0.73021	0.57570	0.66891	1	1	1	1	1	1	1	1	1	1	2	1	1	1
0.93818	0.58504	0.99351	0.70870	1	1	1	1	1	1	1	1	1	1	2	1	2	2
0.76296	0.79965	0.99079	0.71178	1	1	1	1	1	1	1	1	1	1	2	2	2	2
0.99968	0.16740	0.64614	0.71264	2	1	2	2	2	2	1	2	2	2	1	2	2	2
0.25419	0.41860	0.56475	0.73580	2	2	2	1	2	2	2	2	1	1	1	1	2	1
0.33479	0.96820	0.67515	0.74514	1	1	1	1	1	1	1	1	1	1	2	1	1	2
0.73397	0.81664	0.69717	0.78986	1	1	1	1	1	1	1	1	1	1	2	2	2	2
0.99516	0.41130	0.74735	0.81174	1	1	1	1	1	1	1	1	1	1	2	1	1	2
0.71261	0.77599	0.80729	0.86390	1	1	1	2	1	2	1	1	2	1	2	2	2	2
0.31031	0.73509	0.45169	0.86440	2	2	2	1	2	2	2	2	1	1	1	1	2	2
0.34173	0.16419	0.97109	0.92585	1	1	1	2	1	1	1	1	1	1	1	1	2	1
0.79683	0.94851	0.46208	0.95174	1	1	1	1	1	1	1	2	2	2	1	2	1	1
0.65374	0.77054	0.29432	0.95947	1	1	1	1	1	1	1	1	2	1	2	1	1	1
0.99475	0.59125	0.81325	0.97892	1	1	1	1	1	1	1	1	1	1	2	1	1	2
0.12408	0.66342	0.68688	0.05903	2	2	1	2	2	2	1	1	2	2	1	1	1	1
0.38907	0.78738	0.27978	0.07736	1	1	2	1	1	1	2	1	2	2	2	1	2	2
0.23631	0.07894	0.95926	0.12442	2	2	2	2	2	2	2	2	2	2	2	2	2	2
0.31390	0.93908	0.98917	0.14014	1	1	2	1	1	1	1	1	2	1	2	2	1	2

0.08710	0.15899	0.96188	0.15701	2	2	2	2	2	2	2	2	1	2	2	2	1	2
0.35008	0.56404	0.43914	0.15974	2	2	1	2	2	1	1	2	2	1	1	1	1	1
0.55107	0.18790	0.31387	0.16282	2	1	2	2	2	2	2	2	2	2	2	2	2	2
0.91596	0.23458	0.32765	0.16522	2	1	2	1	2	2	2	2	1	2	2	2	2	2
0.22716	0.05367	0.28397	0.16533	2	2	2	2	2	2	2	2	2	2	2	2	2	2
0.35707	0.33832	0.58635	0.19533	1	1	1	1	1	1	1	1	1	1	1	1	1	1
0.73683	0.85756	0.39220	0.23740	2	2	2	2	2	2	2	2	1	2	2	1	2	2
0.13665	0.17855	0.23437	0.25475	2	1	1	1	1	1	1	1	1	1	1	1	1	1
0.27204	0.10794	0.35467	0.26216	1	2	2	2	2	2	2	2	2	2	2	2	2	2
0.21808	0.92969	0.99009	0.26725	1	1	1	2	1	1	1	1	2	2	2	1	2	2
0.89161	0.24164	0.36665	0.27716	1	2	2	2	2	2	2	2	2	2	2	2	2	2
0.40015	0.89359	0.41919	0.28560	1	1	1	2	1	1	1	1	2	2	2	2	2	2
0.40191	0.68240	0.37288	0.29191	1	1	1	1	1	1	1	1	2	1	1	1	1	1
0.35065	0.81152	0.51948	0.29689	1	2	2	2	2	2	1	1	1	1	2	2	1	2
0.58307	0.13282	0.56335	0.36164	1	1	1	1	1	1	1	1	1	1	1	1	1	1
0.33665	0.24580	0.16670	0.36217	1	1	1	1	1	1	1	1	1	1	1	1	1	1
0.71957	0.88729	0.88977	0.39230	1	1	1	1	1	1	1	1	1	1	1	1	2	2
0.48084	0.66779	0.30418	0.39269	2	2	2	1	2	2	2	2	1	2	1	2	2	2
0.19756	0.65775	0.24997	0.39315	2	2	1	2	2	1	1	1	1	1	1	1	1	1
0.46400	0.80799	0.74011	0.41352	1	1	1	2	1	1	1	1	1	1	2	1	1	1
0.53131	0.93828	0.88664	0.42743	2	2	2	2	2	1	1	2	2	2	1	1	2	1
0.15002	0.24134	0.80952	0.43343	2	2	2	1	2	1	2	1	1	1	1	1	1	1
0.06363	0.09849	0.24727	0.44424	2	2	1	1	2	1	2	1	1	1	1	1	1	1
0.67879	0.79542	0.07888	0.47312	1	1	1	2	1	1	1	2	1	1	1	1	1	1
0.25093	0.41834	0.09613	0.47616	2	2	2	2	1	2	1	2	1	1	1	2	1	1
0.83622	0.26044	0.23084	0.48532	1	1	1	1	1	1	1	1	1	1	1	1	1	1
0.28515	0.56571	0.10614	0.49567	2	2	2	2	2	2	2	2	1	1	1	2	1	1
0.17871	0.21561	0.91345	0.51253	2	2	2	1	2	1	2	1	1	1	1	1	1	1
0.44947	0.68364	0.60113	0.51846	1	1	1	2	1	1	1	1	2	1	1	1	1	1
0.65078	0.38969	0.21777	0.52980	1	1	1	1	1	1	1	1	1	1	1	1	1	1
0.81152	0.45370	0.84715	0.54205	1	1	1	2	1	1	1	2	2	2	2	2	1	2
0.07509	0.19062	0.80437	0.55084	2	2	2	1	2	1	2	1	1	1	1	1	1	1
0.23947	0.14843	0.34709	0.56044	1	2	2	2	1	2	2	2	2	2	2	2	2	2
0.28462	0.41218	0.42316	0.57496	1	1	1	2	1	1	1	1	2	2	2	2	2	2
0.73649	0.56865	0.77001	0.57748	2	2	2	2	2	1	2	2	1	1	1	2	1	1
0.25767	0.33518	0.77633	0.57775	1	1	1	2	1	2	1	1	2	2	2	2	2	2
0.81058	0.72328	0.52719	0.58179	2	2	2	2	2	2	2	2	2	1	2	1	1	1
0.14104	0.35472	0.83655	0.61131	2	2	2	1	2	2	2	1	1	1	1	1	2	1
0.31285	0.60796	0.99580	0.63359	1	1	1	2	1	1	1	2	2	2	2	2	2	2
0.94463	0.42915	0.09062	0.63890	1	1	1	1	1	1	1	1	1	1	2	1	1	1
0.70124	0.47811	0.37959	0.64559	1	1	1	1	1	1	1	1	2	2	1	2	2	1
0.81980	0.97918	0.37025	0.65321	1	1	1	2	1	1	1	2	2	1	1	2	1	1
0.07120	0.10363	0.40557	0.65487	1	1	1	1	1	1	1	1	1	1	1	1	1	2
0.58708	0.65215	0.63741	0.65693	1	1	1	1	1	1	1	1	1	1	2	1	1	2
0.41257	0.11410	0.56283	0.66327	2	2	2	2	2	2	2	2	2	2	2	2	2	2
0.87226	0.52003	0.92046	0.66602	1	1	1	1	1	1	1	1	1	1	1	1	1	1
0.67142	0.31425	0.72818	0.67564	1	1	1	1	1	2	2	2	2	2	2	2	2	2
0.17612	0.94798	0.33717	0.67867	2	2	2	2	2	2	2	2	1	1	2	1	1	1
0.45220	0.85665	0.65552	0.69386	1	1	1	2	1	1	1	2	2	2	2	1	1	1
0.73893	0.26284	0.90392	0.69959	1	1	1	1	1	1	1	1	2	1	1	1	1	2
0.25276	0.78435	0.42265	0.70018	2	2	2	2	2	2	1	2	1	1	1	2	1	1
0.78448	0.52226	0.48672	0.70610	1	1	1	1	1	1	1	1	1	1	1	1	1	1
0.22277	0.45292	0.77525	0.70803	2	2	2	1	2	1	2	2	1	1	1	1	2	1
0.43187	0.67439	0.17943	0.70899	1	1	1	1	1	2	1	1	1	1	1	1	2	2
0.21421	0.51092	0.74200	0.71261	2	2	2	1	2	1	2	1	1	1	1	1	2	1
0.17499	0.27945	0.94725	0.72626	2	2	2	1	2	1	1	1	1	1	1	1	2	1

0.64716	0.24642	0.57775	0.75313	1	1	1	1	1	1	2	1	1	1	1	1	1	1
0.93589	0.47041	0.66967	0.76956	1	1	1	1	1	1	2	1	1	1	2	1	1	2
0.70505	0.24731	0.53907	0.77002	2	2	2	2	2	2	2	2	2	1	1	2	1	2
0.80867	0.57190	0.60119	0.78223	1	2	2	2	1	2	2	2	1	2	1	1	2	2
0.75311	0.42841	0.98332	0.78428	1	1	2	2	1	1	1	2	2	2	2	2	2	2
0.80432	0.82495	0.39108	0.80814	1	1	1	1	1	1	1	1	1	1	1	1	1	1
0.80741	0.44774	0.91772	0.80955	1	1	1	1	1	1	1	1	1	1	2	1	1	2
0.91342	0.11345	0.81717	0.81206	2	2	2	2	2	2	2	1	1	1	1	1	1	1
0.81195	0.43734	0.78104	0.81309	1	2	2	2	2	2	2	2	2	1	2	2	2	1
0.36622	0.78161	0.55337	0.83355	2	2	2	1	2	2	2	2	1	2	1	1	2	1
0.64166	0.61020	0.90687	0.83443	1	1	1	1	1	1	1	1	1	1	2	1	2	2
0.30597	0.69092	0.15126	0.83724	2	2	2	2	2	2	2	2	2	2	1	2	2	2
0.48474	0.42218	0.41156	0.84592	2	2	2	2	2	2	2	2	1	2	2	2	2	1
0.69228	0.60446	0.86677	0.88042	1	1	1	1	1	1	1	1	1	1	2	1	2	2
0.74708	0.91834	0.56794	0.89513	1	1	1	1	1	1	1	1	2	1	1	2	1	1
0.84201	0.44338	0.99040	0.91399	2	2	2	2	2	2	2	1	2	1	2	2	1	2
0.31722	0.90332	0.61150	0.91963	2	2	2	1	2	1	2	1	1	2	1	1	2	2
0.59294	0.45548	0.49213	0.92037	2	2	2	2	2	2	1	2	1	1	2	1	1	1
0.13404	0.20583	0.36167	0.92841	2	2	2	2	2	2	2	1	1	1	1	1	1	1
0.77318	0.83990	0.74611	0.94917	1	2	2	2	2	2	2	2	2	2	2	1	2	1
0.15474	0.79344	0.19946	0.95180	1	2	2	2	1	2	2	2	2	2	2	2	2	1
0.33969	0.84875	0.73268	0.95669	2	2	2	2	2	1	2	1	1	1	1	1	2	2
0.55475	0.67939	0.99428	0.97224	2	1	1	1	1	1	1	1	1	1	2	1	1	2
0.87537	0.75855	0.85243	0.97819	1	1	1	1	1	1	1	1	1	1	2	1	1	2
0.43014	0.28368	0.62010	0.98027	2	2	2	2	2	2	2	2	2	2	1	2	2	2
0.92301	0.71828	0.81312	0.98116	1	1	1	2	1	2	1	1	2	2	2	2	2	2
0.24349	0.19513	0.65267	0.99761	2	2	2	1	2	1	2	1	1	1	1	1	1	1
0.67444	0.34466	0.56920	0.07064	2	2	2	2	2	2	2	1	2	1	1	2	2	2
0.17250	0.30960	0.43887	0.07215	1	1	2	2	1	2	2	2	2	2	2	2	2	2
0.27778	0.55334	0.47539	0.07416	2	2	1	2	2	2	1	1	1	2	1	1	1	1
0.10173	0.32576	0.66007	0.10691	1	1	1	1	1	1	1	1	2	2	2	2	2	2
0.89700	0.15991	0.56876	0.10985	1	1	1	2	1	1	1	2	2	2	1	2	2	2
0.97162	0.36018	0.05402	0.11396	2	2	1	1	2	2	2	2	2	2	1	2	2	2
0.73279	0.63570	0.47864	0.11520	1	2	1	2	1	2	2	1	2	2	2	2	2	2
0.13635	0.78438	0.66181	0.13146	1	1	1	2	1	1	1	1	2	2	2	1	1	2
0.13569	0.17259	0.09958	0.13516	2	2	1	2	1	1	1	1	1	1	1	1	1	1
0.42348	0.84160	0.73593	0.14101	1	2	1	2	2	1	1	2	2	1	1	2	1	1
0.74445	0.27301	0.48692	0.14968	1	2	2	2	1	2	1	2	2	2	2	2	2	2
0.39397	0.77753	0.96641	0.15389	1	1	2	1	2	2	2	1	2	2	2	2	2	2
0.57380	0.23341	0.69470	0.15680	1	2	1	2	1	2	2	1	2	2	2	2	2	2
0.47546	0.87491	0.54882	0.15850	2	2	2	2	2	2	2	1	2	2	1	2	2	2
0.39337	0.40709	0.52099	0.16091	1	1	1	2	1	2	1	1	2	2	2	2	2	2
0.64610	0.22173	0.45447	0.16168	1	1	1	2	1	1	1	1	2	2	2	2	2	2
0.26061	0.68544	0.20691	0.16311	2	2	2	2	2	2	1	1	1	1	1	1	1	1
0.34370	0.86768	0.48173	0.16632	2	1	1	2	1	2	1	1	1	1	1	1	1	1
0.08361	0.44025	0.98204	0.17181	1	1	2	2	2	1	1	1	1	1	2	2	2	2
0.38182	0.73039	0.16340	0.17899	2	2	2	1	2	1	2	2	1	2	1	2	2	2
0.83824	0.67215	0.69376	0.18957	1	1	1	2	1	1	1	2	2	2	2	2	2	2
0.27627	0.79960	0.82531	0.19222	1	2	1	1	2	1	1	1	2	2	1	1	1	1
0.74204	0.18673	0.62223	0.20483	1	2	1	2	1	1	1	2	2	2	2	2	2	2
0.06736	0.24075	0.30476	0.21438	2	2	2	2	1	1	1	2	2	2	2	2	2	2
0.51752	0.82570	0.91180	0.21466	1	2	1	2	2	1	1	2	2	1	1	2	1	1
0.26592	0.11043	0.27234	0.22244	1	2	2	2	1	2	2	2	2	2	2	2	2	2
0.38638	0.33703	0.81227	0.22783	2	2	2	2	2	1	2	1	1	2	1	1	2	1
0.10950	0.32859	0.44049	0.23104	2	2	2	2	2	2	2	1	2	2	2	2	2	2
0.27658	0.76824	0.86447	0.23249	2	2	2	2	2	2	1	2	2	1	1	1	1	1

0.27644	0.05984	0.53976	0.23707	2	2	2	2	2	2	2	1	1	2	2	2	1	2
0.93832	0.55417	0.15396	0.24240	2	1	1	1	1	1	1	1	1	1	2	1	1	1
0.73245	0.23154	0.45073	0.24716	1	2	1	2	2	1	1	1	2	2	2	2	2	2
0.67624	0.22581	0.32729	0.24725	2	1	2	2	2	2	2	2	2	2	2	2	2	2
0.32761	0.45677	0.71795	0.24927	2	2	2	2	2	2	2	2	2	2	2	2	2	2
0.36029	0.15787	0.56426	0.25692	2	1	2	2	2	2	1	1	1	2	2	2	1	2
0.98153	0.73709	0.68404	0.25879	1	1	1	2	1	1	1	2	2	1	2	2	1	2
0.83453	0.25939	0.36537	0.25912	1	1	2	1	1	1	1	2	2	2	2	2	2	2
0.96600	0.54085	0.77439	0.25998	1	1	1	2	1	1	1	1	2	1	1	2	2	2
0.45576	0.51738	0.86447	0.26416	2	2	1	2	2	1	1	2	2	2	1	1	2	1
0.50882	0.41978	0.38385	0.26649	1	1	1	1	2	2	1	2	2	2	1	1	2	1
0.52892	0.90257	0.93863	0.27157	1	1	2	2	1	2	1	1	1	2	2	2	1	2
0.40780	0.16969	0.26901	0.27341	1	1	2	1	2	1	2	2	2	2	2	2	2	2
0.19099	0.08344	0.35372	0.28313	1	1	1	1	1	1	1	1	1	1	1	1	1	1
0.81529	0.85808	0.32878	0.29257	1	2	1	2	1	2	2	2	2	2	2	2	2	2
0.50815	0.88424	0.79395	0.29653	1	1	2	1	2	2	2	1	2	2	2	2	2	2
0.37481	0.49029	0.94273	0.30564	1	2	1	1	2	1	1	2	2	2	1	1	2	1
0.17118	0.26205	0.43561	0.31448	1	1	1	1	1	1	1	1	1	1	1	1	1	1
0.47348	0.51462	0.66142	0.31449	1	1	2	2	1	2	1	2	2	2	2	2	2	2
0.63670	0.24099	0.93161	0.31581	2	2	1	2	1	2	2	1	2	2	2	2	2	2
0.52470	0.43643	0.83875	0.31751	1	1	1	1	1	1	1	1	2	2	2	2	2	2
0.16640	0.16498	0.31964	0.32489	1	1	1	1	2	2	2	2	2	2	2	2	2	2
0.33273	0.19041	0.49848	0.32787	1	1	1	1	1	1	1	1	1	1	1	1	1	1
0.33823	0.42765	0.72447	0.32925	1	1	1	1	1	1	1	1	2	2	1	1	1	1
0.30628	0.51946	0.33344	0.33451	1	1	1	2	2	1	1	1	1	1	2	2	2	2
0.57466	0.31275	0.56872	0.34072	2	2	2	2	1	2	2	2	2	2	2	2	2	2
0.81653	0.36175	0.73044	0.34219	2	2	2	2	2	1	2	2	2	2	2	2	2	2
0.16784	0.28325	0.62203	0.34408	2	2	2	2	2	2	2	2	2	2	1	1	2	1
0.24140	0.09054	0.08163	0.34499	1	1	1	1	1	1	1	1	1	1	1	1	1	1
0.60095	0.06374	0.18094	0.34892	2	1	1	1	1	1	2	1	1	1	1	1	1	1
0.45896	0.14141	0.84943	0.35372	1	1	1	2	1	2	2	1	2	1	2	2	1	2
0.98510	0.35377	0.70045	0.35548	1	2	1	2	2	2	1	1	2	2	1	2	2	2
0.51702	0.32648	0.75411	0.36384	1	2	1	2	2	2	1	1	2	2	2	2	1	2
0.08866	0.30529	0.29862	0.36448	2	1	1	2	2	2	1	1	1	1	1	1	1	1
0.53578	0.48873	0.18738	0.37187	2	2	2	2	2	2	2	1	2	2	2	2	2	2
0.49395	0.81624	0.48633	0.37523	2	2	1	2	2	2	2	2	2	2	2	2	2	2
0.63323	0.23352	0.73357	0.37801	1	1	1	2	1	2	1	1	2	2	2	2	2	2
0.45109	0.42335	0.76097	0.38062	1	1	2	1	1	1	1	1	1	1	2	1	1	2
0.56762	0.12295	0.26115	0.38835	1	1	1	2	1	2	1	1	2	2	2	2	2	2
0.55854	0.42924	0.40737	0.39349	1	1	1	1	2	2	2	2	2	2	1	2	2	2
0.27938	0.15915	0.77100	0.39554	2	2	2	1	2	1	2	1	1	1	1	1	1	1
0.20683	0.72850	0.42853	0.39843	1	1	1	2	1	2	1	1	2	2	2	2	2	2
0.18833	0.55539	0.11994	0.39941	2	2	1	1	1	1	2	1	1	1	1	1	1	1
0.34878	0.29624	0.45989	0.40488	1	2	2	2	1	1	2	2	2	2	2	2	2	2
0.87867	0.53683	0.29279	0.40757	1	1	1	1	1	1	1	1	2	1	2	1	1	2
0.18615	0.37698	0.16048	0.40921	1	1	1	1	1	2	1	1	1	1	1	1	1	1
0.61630	0.53983	0.87272	0.41211	1	2	1	2	2	1	1	1	1	2	1	1	2	1
0.32059	0.31044	0.77353	0.41398	1	2	2	2	1	2	1	1	2	2	2	2	1	2
0.37311	0.45880	0.88553	0.41783	2	1	1	1	2	2	1	1	1	1	1	2	2	2
0.89728	0.31088	0.24610	0.41786	2	1	2	2	2	2	2	2	2	2	2	2	2	2
0.61992	0.97862	0.19138	0.42632	2	2	2	2	2	2	2	2	1	1	1	1	1	1
0.22006	0.33233	0.30470	0.42693	1	1	1	1	1	1	1	1	1	1	1	1	1	1
0.80566	0.37819	0.93097	0.44026	1	1	1	2	1	2	1	1	2	1	2	2	2	2
0.95569	0.99907	0.64407	0.44154	1	2	2	1	1	2	2	1	1	2	2	2	1	2
0.61283	0.36122	0.58208	0.44834	1	2	2	1	2	1	2	1	1	1	2	1	1	2
0.14839	0.47591	0.28696	0.44873	2	2	1	2	2	2	2	1	1	1	1	1	1	1

0.13974	0.94965	0.97529	0.45287	2	2	2	2	2	2	2	2	1	1	1	1	2	1
0.38341	0.70980	0.74792	0.45738	1	1	2	1	1	1	2	2	2	2	2	2	2	2
0.05069	0.09419	0.24211	0.45771	2	1	1	2	2	2	1	1	2	2	2	2	2	2
0.50893	0.70624	0.34270	0.45817	2	1	1	2	1	1	1	1	1	1	1	1	1	1
0.28607	0.79806	0.49701	0.45946	2	2	1	2	1	1	1	2	1	1	2	2	1	2
0.13194	0.17544	0.95473	0.46470	2	2	1	1	2	1	2	1	1	1	1	1	1	1
0.21393	0.93212	0.47826	0.46623	2	2	1	2	2	2	2	2	1	1	1	1	1	1
0.55341	0.44865	0.45481	0.46914	1	1	1	2	1	2	1	1	2	2	2	2	2	2
0.59613	0.17872	0.49604	0.47303	1	1	1	1	1	1	1	1	1	1	1	1	1	1
0.76511	0.65643	0.65336	0.47368	1	1	1	2	1	1	1	1	2	2	2	2	2	2
0.58064	0.87619	0.69512	0.47405	2	1	1	1	1	1	1	1	1	1	1	2	2	1
0.96335	0.89965	0.96993	0.47644	1	1	1	1	2	1	2	2	1	2	1	2	2	2
0.57780	0.68223	0.48860	0.48136	1	2	1	1	1	1	1	1	1	1	1	2	1	2
0.56343	0.27782	0.56749	0.49064	2	1	1	1	1	1	1	1	1	1	1	1	1	1
0.37754	0.55912	0.37264	0.49471	1	2	1	1	1	1	1	1	2	1	1	1	1	1
0.98603	0.67835	0.48095	0.49711	1	2	1	2	1	2	1	1	2	2	2	2	2	2
0.41792	0.21838	0.33773	0.50982	2	2	2	1	2	2	2	1	2	2	1	2	2	2
0.84386	0.49786	0.24075	0.51199	2	2	2	2	2	2	2	2	2	2	1	2	2	2
0.48656	0.55721	0.47682	0.51885	1	1	2	2	1	1	1	1	1	1	2	1	1	2
0.68449	0.58484	0.77211	0.52341	2	2	2	2	2	2	2	1	1	1	1	1	1	1
0.75239	0.75923	0.32988	0.52390	1	1	2	1	1	2	2	1	1	1	1	2	2	2
0.37482	0.07783	0.48209	0.52487	2	2	2	2	2	2	2	2	2	2	2	2	2	2
0.94091	0.11097	0.69341	0.52577	2	2	2	2	2	2	2	1	1	1	1	2	2	2
0.58490	0.52748	0.50564	0.52615	2	1	1	1	2	2	2	2	1	2	2	2	2	2
0.37296	0.45754	0.65071	0.53090	1	2	1	2	2	2	1	1	2	2	1	1	2	2
0.97646	0.29434	0.65762	0.53511	1	1	2	1	1	1	1	1	1	1	2	1	2	2
0.88841	0.87817	0.35639	0.53994	1	2	1	2	1	1	1	2	2	2	1	1	1	1
0.63762	0.87425	0.99640	0.54049	1	2	1	2	1	1	1	2	2	2	1	2	1	1
0.45626	0.33714	0.70396	0.54062	1	2	2	2	1	1	1	2	2	2	2	2	2	2
0.79067	0.21328	0.28135	0.54910	1	1	1	1	1	2	2	1	1	1	1	1	1	1
0.91536	0.24850	0.92666	0.55474	1	1	1	1	1	1	1	1	1	1	1	1	2	2
0.82136	0.60475	0.90660	0.55789	1	1	1	2	1	1	1	1	2	2	2	2	1	2
0.87311	0.36463	0.62661	0.55954	1	1	1	1	2	1	1	1	1	1	1	1	1	1
0.13798	0.53332	0.63349	0.56111	1	1	2	2	1	2	1	2	2	2	2	2	2	2
0.14135	0.32412	0.25657	0.56656	1	1	1	2	1	1	1	1	1	1	1	1	1	1
0.99701	0.95963	0.56945	0.56796	1	2	1	1	1	1	1	2	1	2	1	2	2	2
0.86028	0.89530	0.75394	0.57197	1	1	1	1	1	1	1	1	1	1	1	1	2	1
0.79952	0.15946	0.20310	0.57672	1	1	1	1	1	1	2	1	1	1	1	1	1	1
0.14330	0.47831	0.76537	0.57673	2	2	2	1	2	1	2	1	1	1	1	1	2	1
0.76214	0.52341	0.33742	0.57709	2	1	1	1	1	1	2	2	1	2	1	2	2	2
0.39829	0.77930	0.74922	0.58950	1	2	1	1	1	1	1	1	2	2	1	2	1	1
0.10347	0.16428	0.87385	0.59181	2	2	2	1	2	1	2	1	1	1	1	1	1	2
0.49955	0.94109	0.88461	0.59722	1	2	1	2	1	1	1	2	2	2	1	1	2	1
0.94475	0.97257	0.41093	0.61595	2	2	2	2	2	2	2	2	2	2	1	1	2	2
0.55237	0.64530	0.98527	0.61740	2	2	1	2	2	2	2	1	2	1	1	2	1	2
0.36298	0.94520	0.91902	0.62163	1	1	1	2	1	1	1	2	2	2	2	1	1	2
0.88454	0.23432	0.19740	0.62384	1	1	1	1	1	1	2	1	1	1	1	1	1	1
0.24308	0.99671	0.93794	0.62444	2	2	2	1	2	2	2	1	1	1	1	1	1	2
0.45192	0.93116	0.47092	0.62816	1	1	2	2	1	1	1	2	2	2	2	1	2	2
0.93080	0.24394	0.70049	0.63111	2	1	1	1	1	1	1	1	1	1	1	1	1	1
0.68520	0.82656	0.62950	0.63277	2	2	2	1	1	1	2	2	1	1	1	1	2	2
0.45323	0.72165	0.66474	0.63666	2	2	1	2	2	2	2	2	2	2	2	2	2	2
0.74738	0.73318	0.56947	0.64010	1	1	1	1	1	2	2	1	1	1	2	1	1	2
0.69267	0.20998	0.58056	0.64424	1	2	2	2	2	2	2	1	1	1	1	1	1	2
0.31151	0.62944	0.66793	0.64575	2	2	2	1	2	1	2	2	1	1	1	1	2	1
0.28894	0.31244	0.11760	0.64726	1	1	1	1	2	2	2	2	2	2	2	2	2	2

0.75630	0.72107	0.37123	0.65033	2	2	2	1	2	1	2	2	2	2	1	2	2	2
0.64816	0.54024	0.54676	0.65294	2	1	2	2	2	2	2	1	1	1	1	1	1	2
0.31724	0.46718	0.05279	0.66632	2	1	1	1	1	1	1	1	1	1	1	1	1	1
0.49567	0.88568	0.89621	0.66718	2	2	1	2	2	2	1	2	2	2	1	1	1	1
0.92380	0.44497	0.57023	0.66831	2	1	1	1	2	2	2	2	2	2	2	2	2	1
0.78421	0.67261	0.12155	0.67744	1	2	2	2	2	2	2	2	2	2	2	2	2	2
0.44956	0.97680	0.93849	0.68087	2	2	2	1	2	1	2	2	1	2	1	2	1	2
0.24040	0.31426	0.83385	0.68143	2	2	2	1	2	1	2	1	1	1	1	1	2	1
0.38539	0.51855	0.58188	0.68264	1	1	2	2	1	1	1	2	2	2	2	2	2	2
0.48863	0.63370	0.99441	0.68275	1	1	2	2	1	1	1	1	2	1	2	2	2	2
0.61450	0.37463	0.43538	0.68514	2	1	1	1	2	1	1	1	1	1	1	1	1	2
0.63891	0.26040	0.13974	0.68696	1	1	1	1	1	1	1	1	1	1	1	1	1	1
0.63261	0.23599	0.89787	0.69083	2	2	2	2	1	1	1	2	1	1	1	2	1	2
0.86066	0.94288	0.24754	0.69542	2	1	1	1	2	2	2	2	1	1	1	1	2	2
0.70015	0.20575	0.64898	0.69998	1	2	1	1	1	1	1	1	1	1	1	1	1	2
0.43979	0.62320	0.19568	0.70263	1	1	2	2	1	1	1	1	2	1	2	2	1	2
0.59860	0.68870	0.33312	0.70458	2	2	1	1	1	1	2	1	1	1	1	1	1	1
0.66098	0.70674	0.31839	0.70618	2	2	2	1	2	2	2	2	2	2	1	2	2	2
0.86569	0.71189	0.50283	0.71516	2	1	1	2	1	2	1	1	2	1	2	1	2	2
0.90813	0.50695	0.85852	0.71645	1	1	1	1	1	1	1	1	2	1	2	1	1	2
0.58835	0.98207	0.46119	0.71713	1	1	1	2	1	1	1	1	2	1	1	2	1	1
0.44037	0.09221	0.16032	0.71827	1	1	1	1	1	1	1	1	1	1	1	1	1	1
0.77949	0.98788	0.56437	0.72061	2	1	1	1	1	1	1	1	1	1	2	1	1	2
0.11755	0.57153	0.17146	0.72138	2	2	2	2	2	2	1	2	1	1	1	1	1	1
0.99586	0.78611	0.59220	0.72381	2	2	2	2	1	2	1	1	2	2	2	2	1	1
0.85556	0.47706	0.75312	0.72633	1	1	2	1	2	2	2	1	1	1	1	1	1	2
0.97809	0.63278	0.35334	0.73262	1	2	1	1	2	1	2	1	1	1	1	1	1	1
0.14301	0.66515	0.74595	0.74213	2	1	1	2	2	2	1	1	1	2	2	2	2	2
0.61639	0.40414	0.55539	0.74404	1	1	1	1	1	2	2	1	1	1	1	1	1	1
0.78722	0.39506	0.23889	0.74864	1	1	1	1	1	2	1	1	1	1	2	1	1	1
0.81710	0.33888	0.97661	0.75085	1	1	1	2	1	1	1	1	2	2	1	2	1	2
0.45673	0.38219	0.65026	0.75169	1	1	1	1	1	1	1	1	1	1	2	1	2	1
0.16777	0.24155	0.84378	0.75633	2	2	2	1	2	1	2	1	1	2	1	1	2	1
0.80583	0.73200	0.80070	0.75812	2	2	2	2	2	1	2	2	2	2	1	2	2	2
0.65650	0.37598	0.68684	0.75885	2	1	2	2	2	2	2	2	2	2	2	2	2	2
0.90903	0.50204	0.33584	0.76159	1	1	1	1	1	1	1	1	1	1	2	1	1	2
0.54933	0.94858	0.93383	0.76213	1	1	1	1	1	1	1	1	1	1	2	1	1	2
0.64935	0.65969	0.52255	0.76940	1	1	2	1	1	1	2	1	1	1	2	1	1	2
0.56054	0.46238	0.18797	0.77144	1	1	1	1	1	1	1	1	1	1	1	1	1	1
0.73359	0.88668	0.94108	0.77168	1	2	1	2	1	1	1	1	2	2	1	2	2	1
0.15118	0.45134	0.97965	0.77289	2	2	2	1	2	2	2	1	1	1	1	1	2	1
0.84702	0.59205	0.22603	0.77354	1	1	1	1	1	1	1	1	2	1	2	2	2	2
0.39725	0.28907	0.26602	0.77791	1	1	1	1	1	1	1	1	1	1	1	1	1	1
0.30844	0.29100	0.06935	0.78833	1	1	1	2	1	1	1	1	1	1	1	1	1	1
0.62128	0.76042	0.69216	0.79263	2	1	1	1	2	2	2	1	2	2	1	2	2	2
0.81966	0.33717	0.26096	0.79270	1	1	1	1	1	1	2	1	1	1	1	1	1	1
0.77886	0.47949	0.67256	0.79374	1	1	1	1	1	1	2	1	1	1	1	1	1	2
0.83323	0.72723	0.82975	0.79688	2	2	2	2	2	2	1	2	2	2	2	2	2	2
0.22766	0.60932	0.25381	0.79790	2	2	2	1	1	1	2	1	1	1	1	1	1	2
0.88220	0.93651	0.84978	0.79818	1	1	1	1	1	1	1	1	2	1	2	1	2	2
0.49888	0.21839	0.50287	0.80353	2	2	2	2	2	2	2	2	2	2	1	2	2	2
0.68531	0.63358	0.60049	0.81186	1	1	1	1	2	2	2	1	1	1	1	1	1	1
0.95387	0.65452	0.93956	0.81897	1	1	1	1	1	2	1	1	1	1	1	1	1	2
0.66946	0.66012	0.42044	0.82346	2	2	2	1	2	1	2	2	1	2	1	2	2	2
0.25930	0.25136	0.64412	0.82466	2	2	2	1	2	1	2	1	1	1	1	1	1	1
0.62225	0.45171	0.19726	0.82842	1	1	1	2	1	2	1	1	1	1	1	1	1	1

0.65852	0.79800	0.46372	0.84113	1	1	1	2	1	2	1	1	1	2	2	2	1	1
0.59447	0.64883	0.69726	0.84839	1	1	1	1	1	1	2	2	2	2	2	2	2	2
0.84168	0.17058	0.57739	0.84846	2	2	2	2	2	2	2	2	2	2	1	2	2	2
0.79710	0.45258	0.27331	0.85652	1	1	1	1	1	2	2	1	1	1	1	1	1	1
0.34902	0.79436	0.67785	0.85707	2	2	2	1	2	1	1	2	1	2	1	1	2	1
0.47531	0.56182	0.44848	0.85960	2	1	1	2	1	1	1	1	2	1	1	2	1	1
0.93305	0.79679	0.88187	0.86123	1	1	1	1	1	2	1	1	2	1	2	1	2	2
0.86888	0.53992	0.24282	0.86898	1	1	2	1	1	1	2	2	1	2	2	1	1	2
0.88179	0.39026	0.32762	0.86918	1	1	2	1	2	2	2	2	2	2	2	2	2	2
0.23590	0.39999	0.62217	0.87190	2	2	2	2	2	1	1	2	2	2	2	2	2	2
0.21787	0.88787	0.80884	0.87426	1	1	1	1	1	1	1	1	2	2	2	2	1	2
0.89233	0.59379	0.76765	0.88013	1	2	2	1	2	2	2	1	1	1	1	1	1	2
0.60577	0.73506	0.51629	0.88408	1	1	1	1	1	1	1	1	1	1	1	1	1	1
0.82090	0.92762	0.62348	0.88711	1	1	1	1	1	1	1	1	1	1	1	2	1	1
0.73096	0.42285	0.31758	0.88751	1	2	1	2	1	2	2	1	1	1	2	1	1	2
0.90835	0.74683	0.76695	0.89751	2	2	1	2	2	1	1	2	2	1	1	2	1	2
0.48415	0.44100	0.35153	0.89909	1	2	2	2	2	2	1	1	2	1	2	2	1	1
0.62330	0.40725	0.77087	0.90077	1	1	2	1	1	2	2	1	1	1	1	1	1	1
0.63197	0.54863	0.20850	0.90427	1	1	2	2	1	1	1	1	1	1	1	1	1	2
0.94254	0.21818	0.12337	0.91437	1	1	2	1	1	1	2	1	1	1	2	1	1	2
0.63883	0.37254	0.75522	0.91863	2	2	1	1	2	2	2	2	1	1	1	1	2	1
0.27696	0.56964	0.32037	0.92066	2	2	2	2	1	2	2	1	2	2	1	1	1	2
0.52225	0.46672	0.75939	0.92400	2	2	1	1	1	1	2	2	1	1	1	1	2	1
0.76577	0.81279	0.87818	0.92489	1	1	1	2	1	1	1	1	2	1	2	2	1	2
0.82308	0.67722	0.22087	0.92963	2	2	2	2	2	1	1	2	1	2	2	1	1	1
0.80915	0.86134	0.23647	0.93192	2	2	2	2	2	1	2	2	1	1	1	2	1	1
0.87862	0.58779	0.85078	0.93230	1	2	1	2	1	1	1	1	2	2	1	2	1	2
0.28781	0.97891	0.32885	0.93391	2	2	2	1	2	2	2	1	1	1	1	2	2	2
0.72421	0.96112	0.66934	0.93437	1	1	1	2	1	1	1	1	2	1	1	2	1	1
0.68296	0.72768	0.38451	0.93493	1	1	1	1	1	1	1	1	1	1	2	1	1	1
0.96802	0.36110	0.55639	0.93508	1	1	1	1	1	2	2	1	1	1	1	1	1	1
0.31401	0.66954	0.82596	0.93713	1	1	1	1	1	1	1	1	2	1	2	1	1	2
0.58488	0.97692	0.90625	0.94689	1	2	1	2	2	2	1	1	2	2	1	2	1	1
0.79518	0.35395	0.53953	0.94941	2	1	2	2	2	2	2	2	1	2	1	2	1	1
0.89530	0.49696	0.74325	0.95661	1	2	2	2	1	1	2	2	2	2	1	2	1	1
0.62941	0.12448	0.49998	0.95875	2	1	2	1	2	2	2	2	2	2	2	2	2	1
0.44399	0.28068	0.49653	0.95899	1	1	1	1	2	2	1	2	2	2	2	2	2	2
0.71961	0.67620	0.66305	0.96465	1	1	2	2	2	2	1	1	2	1	1	1	1	1
0.59899	0.15455	0.70137	0.97013	1	2	1	1	1	2	2	1	1	1	1	1	1	2
0.83159	0.64586	0.59191	0.97045	1	1	1	2	1	1	1	1	2	1	1	2	1	2
0.54234	0.76255	0.52063	0.97261	2	2	2	1	2	1	2	2	1	2	1	2	2	1
0.37343	0.86727	0.65047	0.97475	2	1	2	1	2	1	1	2	1	2	1	1	2	2
0.36266	0.25674	0.61589	0.97505	2	2	1	1	1	1	1	1	2	2	2	2	2	1
0.90089	0.44475	0.65179	0.97923	1	1	2	1	1	2	2	1	1	1	1	1	1	1
0.38780	0.75378	0.78113	0.98262	1	1	1	2	1	1	1	1	2	2	2	2	1	2
