# Supplementary material for: Longitudinal study on circulating miRNAs in patients after lung cancer resection
Source: Oncotarget. 2015 May 29;6(18):16674–85. doi: 10.18632/oncotarget.4322 (PMC4599298; doi:10.18632/oncotarget.4322)
Supplement: Supplementary file 6 [file oncotarget-06-16674-s006.pdf]

# Lung Cancer Patients

| Patient | gender | age at resection | tumor type | TNM | metastases during follow-up? | COPD Gold | py (smoker) | radio therapy (RT) and/or chemo therapy (ChT) |
|---|---|---|---|---|---|---|---|---|
| A | female | 79 | Squamous Cell Lung Cancer | T2N0 | no metastases | II | 35 | no ChT |
| B | male | 73 | Squamous Cell Lung Cancer | T1N0 | no metastases | II | 50 | no ChT |
| C | female | 48 | Adenocarcinoma | YPT0YPN0 | no metastases | 0 | 30 | pre-surgical RT and ChT |
| D | male | 65 | Squamous Cell Lung Cancer | T2N0 | no metastases | II | 60 | no ChT |
| E | male | 63 | Adenocarcinoma | T1N0 | no metastases | 0 | never smoker | no ChT |
| F | male | 57 | Squamous Cell Lung Cancer | T2N0 | metastases | 0 | 20 | no ChT |
| G | male | 71 | Squamous Cell Lung Cancer | T2N0 | no metastases | 0 | never smoker | pre-surgical RT and ChT |
| H | female | 69 | Adenocarcinoma | T1N2 | metastases | 0 | | post-surgical ChT (only 1 cycle) |
| I | male | 79 | Squamous Cell Lung Cancer | T2N0 | no metastases | II | <15 | no ChT |
| J | male | 58 | Adenocarcinoma | *T2N0* | no metastases | II | 80 | no ChT |
| K | male | 66 | Squamous Cell Lung Cancer | T2N1R1 | no metastases | I | 35 | no ChT |
| L | male | 52 | Squamous Cell Lung Cancer | T2N1 | no metastases | II | 40 | no ChT |
| M | male | 55 | Squamous Cell Lung Cancer | *T2N0* | no metastases | 0 | 80 | no ChT |
| N | male | 47 | Squamous Cell Lung Cancer | T1N0 | no metastases | 0 | 50 | no ChT |
| O | female | 58 | Adenosquamous Lung Cancer | T1N0 | no metastases | 0 | 25 | no ChT |
| P | female | 77 | Adenocarcinoma | T2N0 | no metastases | I | never smoker | no ChT |
| Q | female | 54 | Adenocarcinoma | T1N0 | no metastases | 0 | 35 | no ChT |
| R | female | 70 | Adenocarcinoma | T1N0 | no metastases | 0 | never smoker | no ChT |
| S | female | 59 | Squamous Cell Lung Cancer | T1N0 | no metastases | 0 | 20 | no ChT |
| T | male | 69 | Adenosquamous Lung Cancer | T2N1 | metastases | 0 | 20 | post-surgical ChT |
| U | male | 61 | Squamous Cell Lung Cancer | T1N0 | no metastases | I | 40 | no ChT |
| V | male | 64 | Adenocarcinoma | *T2N1* | metastases | II | 30 | no ChT |
| W | female | 69 | Adenocarcinoma | T1N0 | metastases | 0 | 30 | no ChT |
| X | male | 70 | Bronchioloalveolar carcinoma | -- | metastases | 0 | 15 | no ChT |
| Y | male | 59 | Adenocarcinoma | T2N2 | metastases | II | 40 | post-surgical ChT |
| Z | male | 56 | Squamous Cell Lung Cancer | *T3N1* | metastases | II | 40 | no ChT |

# Non-Cancer Control Patients

| Patient | gender | age | disease |
|---|---|---|---|
| control 1 | male | 57 | pleurisy |
| control 2 | male | 52 | chronic pleurisy |
| control 11 | female | 57 | echinococcus |
| control 12 | male | 68 | pulmonary infarction |
| control 3 | male | 58 | chronic pneumonia |
| control 4 | male | 58 | pneumonia |
| control 5 | male | 47 | anthracosilicosis |
| control 6 | female | 45 | anthracosis |
| control 7 | male | 61 | tuberculoma |
| control 8 | female | 37 | tuberculoma |
| control 9 | male | 42 | air cyst |
| control 10 | male | 47 | air cyst |
